# Synthesis and biological activities of novel trifluoromethylpyridine amide derivatives containing sulfur moieties†

**DOI:** 10.1039/d0ra07301f

**Published:** 2020-09-28

**Authors:** S. X. Guo, F. He, A. L. Dai, R. F. Zhang, S. H. Chen, J. Wu

**Affiliations:** State Key Laboratory Breeding Base of Green Pesticide and Agricultural Bioengineering, Key Laboratory of Green Pesticide and Agricultural Bioengineering, Ministry of Education, Research and Development Center for Fine Chemicals, Guizhou University Huaxi District Guiyang 550025 P. R. China wujian2691@126.com jwu6@gzu.edu.cn

## Abstract

A series of trifluoromethylpyridine amide derivatives containing sulfur moieties (thioether, sulfone and sulfoxide) was designed and synthesized. Their antibacterial activities against *Xanthomonas oryzae* pv. *oryzae* (*Xoo*), *Ralstonia solanacearum* (*R. solanacearum*) and insecticidal activities against *P. xylostella* were evaluated. Notably, the half-maximal effective concentration (EC_50_) value of sulfone-containing compound F10 is 83 mg L^−1^ against *Xoo*, which is better than that of commercial thiodiazole copper (97 mg L^−1^) and bismerthiazol (112 mg L^−1^). Thioether-containing compounds E1, E3, E5, E6, E10, E11 and E13 showed much higher activities against *R. solanacearum* with the EC_50_ value from 40 to 78 mg L^−1^, which are much lower than that of thiodiazole copper (87 mg L^−1^) and bismerthiazol (124 mg L^−1^). Generally, most of the sulfone-containing compounds and sulfoxide-containing compounds showed higher activities against *Xoo* than that of the corresponding thioether-containing compound, but most of the thioether-containing compounds contributed higher antibacterial activities against *R. solanacearum*. Furthermore, title compounds E3, E11, E24 and G2 showed good insecticidal activities of 75%, 70%, 70% and 75%, respectively.

## Abstract 2

Novel trifluoromethylpyridine amide derivatives containing sulfur moieties were synthesized, which exhibited good antibacterial and insecticidal activities for potential crop protection agents.

## Graphical abstract

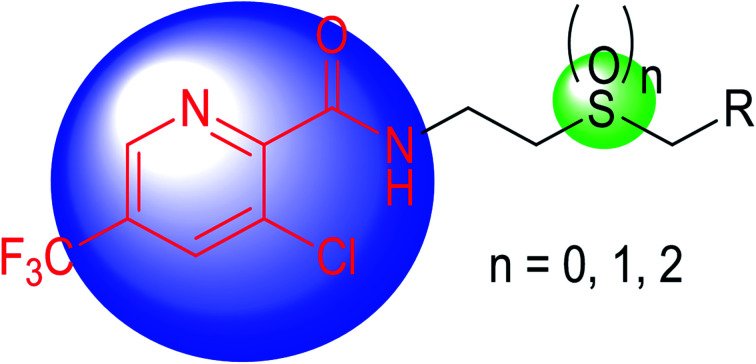

## 1 Introduction

Crop diseases caused by bacteria, fungi, viruses, nematodes and oomycetes have posed a huge challenge for crop production, so that it is hard to provide sufficient food for the growing population.^1,2^ Particularly, rice bacterial leaf blight caused by *Xanthomonas oryzae* pv. *oryzae* (*Xoo*) can reduce rice yields by 80%.^3,4^ Tobacco bacterial wilt caused by *Ralstonia solanacearum* (*R. solanacearum*), is another devastating disease.^5^ Pesticides play a crucial role in controlling crop diseases for agricultural cultivating systems, rapidly increasing the crop yields and food production.^1,6^ However, along with the resistance and cross resistance, the efficiencies of many pesticides have gradually reduced. Currently, because of the potential environmental, ecological and health risks, some pesticides have been gradually banned and withdrawn from the market. For example, bismerthiazol, used as a bactericide against rice bacterial blight, was banned by the Ministration of Agriculture, P. R. China due to its harmful effects towards some creatures. Consequently, the development of eco-friendly pesticides with novel action modes is urgently needed.

**Fig. 1:**
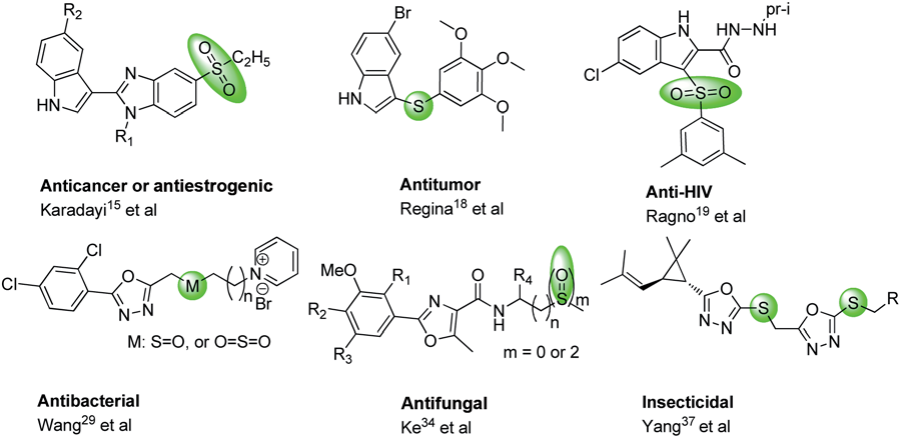
Some bio-active molecules containing thioether, sulfoxide or sulfone.

Sulfur element is a crucial part of proteins and amino acids. It can be found in many secondary metabolites in living organisms, and exits also in many bio-active compounds (Fig. 1) with broaden biological activities,^7–14^ especially using for anti-cancer,^15–18^ anti-HIV,^19,20^ and treating acid-related disorder,^21^ and also using for the inhibitors of topoisomerase 1,^22^ anhydrase II,^23^ mutated B-Raf,^24,25^ cyclooxygenase-2.^26,27^ Particularly, compounds containing thioether, sulfone or sulfoxide moieties could exhibit significantly anti-bacterial,^28–32^ anti-fungal,^33,34^ herbicidal^35^ and insecticidal^36,37^ activities for crop protection. Thus, sulfur-containing molecules show promising properties for drug design and medicinal chemistry.^38–40^

Over decades, trifluoromethylpyridine ring, a special fluorinated nitrogen heterocycle, has been a hot spot for the creation of novel pesticides.^41–43^ For the period 2000–2017, among a total 166 ISO common name proposals, 10 (6%) contain a trifluoromethylpyridine, including 3 fungicides, 2 herbicides and 5 insecticides.^43^ Our previous works^44–46^ also revealed that some compounds containing trifluoromethyl pyridine showed excellent anti-virus, anti-bacterial and insecticidal activities.

Consequently, considering the concepts mentioned above, this work focused on the synthesis of trifluoromethylpyridine amide derivatives containing the thioether, sulfoxide or sulfone substructure, and their biological evaluation. Their primary structure–activity relationship for these novel trifluoromethylpyridine amide derivatives was also discussed.

## 2 Results and discussion

### 2.1 Design

**Fig. 2:**
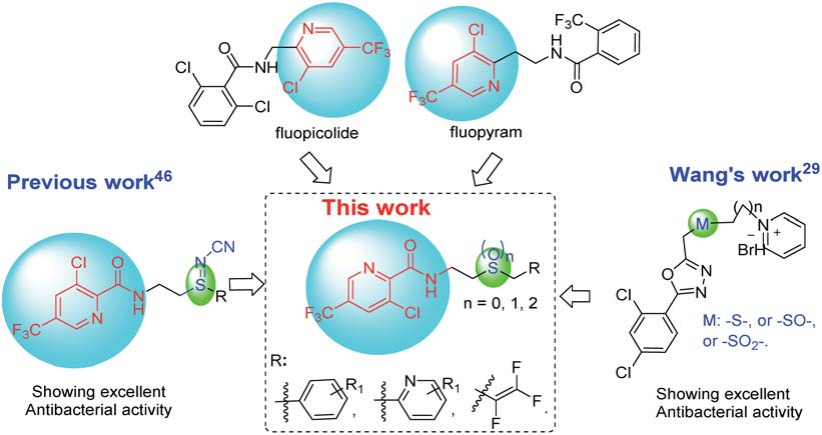
The design of the title compounds.

**Scheme 1:**
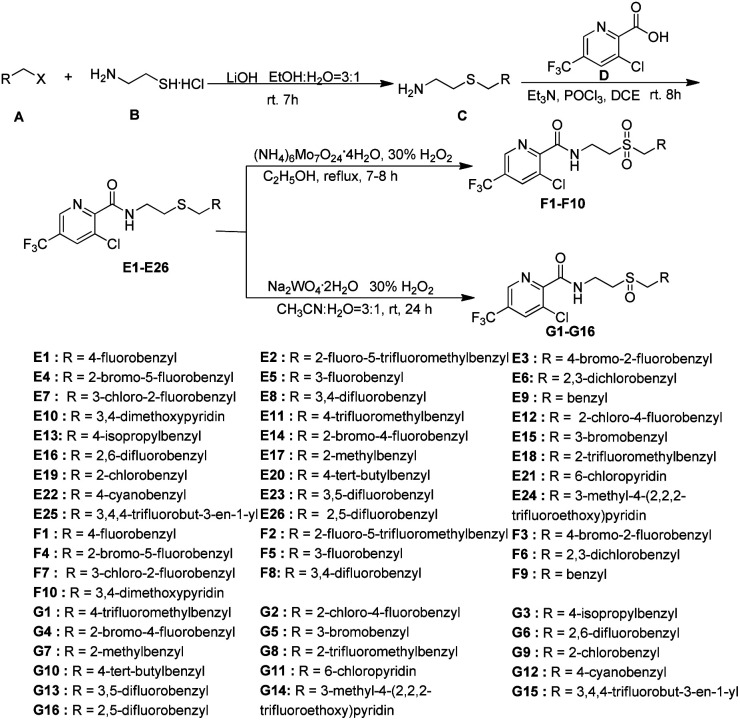
The synthetic route of the title compounds E1–E26, F1–F10, and G1–G16.

The commercial fungicides such as fluopicolide and fluopyram (Fig. 2) are typical trifluoromethylpyridine derivatives.^43^ Our previous work has revealed that trifluoromethyl pyridinamide derivatives containing a structure of “–S=N–CN” showed significantly bactericidal activities.^46^ Wang and co-workers^29^ has reported that some novel sulfur contained compounds can be used as potential antibacterial agents.^29^ Thus as shown in Fig. 2, this work sought to modify the previous structure through changing the “–S=N–CN” to “–S–”, “–SO^2^–” or “–SO–” to obtain a series of novel thioether-containing compounds E1–E26. Then, the chemoselectivities of sulfone-containing compounds F1–F10 and sulfoxide-containing compounds G1–G16, were obtained by the oxidation of compounds E1–E26 in different conditions (Scheme 1). Their antibacterial activities against *Xoo* and *R. solanacearum*, and insecticidal activities against *P. xylostella* were evaluated. Desirably, some synthesized compounds could show higher antibacterial activities than that of commercialized thiodiazole copper and bismerthiazol. It's the first time that trifluoromethylpyridine amide derivatives containing the sulfur moiety were designed and synthesized for anti-bacterial and insecticidal applications.

### 2.2 Chemistry

According to the reported methods,^29,47,48^ the title compounds E1–E26, F1–F10 and G1–G16 could be easily obtained as shown in Scheme 1. The thioether-containing compounds E1–E26 were firstly synthesized *via* two steps. Firstly, haloalkane (A) was treated with aminoethyl mercaptan (B) using LiOH as base and the mixture of ethanol and H_2_O in a ratio of 3 : 1 as solvent under room temperature (rt) to obtain differently substituted 2-(ethylthio)amines (C).^29,47,48^ After being activated by POCl_3_, 3-chloro-5-(trifluoromethyl)picolinic acid (D) condensed with intermediate C to obtain thioether-containing compounds E1–E26.^29,47,48^ Thioether-containing compounds E1–E10 were then converted to corresponding sulfone-containing compounds F1–F10 in the presence of ammonium molybdate, 30% H_2_O_2_ as oxidant and ethanol as solvent at 100 °C.^47,48^ Different with the synthesis of sulfone compounds F1–F10, sulfoxide-containing compounds G1–G16 were obtained through the oxidation of E11–E26 in the presence of sodium tungstate dehydrate, 30% H_2_O_2_ as oxidant and the mixture of acetonitrile and water in a ratio of 3 : 1 as solvent at rt.^47,48^

The title thioether-containing compounds E1–E26, sulfone-containing compounds F1–F10 and sulfoxide-containing compounds G1–G16 were characterized by the ^1^H NMR, ^19^F NMR, ^13^C NMR and HR-MS. Taking sulfone-containing compound F1 as an example, in the ^1^H NMR spectrum, the proton near “N” atom of trifluoromethylpyridine ring appeared as a doublet at *δ* 8.72 ppm. The proton of –CONH– was split into a triplet at *δ* 8.40 ppm. Another proton of trifluoromethylpyridine ring appeared also as a doublet at *δ* 8.07. The four protons of benzene ring were characterized as two multiples at *δ* 7.33–7.23 and *δ* 7.15–7.00. The protons of methylene near the benzene ring appeared as a quartet at *δ* 4.03. The protons of methylene near the carbonyl appeared as multiple at *δ* 3.11–2.77, and the protons of methylene appeared at *δ* 3.96 as double doublets. In ^13^C NMR spectrum, the carbons near “–CF_3_” or “F” were all split into quartets due to the coupling coefficients of “F”. For instance, the carbon of “–CF_3_” group was split into a quartet at *δ*_C_ 122.1 ppm with the coupling constant of (^1^*J*_F–C_) 273.3 Hz. Carbon at the position 5 in the pyridine, was split into another quartet with coupling constant of 34.1 Hz at *δ*_C_ 129.4. Two carbons at the position 4 and 6 of trifluoromethylpyridine, were also split into two quartets with smaller coupling constants. Other carbons near the atom “F” could also be split with different coupling constants. In the ^19^F NMR spectra, the fluorine of “–CF_3_” appeared at the shift of −62.57 ppm, and the fluorine on the benzene ring appeared at the shift of −112.80 ppm.

### 2.3 Preliminary *in vitro* antibacterial activity test

According to the reported method,^49^ the method of turbidity was adopted to evaluate the antibacterial activities of title compounds against *Xanthomonas oryzae* pv. *oryzae* (*Xoo*), *Ralstonia solanacearum* (*R. solanacearum*). The commercial thiodiazole copper (TC) and bismerthiazol (BT) were used as the positive controls. The results shown in Table 1 revealed that some of the synthesized compounds showed higher activities against *Xoo* and *R. solanacearum* than that of commercial bactericides at the concentration of 100 and 50 mg L^−1^. For example, at the concentration of 50 mg L^−1^, compounds E20 (R is 4-*tert*-butylbenzyl), E15 (R is 3-bromobenzyl), E9 (R is benzyl), E16 (R is 2,6-difluorobenzyl), E13 (R is 4-isopropylbenzyl) and E24 (R is 3-methyl-4-(2,2,2-trifluoroethoxy)pyridine) showed good activities against *Xoo* of 35%, 35%, 33%, 33%, 32% and 31% respectively, which are close to that of BT (31%). Desirably, when two electron withdraw groups, 3,4-dimethoxypyridine, substituted in the phenyl, sulfone-containing compound F10 exhibited the highest activities of 53% (100 mg L^−1^) and 42% (50 mg L^−1^) against *Xoo*, which are slightly higher than that of TC (53%, 39%) and BT (51%, 31%). Sulfone-containing compounds F1–F10 showed the different activities against *Xoo* as follows: 3,4-dimethoxypyridin (F10) > benzyl (F9) > 4-bromo-2-fluorobenzyl (F3) > 4-fluorobenzyl (F1) > 3-fluorobenzyl (F5) > 2,3-dichlorobenzyl (F6) > 2-fluoro-5-trifluoromethylbenzyl (F2) > 2-bromo-5-fluorobenzyl (F4) > 3-chloro-2-fluorobenzyl (F7) > 3,4-difluorobenzyl (F8). Among sulfone-containing compounds, compounds F3 (31%), F5 (31%) and F9 (32%) showed activities of 31%, 31% and 32% respectively against *Xoo* at 50 mg L^−1^, which are close to than that of BT (31%). Among sulfoxide-containing compounds, compounds G5 where R is 3-bromobenzyl and G16 where R is 2,5-difluorobenzyl showed activities of 32% and 35%, respectively against *Xoo* at 50 mg L^−1^, which are closed to that of BT (31%). The majority of oxidized compounds (sulfone or sulfoxide-containing compounds) could show higher activities against *Xoo*, compared with the thioether-containing compounds. For example, oxidizing thioether-containing compounds E3 (9%), E4 (13%), E7 (7%) and E17 (4%) to corresponding F3 (47%), F4 (32%), F7 (31%) and G7 (36%) could provide 38%, 26%, 24% and 32% higher activities against *Xoo* at 100 mg L^−1^, respectively. However, compounds E8 (32%), E22 (42%) and E24 (38%) showed higher activities against *Xoo* than that of corresponding oxidized compounds F8 (19%), G9 (18%) and G24 (28%). Thioether-containing compounds E13 (39%), E19 (44%), E20 (43%) and E23 (42%) showed slightly higher activities against *Xoo* at 100 mg L^−1^ than that of corresponding oxidized compounds G3 (35%), G9 (37%), G10 (33%) and G13 (39%). The rest of oxidized compounds owned higher activities against *Xoo* than that of corresponding thioether-containing compounds. Regarding *R. solanacearum*, thioether-containing compounds E1 (57%, 54%), E3 (53%, 44%), E5 (64%, 50%), E6 (67%, 52%), E10 (62%, 45%), E11 (54%) and E13 (63%, 56%) exhibited higher activities than that of TC (51%, 40%) and much higher activities than that of BT (38%, 17%) at 100 or 50 mg L^−1^. According to their activities, the R groups of them can be sorted as follows: 2,3-dichlorobenzyl (E6) > 3-fluorobenzyl (E5) > 4-isopropylbenzyl (E13) > 3,4-dimethoxypyridin (E10) > 4-fluorobenzyl (E1) > 4-trifluoromethylbenzyl (E11) > 4-bromo-2-fluorobenzyl (E3). At 100 or 50 mg L^−1^, there are other thioether-containing compounds showing higher activities against *R. solanacearum* than that of BT (38%, 17%), including R groups are 3-methyl-4-(2,2,2-trifluoroethoxy)pyridine (E24, 50%, 28%), 2-trifluoromethylbenzyl (E18, 49%, 33%), 3-chloro-2-fluorobenzyl (E7, 48%, 45%), 2-fluoro-5-trifluoromethylbenzyl (E2, 44%, 30%), 3,5-difluorobenzyl (E23, 40%, 32%), 2-bromo-4-fluorobenzyl (E14, 40%) and 2-methylbenzyl (E17, 40%, 24%). Sulfone-containing compounds F4 (43%) and F5 (48%) could also show higher activities than that of BT against *R. solanacearum*. Among the sulfoxide-containing compounds, compounds G14 (43%) and G15 (42%) showed higher activities against *R. solanacearum* than that of BT. Their R groups are 3-methyl-4-(2,2,2-trifluoroethoxy)pyridine and 3,4,4-trifluorobut-3-en-1-yl, respectively. Sulfone-containing compound F4 (43%) and sulfoxide-containing compounds G6 (36%), G10 (37%), G15 (42%), G16 (25%) showed higher activities against *R. solanacearum* than that of corresponding thioether-containing compounds E4 (30%), E16 (30%), E20 (5%), E25 (3%) and E26 (10%). But the rest of sulfone-containing compounds or sulfoxide-containing compounds exhibited much lower activities than that of corresponding thioether-containing compounds, which is totally different with the activities against *Xoo*. Particularly, when R is 2,3-dichlorobenzyl, thioether-containing compound E6 could show the highest activity (67%), which is much higher than that of TC and BT, but the corresponding oxidized sulfone-containing compound F6 owned no activity (1%) against *R. solanacearum*. In addition, thioether-containing compound E13 also exhibited much higher activity (63%) against *R. solanacearum* than that of both TC and BT, however, sulfoxide-containing compound G3, the oxidized product of E13, are almost no activity against *R. solanacearum*.

**Table 1:** Antibacterial activities of title compounds E1–E26, F1–F10 and G1–G16 against *Xoo* and *R. solanacearum*

Compounds	Activity^a^ (%)				Compounds	Activity^a^ (%)			
	*Xoo*		*R. solanacearum*			*Xoo*		*R. solanacearum*	
	100 mg L^−1^	50 mg L^−1^	100 mg L^−1^	50 mg L^−1^		100 mg L^−1^	50 mg L^−1^	100 mg L^−1^	50 mg L^−1^
E1	40 ± 1.2	18 ± 2.4	57 ± 0.3	54 ± 0.8	F1	41 ± 1.9	29 ± 1.4	30 ± 1.0	25 ± 4.1
E2	26 ± 0.3	16 ± 3.0	44 ± 3.0	30 ± 1.3	F2	33 ± 1.6	28 ± 2.3	10 ± 1.1	—
E3	9 ± 2.7	—	53 ± 2.5	44 ± 3.3	F3	47 ± 3.5	31 ± 0.4	9 ± 1.8	—
E4	13 ± 0.5	2 ± 1.2	30 ± 1.9	10 ± 2.3	F4	32 ± 2.8	22 ± 0.8	43 ± 1.8	18 ± 2.8
E5	27 ± 1.5	21 ± 2.3	64 ± 4.4	50 ± 3.5	F5	45 ± 3.8	31 ± 1.7	48 ± 4.8	45 ± 3.5
E6	14 ± 0.8	10 ± 4.2	67 ± 2.2	52 ± 4.7	F6	38 ± 2.2	24 ± 2.3	1 ± 0.1	—
E7	7 ± 2.2	—	48 ± 1.6	45 ± 2.9	F7	31 ± 0.3	21 ± 0.3	28 ± 2.6	14 ± 5.0
E8	32 ± 0.5	16 ± 2.4	8 ± 5.0	—	F8	19 ± 0.1	21 ± 1.4	21 ± 3.4	12 ± 0.3
E9	45 ± 4.4	33 ± 1.7	50 ± 4.0	11 ± 3.4	F9	47 ± 3.5	32 ± 3.1	32 ± 1.8	21 ± 1.1
E10	29 ± 0.3	10 ± 1.6	62 ± 2.3	45 ± 3.6	F10	53 ± 4.2	42 ± 4.8	36 ± 1.9	34 ± 3.3
E11	16 ± 4.9	7 ± 2.3	54 ± 2.0	27 ± 4.5	G1	28 ± 1.5	29 ± 2.0	2 ± 1.3	—
E12	14 ± 4.3	4 ± 2.4	50 ± 3.5	40 ± 1.2	G2	29 ± 1.6	20 ± 2.8	15 ± 2.3	2 ± 0.3
E13	39 ± 2.6	32 ± 4.8	63 ± 3.3	56 ± 2.0	G3	35 ± 1.2	17 ± 2.4	14 ± 1.7	8 ± 0.3
E14	35 ± 4.7	30 ± 1.0	40 ± 2.2	14 ± 4.0	G4	35 ± 3.0	24 ± 2.6	28 ± 2.0	21 ± 1.5
E15	41 ± 2.3	35 ± 4.5	7 ± 3.1	—	G5	48 ± 2.0	32 ± 1.0	19 ± 2.0	14 ± 3.5
E16	43 ± 2.7	33 ± 0.4	30 ± 2.1	14 ± 3.0	G6	46 ± 2.2	30 ± 0.9	36 ± 4.3	11 ± 3.5
E17	4 ± 1.5	—	40 ± 1.1	24 ± 0.7	G7	36 ± 2.3	26 ± 0.2	25 ± 3.1	18 ± 3.2
E18	23 ± 1.6	3 ± 0.9	49 ± 3.4	33 ± 4.4	G8	44 ± 4.9	24 ± 5.0	26 ± 0.4	23 ± 1.5
E19	44 ± 1.7	26 ± 0.5	34 ± 3.8	27 ± 5.0	G9	37 ± 4.4	20 ± 3.5	34 ± 0.3	29 ± 1.2
E20	43 ± 1.7	35 ± 3.6	5 ± 1.0	—	G10	33 ± 2.2	23 ± 3.8	37 ± 5.0	20 ± 1.1
E21	35 ± 2.7	21 ± 2.1	23 ± 3.9	9 ± 0.8	G11	36 ± 1.4	22 ± 4.8	2 ± 0.2	—
E22	42 ± 4.2	25 ± 4.1	34 ± 3.9	18 ± 0.5	G12	18 ± 1.1	10 ± 1.4	3 ± 1.8	—
E23	42 ± 1.6	21 ± 2.8	40 ± 3.2	32 ± 2.3	G13	39 ± 4.1	29 ± 3.9	11 ± 0.7	7 ± 0.5
E24	38 ± 4.0	31 ± 4.2	50 ± 0.3	28 ± 0.6	G14	35 ± 3.9	17 ± 0.3	43 ± 5.0	19 ± 2.3
E25	33 ± 2.6	23 ± 3.9	3 ± 0.9	—	G15	36 ± 2.2	26 ± 0.8	42 ± 3.4	38 ± 2.4
E26	28 ± 3.9	15 ± 2.0	10 ± 4.2	—	G16	40 ± 2.9	35 ± 3.8	25 ± 2.3	23 ± 0.1
Thiodiazole copper (TC)	53 ± 0.8	39 ± 4.6	51 ± 3.0	40 ± 4.3	Bismerthiazol (BT)	51 ± 3.7	31 ± 1.8	38 ± 2.5	17 ± 1.5

a The antibacterial activities are mean of three independent experiments.

### 2.4 The EC_50_ values of active title compounds against *Xoo* or *R. solanacearum*

The half-maximal effective concentration (EC_50_) values of sulfone-containing compound F10 against *Xoo*, and thioether-containing compounds E1, E3, E5, E6, E10, E11, and E13 against *R. solanacearum* were further evaluated. The results listed in Table 2 indicated the EC_50_ value against *Xoo* of sulfone-containing compound F10 is 83 mg L^−1^, which is much lower than that of TC (97 mg L^−1^) and BT (112 mg L^−1^). Furthermore, the EC_50_ values against *R. solanacearum* of thioether-containing compounds E6 and E13 are 41 mg L^−1^ and 40 mg L^−1^ respectively, which are twice lower than that of TC (87 mg L^−1^) and third lower than that of BT (124 mg L^−1^). Thioether-containing compounds E1, E3, E5, E10 and E11 had the EC_50_ values of 53, 75, 53, 78 and 73 mg L^−1^ respectively, which are also much lower than that of TC and BT.

**Table 2:** The EC_50_ values of title compounds against *Xoo* or *R. solanacearum*

Compounds	*Xoo*			Compounds	*R. solanacearum*		
	Regression equation	*R* ^2^	EC_50_^a^ (mg L^−1^)		Regression equation	*R* ^2^	EC_50_^a^ (mg L^−1^)
F10	*y* = 0.8557*x* + 3.3573	1.00	83 ± 0.1	E1	*y* = 0.7768*x* + 3.661	0.98	53 ± 0.3
Thiodiazole copper (TC)	*y* = 0.996*x* + 3.065	0.99	97 ± 1.2	E3	*y* = 2.4159*x* + 0.4676	0.94	75 ± 0.5
Bismerthiazol (BT)	*y* = 2.5861*x* − 0.0104	0.97	112 ± 1.2	E5	*y* = 1.4452*x* + 2.5102	1.00	53 ± 0.9
				E6	*y* = 2.3726*x* + 1.167	1.00	41 ± 0.2
				E10	*y* = 1.34*x* + 2.4627	0.97	78 ± 0.2
				E11	*y* = 1.3523*x* + 2.4812	0.99	73 ± 1.2
				E13	*y* = 0.9336*x* + 3.507	0.99	40 ± 2.1
				Thiodiazole copper (TC)	*y* = 1.6934*x* + 1.7154	0.94	87 ± 1.1
				Bismerthiazol (BT)	*y* = 2.0182*x* + 0.7772	0.97	124 ± 1.3

a Each experiment of EC_50_ value is performed in triplicates.

### 2.5 Insecticidal activity test

The insecticidal activities of synthesized compounds against *P. xylostella* are shown in Table 3. The chlorpyrifos and avermectin were used as positive controls. The results (Table 3) revealed that some compounds could show moderate insecticidal activities. Thioether-containing compound E3 where R is 4-bromo-2-fluorobenzyl showed the highest activity of 75% against *P. xylostella*, but the activity could sharply decrease after being oxidized into corresponding sulfone-containing compound F3 (10%). When R was changed to 4-trifluoromethylbenzyl (E11) or 3-methyl-4-(2,2,2-trifluoroethoxy)pyridine (E24), the activity could slightly decrease to 70%. Thioether-containing compounds E5 and E12 both showed the activities of 55% against *P. xylostella*, and their R groups are 3-fluorobenzyl and 2-chloro-4-fluorobenzyl, respectively. Thioether-containing compounds E1 (2-bromo-5-fluorobenzyl), E4 (4-fluorobenzyl), E7 (3-chloro-2-fluorobenzyl), E15 (3-bromobenzyl) all showed activities of 50%. The rest of thioether-containing compounds showed lower activities less than 50%. In addition, the insecticidal activities of sulfone-containing compounds could be sorted as follows: 3-chloro-2-fluorobenzyl (F7) > 2-fluoro-5-trifluoromethylbenzyl (F2) > 2-bromo-5-fluorobenzyl (F4) > 4-fluorobenzyl (F1) > 2,3-dichlorobenzyl (F6) = 3,4-difluorobenzyl (F8) = benzyl (F9) = 3,4-dimethoxypyridin (F10) > 4-bromo-2-fluorobenzyl (F3) > 3-fluorobenzyl (F5). Specially, sulfone-containing compounds F2, F7, F9 and F10 all showed higher activities than that of corresponding thioether-containing compounds E2, E7, E9 and E10, but the rest of sulfone-containing compounds all showed activities less than that of corresponding thioether-containing compounds. When R is 2-chloro-4-fluorobenzyl, sulfoxide-containing compound G2 showed activity of 75%. Sulfoxide-containing compounds G3, G6 and G7 showed the activities of 60%, 60% and 50% respectively, which are all higher than that of corresponding thioether-containing compounds E13, E16 and E17. The activities of the rest of sulfoxide-containing compounds are all less than 50%.

**Table 3:** Insecticidal activities of title compounds against *P. xylostella*

Compounds	Activity^a^ (%) at 500 mg L^−1^	Compounds	Activity^a^ (%) at 500 mg L^−1^
E1	50 ± 0	F1	35 ± 0
E2	45 ± 2.9	F2	50 ± 0
E3	75 ± 0	F3	10 ± 3.3
E4	50 ± 0	F4	40 ± 3.3
E5	55 ± 0	F5	10 ± 0
E6	40 ± 2.9	F6	30 ± 3.3
E7	50 ± 3.3	F7	60 ± 0
E8	30 ± 0	F8	30 ± 0
E9	10 ± 5	F9	30 ± 0
E10	10 ± 0	F10	30 ± 0
E11	70 ± 0	G1	30 ± 0
E12	55 ± 3.3	G2	75 ± 0
E13	30 ± 2.9	G3	60 ± 0
E14	10 ± 2.9	G4	40 ± 0
E15	50 ± 0	G5	30 ± 0
E16	20 ± 0	G6	60 ± 0
E17	40 ± 0	G7	50 ± 2.9
E18	30 ± 0	G8	20 ± 0
E19	20 ± 0	G9	20 ± 0
E20	30 ± 5	G10	10 ± 0
E21	20 ± 3.3	G11	30 ± 0
E22	30 ± 0	G12	10 ± 0
E23	35 ± 0	G13	20 ± 0
E24	70 ± 0	G14	10 ± 3.3
E25	10 ± 0	G15	40 ± 0
E26	10 ± 0	G16	30 ± 3.3
Chlorpyrifos	100 ± 0	Avermectin	100 ± 0

a The each insecticidal test were performed in triplicates.

## 3 Conclusion

A series of sulfur-containing trifluoromethylpyridine amide derivatives has been designed and synthesized. Their antibacterial activities against *Xanthomonas oryzae* pv. *oryzae* (*Xoo*), *Ralstonia solanacearum* (*R. solanacearum*) and insecticidal activities against *P. xylostella* were evaluated. Notably, sulfone-containing compound F10 (53%, 42%) showing the highest activity against *Xoo* had the EC_50_ of 83 mg L^−1^, which is much lower than that of TC (97 mg L^−1^) and BT (112 mg L^−1^). Thioether-containing compounds E1 (57%, 54%), E3 (53%, 44%), E5 (64%, 50%), E6 (67%, 52%), E9 (50%, 11%), E10 (62%, 45%), E11 (54%, 27%), and E13 (53%, 44%) showed higher activities than that of TC (51%, 40%) showed excellent antibacterial activities against *R. solanacearum* with EC_50_ values ranging from 40–73 mg L^−1^, which are all much lower than that of TC (87 mg L^−1^) and BT (124 mg L^−1^). Generally, most of oxidized compounds could show higher activities against *Xoo* than that of corresponding thioether-containing compounds, but most of thioether-containing compounds contributed higher activities against *R. solanacearum*. Furthermore, compounds E3, E11, E24 and G2 showed also moderate insecticidal activities of 75%, 70%, 70% and 75%, respectively.

## 4 Experimental section

### 4.1 Materials and methods

All reagents and solvents were purchased from Accela Chem-Bio Co., Ltd (Shanghai, China) and Innochem Co., Ltd (Beijing, China). Melting points of the synthesized compounds were measured using a XT-4 binocular microscope (Beijing Tech Instrument Co., China). Using CDCl_3_ as solvent, the spectra of ^1^H, ^19^F and ^13^C NMR of title compounds were recorded on AVANCE III HD 400M NMR (Bruker Corporation, Switzerland) spectrometer operating at room temperature. HR-MS was recorded on an Orbitrap LC-MS instrument (Q-Exative, Thermo Scientific™, and American). The course of the reactions was monitored by TLC.

### 4.2 Synthetic procedures

#### 4.2.1 Synthesis of substituted aminoethyl sulfide (C)

According to the reported literatures,^47,48^ substituted intermediate C could be easily obtained as shown in Scheme 1. Taking 2-((4-fluorobenzyl)thio)ethanamine as an example, to a mixture of LiOH (72.63 mmol) resolving in 15 mL water and EtOH (45 mL) stirred at room temperature was added 2-aminoethyl mercaptan (B, 34.58 mmol). Subsequently, 1-(chloromethyl)-4-fluorobenzene (A, 34.58 mmol) was added dropwise and stirred at room temperature for about 7 h. The reaction was monitored by TLC. After the completion of the reaction, the reaction mixture was concentrated under reduced pressure, and the residue was extracted with dichloromethane and water. The organic phase was washed with NaOH solution to provide a crude production, which was purified using silica gel (200–300 mesh) column chromatography with dichloromethane/methanol (20 : 1).

#### 4.2.2 The synthesis of thioether-containing compounds E1–E26^29,47,48^

Taking E1 as an example, 2-(((2-aminoethyl)thio)methyl)-5-fluorobenzene-1-ylium (C, 2.64 mmol), 3-chloro-5-(trifluoromethyl)picolinic acid (D, 2.64 mmol) and Et_3_N (2.64 mmol) were added in one portion using 1,2-dichloroethane (DCE, 6 mL) as solvent, which was stirred at room temperature. Subsequently, POCl_3_ diluted by DCE was added dropwise and refluxed for 8 h. The resulted mixture was concentrated under reduced pressure, and washed with Na_2_CO_3_. The resulting solid was filtrated and washed with water to provide crude product, which was purified by silica gel (200–300 mesh) column chromatography with ethyl acetate/petroleum ether (1 : 3). Along with similar method, thioether-containing compounds E2–E26 could be also obtained. The spectral data of E1–E26 are listed below, and the spectra are shown in the ESI data.†

##### 3-Chloro-*N*-(2-((4-fluorobenzyl)thio)ethyl)-5-(trifluoromethyl)picolinamide (E1)

Yield 83%; yellow solid; mp 96–97 °C. ^1^H NMR (400 MHz, CDCl_3_) *δ* 8.72 (s, 1H, pyridine-H), 8.08 (s, 1H, pyridine-H), 7.99 (s, 1H, CO–NH), 7.30 (dd, *J* = 8.4, 5.4 Hz, 2H, Ar–H), 6.98 (t, *J* = 8.6 Hz, 2H, Ar–H), 3.75 (s, 2H, –CH_2_), 3.62 (q, *J* = 6.4 Hz, 2H, –CH_2_), 2.69 (t, *J* = 6.5 Hz, 2H, –CH_2_). ^13^C NMR (100 MHz, CDCl_3_) *δ* 162.3, 161.9 (d, *J* = 245.8 Hz), 149.1, 142.8 (q, *J* = 3.8 Hz), 137.7 (q, *J* = 3.6 Hz), 133.7 (d, *J* = 3.2 Hz), 132.2, 130.4 (d, *J* = 8.1 Hz), 129.3 (q, *J* = 34.0 Hz), 122.2 (q, *J* = 273.4 Hz), 115.5 (d, *J* = 21.5 Hz), 38.4, 35.2, 30.9. ^19^F NMR (376 MHz, CDCl_3_) *δ* −62.52, −115.22. HRMS: [M + H]^+^ calcd for C_16_H_14_ClF_4_N_2_OS: 393.04460; found: 393.04370.

##### 3-Chloro-*N*-(2-((2-fluoro-5-(trifluoromethyl)benzyl)thio)ethyl)-5-(trifluoromethyl)picolinamide (E2)

Yield 83%; yellow solid; mp 101–103 °C. ^1^H NMR (400 MHz, CDCl_3_) *δ* 8.73 (s, 1H, pyridine-H), 8.08 (d, *J* = 1.3 Hz, 2H, pyridine-H, CO–NH), 7.69 (dd, *J* = 6.7, 2.0 Hz, 1H, Ar–H), 7.54–7.49 (m, 1H, Ar–H), 7.16 (t, *J* = 8.9 Hz, 1H, Ar–H), 3.85 (s, 2H, –CH_2_), 3.69 (q, *J* = 6.4 Hz, 2H, –CH_2_), 2.77 (t, *J* = 6.5 Hz, 2H, –CH_2_). ^13^C NMR (100 MHz, CDCl_3_) *δ* 162.5 (dd, *J* = 252.5, 1.3 Hz), 162.3, 148.9 (d, *J* = 1.0 Hz), 142.9 (q, *J* = 3.8 Hz), 137.7 (q, *J* = 3.6 Hz), 132.2, 129.3 (dd, *J* = 102.0 Hz, *J* = 34.0 Hz), 128.6–128.0 (m), 127.0 (dd, *J* = 33.1, 3.6 Hz), 126.7 (d, *J* = 16.0 Hz), 126.4 (dt, *J* = 13.0, 3.7 Hz), 123.6 (q, *J* = 272.0 Hz), 122.2 (q, *J* = 273.4 Hz), 116.2 (d, *J* = 23.4 Hz), 38.3, 31.5, 28.5 (d, *J* = 2.7 Hz). ^19^F NMR (376 MHz, CDCl_3_) *δ* −61.97, −62.57, *δ* −112.57 (dd, *J* = 14.2, 7.8 Hz). HRMS: [M + H]^+^ calcd for C_17_H_13_ClF_7_N_2_OS: 461.03199; found: 461.03098.

##### *N*-(2-((4-Bromo-2-fluorobenzyl)thio)ethyl)-3-chloro-5-(trifluoromethyl)picolinamide (E3)

Yield 77%; brown solid; mp 95–96 °C. ^1^H NMR (400 MHz, CDCl_3_) *δ* 8.73 (d, *J* = 1.0 Hz, 1H, pyridine-H), 8.08 (d, *J* = 1.3 Hz, 1H, –CO–NH), 8.05 (s, 1H, pyridine-H), 7.30–7.12 (m, 3H, Ar–H), 3.75 (s, 2H, –CH_2_), 3.67 (q, *J* = 6.4 Hz, 2H, –CH_2_), 2.73 (t, *J* = 6.5 Hz, 2H, –CH_2_). ^13^C NMR (100 MHz, CDCl_3_) *δ* 162.3, 160.6 (d, *J* = 251.4 Hz), 149.0, 142.9 (q, *J* = 3.7 Hz), 137.7 (q, *J* = 3.6 Hz), 132.2, 132.0 (d, *J* = 4.5 Hz), 129.3 (q, *J* = 34.0 Hz), 127.7 (d, *J* = 3.7 Hz), 124.7 (d, *J* = 14.9 Hz), 122.2 (d, *J* = 273.4 Hz), 121.2 (d, *J* = 9.5 Hz), 119.2 (d, *J* = 25.1 Hz), 38.4, 31.2, 28.4 (d, *J* = 2.6 Hz). ^19^F NMR (376 MHz, CDCl_3_) *δ* −62.52, −115.12. HRMS: [M + H]^+^ calcd for C_16_H_13_BrClF_4_N_2_OS: 470.95511; found: 470.95456.

##### *N*-(2-((2-Bromo-5-fluorobenzyl)thio)ethyl)-3-chloro-5-(trifluoromethyl)picolinamide (E4)

Yield 87%; gray solid; mp 111–112 °C. ^1^H NMR (400 MHz, CDCl_3_) *δ* 8.77–8.68 (m, 1H, pyridine-H), 8.14–8.01 (m, 2H, pyridine-H, –CO–NH), 7.50 (dd, *J* = 8.8, 5.3 Hz, 1H, Ar–H), 7.20 (dd, *J* = 9.1, 3.0 Hz, 1H, Ar–H), 6.85 (td, *J* = 8.3, 3.0 Hz, 1H, Ar–H), 3.87 (s, 2H, –CH_2_), 3.69 (q, *J* = 6.4 Hz, 2H, –CH_2_), 2.78 (t, *J* = 6.5 Hz, 2H, –CH_2_). ^13^C NMR (100 MHz, CDCl_3_) *δ* 162.3, 161.9 (d, *J* = 247.6 Hz), 149.0, 142.9 (q, *J* = 3.8 Hz), 139.6 (d, *J* = 7.3 Hz), 137.7 (q, *J* = 3.6 Hz), 134.2 (d, *J* = 8.0 Hz), 132.2, 129.3 (q, *J* = 34.0 Hz), 122.2 (d, *J* = 273.4 Hz), 118.5 (d, *J* = 3.3 Hz), 117.8 (d, *J* = 23.3 Hz), 116.1 (d, *J* = 22.4 Hz), 38.5, 36.1, 31.4. ^19^F NMR (376 MHz, CDCl_3_) *δ* −62.52, −114.19. HRMS: [M + H]^+^ calcd for C_16_H_13_BrClF_4_N_2_OS: 470.95511; found: 470.95468.

##### 3-Chloro-*N*-(2-((3-fluorobenzyl)thio)ethyl)-5-(trifluoromethyl)picolinamide (E5)

Yield 66%; yellow solid; mp 103–104 °C. ^1^H NMR (400 MHz, CDCl_3_) *δ* 8.73 (d, *J* = 1.1 Hz, 1H, pyridine-H), 8.08 (d, *J* = 1.3 Hz, 1H, pyridine-H), 8.02 (s, 1H, –CO–NH), 7.33–7.19 (m, 1H, Ar–H), 7.13–7.06 (m, 2H, Ar–H), 6.92 (td, *J* = 8.3, 2.0 Hz, 1H, Ar–H), 3.76 (s, 2H, –CH_2_), 3.63 (q, *J* = 6.3 Hz, 2H, –CH_2_), 2.71 (t, *J* = 6.5 Hz, 2H, –CH_2_). ^13^C NMR (100 MHz, CDCl_3_) *δ* 162.9 (d, *J* = 246.4 Hz), 162.3, 149.0, 142.9 (q, *J* = 3.8 Hz), 140.6 (d, *J* = 7.2 Hz), 137.7 (q, *J* = 3.5 Hz), 132.2, 130.1 (d, *J* = 8.3 Hz), 129.3 (q, *J* = 33.9 Hz), 124.6 (d, *J* = 2.8 Hz), 122.2 (d, *J* = 273.4 Hz), 115.8 (d, *J* = 21.7 Hz), 114.2 (d, *J* = 21.1 Hz), 38.3, 35.5 (d, *J* = 1.8 Hz), 31.0. ^19^F NMR (376 MHz, CDCl_3_) *δ* −62.52, −112.81. HRMS: [M − H]^−^ calcd for C_16_H_12_ClF_4_N_2_OS: 391.02895; found: 391.03027.

##### 3-Chloro-*N*-(2-((2,3-dichlorobenzyl)thio)ethyl)-5-(trifluoromethyl)picolinamide (E6)

Yield 80%; white solid; mp 130–132 °C. ^1^H NMR (400 MHz, CDCl_3_) *δ* 8.73 (dd, *J* = 1.8, 0.7 Hz, 1H, pyridine-H), 8.12–8.01 (m, 2H, pyridine-H, –CO–NH), 7.35 (dd, *J* = 8.0, 1.6 Hz, 1H, Ar–H), 7.31 (dd, *J* = 7.7, 1.6 Hz, 1H, Ar–H), 7.16 (t, *J* = 7.8 Hz, 1H, Ar–H), 3.92 (s, 2H, –CH_2_), 3.68 (q, *J* = 6.4 Hz, 2H, –CH_2_), 2.77 (t, *J* = 6.5 Hz, 2H, –CH_2_). ^13^C NMR (100 MHz, CDCl_3_) *δ* 162.3, 149.0, 142.9 (q, *J* = 3.8 Hz), 138.2, 137.7 (q, *J* = 3.6 Hz), 133.6, 132.4, 132.2, 129.4, 129.3 (q, *J* = 34.0 Hz), 128.9, 127.2, 122.2 (d, *J* = 273.5 Hz), 38.6, 34.5, 31.4. ^19^F NMR (376 MHz, CDCl_3_) *δ* −62.51. HRMS: [M + H]^+^ calcd for C_16_H_13_Cl_3_F_3_N_2_OS: 442.97608; found: 442.97546.

##### 3-Chloro-*N*-(2-((3-chloro-2-fluorobenzyl)thio)ethyl)-5-(trifluoromethyl)picolinamide (E7)

Yield 81%; gray solid; mp 97–98 °C. ^1^H NMR (400 MHz, CDCl_3_) *δ* 8.73 (d, *J* = 1.1 Hz, 1H, pyridine-H), 8.07 (t, *J* = 3.1 Hz, 2H, pyridine-H, –CO–NH), 7.35–7.16 (m, 2H, Ar–H), 7.04 (td, *J* = 7.9, 1.1 Hz, 1H, Ar–H), 3.81 (d, *J* = 0.8 Hz, 2H, –CH_2_), 3.68 (q, *J* = 6.4 Hz, 2H, –CH_2_), 2.76 (t, *J* = 6.5 Hz, 2H, –CH_2_). ^13^C NMR (100 MHz, CDCl_3_) *δ* 162.3, 157.5, 149.0, 142.9 (q, *J* = 3.9 Hz), 137.7 (q, *J* = 3.6 Hz), 132.2, 129.6, 129.3 (q, *J* = 34.0 Hz), 129.2 (d, *J* = 3.3 Hz), 127.3 (d, *J* = 14.8 Hz), 124.6 (d, *J* = 4.8 Hz), 122.2 (q, *J* = 273.3 Hz), 121.3 (d, *J* = 18.0 Hz), 38.4, 31.3, 29.0 (d, *J* = 2.8 Hz). ^19^F NMR (376 MHz, CDCl_3_) *δ* −62.52, −119.81. HRMS: [M − H]^−^ calcd for C_16_H_11_Cl_2_F_4_N_2_OS: 424.98998; found: 424.99158.

##### 3-Chloro-*N*-(2-((3,4-difluorobenzyl)thio)ethyl)-5-(trifluoromethyl)picolinamide (E8)

Yield 68%; white solid; mp 97–98 °C. ^1^H NMR (400 MHz, CDCl_3_) *δ* 8.73 (s, 1H, pyridine-H), 8.09 (s, 1H, pyridine-H), 8.02 (s, 1H, –CO–NH), 7.29–7.17 (m, 1H, Ar–H), 7.14–7.01 (m, 2H, Ar–H), 3.73 (s, 2H, –CH_2_), 3.67–3.58 (m, 2H, –CH_2_), 2.70 (td, *J* = 6.5, 1.6 Hz, 2H, –CH_2_). ^13^C NMR (100 MHz, CDCl_3_) *δ* 162.3, 150.3 (dd, *J* = 248.8, 12.9 Hz), 149.5 (dd, *J* = 247.9, 12.7 Hz), 148.9, 142.9 (q, *J* = 3.7 Hz), 137.8 (q, *J* = 3.6 Hz), 135.1 (dd, *J* = 5.2, 4.1 Hz), 132.2, 129.3 (q, *J* = 33.9 Hz), 124.8 (dd, *J* = 6.2, 3.6 Hz), 122.2 (q, *J* = 273.5 Hz), 117.7 (d, *J* = 17.4 Hz), 117.2 (d, *J* = 17.2 Hz), 38.3, 35.1, 30.9. ^19^F NMR (376 MHz, CDCl_3_) *δ* −62.54, *δ* −137.19 (d, *J* = 21.2 Hz), −139.64 (d, *J* = 21.2 Hz). HRMS: [M − H]^−^ calcd for C_16_H_11_ClF_5_N_2_OS: 409.01953; found: 409.02078.

##### *N*-(2-(Benzylthio)ethyl)-3-chloro-5-(trifluoromethyl)picolinamide (E9)

Yield 75%; brown solid; mp 109–110 °C. ^1^H NMR (400 MHz, CDCl_3_) *δ* 8.73 (d, *J* = 1.0 Hz, 1H, pyridine-H), 8.07 (d, *J* = 1.3 Hz, 1H, pyridine-H), 8.01 (s, 1H, –CO–NH), 7.36–7.27 (m, 4H, Ar–H), 7.26–7.20 (m, 1H, Ar–H), 3.78 (s, 2H, –CH_2_), 3.62 (q, *J* = 6.3 Hz, 2H, –CH_2_), 2.70 (t, *J* = 6.5 Hz, 2H, –CH_2_). ^13^C NMR (100 MHz, CDCl_3_) *δ* 162.3, 149.2, 142.9 (q, *J* = 3.8 Hz), 138.0, 137.7 (q, *J* = 3.6 Hz), 132.2, 129.2 (q, *J* = 34.1 Hz), 128.9, 128.6, 127.2, 122.2 (q, *J* = 273.5 Hz), 38.3, 35.9, 30.9. ^19^F NMR (376 MHz, CDCl_3_) *δ* −62.51. HRMS: [M − H]^−^ calcd for C_16_H_13_ClF_3_N_2_OS: 373.03955; found: 373.03837.

##### 3-Chloro-*N*-(2-(((3,4-dimethoxypyridin-2-yl)methyl)thio)ethyl)-5-(trifluoromethyl)picolinamide (E10)

Yield 60%; gray solid; mp 76–77 °C. ^1^H NMR (400 MHz, CDCl_3_) *δ* 8.72 (d, *J* = 1.0 Hz, 1H, pyridine-H), 8.45 (s, 1H, pyridine-H), 8.14 (d, *J* = 5.5 Hz, 1H, pyridine-H), 8.06 (d, *J* = 1.3 Hz, 1H, –CO–NH), 6.76 (d, *J* = 5.6 Hz, 1H, pyridine-H), 3.97–3.86 (m, 8H, –(OCH_3_)_2_, –CH_2_), 3.73 (dd, *J* = 12.3, 6.0 Hz, 2H, –CH_2_), 2.86 (t, *J* = 6.2 Hz, 2H, –CH_2_). ^13^C NMR (100 MHz, CDCl_3_) *δ* 162.5, 158.8, 152.7, 150.0, 145.4, 143.4, 142.9 (q, *J* = 3.8 Hz), 137.3 (q, *J* = 3.6 Hz), 131.9, 129.0 (q, *J* = 33.9 Hz), 122.2 (q, *J* = 273.4 Hz), 106.9, 61.1, 55.7, 39.1, 31.69, 31.4. ^19^F NMR (376 MHz, CDCl_3_) *δ* −62.50. HRMS: [M + H]^+^ calcd for C_17_H_18_ClF_3_N_3_O_3_S: 436.07040; found: 436.06998.

##### 3-Chloro-5-(trifluoromethyl)-*N*-(2-((4-(trifluoromethyl)benzyl)thio)ethyl)picolinamide (E11)

Yield 88%; yellow solid; mp 114–115 °C. ^1^H NMR (400 MHz, CDCl_3_) *δ* 8.75–8.70 (m, 1H, pyridine-H), 8.12–8.06 (m, 1H, pyridine-H), 8.03 (s, 1H, –CO–NH), 7.57 (d, *J* = 8.1 Hz, 2H, Ar–H), 7.47 (d, *J* = 8.1 Hz, 2H), 3.82 (s, 2H), 3.65 (q, *J* = 6.5 Hz, 2H), 2.69 (t, *J* = 6.6 Hz, 2H). ^13^C NMR (100 MHz, CDCl_3_) *δ* 162.3, 148.9, 142.9 (q, *J* = 3.9 Hz), 142.2, 137.8 (q, *J* = 3.6 Hz), 132.2, 129.4 (q, *J* = 32.6 Hz), 129.3 (q, *J* = 34.0 Hz), 129.2, 125.6 (q, *J* = 3.8 Hz), 124.1 (q, *J* = 272.0 Hz), 122.1 (q, *J* = 273.4 Hz), 38.3, 35.4, 30.9. ^19^F NMR (376 MHz, CDCl_3_) *δ* −62.48, −62.54. HRMS: [M + H]^+^ calcd for C_17_H_14_ClF_6_N_2_OS: 443.04041; found: 443.04141.

##### 3-Chloro-*N*-(2-((2-chloro-4-fluorobenzyl)thio)ethyl)-5-(trifluoromethyl)picolinamide (E12)

Yield 68%; gray solid; mp 90–91 °C. ^1^H NMR (400 MHz, CDCl_3_) *δ* 8.73 (s, 1H), 8.08 (d, *J* = 0.4 Hz, 2H), 7.38 (dd, *J* = 8.5, 6.1 Hz, 1H), 7.18–7.06 (m, 1H), 6.95 (ddd, *J* = 8.2, 2.5, 1.2 Hz, 1H), 3.86 (s, 2H), 3.68 (q, *J* = 6.4 Hz, 2H), 2.75 (t, *J* = 6.5 Hz, 2H). ^13^C NMR (100 MHz, CDCl_3_) *δ* 162.3, 161.6 (d, *J* = 249.7 Hz), 149.0, 142.9 (q, *J* = 3.8 Hz), 137.7 (q, *J* = 3.5 Hz), 134.6 (d, *J* = 10.3 Hz), 132.2, 131.8, 131.7, 129.3 (q, *J* = 34.0 Hz), 122.2 (q, *J* = 273.4 Hz), 117.2 (d, *J* = 24.7 Hz), 114.2 (d, *J* = 21.1 Hz), 38.6, 32.8, 31.2. ^19^F NMR (376 MHz, CDCl_3_) *δ* −62.52, −112.69. HRMS: [M + H]^+^ calcd for C_16_H_13_Cl_2_F_4_N_2_OS: 427.00563; found: 427.00470.

##### 3-Chloro-*N*-(2-((4-isopropylbenzyl)thio)ethyl)-5-(trifluoromethyl)picolinamide (E13)

Yield 79%; yellow solid; mp 98–100 °C. ^1^H NMR (400 MHz, CDCl_3_) *δ* 8.73 (d, *J* = 1.1 Hz, 1H), 8.11–8.06 (m, 1H), 8.03 (s, 1H), 7.28–7.23 (m, 2H), 7.17 (d, *J* = 8.1 Hz, 2H), 3.75 (s, 2H), 3.64 (dd, *J* = 12.7, 6.3 Hz, 2H), 2.88 (dt, *J* = 13.9, 6.9 Hz, 1H), 2.70 (t, *J* = 6.5 Hz, 2H), 1.23 (d, *J* = 6.9 Hz, 6H). ^13^C NMR (100 MHz, CDCl_3_) *δ* 162.2, 149.2, 147.9, 142.8 (q, *J* = 3.8 Hz), 137.6 (q, *J* = 3.5 Hz), 135.1, 132.1, 129.2 (d, *J* = 34.0 Hz), 128.8, 126.7, 122.2 (q, *J* = 273.5 Hz), 38.3, 35.5, 33.8, 30.8, 24.0. ^19^F NMR (376 MHz, CDCl_3_) *δ* −62.52. HRMS: [M − H]^−^ calcd for C_19_H_19_ClF_3_N_2_OS: 415.08532; found: 415.08630.

##### *N*-(2-((2-Bromo-4-fluorobenzyl)thio)ethyl)-3-chloro-5-(trifluoromethyl)picolinamide (E14)

Yield 64%; yellow solid; mp 92–94 °C. ^1^H NMR (400 MHz, CDCl_3_) *δ* 8.73 (d, *J* = 1.1 Hz, 1H), 8.08 (d, *J* = 1.3 Hz, 1H), 8.05 (s, 1H), 7.39 (dd, *J* = 8.5, 5.9 Hz, 1H), 7.29 (dd, *J* = 8.2, 2.6 Hz, 1H), 6.99 (td, *J* = 8.3, 2.6 Hz, 1H), 3.87 (s, 2H), 3.68 (q, *J* = 6.4 Hz, 2H), 2.76 (t, *J* = 6.5 Hz, 2H). ^13^C NMR (100 MHz, CDCl_3_) *δ* 162.3, 161.4 (d, *J* = 250.8 Hz), 149.1, 142.8 (q, *J* = 3.8 Hz), 137.7 (q, *J* = 3.6 Hz), 133.5 (d, *J* = 3.6 Hz), 132.2, 131.7 (d, *J* = 8.4 Hz), 129.3 (q, *J* = 34.0 Hz), 124.4 (d, *J* = 9.6 Hz), 122.2 (q, *J* = 273.4 Hz), 120.3 (d, *J* = 24.5 Hz), 114.8 (d, *J* = 21.1 Hz), 38.6, 35.5, 31.3. ^19^F NMR (376 MHz, CDCl_3_) *δ* −62.55, −112.79. HRMS: [M + H]^+^ calcd for C_16_H_13_BrClF_4_N_2_OS: 470.95511; found: 470.95468.

##### *N*-(2-((3-Bromobenzyl)thio)ethyl)-3-chloro-5-(trifluoromethyl)picolinamide (E15)

Yield 82%; gray solid; mp 89–91 °C. ^1^H NMR (400 MHz, CDCl_3_) *δ* 8.74 (d, *J* = 1.0 Hz, 1H), 8.07 (d, *J* = 1.4 Hz, 1H), 8.01 (s, 1H), 7.51 (t, *J* = 1.6 Hz, 1H), 7.36 (d, *J* = 7.9 Hz, 1H), 7.26 (t, *J* = 3.8 Hz, 1H), 7.17 (t, *J* = 7.8 Hz, 1H), 3.73 (s, 2H), 3.63 (q, *J* = 6.3 Hz, 2H), 2.71 (t, *J* = 6.5 Hz, 2H). ^13^C NMR (100 MHz, CDCl_3_) *δ* 162.3, 149.1, 142.9 (q, *J* = 3.8 Hz), 140.4, 137.7 (q, *J* = 3.5 Hz), 132.2, 131.9, 130.3, 130.1, 129.3 (q, *J* = 33.9 Hz), 127.5, 122.7, 122.2 (q, *J* = 273.4 Hz), 38.4, 35.5, 31.1. ^19^F NMR (376 MHz, CDCl_3_) *δ* −62.54. HRMS: [M + H]^+^ calcd for C_16_H_14_BrClF_3_N_2_OS: 452.96454; found: 452.96402.

##### 3-Chloro-*N*-(2-((2,6-difluorobenzyl)thio)ethyl)-5-(trifluoromethyl)picolinamide (E16)

Yield 72%; white solid; mp 112–113 °C. ^1^H NMR (400 MHz, CDCl_3_) *δ* 8.76–8.70 (m, 1H), 8.07 (dd, *J* = 1.2, 0.6 Hz, 2H), 7.25–7.16 (m, 1H), 6.93–6.85 (m, 2H), 3.82 (s, 2H), 3.71 (q, *J* = 6.2 Hz, 2H), 2.80 (t, *J* = 6.4 Hz, 2H). ^13^C NMR (100 MHz, CDCl_3_) *δ* 161.1 (dd, *J* = 248.7, 7.9 Hz), 162.2, 149.1, 142.9 (q, *J* = 3.8 Hz), 137.7 (q, *J* = 3.4 Hz), 132.2, 129.2 (q, *J* = 34.0 Hz), 128.8 (t, *J* = 10.3 Hz), 122.2 (q, *J* = 273.4 Hz), 115.0 (t, *J* = 19.2 Hz), 111.4 (q, *J* = 12.6 Hz), 38.3, 31.6, 22.3. ^19^F NMR (376 MHz, CDCl_3_) *δ* −62.52, −115.00. HRMS: [M + H]^+^ calcd for C_16_H_13_ClF_5_N_2_OS: 411.03518; found: 411.03448.

##### 3-Chloro-*N*-(2-((2-methylbenzyl)thio)ethyl)-5-(trifluoromethyl)picolinamide (E17)

Yield 82%; gray solid; mp 84–85 °C. ^1^H NMR (400 MHz, CDCl_3_) *δ* 8.72 (d, *J* = 1.0 Hz, 1H), 8.07 (d, *J* = 1.3 Hz, 1H), 8.02 (s, 1H), 7.25–7.19 (m, 1H), 7.16–7.11 (m, 3H), 3.78 (s, 2H), 3.65 (q, *J* = 6.3 Hz, 2H), 2.74 (t, *J* = 6.5 Hz, 2H), 2.41 (s, 3H). ^13^C NMR (100 MHz, CDCl_3_) *δ* 162.2, 149.1, 142.9 (q, *J* = 3.9 Hz), 137.7 (q, *J* = 3.6 Hz), 136.7, 135.6, 132.2, 130.9, 129.7, 129.2 (d, *J* = 33.9 Hz), 127.5, 126.0, 122.2 (d, *J* = 273.3 Hz), 38.6, 34.2, 31.3, 19.2. ^19^F NMR (376 MHz, CDCl_3_) *δ* −62.50. HRMS: [M + H]^+^ calcd for C_17_H_17_ClF_3_N_2_OS: 389.06967; found: 389.06924.

##### 3-Chloro-5-(trifluoromethyl)-*N*-(2-((2-(trifluoromethyl)benzyl)thio)ethyl)picolinamide (E18)

Yield 60%; yellow solid; mp 77–78 °C. ^1^H NMR (400 MHz, CDCl_3_) *δ* 8.73 (d, *J* = 0.8 Hz, 1H), 8.08 (dd, *J* = 1.3, 0.5 Hz, 2H), 7.64 (t, *J* = 8.4 Hz, 2H), 7.52 (t, *J* = 7.5 Hz, 1H), 7.35 (t, *J* = 7.6 Hz, 1H), 3.96 (s, 2H), 3.66 (q, *J* = 6.4 Hz, 2H), 2.79 (t, *J* = 6.5 Hz, 2H). ^13^C NMR (100 MHz, CDCl_3_) *δ* 162.3, 149.0, 142.9 (q, *J* = 3.8 Hz), 137.7 (q, *J* = 3.6 Hz), 136.8 (d, *J* = 1.4 Hz), 132.2, 132.1, 131.5, 129.3 (q, *J* = 33.9 Hz), 128.5 (q, *J* = 29.9 Hz), 127.3, 126.2 (q, *J* = 5.6 Hz), 124.3 (d, *J* = 274.0 Hz), 122.2 (d, *J* = 273.5 Hz), 38.5, 32.5 (d, *J* = 2.0 Hz), 31.9. ^19^F NMR (376 MHz, CDCl_3_) *δ* −59.05, −62.53. HRMS: [M − H]^−^ calcd for C_17_H_12_ClF_6_N_2_OS: 441.02704; found: 441.02576.

##### 3-Chloro-*N*-(2-((2-chlorobenzyl)thio)ethyl)-5-(trifluoromethyl)picolinamide (E19)

Yield 43%; brown solid; mp 77–78 °C. ^1^H NMR (400 MHz, CDCl_3_) *δ* 8.73 (d, *J* = 1.1 Hz, 1H), 8.07 (d, *J* = 1.3 Hz, 2H), 7.39 (dd, *J* = 7.3, 1.9 Hz, 1H), 7.36 (dd, *J* = 7.6, 1.6 Hz, 1H), 7.25–7.16 (m, 2H), 3.90 (s, 2H), 3.68 (q, *J* = 6.3 Hz, 2H), 2.76 (t, *J* = 6.5 Hz, 2H). ^13^C NMR (100 MHz, CDCl_3_) *δ* 162.3, 149.1, 142.9 (q, *J* = 3.8 Hz), 137.7 (q, *J* = 3.6 Hz), 135.8, 134.0, 132.2, 130.9, 129.9, 129.2 (q, *J* = 34.1 Hz), 128.7, 127.0, 122.2 (q, *J* = 273.5 Hz), 38.6, 33.5, 31.3. ^19^F NMR (376 MHz, CDCl_3_) *δ* −62.51. HRMS: [M + H]^+^ calcd for C_16_H_14_Cl_2_F_3_N_2_OS: 409.01505; found: 409.01443.

##### *N*-(2-((4-(*tert*-Butyl)benzyl)thio)ethyl)-3-chloro-5-(trifluoromethyl)picolinamide (E20)

Yield 84%; gray solid; mp 111–113 °C. ^1^H NMR (400 MHz, CDCl_3_) *δ* 8.74 (d, *J* = 1.1 Hz, 1H), 8.08 (d, *J* = 1.3 Hz, 1H), 8.04 (s, 1H), 7.37–7.31 (m, 2H), 7.30–7.18 (m, 2H), 3.75 (s, 2H), 3.64 (q, *J* = 6.3 Hz, 2H), 2.70 (t, *J* = 6.5 Hz, 2H), 1.30 (s, 9H). ^13^C NMR (100 MHz, CDCl_3_) *δ* 162.3, 150.2, 149.2, 142.9 (q, *J* = 3.8 Hz), 137.7 (q, *J* = 3.5 Hz), 134.7, 132.2, 129.2 (q, *J* = 33.9 Hz), 128.6, 125.6, 122.2 (d, *J* = 273.4 Hz), 38.3, 35.4, 34.5, 31.3, 30.9. ^19^F NMR (376 MHz, CDCl_3_) *δ* −62.51. HRMS: [M + H]^+^ calcd for C_20_H_23_ClF_3_N_2_OS: 431.11662; found: 431.11563.

##### 3-Chloro-*N*-(2-(((6-chloropyridin-3-yl)methyl)thio)ethyl)-5-(trifluoromethyl)picolinamide (E21)

Yield 48%; brown solid; mp 88–89 °C. ^1^H NMR (400 MHz, CDCl_3_) *δ* 8.73 (s, 1H), 8.34 (d, *J* = 2.2 Hz, 1H), 8.09 (s, 1H), 8.05 (s, 1H), 7.70 (dd, *J* = 8.2, 2.4 Hz, 1H), 7.29 (d, *J* = 8.2 Hz, 1H), 3.76 (s, 2H), 3.66 (q, *J* = 6.5 Hz, 2H), 2.70 (t, *J* = 6.6 Hz, 2H). ^13^C NMR (100 MHz, CDCl_3_) *δ* 162.4, 150.3, 149.7, 148.8, 142.9 (q, *J* = 3.8 Hz), 139.3, 137.8 (q, *J* = 3.6 Hz), 132.9, 132.3, 129.4 (q, *J* = 34.0 Hz), 124.3, 122.1 (q, *J* = 273.4 Hz), 38.4, 32.3, 31.0. ^19^F NMR (376 MHz, CDCl_3_) *δ* −62.52. HRMS: [M − H]^−^ calcd for C_15_H_11_Cl_2_F_3_N_3_OS: 407.99465; found: 407.99591.

##### 3-Chloro-*N*-(2-((4-cyanobenzyl)thio)ethyl)-5-(trifluoromethyl)picolinamide (E22)

Yield 83%; gray solid; mp 87–89 °C. ^1^H NMR (400 MHz, CDCl_3_) *δ* 8.73 (d, *J* = 1.0 Hz, 1H), 8.12–8.07 (m, 1H), 8.03 (s, 1H), 7.61 (d, *J* = 8.1 Hz, 2H), 7.47 (d, *J* = 8.2 Hz, 2H), 3.81 (s, 2H), 3.64 (q, *J* = 6.5 Hz, 2H), 2.68 (t, *J* = 6.7 Hz, 2H). ^13^C NMR (100 MHz, CDCl_3_) *δ* 162.3, 148.9, 143.7, 142.9 (q, *J* = 3.8 Hz), 137.8 (q, *J* = 3.6 Hz), 132.4, 132.3, 129.7, 129.4 (q, *J* = 33.3 Hz), 122.1 (d, *J* = 273.4 Hz), 118.7, 111.1, 38.4, 35.6, 31.0. ^19^F NMR (376 MHz, CDCl_3_) *δ* −62.51. HRMS: [M − H]^−^ calcd for C_17_H_12_ClF_3_N_3_OS: 398.03362; found: 398.03470.

##### 3-Chloro-*N*-(2-((3,5-difluorobenzyl)thio)ethyl)-5-(trifluoromethyl)picolinamide (E23)

Yield 83%; white solid; mp 94–95 °C. ^1^H NMR (400 MHz, CDCl_3_) *δ* 8.74 (d, *J* = 1.1 Hz, 1H), 8.13–8.07 (m, 1H), 8.03 (s, 1H), 6.99–6.82 (m, 2H), 6.68 (tt, *J* = 8.9, 2.3 Hz, 1H), 3.74 (s, 2H), 3.64 (q, *J* = 6.4 Hz, 2H), 2.72 (t, *J* = 6.5 Hz, 2H). ^13^C NMR (100 MHz, CDCl_3_) *δ* 163.0 (dd, *J* = 249.0, 12.8 Hz), 162.3, 148.9, 142.9 (q, *J* = 3.8 Hz), 142.1 (t, *J* = 9.0 Hz), 137.8 (q, *J* = 3.6 Hz), 132.3, 129.3 (q, *J* = 33.9 Hz), 122.2 (q, *J* = 273.5 Hz), 111.8 (q, *J* = 11.7 Hz), 102.8 (t, *J* = 25.3 Hz), 38.3, 35.5, 31.1. ^19^F NMR (376 MHz, CDCl_3_) *δ* −62.53, −109.51. HRMS: [M − H]^−^ calcd for C_16_H_11_ClF_5_N_2_OS: 409.01953; found: 409.02075.

##### 3-Chloro-*N*-(2-(((3-methyl-4-(trifluoromethoxy)pyridin-2-yl)methyl)thio)ethyl)-5-(trifluoromethyl)picolinamide (E24)

Yield 77%; gray solid; mp 124–125 °C. ^1^H NMR (400 MHz, CDCl_3_) *δ* 8.72 (s, 1H), 8.40 (s, 1H), 8.27 (d, *J* = 5.7 Hz, 1H), 8.06 (d, *J* = 1.1 Hz, 1H), 6.63 (d, *J* = 5.7 Hz, 1H), 4.39 (q, *J* = 7.9 Hz, 2H), 3.93 (s, 2H), 3.70 (dd, *J* = 12.4, 6.0 Hz, 2H), 2.82 (t, *J* = 6.3 Hz, 2H), 2.29 (s, 3H). ^13^C NMR (100 MHz, CDCl_3_) *δ* 162.5, 161.8, 158.1, 149.8, 147.5, 142.9 (q, *J* = 3.8 Hz), 137.4 (q, *J* = 3.7 Hz), 131.9, 129.0 (q, *J* = 33.8 Hz), 123.0 (q, *J* = 277.8 Hz), 122.2 (q, *J* = 273.3 Hz), 121.1, 105.4, 65.4 (q, *J* = 36.3 Hz), 39.2, 35.4, 31.1, 10.6. ^19^F NMR (376 MHz, CDCl_3_) *δ* −62.53, −73.85. HRMS: [M + H]^+^ calcd for C_18_H_17_ClF_6_N_3_O_2_S: 488.06287; found: 488.06143.

##### 3-Chloro-*N*-(2-((3,4,4-trifluorobut-3-en-1-yl)thio)ethyl)-5-(trifluoromethyl)picolinamide (E25)

Yield 84%; brown solid; mp 69–70 °C. ^1^H NMR (400 MHz, CDCl_3_) *δ* 8.74 (d, *J* = 1.0 Hz, 1H), 8.11 (s, 1H), 8.08 (d, *J* = 1.4 Hz, 1H), 3.68 (q, *J* = 6.5 Hz, 2H), 2.83 (t, *J* = 5.4 Hz, 2H), 2.79 (t, *J* = 6.0 Hz, 2H), 2.60 (dddd, *J* = 11.1, 7.1, 5.3, 3.2 Hz, 2H). ^13^C NMR (100 MHz, CDCl_3_) *δ* 162.3, 153.6 (ddd, *J* = 287.1, 273.3, 46.3 Hz), 148.9, 142.9 (q, *J* = 3.8 Hz), 137.7 (q, *J* = 3.6 Hz), 132.2, 129.3 (q, *J* = 34.0 Hz), 127.2 (ddd, *J* = 69.7, 56.0, 16.2 Hz), 122.2 (q, *J* = 273.4 Hz), 38.7, 31.6, 27.6, 26.4 (dd, *J* = 21.9, 2.6 Hz). ^19^F NMR (376 MHz, CDCl_3_) *δ* −62.55, −103.74 (dd, *J* = 85.2, 32.5 Hz), −123.06 (dd, *J* = 114.4, 85.2 Hz), −175.59 (dd, *J* = 114.2, 32.4 Hz). HRMS: [M + H]^+^ calcd for C_12_H_8_ClF_4_N_2_OS: 393.04372; found: 393.04463.

##### 3-Chloro-*N*-(2-((2,5-difluorobenzyl)thio)ethyl)-5-(trifluoromethyl)picolinamide (E26)

Yield 62%; white solid; mp 111–112 °C. ^1^H NMR (400 MHz, CDCl_3_) *δ* 8.74 (d, *J* = 1.1 Hz, 1H), 8.08 (d, *J* = 1.3 Hz, 1H), 7.12 (ddd, *J* = 8.8, 5.8, 3.1 Hz, 1H), 7.00 (td, *J* = 9.0, 4.5 Hz, 1H), 6.94–6.86 (m, 1H), 3.77 (s, 2H), 3.67 (q, *J* = 6.3 Hz, 2H), 2.76 (t, *J* = 6.5 Hz, 2H). ^13^C NMR (100 MHz, CDCl_3_) *δ* 162.3, 158.6 (dd, *J* = 242.8, 2.4 Hz), 156.8 (dd, *J* = 242.2, 2.5 Hz), 149.0, 142.9 (q, *J* = 3.8 Hz), 137.7 (q, *J* = 3.6 Hz), 132.2, 129.3 (q, *J* = 34.0 Hz), 127.1 (dd, *J* = 17.4, 7.6 Hz), 122.2 (q, *J* = 273.3 Hz), 117.2 (dd, *J* = 24.4, 4.1 Hz), 116.6 (dd, *J* = 24.9, 8.7 Hz), 115.4 (dd, *J* = 22.8, 7.3 Hz), 38.3, 31.3, 28.6. ^19^F NMR (376 MHz, CDCl_3_) *δ* −62.53, *δ* −118.44 (d, *J* = 17.8 Hz), −124.24 (d, *J* = 17.8 Hz). HRMS: [M − H]^−^ calcd for C_16_H_11_ClF_5_N_2_OS: 409.01953; found: 409.02078.

#### 4.2.3 The synthesis of sulfone-containing compounds F1–F10

Taking F1 as an example, to a mixture of 3-chloro-*N*-(2-((4-fluorobenzyl)thio)ethyl)-5-(trifluoromethyl)picolinamide (E1, 1.27 mmol) and 20 mL EtOH stirred at room temperature, H_2_O_2_ (53.46 mmol) and ammonium molybdate (0.089 mmol) were added. The resulting solution was stirred at 100 °C and monitored by TLC. After about 7–8 h, the reaction could be completed. The solvent was removed under reduced pressure to provide crude product, which was purified by silica gel (200–300 mesh) column chromatography with ethyl acetate/petroleum ether (1 : 3) to obtain pure sulfone-containing compound F1. The sulfone-containing compounds F2–F10 could be also synthesized with similar method. The data of F1–F10 are listed below, and the spectra are shown in ESI data.†

##### 3-Chloro-*N*-(2-((4-fluorobenzyl)sulfonyl)ethyl)-5-(trifluoromethyl)picolinamide (F1)

Yield 41%; white solid; mp 132–133 °C. ^1^H NMR (400 MHz, CDCl_3_) *δ* 8.72 (d, *J* = 1.1 Hz, 1H, pyridine-H), 8.40 (t, *J* = 5.3 Hz, 1H, –CO–NH–), 8.07 (d, *J* = 1.4 Hz, 1H, pyridine-H), 7.33–7.23 (m, 2H, Ar–H), 7.15–7.00 (m, 2H, Ar–H), 4.03 (q, *J* = 13.2 Hz, 2H, –CH_2_–), 3.96 (dd, *J* = 12.1, 6.1 Hz, 2H, –CH_2_–), 3.11–2.77 (m, 2H, –CH_2_–). ^13^C NMR (100 MHz, CDCl_3_) *δ* 162.9 (d, *J* = 248.2 Hz), 162.8, 148.7, 143.0 (q, *J* = 3.8 Hz), 137.6 (q, *J* = 3.6 Hz), 132.1, 131.9 (d, *J* = 8.3 Hz), 129.4 (q, *J* = 34.1 Hz), 125.2 (d, *J* = 3.3 Hz), 122.1 (q, *J* = 273.3 Hz), 116.1 (d, *J* = 21.7 Hz), 57.4, 49.5, 34.4. ^19^F NMR (376 MHz, CDCl_3_) *δ* −62.57, −112.80. HRMS: calculated for C_16_H_14_O_3_N_2_ClF_4_S [M + H]^+^: 435.03443; found: 425.03378.

##### 3-Chloro-*N*-(2-((2-fluoro-5-(trifluoromethyl)benzyl)sulfonyl)ethyl)-5-(trifluoromethyl)picolinamide (F2)

Yield 50%; white solid; mp 150–151 °C. ^1^H NMR (400 MHz, CDCl_3_) *δ* 8.73 (d, *J* = 0.9 Hz, 1H, pyridine-H), 8.37 (s, 1H, –CO–NH–), 8.07 (d, *J* = 1.4 Hz, 1H, pyridine-H), 7.71–7.60 (m, 2H, Ar–H), 7.26 (dd, *J* = 11.5, 6.1 Hz, 1H, Ar–H), 4.14 (dd, *J* = 38.7, 13.2 Hz, 2H, –CH_2_–), 3.99 (q, *J* = 6.1 Hz, 2H, –CH_2_–), 3.33–2.55 (m, 2H, –CH_2_–). ^13^C NMR (100 MHz, CDCl_3_) *δ* 162.8, 162.8 (d, *J* = 253.8 Hz), 148.6, 143.0 (q, *J* = 3.8 Hz), 137.7 (q, *J* = 3.6 Hz), 132.2, 129.9 (q, *J* = 8.0 Hz), 129.4 (q, *J* = 34.1 Hz), 128.0 (d, *J* = 9.4 Hz), 127.4 (q, *J* = 33.4 Hz), 123.4 (q, *J* = 272.2 Hz), 122.1 (d, *J* = 273.5 Hz), 118.3 (d, *J* = 16.4 Hz), 116.5 (d, *J* = 23.2 Hz), 50.8, 50.3, 34.3. ^19^F NMR (376 MHz, CDCl_3_) *δ* −62.03, −62.60, −110.62. HRMS: calculated for C_16_H_14_O_3_N_2_ClF_4_S [M + H]^+^: 477.02690; found: 477.02563.

##### *N*-(2-((4-Bromo-2-fluorobenzyl)sulfonyl)ethyl)-3-chloro-5-(trifluoromethyl)picolinamide (F3)

Yield 47%; yellow solid; mp 124–125 °C. ^1^H NMR (400 MHz, CDCl_3_) *δ* 8.72 (d, *J* = 1.0 Hz, 1H, pyridine-H), 8.38 (t, *J* = 5.2 Hz, 1H, –CO–NH–), 8.07 (d, *J* = 1.2 Hz, 1H, pyridine-H), 7.32 (d, *J* = 8.8 Hz, 2H, Ar–H), 7.25 (dd, *J* = 14.0, 6.0 Hz, 1H, Ar–H), 4.06 (dd, *J* = 36.8, 13.4 Hz, 2H, –CH_2_–), 3.96 (dt, *J* = 7.1, 3.4 Hz, 2H, –CH_2_–), 3.15–2.79 (m, 2H, –CH_2_–). ^13^C NMR (100 MHz, CDCl_3_) *δ* 162.8, 160.7 (d, *J* = 252.3 Hz), 148.7, 143.0 (q, *J* = 3.8 Hz), 137.6 (q, *J* = 3.5 Hz), 133.4 (d, *J* = 3.9 Hz), 132.1, 129.4 (q, *J* = 34.1 Hz), 128.1 (d, *J* = 3.7 Hz), 123.2, 122.1 (q, *J* = 273.5 Hz), 119.5 (d, *J* = 24.9 Hz), 116.0 (d, *J* = 15.3 Hz), 50.6, 50.0, 34.4. ^19^F NMR (376 MHz, CDCl_3_) *δ* −62.57, −113.50. HRMS: calculated for C_16_H_13_O_3_N_2_BrClF_4_S [M + H]^+^: 502.94494; found: 502.94443.

##### *N*-(2-((2-Bromo-5-fluorobenzyl)sulfonyl)ethyl)-3-chloro-5-(trifluoromethyl)picolinamide (F4)

Yield 44%; white solid; mp 146–147 °C. ^1^H NMR (400 MHz, CDCl_3_) *δ* 8.73 (d, *J* = 1.1 Hz, 1H, pyridine-H), 8.41 (t, *J* = 5.3 Hz, 1H, –CO–NH–), 8.07 (d, *J* = 1.3 Hz, 1H, pyridine-H), 7.58 (dd, *J* = 8.8, 5.2 Hz, 1H, Ar–H), 7.17 (dd, *J* = 8.7, 3.0 Hz, 1H, Ar–H), 6.97 (ddd, *J* = 8.8, 7.9, 3.0 Hz, 1H, Ar–H), 4.22 (dd, *J* = 48.0, 13.0 Hz, 2H, –CH_2_–), 4.05–3.95 (m, 2H, –CH_2_–), 3.25–2.90 (m, 2H, –CH_2_–). ^13^C NMR (100 MHz, CDCl_3_) *δ* 162.8, 161.7 (d, *J* = 249.1 Hz), 148.8, 143.1 (q, *J* = 3.8 Hz), 137.6 (q, *J* = 3.6 Hz) 134.5 (d, *J* = 8.1 Hz), 132.1, 131.8 (d, *J* = 8.0 Hz), 129.4 (q, *J* = 34.0 Hz), 122.1 (q, *J* = 273.4 Hz), 119.5 (d, *J* = 23.5 Hz), 119.2 (d, *J* = 3.5 Hz), 117.6 (d, *J* = 22.4 Hz), 58.3, 50.1, 34.5. ^19^F NMR (376 MHz, CDCl_3_) *δ* −62.57, −113.05. HRMS: calculated for C_16_H_13_O_3_N_2_BrClF_4_S [M + H]^+^: 502.94494; found: 502.94223.

##### 3-Chloro-*N*-(2-((3-fluorobenzyl)sulfonyl)ethyl)-5-(trifluoromethyl)picolinamide (F5)

Yield 50%; yellow solid; mp 97–98 °C. ^1^H NMR (400 MHz, CDCl_3_) *δ* 8.73 (d, *J* = 1.1 Hz, 1H, pyridine-H), 8.41 (t, *J* = 5.3 Hz, 1H, –CO–NH–), 8.07 (d, *J* = 1.3 Hz, 1H, pyridine-H), 7.41–7.30 (m, 1H, Ar–H), 7.16–6.94 (m, 3H, Ar–H), 4.10–4.00 (m, 2H, –CH_2_–), 4.00–3.93 (m, 2H, –CH_2_–), 3.13–2.79 (m, 2H, –CH_2_–). ^13^C NMR (100 MHz, CDCl_3_) *δ* 162.9 (d, *J* = 247.8 Hz), 162.8, 148.8, 143.1 (q, *J* = 3.8 Hz), 137.6 (q, *J* = 3.6 Hz), 132.1, 131.7 (d, *J* = 7.7 Hz), 130.6 (d, *J* = 8.3 Hz), 129.4 (q, *J* = 34.0 Hz), 125.9 (d, *J* = 3.0 Hz), 122.1 (q, *J* = 273.4 Hz), 117.1 (d, *J* = 22.0 Hz), 115.7 (d, *J* = 21.0 Hz), 57.8, 49.7, 34.4. ^19^F NMR (376 MHz, CDCl_3_) *δ* −62.57, −111.70. HRMS: calculated for C_16_H_14_O_3_N_2_ClF_4_S [M + H]^+^: 425.03443; found: 425.03293.

##### 3-Chloro-*N*-(2-((2,3-dichlorobenzyl)sulfonyl)ethyl)-5-(trifluoromethyl)picolinamide (F6)

Yield 44%; faint yellow solid; mp 161–162 °C. ^1^H NMR (400 MHz, CDCl_3_) *δ* 8.73 (d, *J* = 1.1 Hz, 1H, pyridine-H), 8.41 (t, *J* = 5.5 Hz, 1H, –CO–NH–), 8.07 (d, *J* = 1.3 Hz, 1H, pyridine-H), 7.48 (dd, *J* = 8.0, 1.6 Hz, 1H, Ar–H), 7.32 (dd, *J* = 7.7, 1.6 Hz, 1H, Ar–H), 7.23 (t, *J* = 7.8 Hz, 1H, Ar–H), 4.27 (dd, *J* = 39.0, 12.9 Hz, 2H, –CH_2_–), 4.01 (dd, *J* = 12.1, 6.1 Hz, 2H, –CH_2_–), 3.23–2.89 (m, 2H, –CH_2_–). ^13^C NMR (100 MHz, CDCl_3_) *δ* 162.8, 148.7, 143.1 (q, *J* = 3.8 Hz), 137.6 (q, *J* = 3.6 Hz), 134.0, 133.0, 132.1, 130.9, 130.6, 130.3, 129.4 (q, *J* = 34.0 Hz), 127.7, 122.1 (q, *J* = 273.5 Hz), 57.0, 50.2, 34.4. ^19^F NMR (376 MHz, CDCl_3_) *δ* −62.56. HRMS: calculated for C_16_H_13_O_3_N_2_Cl_3_F_4_S [M + H]^+^: 474.96591; found: 474.96579.

##### 3-Chloro-*N*-(2-((3-chloro-2-fluorobenzyl)sulfonyl)ethyl)-5-(trifluoromethyl)picolinamide (F7)

Yield 40%; pale yellow solid; mp 145–146 °C. ^1^H NMR (400 MHz, CDCl_3_) *δ* 8.73 (d, *J* = 1.1 Hz, 1H), 8.39 (t, *J* = 5.5 Hz, 1H), 8.07 (d, *J* = 1.3 Hz, 1H), 7.45–7.36 (m, 1H), 7.30–7.24 (m, 1H), 7.12 (td, *J* = 7.9, 1.0 Hz, 1H), 4.13 (ddd, *J* = 30.9, 13.2, 1.1 Hz, 2H), 4.02–3.94 (m, 2H), 3.19–2.83 (m, 2H). ^19^F NMR (376 MHz, CDCl_3_) *δ* −62.56, −117.99. ^13^C NMR (100 MHz, CDCl_3_) *δ* 162.8, 156.5 (d, *J* = 249.8 Hz), 148.7, 143.0 (q, *J* = 3.9 Hz), 137.6 (q, *J* = 3.5 Hz), 132.1, 131.2, 130.6 (d, *J* = 2.7 Hz), 129.4 (q, *J* = 34.0 Hz), 125.1 (d, *J* = 4.8 Hz), 122.1 (q, *J* = 273.4 Hz), 121.7 (d, *J* = 17.9 Hz), 118.7 (d, *J* = 15.2 Hz), 51.2, 50.1, 34.4. HRMS: calculated for C_16_H_13_O_3_N_2_Cl_2_F_4_S [M + H]^+^: 458.99546; found: 458.99557.

##### 3-Chloro-*N*-(2-((3,4-difluorobenzyl)sulfonyl)ethyl)-5-(trifluoromethyl)picolinamide (F8)

Yield 84%; white solid; mp 163–164 °C. ^1^H NMR (400 MHz, CDCl_3_) *δ* 8.74 (d, *J* = 1.1 Hz, 1H, pyridine-H), 8.32 (t, *J* = 5.5 Hz, 1H, –CO–NH–), 8.09 (d, *J* = 1.3 Hz, 1H, pyridine-H), 7.35–7.27 (m, 1H, Ar–H), 7.25–7.11 (m, 2H, Ar–H), 4.26 (s, 2H, –CH_2_–), 3.96 (dd, *J* = 12.2, 6.2 Hz, 2H, –CH_2_–), 3.44–3.07 (m, 2H, –CH_2_–). ^13^C NMR (100 MHz, CDCl_3_) *δ* 162.6, 151.1 (dd, *J* = 248.7, 9.4 Hz), 150.5 (dd, *J* = 257.0, 19.3 Hz), 148.2, 143.1 (q, *J* = 3.8 Hz), 137.8 (q, *J* = 3.5 Hz), 132.3, 129.6 (q, *J* = 34.1 Hz), 127.1 (dd, *J* = 6.6, 3.8 Hz), 124.0 (dd, *J* = 6.1, 4.1 Hz), 122.1 (d, *J* = 273.5 Hz), 120.0 (d, *J* = 18.0 Hz), 118.1 (d, *J* = 17.5 Hz), 59.4, 50.8, 33.1. ^19^F NMR (376 MHz, CDCl_3_) *δ* −62.59, −135.47 (d, ^3^*J*_F–F_ = 21.2 Hz), −135.85 (d, ^3^*J*_F–F_ = 21.2 Hz). HRMS: calculated for C_16_H_13_O_3_N_3_ClF_5_S [M + H]^+^: 443.02501; found: 443.02417.

##### *N*-(2-(Benzylsulfonyl)ethyl)-3-chloro-5-(trifluoromethyl)picolinamide (F9)

Yield 39%; soil white solid; mp 137–138 °C. ^1^H NMR (400 MHz, CDCl_3_) *δ* 8.72 (d, *J* = 1.1 Hz, 1H, pyridine-H), 8.41 (t, *J* = 4.8 Hz, 1H, –CO–NH–), 8.06 (d, *J* = 1.3 Hz, 1H, pyridine-H), 7.41–7.33 (m, 3H, Ar–H), 7.30 (dt, *J* = 5.3, 4.3 Hz, 2H, Ar–H), 4.13–4.03 (m, 2H, –CH_2_–), 3.96 (hd, *J* = 9.2, 6.1 Hz, 2H, –CH_2_–), 3.08–2.76 (m, 2H, –CH_2_–). ^13^C NMR (100 MHz, CDCl_3_) *δ* 162.7, 148.9, 143.1 (q, *J* = 3.8 Hz), 137.6 (q, *J* = 3.6 Hz), 132.1, 130.1, 129.4 (q, *J* = 23.9 Hz), 129.3, 129.1, 128.6, 122.1 (q, *J* = 273.4 Hz), 58.5, 49.3, 34.4. ^19^F NMR (376 MHz, CDCl_3_) *δ* −62.56. HRMS: calculated for C_16_H_15_O_3_N_2_ClF_3_S [M + H]^+^: 407.04385; found: 407.04343.

##### 3-Chloro-*N*-(2-(((3,4-dimethoxypyridin-2-yl)methyl)sulfonyl)ethyl)-5-(trifluoromethyl)picolinamide (F10)

Yield 35%; pale yellow solid; mp 113–114 °C. ^1^H NMR (400 MHz, CDCl_3_) *δ* 8.72 (d, *J* = 1.0 Hz, 1H, pyridine-H), 8.63 (t, *J* = 5.2 Hz, 1H, –CO–NH–), 8.21 (d, *J* = 5.5 Hz, 1H, pyridine-H), 8.05 (d, *J* = 1.3 Hz, 1H, pyridine-H), 6.82 (d, *J* = 5.5 Hz, 1H, pyridine-H), 4.37 (dd, *J* = 63.7, 12.4 Hz, 2H, –CH_2_–), 4.03 (dt, *J* = 8.4, 3.9 Hz, 2H, –CH_2_–), 3.92 (d, *J* = 3.4 Hz, 6H, –CH_3_), 3.28–3.04 (m, 2H, –CH_2_–). ^13^C NMR (100 MHz, CDCl_3_) *δ* 162.69, 158.83, 149.33, 146.07, 145.09, 144.29, 143.01 (q, *J* = 3.8 Hz), 137.40 (q, *J* = 3.5 Hz), 131.90, 129.13 (q, *J* = 34.0 Hz), 122.16 (q, *J* = 273.4 Hz), 107.74, 61.50, 55.79, 54.66, 49.85, 34.33. ^19^F NMR (376 MHz, CDCl_3_) *δ* −62.56. HRMS: calculated for C_17_H_18_O_5_N_3_ClF_3_S [M + H]^+^: 468.06023; found: 468.06030.

#### 4.2.4 The synthesis of sulfoxide-containing compounds G1–G16

According to the reported method,^14^ sulfoxide-containing compounds G1–G16 were also synthesized *via* the oxidation of thioether-containing compounds E11–E26. For G1 as an example, compound E11 (1.27 mmol) were treated with a mixture of H_2_O_2_ (26.73 mmol) and Na_2_WO_4_·2H_2_O (0.089 mmol). The resulting mixture was stirred at room temperature for about 24 h. After the completion of the reaction, solvent was removed under reduced pressure to provide crude product, which was purified by silica gel (200–300 mesh) column chromatography with ethyl acetate/petroleum ether (1 : 3). Other sulfoxide-containing compounds G2–G16 could be also synthesized with the similar method. The confirming data are listed below, and the spectra are listed in ESI data.†

##### 3-Chloro-5-(trifluoromethyl)-*N*-(2-((4-(trifluoromethyl)benzyl) sulfinyl)ethyl)picolinamide (G1)

Yield 54%; white solid; mp 184–185 °C. ^1^H NMR (400 MHz, CDCl_3_) *δ* 8.72 (s, 1H, pyridine-H), 8.37 (br, 1H, –CO–NH–), 8.07 (s, 1H, pyridine-H), 7.65 (d, *J* = 8.0 Hz, 2H, Ar–H), 7.45 (d, *J* = 7.9 Hz, 2H, Ar–H), 4.10 (dd, *J* = 37.2, 13.0 Hz, 2H, –CH_2_–), 3.96 (dt, *J* = 10.8, 5.6 Hz, 2H, –CH_2_–), 3.0 (dtd, *J* = 18.8, 12.8, 5.9 Hz, 2H, –CH_2_–). ^13^C NMR (100 MHz, CDCl_3_) *δ* 162.83, 148.61, 143.03 (q, *J* = 3.8 Hz), 137.7 (q, *J* = 3.6 Hz), 133.5, 132.2, 130.8 (q, *J* = 32.7 Hz), 130.6, 129.5 (q, *J* = 34.0 Hz), 126.0 (q, *J* = 3.7 Hz), 123.9 (q, *J* = 272.3 Hz), 122.1 (q, *J* = 273.5 Hz), 57.6, 50.0, 34.4. ^19^F NMR (376 MHz, CDCl_3_) *δ* −62.59, −62.76. HRMS: calculated for C_17_H_14_O_2_N_2_ClF_6_S [M + H]^+^: 459.03632; found: 459.03659.

##### 3-Chloro-*N*-(2-((2-chloro-4-fluorobenzyl)sulfinyl)ethyl)-5-(trifluoromethyl)picolinamide (G2)

Yield 60%; soil white solid; mp 165–166 °C. ^1^H NMR (400 MHz, CDCl_3_) *δ* 8.73 (d, *J* = 1.1 Hz, 1H, pyridine-H), 8.38 (t, *J* = 5.2 Hz, 1H, –CO–NH–), 8.12–8.02 (m, 1H, pyridine-H), 7.46–7.37 (m, 1H, Ar–H), 7.32–7.23 (m, 1H, Ar–H), 7.12 (td, *J* = 8.0, 0.8 Hz, 1H, Ar–H), 4.14 (dt, *J* = 29.9, 7.1 Hz, 2H, –CH_2_–), 3.99 (q, *J* = 6.1 Hz, 2H, –CH_2_–), 2.98 (ddt, *J* = 13.2, 11.1, 6.1 Hz, 2H, –CH_2_–). ^13^C NMR (100 MHz, CDCl_3_) *δ* 162.8, 156.5 (d, *J* = 249.8 Hz), 148.7, 143.0 (q, *J* = 3.8 Hz), 137.6 (q, *J* = 3.5 Hz), 132.1, 131.2, 130.6 (d, *J* = 2.8 Hz), 129.4 (q, *J* = 34.1 Hz), 125.0 (d, *J* = 4.8 Hz), 122.1 (q, *J* = 273.5 Hz), 121.8 (d, *J* = 18.0 Hz), 118.7 (d, *J* = 15.2 Hz), 51.3, 50.2, 34.4. ^19^F NMR (376 MHz, CDCl_3_) *δ* −62.58, −117.98. HRMS: calculated for C_16_H_13_O_2_N_2_Cl_2_F_4_S [M + H]^+^: 443.00054; found: 442.99860.

##### 3-Chloro-*N*-(2-((4-isopropylbenzyl)sulfinyl)ethyl)-5-(trifluoromethyl)picolinamide (G3)

Yield 45%; faint yellow solid; mp 180–181 °C. ^1^H NMR (400 MHz, CDCl_3_) *δ* 8.73 (d, *J* = 1.1 Hz, 1H, pyridine-H), 8.44 (t, *J* = 5.4 Hz, 1H, –CO–NH–), 8.06 (d, *J* = 1.4 Hz, 1H, pyridine-H), 7.26–7.19 (m, 4H, Ar–H), 4.05 (q, *J* = 13.0 Hz, 2H, –CH_2_–), 4.00–3.92 (m, 2H, –CH_2_–), 3.02 (ddd, *J* = 13.1, 7.4, 5.5 Hz, 1H, –CH–), 2.93–2.78 (m, 2H, –CH_2_–), 1.24 (d, *J* = 6.9 Hz, 6H, –CH_3_). ^13^C NMR (100 MHz, CDCl_3_) *δ* 162.7, 149.5, 149.0, 143.1 (q, *J* = 3.8 Hz), 137.6 (q, *J* = 3.6 Hz), 132.0, 130.1, 129.3 (q, *J* = 34.0 Hz), 127.2, 126.4, 122.1 (q, *J* = 273.5 Hz), 58.2, 49.2, 34.5, 33.9, 23.9. ^19^F NMR (376 MHz, CDCl_3_) *δ* −62.56. HRMS: calculated for C_19_H_21_O_2_N_2_ClF_3_S [M + H]^+^: 433.09589; found: 433. 09573.

##### *N*-(2-((2-Bromo-4-fluorobenzyl)sulfinyl)ethyl)-3-chloro-5-(trifluoromethyl)picolinamide (G4)

Yield 38%; faint yellow solid; mp 184–185 °C. ^1^H NMR (400 MHz, CDCl_3_) *δ* 8.73 (d, *J* = 1.1 Hz, 1H, pyridine-H), 8.38 (t, *J* = 5.1 Hz, 1H, –CO–NH–), 8.07 (d, *J* = 1.4 Hz, 1H, pyridine-H), 7.39 (ddd, *J* = 8.1, 7.5, 4.2 Hz, 2H, Ar–H), 7.11–6.99 (m, 1H, Ar–H), 4.22 (dd, *J* = 46.5, 13.1 Hz, 2H, –CH_2_–), 4.01 (ddd, *J* = 8.0, 5.5, 1.4 Hz, 2H, –CH_2_–), 3.25–2.84 (m, 2H, –CH_2_–). ^13^C NMR (100 MHz, CDCl_3_) *δ* 162.8, 162.4 (d, *J* = 253.3 Hz), 148.8, 143.1 (q, *J* = 3.9 Hz), 137.62 (q, *J* = 3.6 Hz), 133.5 (d, *J* = 8.6 Hz), 132.1, 129.4 (q, *J* = 34.1 Hz), 125.8 (d, *J* = 3.7 Hz), 125.2 (d, *J* = 9.7 Hz), 122.1 (q, *J* = 273.4 Hz), 120.7 (d, *J* = 24.7 Hz), 115.3 (d, *J* = 21.2 Hz), 57.7, 49.9, 34.5. ^19^F NMR (376 MHz, CDCl_3_) *δ* −62.58, −110.16. HRMS: calculated for C_16_H_13_O_2_N_2_BrClF_4_S [M + H]^+^: 489.95003; found: 489.95001.

##### *N*-(2-((3-Bromobenzyl)sulfinyl)ethyl)-3-chloro-5-(trifluoromethyl)picolinamide (G5)

Yield 50%; white solid; mp 158–159 °C. ^1^H NMR (400 MHz, CDCl_3_) *δ* 8.73 (d, *J* = 1.0 Hz, 1H, pyridine-H), 8.41 (t, *J* = 5.4 Hz, 1H, –CO–NH–), 8.07 (d, *J* = 1.3 Hz, 1H, pyridine-H), 7.52–7.45 (m, 2H, Ar–H), 7.33–7.19 (m, 2H, Ar–H), 4.06–3.99 (m, 2H, –CH_2_–), 3.99–3.93 (m, 2H, –CH_2_–), 3.21–2.69 (m, 2H, –CH_2_–). ^13^C NMR (100 MHz, CDCl_3_) *δ* 162.8, 148.7, 143.1 (q, *J* = 3.8 Hz), 137.7 (q, *J* = 3.5 Hz), 133.0, 132.1, 131.8, 131.7, 130.6, 129.4 (q, *J* = 34.1 Hz), 128.8, 123.0, 122.1 (q, *J* = 273.4 Hz), 57.7, 49.8, 34.4. ^19^F NMR (376 MHz, CDCl_3_) *δ* −62.56. HRMS: calculated for C_16_H_14_O_2_N_2_BrClF_3_S [M + H]^+^: 468.95945; found: 468.95941.

##### 3-Chloro-*N*-(2-((2,6-difluorobenzyl)sulfinyl)ethyl)-5-(trifluoromethyl)picolinamide (G6)

Yield 40%; white solid; mp 144–145 °C. ^1^H NMR (400 MHz, CDCl_3_) *δ* 8.73 (d, *J* = 1.1 Hz, 1H, pyridine-H), 8.43 (t, *J* = 5.3 Hz, 1H, –CO–NH–), 8.06 (d, *J* = 1.3 Hz, 1H, pyridine-H), 7.33 (tt, *J* = 8.4, 6.5 Hz, 1H, Ar–H), 7.04–6.91 (m, 2H, Ar–H), 4.30–4.15 (m, 2H, –CH_2_–), 4.01 (ddd, *J* = 8.4, 5.3, 1.6 Hz, 2H, –CH_2_–), 3.20–2.86 (m, 2H, –CH_2_–). ^13^C NMR (100 MHz, CDCl_3_) *δ* 161.5 (d, *J* = 250.7 Hz), 161.5 (d, *J* = 250.7 Hz), 148.9, 143.1 (q, *J* = 3.9 Hz), 137.5 (q, *J* = 3.6 Hz), 132.0, 130.8 (t, *J* = 10.2 Hz), 129.3 (q, *J* = 34.0 Hz), 122.1 (q, *J* = 273.5 Hz), 111.8 (q, *J* = 25.3 Hz), 106.3 (t, *J* = 19.4 Hz), 50.2, 45.8, 34.4. ^19^F NMR (376 MHz, CDCl_3_) *δ* −62.57, −112.09. HRMS: calculated for C_16_H_13_O_2_N_2_ClF_5_S [M + H]^+^: 427.03009; found: 427.03006.

##### 3-Chloro-*N*-(2-((2-methylbenzyl)sulfinyl)ethyl)-5-(trifluoromethyl)picolinamide (G7)

Yield 52%; white solid; mp 136–137 °C. ^1^H NMR (400 MHz, CDCl_3_) *δ* 8.72 (d, *J* = 0.9 Hz, 1H, pyridine-H), 8.45 (t, *J* = 5.1 Hz, 1H, –CO–NH–), 8.06 (d, *J* = 1.3 Hz, 1H, pyridine-H), 7.26–7.17 (m, 4H, Ar–H), 4.15 (dd, *J* = 52.1, 12.9 Hz, 2H, –CH_2_–), 4.05–3.91 (m, 2H, –CH_2_–), 3.13–2.87 (m, 2H, –CH_2_–), 2.41 (s, 3H, –CH_3_). ^13^C NMR (100 MHz, CDCl_3_) *δ* 162.7, 149.0, 143.1 (q, *J* = 3.8 Hz), 137.5 (q, *J* = 7.2 Hz), 137.4, 132.0, 131.1, 131.0, 129.3 (q, *J* = 34.0 Hz), 128.9, 128.0, 126.7, 122.1 (q, *J* = 273.5 Hz), 57.2, 49.6, 34.5, 19.9. ^19^F NMR (376 MHz, CDCl_3_) *δ* −62.56. HRMS: calculated for C_17_H_17_O_2_N_2_ClF_3_S [M + H]^+^: 405.06459; found: 495.06454.

##### 3-Chloro-5-(trifluoromethyl)-*N*-(2-((2-(trifluoromethyl)benzyl)sulfinyl)ethyl)picolinamide (G8)

Yield 35%; white solid; mp 131–132 °C. ^1^H NMR (400 MHz, CDCl_3_) *δ* 8.73 (d, *J* = 1.0 Hz, 1H, pyridine-H), 8.43 (t, *J* = 5.2 Hz, 1H, –CO–NH–), 8.07 (d, *J* = 1.3 Hz, 1H, pyridine-H), 7.72 (d, *J* = 7.8 Hz, 1H, Ar–H), 7.61–7.54 (m, 2H, Ar–H), 7.49 (td, *J* = 8.2, 4.7 Hz, 1H, Ar–H), 4.29–4.12 (m, 2H, –CH_2_–), 4.10–3.94 (m, 2H, –CH_2_–), 3.21–2.93 (m, 2H, –CH_2_–). ^13^C NMR (100 MHz, CDCl_3_) *δ* 162.7, 148.8, 143.1 (q, *J* = 3.8 Hz), 137.6 (q, *J* = 3.6 Hz), 132.9, 132.3, 132.1, 129.3 (q, *J* = 34.1 Hz), 129.1 (q, *J* = 30.1 Hz), 128.8, 128.8, 126.7 (q, *J* = 5.5 Hz), 124.1 (q, *J* = 273.9 Hz), 122.1 (q, *J* = 273.5 Hz), 56.2, 50.7, 34.5. ^19^F NMR (376 MHz, CDCl_3_) *δ* −58.50, −62.58. HRMS: calculated for C_17_H_14_O_2_N_2_ClF_6_S [M + H]^+^: 459.03632; found: 459.03586.

##### 3-Chloro-*N*-(2-((2-chlorobenzyl)sulfinyl)ethyl)-5-(trifluoromethyl)picolinamide (G9)

Yield 45%; faint yellow solid; mp 127–128 °C. ^1^H NMR (400 MHz, CDCl_3_) *δ* 8.73 (d, *J* = 1.1 Hz, 1H, pyridine-H), 8.42 (t, *J* = 5.2 Hz, 1H, –CO–NH–), 8.06 (d, *J* = 1.3 Hz, 1H, pyridine-H), 7.46–7.38 (m, 2H, Ar–H), 7.34–7.28 (m, 2H, Ar–H), 4.26 (dd, *J* = 29.2, 13.0 Hz, 2H, –CH_2_–), 4.08–3.92 (m, 2H, –CH_2_–), 3.20–2.85 (m, 2H, –CH_2_–). ^13^C NMR (100 MHz, CDCl_3_) *δ* 162.7, 148.9, 143.1 (q, *J* = 3.9 Hz), 137.6 (q, *J* = 3.6 Hz), 134.6, 132.5, 132.1, 130.1, 130.0, 129.3 (q, *J* = 34.0 Hz), 127.8, 127.4, 122.1 (q, *J* = 273.5 Hz), 56.1, 49.8, 34.5. ^19^F NMR (376 MHz, CDCl_3_) *δ* −62.55. HRMS: calculated for C_15_H_14_O_3_N_2_Cl_2_F_3_S [M + H]^+^: 441.00488; found: 441.00427.

##### *N*-(2-((4-(*tert*-Butyl)benzyl)sulfinyl)ethyl)-3-chloro-5-(trifluoromethyl)picolinamide (G10)

Yield 55%; faint creamy yellow solid; mp 131–133 °C. ^1^H NMR (400 MHz, CDCl_3_) *δ* 8.73 (d, *J* = 0.9 Hz, 1H, pyridine-H), 8.45 (t, *J* = 5.4 Hz, 1H, –CO–NH–), 8.06 (d, *J* = 1.2 Hz, 1H, pyridine-H), 7.40 (d, *J* = 8.2 Hz, 2H, Ar–H), 7.23 (d, *J* = 8.2 Hz, 2H, Ar–H), 4.05 (q, *J* = 13.0 Hz, 2H, –CH_2_–), 4.00–3.93 (m, 2H, –CH_2_–), 3.09–2.78 (m, 2H, –CH_2_–), 1.31 (s, 9H, –C(CH_3_)_3_). ^13^C NMR (100 MHz, CDCl_3_) *δ* 162.8, 151.7, 149.0, 143.1 (q, *J* = 3.8 Hz), 137.6 (q, *J* = 3.6 Hz), 132.0, 129.8, 129.3 (d, *J* = 34.0 Hz), 126.1, 126.1, 122.1 (q, *J* = 273.4 Hz), 58.1, 49.2, 34.7, 34.5, 31.3. ^19^F NMR (376 MHz, CDCl_3_) *δ* −62.56. HRMS: calculated for C_20_H_23_O_2_N_2_ClF_3_S [M + H]^+^: 447.1154; found: 477.11038.

##### 3-Chloro-*N*-(2-(((6-chloropyridin-3-yl)methyl)sulfinyl)ethyl)-5-(trifluoromethyl)picolinamide (G11)

Yield 72%; soil yellow solid; mp 136–137 °C. ^1^H NMR (400 MHz, CDCl_3_) *δ* 8.73 (d, *J* = 1.1 Hz, 1H, pyridine-H), 8.38 (t, *J* = 5.2 Hz, 1H, –CO–NH–), 8.35 (d, *J* = 2.3 Hz, 1H, pyridine-H), 8.08 (d, *J* = 1.3 Hz, 1H, pyridine-H), 7.68 (dd, *J* = 8.2, 2.4 Hz, 1H, pyridine-H), 7.37 (d, *J* = 8.2 Hz, 1H, pyridine-H), 4.16–3.86 (m, 4H, –CH_2_–), 3.25–2.76 (m, 2H, –CH_2_–). ^13^C NMR (100 MHz, CDCl_3_) *δ* 162.9, 151.9, 150.7, 148.4, 143.0 (q, *J* = 3.8 Hz), 140.4, 137.8 (q, *J* = 3.6 Hz), 132.2, 129.5 (q, *J* = 34.0 Hz), 124.7, 124.5, 122.1 (q, *J* = 273.5 Hz), 53.9, 50.3, 34.3. ^19^F NMR (376 MHz, CDCl_3_) *δ* −62.56. HRMS: calculated for C_15_H_13_O_2_N_3_Cl_2_F_3_S [M + H]^+^: 426.00521; found: 426.00473.

##### 3-Chloro-*N*-(2-((4-cyanobenzyl)sulfinyl)ethyl)-5-(trifluoromethyl)picolinamide (G12)

Yield 39%; pale yellow solid; mp 172–173 °C. ^1^H NMR (400 MHz, CDCl_3_) *δ* 8.72 (s, 1H, pyridine-H), 8.36 (t, *J* = 5.3 Hz, 1H, –CO–NH–), 8.08 (s, 1H, pyridine-H), 7.69 (d, *J* = 7.7 Hz, 2H, Ar–H), 7.45 (d, *J* = 7.9 Hz, 2H, Ar–H), 4.09 (dd, *J* = 53.4, 13.0 Hz, 2H, –CH_2_–), 4.00–3.83 (m, 2H, –CH_2_–), 3.31–2.77 (m, 2H, –CH_2_–). ^13^C NMR (100 MHz, CDCl_3_) *δ* 162.9, 148.5, 143.0 (q, *J* = 3.8 Hz), 137.8 (q, *J* = 3.6 Hz), 135.0, 132.7, 132.2, 131.0, 129.5 (q, *J* = 34.1 Hz), 122.1 (q, *J* = 273.5 Hz), 118.3, 112.6, 57.6, 50.3, 34.3. ^19^F NMR (376 MHz, CDCl_3_) *δ* −62.56. HRMS: calculated for C_17_H_11_O_2_N_3_Cl_2_F_3_S [M + H]^+^: 432.03910; found: 432.03787.

##### 3-Chloro-*N*-(2-((3,5-difluorobenzyl)sulfinyl)ethyl)-5-(trifluoromethyl)picolinamide (G13)

Yield 34%; soil white solid; mp 154–155 °C. ^1^H NMR (400 MHz, CDCl_3_) *δ* 8.73 (d, *J* = 1.1 Hz, 1H, pyridine-H), 8.40 (t, *J* = 5.5 Hz, 1H, –CO–NH–), 8.08 (d, *J* = 1.4 Hz, 1H, pyridine-H), 6.93–6.85 (m, 2H, Ar–H), 6.82 (ddt, *J* = 11.2, 8.9, 2.5 Hz, 1H, Ar–H), 4.11–3.87 (m, 4H, –CH_2_–), 3.23–2.80 (m, 2H, –CH_2_–). ^13^C NMR (100 MHz, CDCl_3_) *δ* 163.2 (d, *J* = 250.3 Hz), 163.0 (d, *J* = 250.3 Hz), 162.9, 148.6, 143.1 (q, *J* = 3.9 Hz), 137.7 (q, *J* = 3.5 Hz), 133.2 (t, *J* = 9.6 Hz), 132.2, 129.5 (q, *J* = 34.1 Hz), 122.1 (q, *J* = 273.4 Hz), 113.2 (dd, *J* = 11.4 Hz, 25.9 Hz), 104.3 (t, *J* = 25.1 Hz), 57.5, 50.2, 34.3. ^19^F NMR (376 MHz, CDCl_3_) *δ* −62.58, −108.29. HRMS: calculated for C_16_H_13_O_2_N_2_ClF_5_S [M + H]^+^: 427.03009; found: 427.02844.

##### 3-Chloro-*N*-(2-(((3-methyl-4-(2,2,2-trifluoroethoxy)pyridin-2-yl)methyl)sulfinyl)ethyl)-5-(trifluoromethyl)picolinamide (G14)

Yield 41%; white solid; mp 126–127 °C. ^1^H NMR (400 MHz, CDCl_3_) *δ* 8.72 (d, *J* = 1.0 Hz, 1H, pyridine-H), 8.62 (t, *J* = 5.1 Hz, 1H, –CO–NH–), 8.35 (d, *J* = 5.6 Hz, 1H, pyridine-H), 8.06 (d, *J* = 1.4 Hz, 1H, pyridine-H), 6.70 (d, *J* = 5.6 Hz, 1H, pyridine-H), 4.51–4.25 (m, 4H, –CH_2_–), 4.02 (dd, *J* = 11.8, 5.9 Hz, 2H, –CH_2_–), 3.50–2.79 (m, 2H, –CH_2_–), 2.32 (s, 3H, –CH_3_). ^13^C NMR (100 MHz, CDCl_3_) *δ* 162.7, 161.9, 150.9, 149.2, 148.4, 143.0 (q, *J* = 3.8 Hz), 137.5 (q, *J* = 3.5 Hz), 132.0, 129.2 (q, *J* = 34.0 Hz), 122.8 (q, *J* = 277.7 Hz), 122.141 (q, *J* = 273.5 Hz), 123.4, 105.9, 65.4 (q, *J* = 36.5 Hz), 57.5, 50.2, 34.3, 11.2. ^19^F NMR (376 MHz, CDCl_3_) *δ* −62.57, −73.78. HRMS: calculated for C_18_H_17_O_3_N_3_ClF_6_S [M + H]^+^: 504.05779; found: 504.05603.

##### 3-Chloro-*N*-(2-((3,4,4-trifluorobut-3-en-1-yl)sulfinyl)ethyl)-5-(trifluoromethyl)picolinamide (G15)

Yield 30%; pale yellow solid; mp 100–101 °C. ^1^H NMR (400 MHz, CDCl_3_) *δ* 8.74 (s, 1H, pyridine-H), 8.45 (s, 1H, –CO–NH–), 8.08 (d, *J* = 1.2 Hz, 1H, pyridine-H), 4.01 (dd, *J* = 11.8, 5.9 Hz, 2H, –CH_2_–), 3.31–2.94 (m, 2H, –CH_2_–), 2.86 (ddd, *J* = 12.6, 10.7, 8.4 Hz, 2H, –CH_2_–). ^13^C NMR (100 MHz, CDCl_3_) *δ* 162.8, 153.1 (ddd, *J* = 288.2, 275.3, 45.7 Hz), 148.6, 143.1 (q, *J* = 3.8 Hz), 137.7 (q, *J* = 3.5 Hz), 132.2, 129.5 (q, *J* = 34.4 Hz), 126.3 (ddd, *J* = 234.8, 53.5, 17.5 Hz), 122.1 (q, *J* = 273.5 Hz), 51.2, 48.1, 34.5, 19.8 (dd, *J* = 22.2, 2.3 Hz). ^19^F NMR (376 MHz, CDCl_3_) *δ* −62.59, −102.12 (dd, *J* = 82.1, 33.0 Hz), −121.38 (ddd, *J* = 115.7, 82.5, 4.4 Hz), −175.45 (dd, *J* = 114.8, 33.0 Hz). HRMS: calculated for C_13_H_11_O_2_N_3_Cl_2_F_3_S_2_ [M + H]^+^: 431.96163; found: 431.96161.

##### 3-Chloro-*N*-(2-((2,5-difluorobenzyl)sulfinyl)ethyl)-5-(trifluoromethyl)picolinamide (G16)

Yield 30%; white solid; mp 123–125 °C. ^1^H NMR (400 MHz, CDCl_3_) *δ* 8.73 (d, *J* = 0.9 Hz, 1H), 8.40 (t, *J* = 4.8 Hz, 1H), 8.07 (d, *J* = 1.4 Hz, 1H), 7.18–6.93 (m, 2H), 4.08 (dd, *J* = 36.3, 13.2 Hz, 2H), 3.98 (dd, *J* = 12.0, 6.1 Hz, 2H), 3.17–2.82 (m, 2H). ^13^C NMR (100 MHz, CDCl_3_) *δ* 162.8, 158.5 (dd, *J* = 244.3, 2.3 Hz), 157.0 (dd, *J* = 243.6, 2.6 Hz), 148.7, 143.0 (q, *J* = 3.8 Hz), 137.6 (q, *J* = 3.5 Hz), 132.1, 129.4 (q, *J* = 34.1 Hz), 122.1 (q, *J* = 273.4 Hz), 118.9 (d, *J* = 3.6 Hz), 118.6 (d, *J* = 3.6 Hz), 118.4 (dd, *J* = 17.9, 8.3 Hz), 117.0 (td, *J* = 24.4, 8.6 Hz), 50.8, 50.0, 34.4. ^19^F NMR (376 MHz, CDCl_3_) *δ* −62.57, −117.43 (d, *J* = 17.9 Hz), −122.28 (d, *J* = 17.8 Hz). HRMS: calculated for C_16_H_13_O_2_N_2_ClF_5_S [M + H]^+^: 427.03009; found: 427.02939.

#### 4.2.5 *In vitro* antibacterial activities against *Xoo* and *R. solanacearum* test

According to the reported procedure,^49^*in vitro* antibacterial activities of title compounds against *Xoo* and *R. solanacearum* were carried out. Firstly, 5 mL nutrient broths (NB) containing synthesized compounds solution (100 mg L^−1^ or 50 mg L^−1^) were prepared. NB containing commercialized thiodiazole copper (TC) or bismerthiazol (BT) solution (100 mg L^−1^ or 50 mg L^−1^) were also prepared as the positive controls, and NB containing sterile distilled water was used as negative control. Following this, 40 μL *Xoo* or *R. solanacearum*, was added to each NB medium. The inoculated samples above were cultured together in a shaker (180 rpm, 28 °C) for about 24–48 h, until negative control had grown to logarithmic phase. Using a microplate reader, the turbidity of each inoculated samples was measured under 595 nm, which was corrected by the equation of *T* = OD_bacterial_ − OD_no bacterial_. Then the final activities against *Xoo* or *R. solanacearum*, was calculated by the following equation:Activity: *I* = (*C* − *T*)/*C* × 100%,*C* represents the turbidity of the NB without treatment solution (negative control), and *T* represents the corrected turbidity. The antibacterial activities of synthesized compounds against *Xoo* or *R. solanacearum*, were tested for three times.

#### 4.2.6 Insecticidal activity test against *P. xylostella*

The insecticidal activity was tested at 25 ± 1 °C according to statistical requirements. Mortalities were calculated and based on a percentage scale using Abbott's formula.^50^ Using previously procedures,^51^ fresh cabbage discs (diameter 9 cm) were dipped into the synthesized solutions (500 mg L^−1^) and placed in a Petri dish with two moist filter papers. The chlorpyrifos and avermectin were used as positive control and water without any compounds was used as negative control at the same condition. Fifteen larvae of second instar *P. xylostella* were carefully transferred to the Petri dish and cultivated for 72 h. Three replicates were measured for each treatment.

## Conflicts of interest

There are no conflicts to declare.

## Supplementary Material

RA-010-D0RA07301F-s001
