# Supplementary material for: Synthesis and biological activities of novel trifluoromethylpyridine amide derivatives containing sulfur moieties
Source: RSC Adv. 2020 Sep 28;10(59):35658–70. doi: 10.1039/d0ra07301f (PMC9056882; doi:10.1039/d0ra07301f)
Supplement: RA-010-D0RA07301F-s001 [file RA-010-D0RA07301F-s001.pdf]

Electronic Supplementary Material (ESI) for RSC Advances.
This journal is © The Royal Society of Chemistry 2020

Supporting information

Synthesis and biological activities of novel trifluoromethylpyridine amide derivatives containing sulfur moiety

S. X. Guo,[a†] F. He,[a†] A. L. Dai,[a] R. F. Zhang,[a] S. H. Chen,[a] and J. Wu[a‡]

[a]State Key Laboratory Breeding Base of Green Pesticide and Agricultural Bioengineering, Key Laboratory of Green Pesticide and Agricultural Bioengineering, Ministry of Education, Research and Development Center for Fine Chemicals, Guizhou University, Huaxi District, Guiyang 550025, P.R. China;

† Co-first author for the manuscript.

‡To whom correspondence should be addressed. Tel.: +86(851)88292090; fax: +86(851) 88292090. E-mails: jianwu2691@126.com, jwu6@gzu.edu.cn

The ^{1}H, ^{13}C, ^{19}F NMR spectra of the title compounds

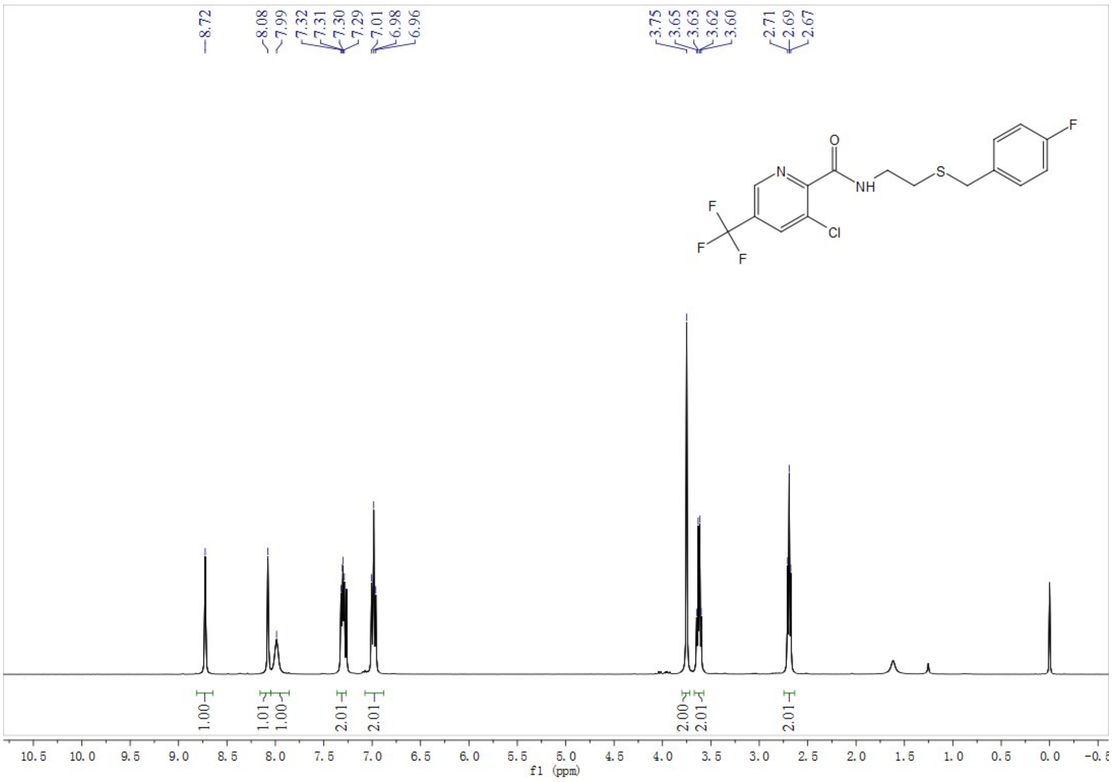

Fig. S1 ^{1}H NMR spectra of compound **E1**

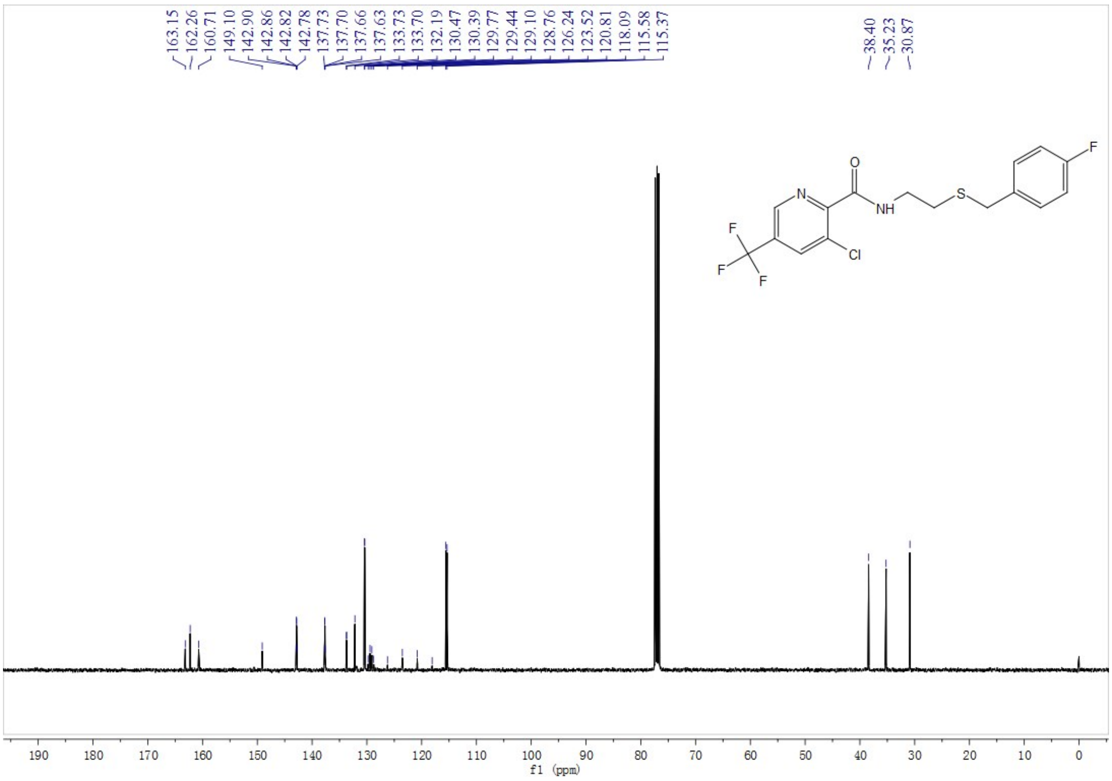

Fig. S2 ^{13}C NMR spectra of compound **E1**

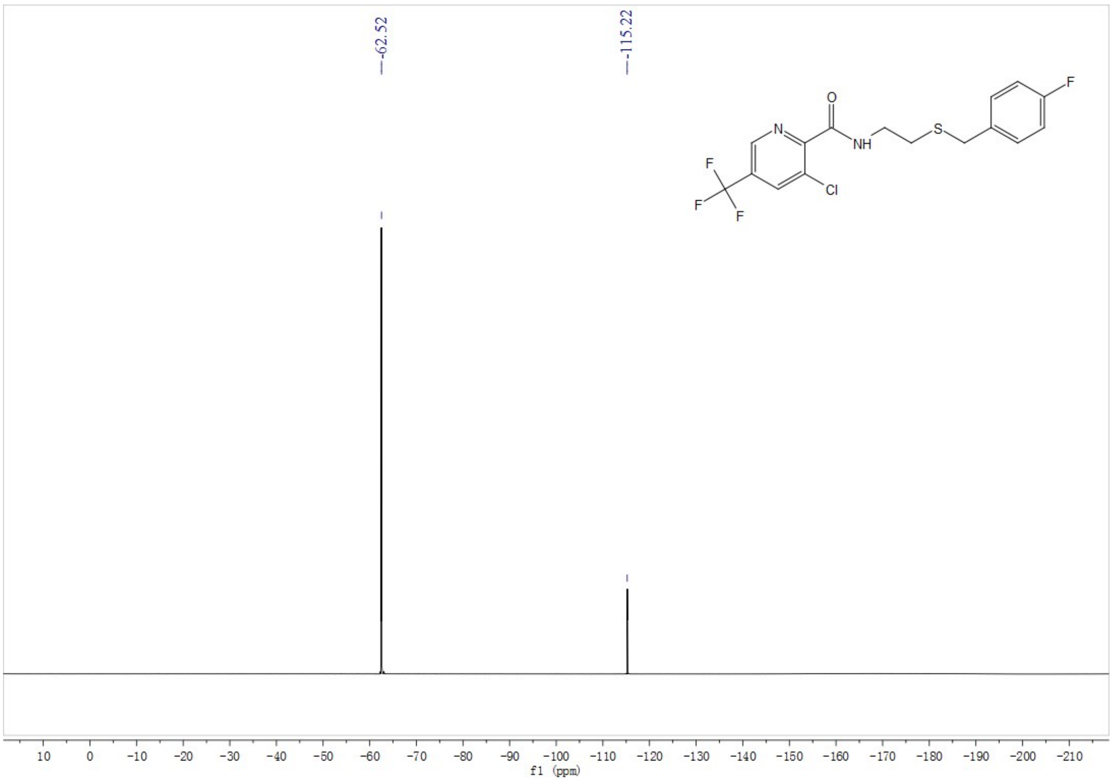

Fig. S3 ^{19}F NMR spectra of compound **E1**

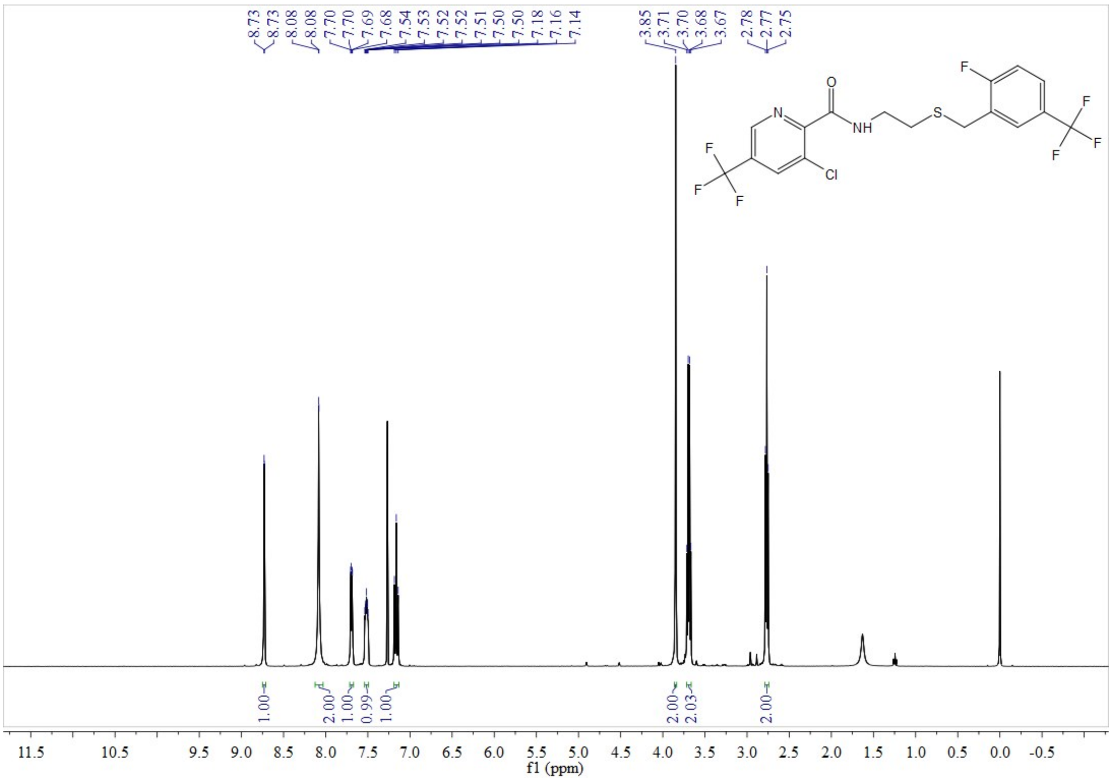

Fig. S4 ^{1}H NMR spectra of compound **E2**

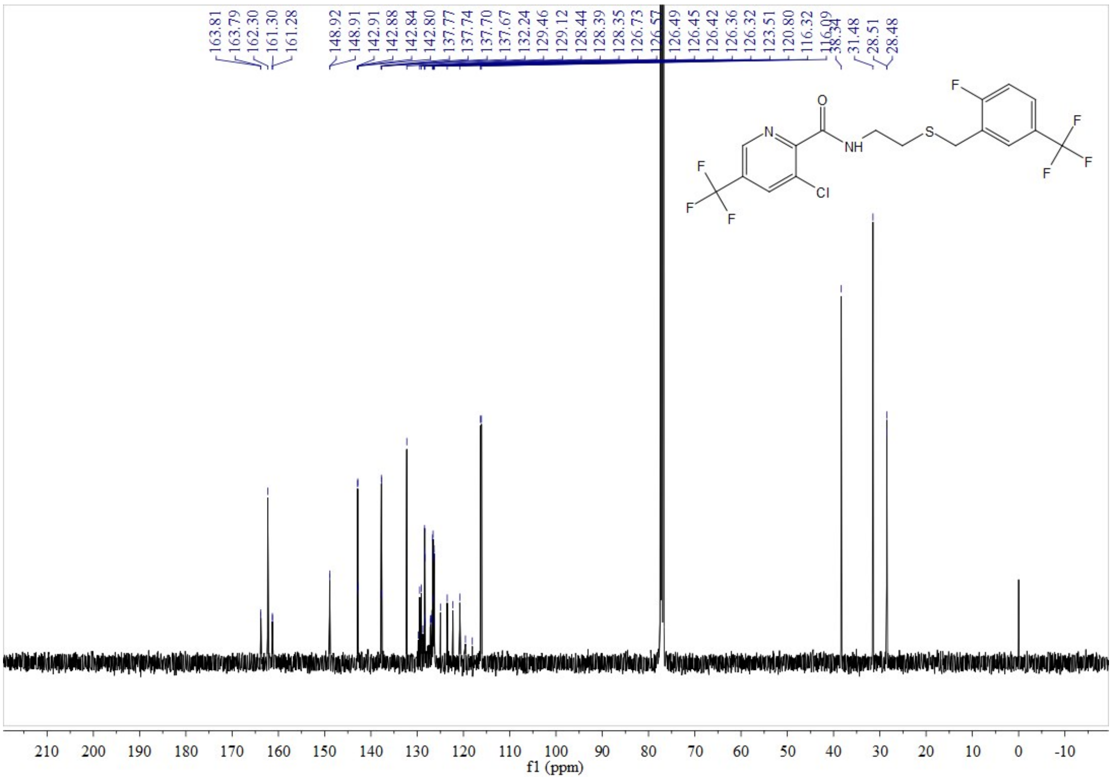

Fig. S5 ^{13}C NMR spectra of compound **E2**

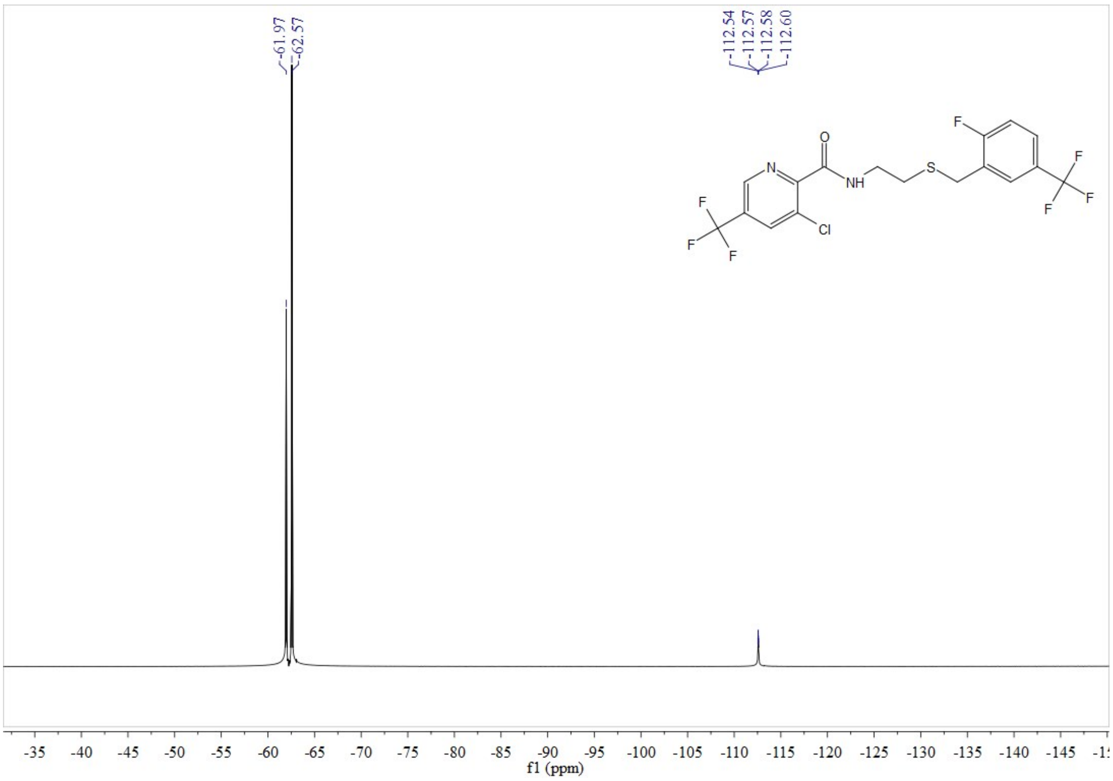

Fig. S6 ^{19}F NMR spectra of compound **E2**

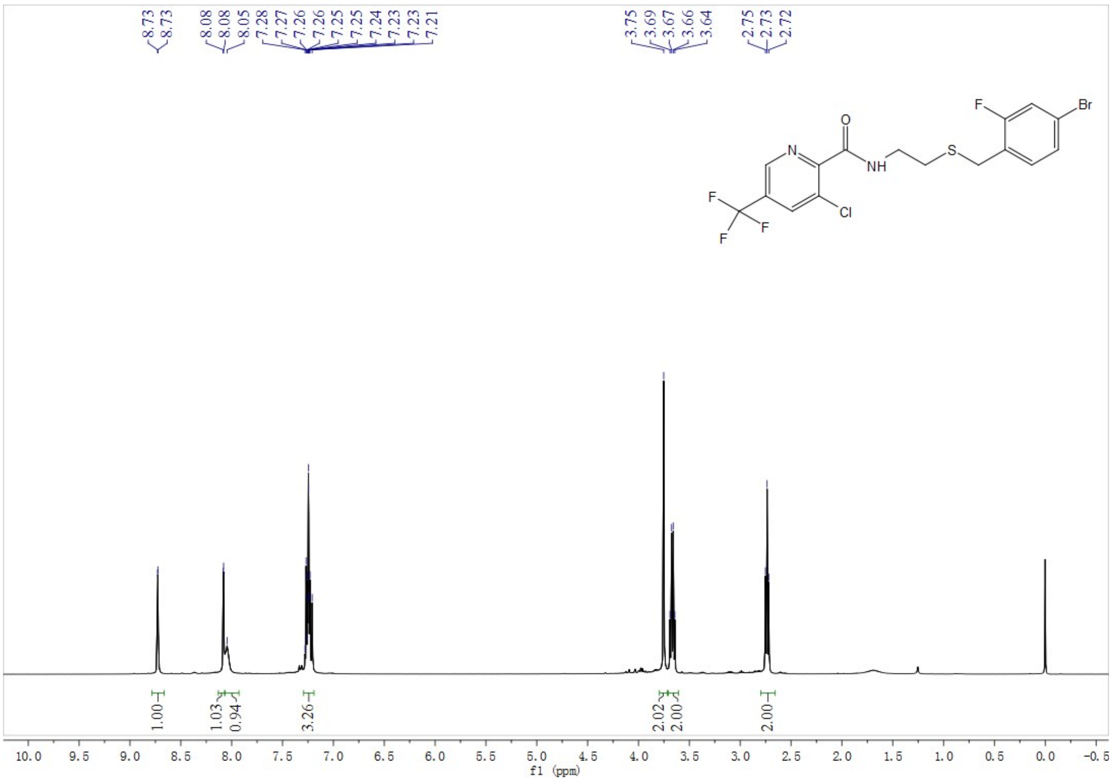

Fig. S7 ^{1}H NMR spectra of compound **E 3**

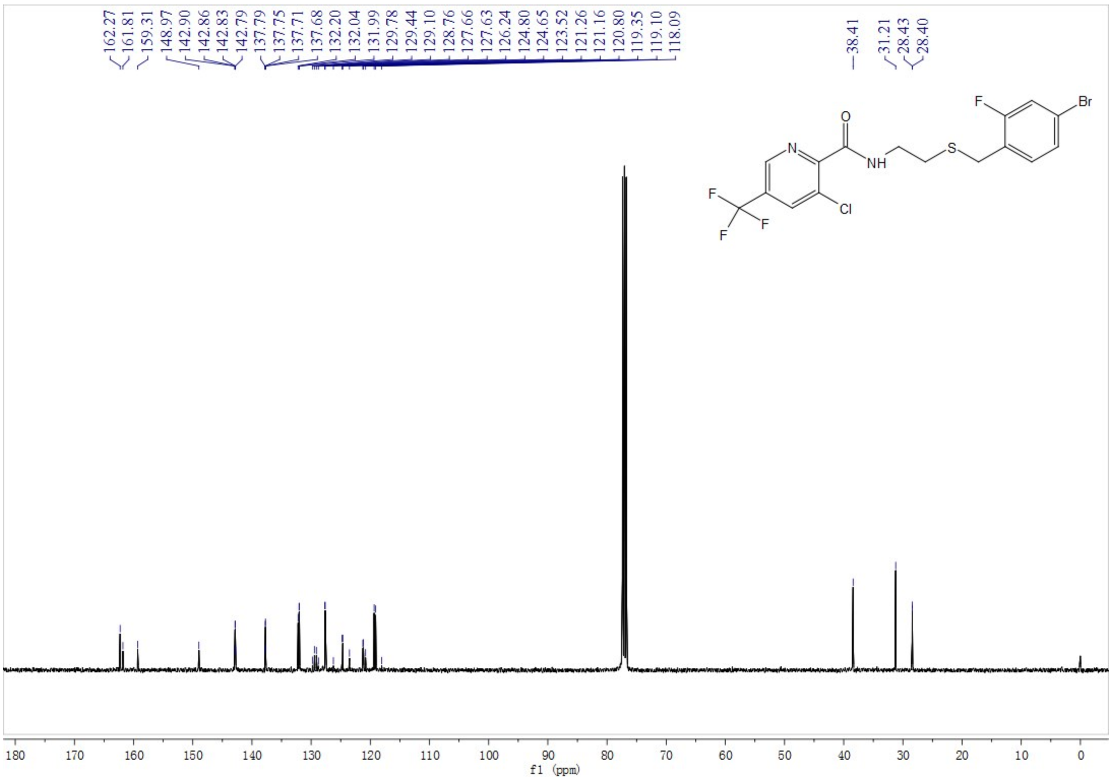

Fig. S8 ^{13}C NMR spectra of compound **E3**

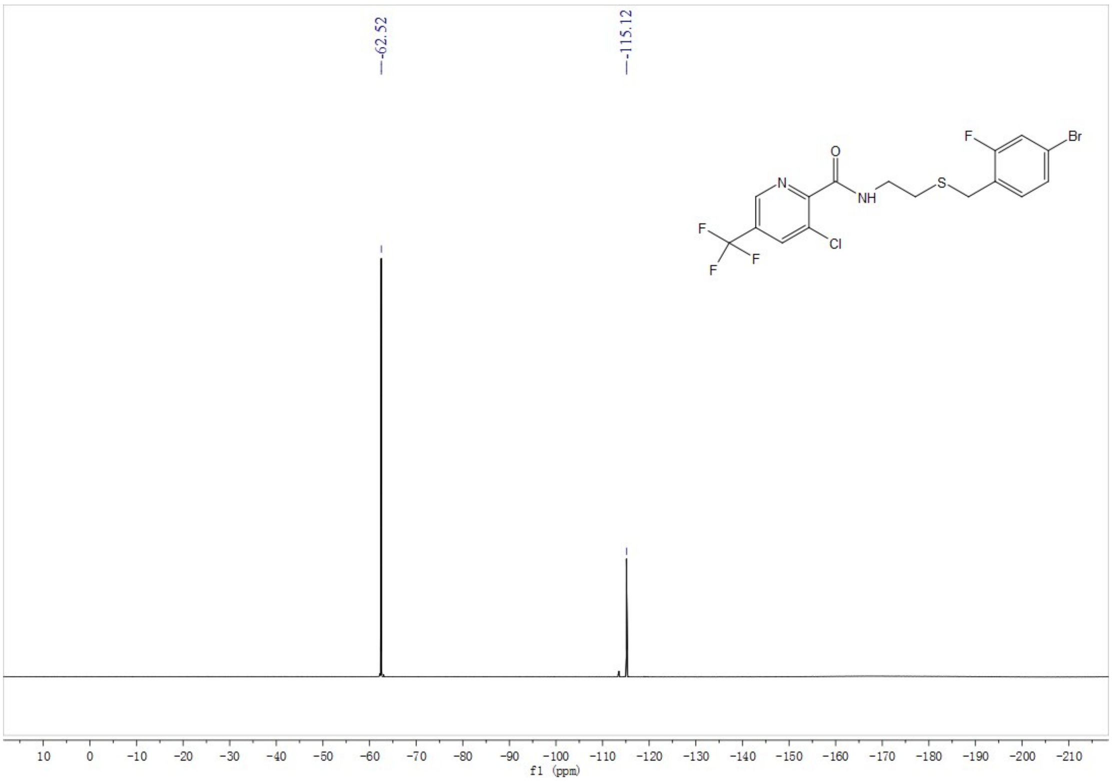

Fig. S9 19F NMR spectra of compound **E3**

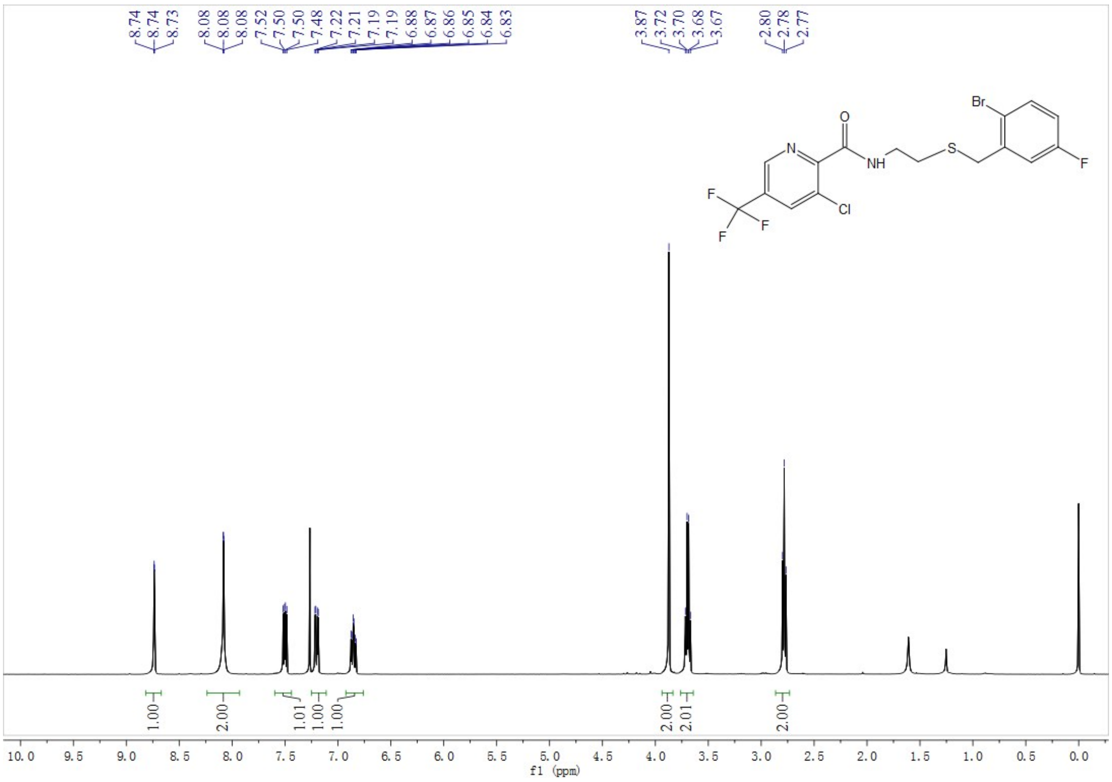

Fig. S10 ^{1}H NMR spectra of compound **E4**

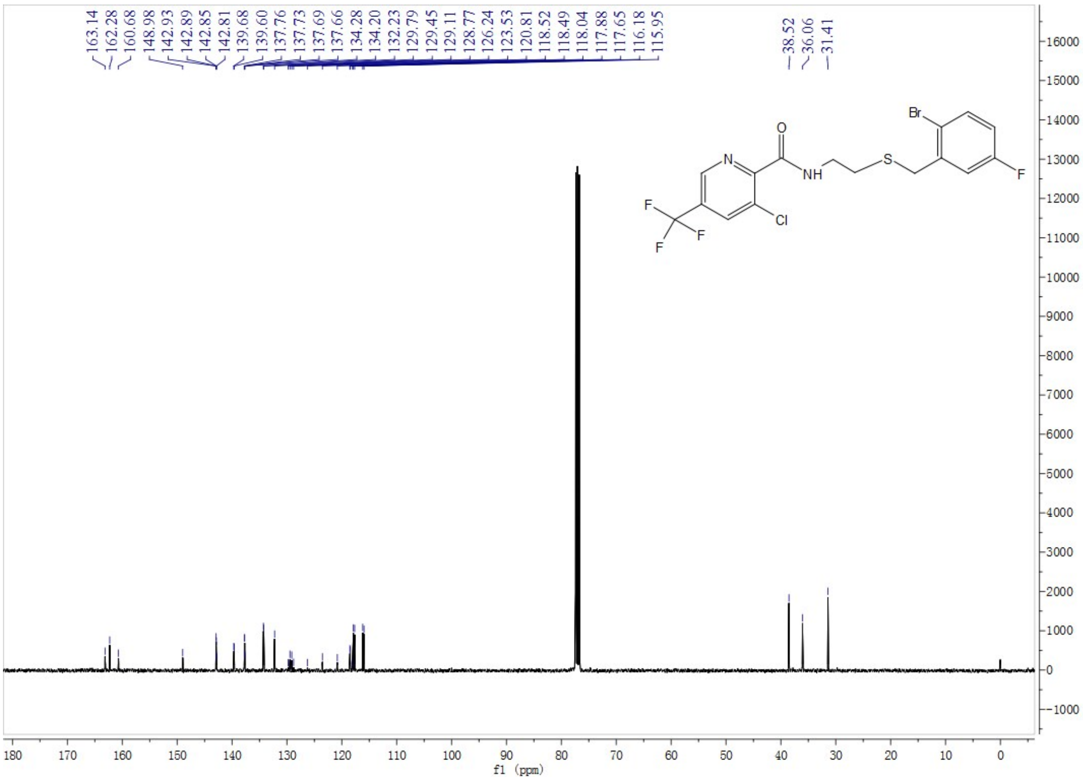

Fig. S11 ^{13}C NMR spectra of compound **E4**

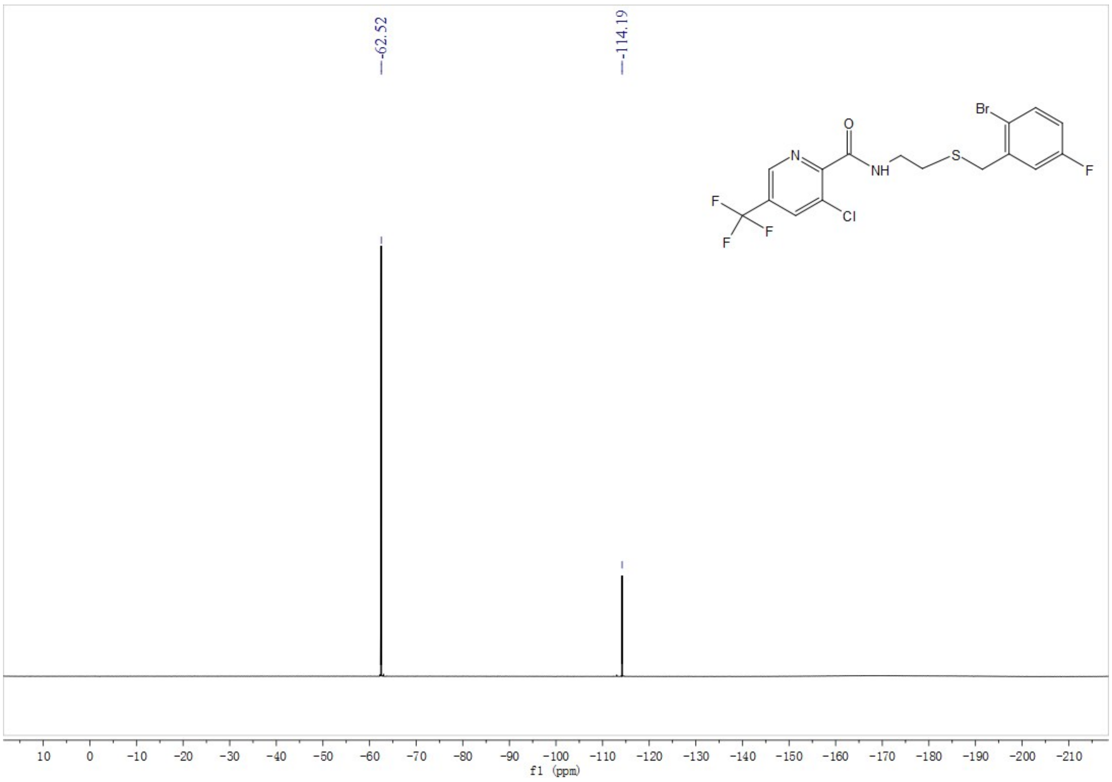

Fig. S12 ^{19}F NMR spectra of compound **E4**

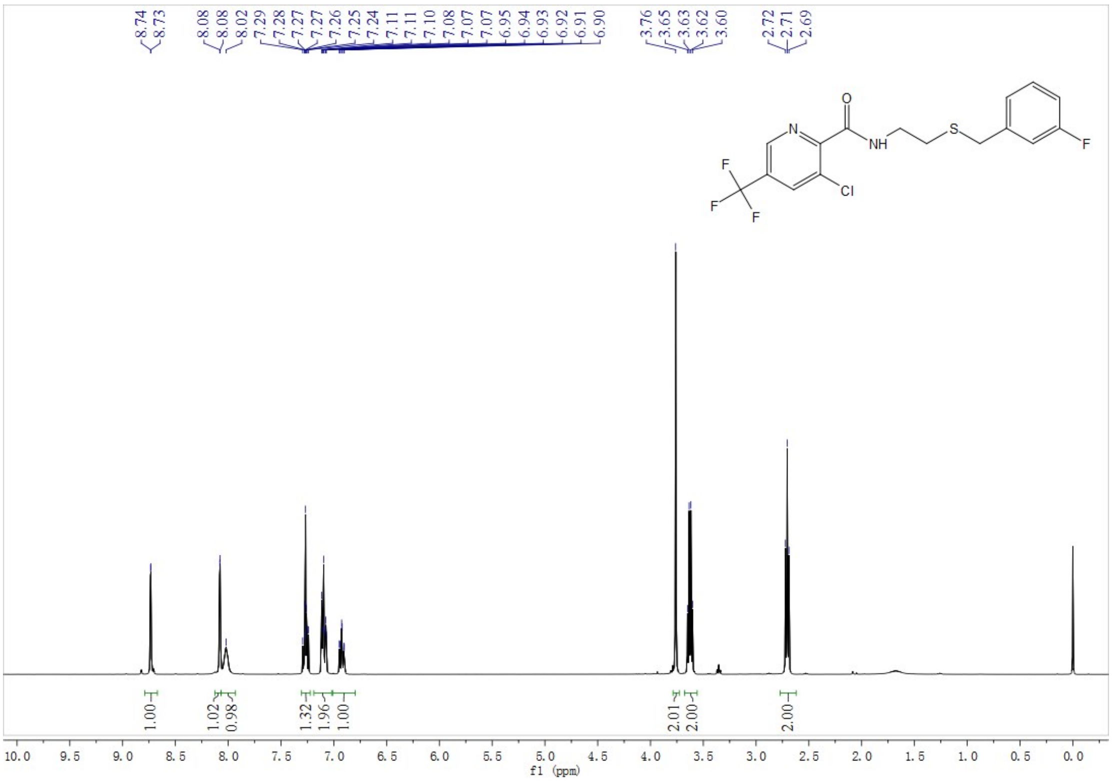

Fig. S13 ¹H NMR spectra of compound **E5**

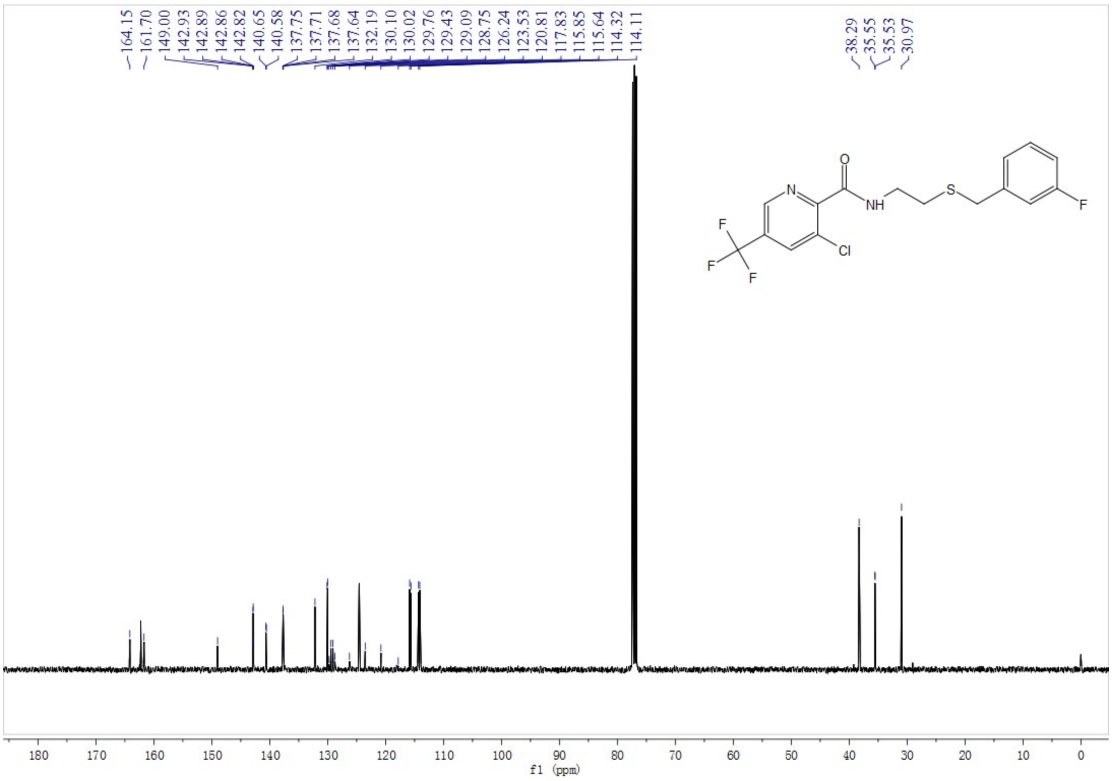

Fig. S14 ¹³C NMR spectra of compound **E5**

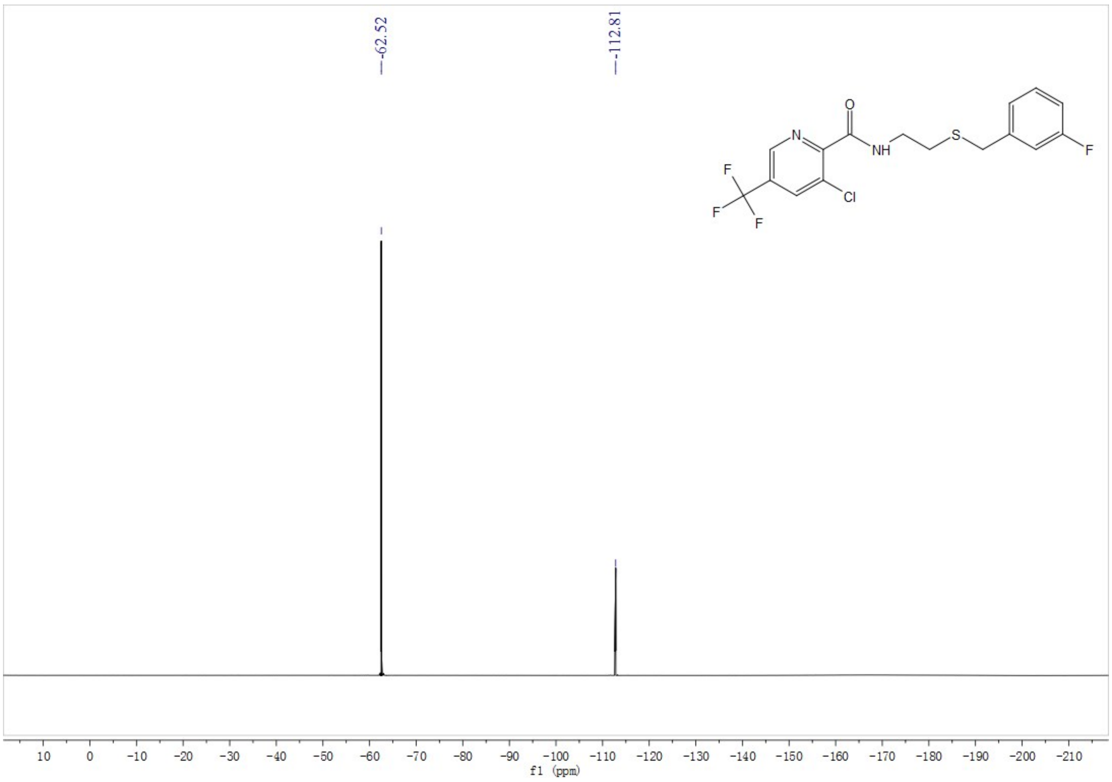

Fig. S15 ^{19}F NMR spectra of compound **E5**

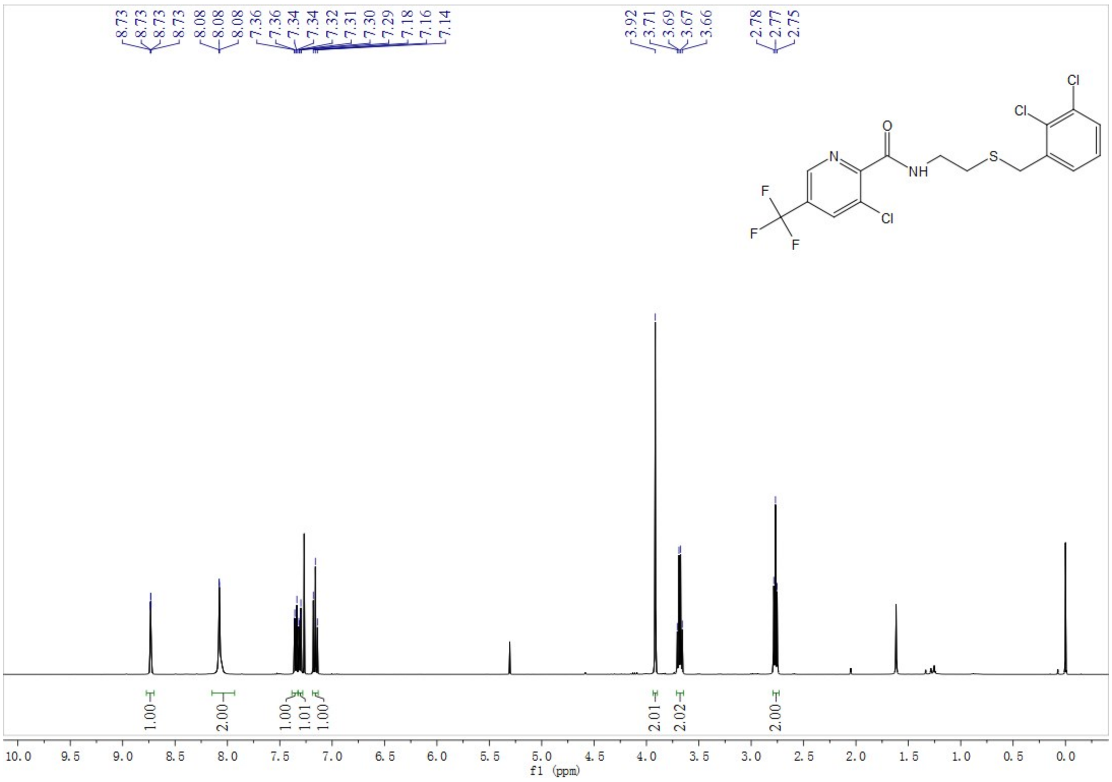

Fig. S16 ^{1}H NMR spectra of compound **E6**

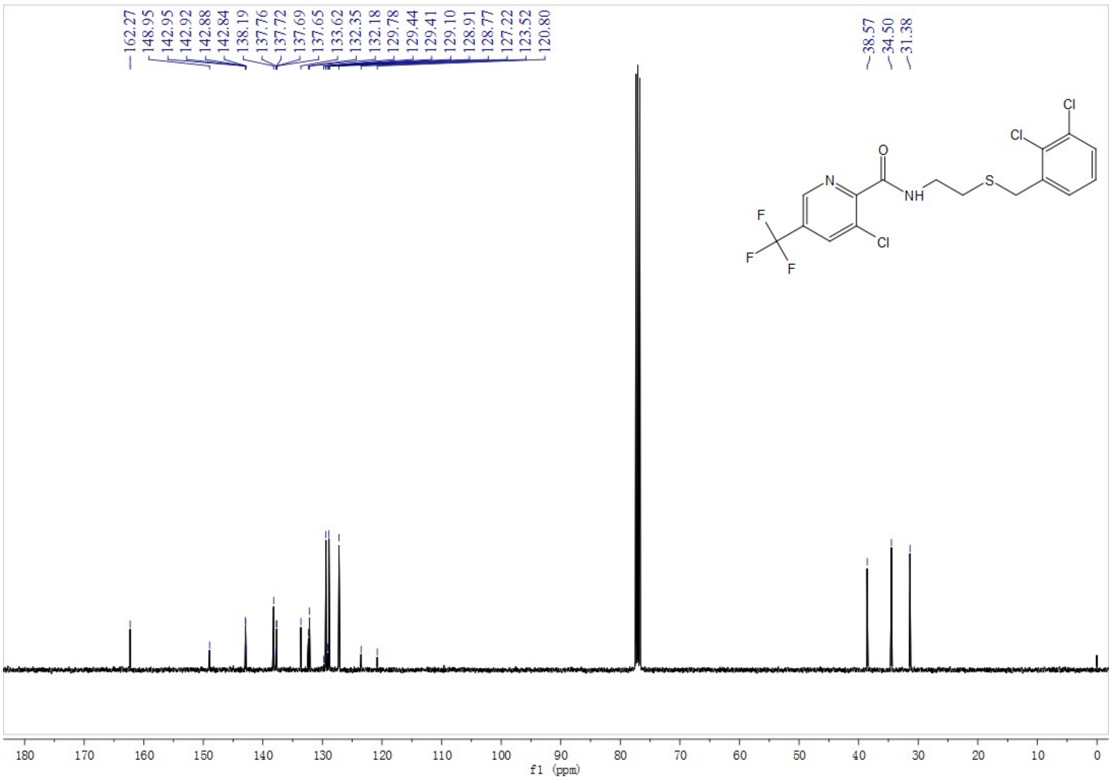

Fig. S17 ^{13}C NMR spectra of compound **E6**

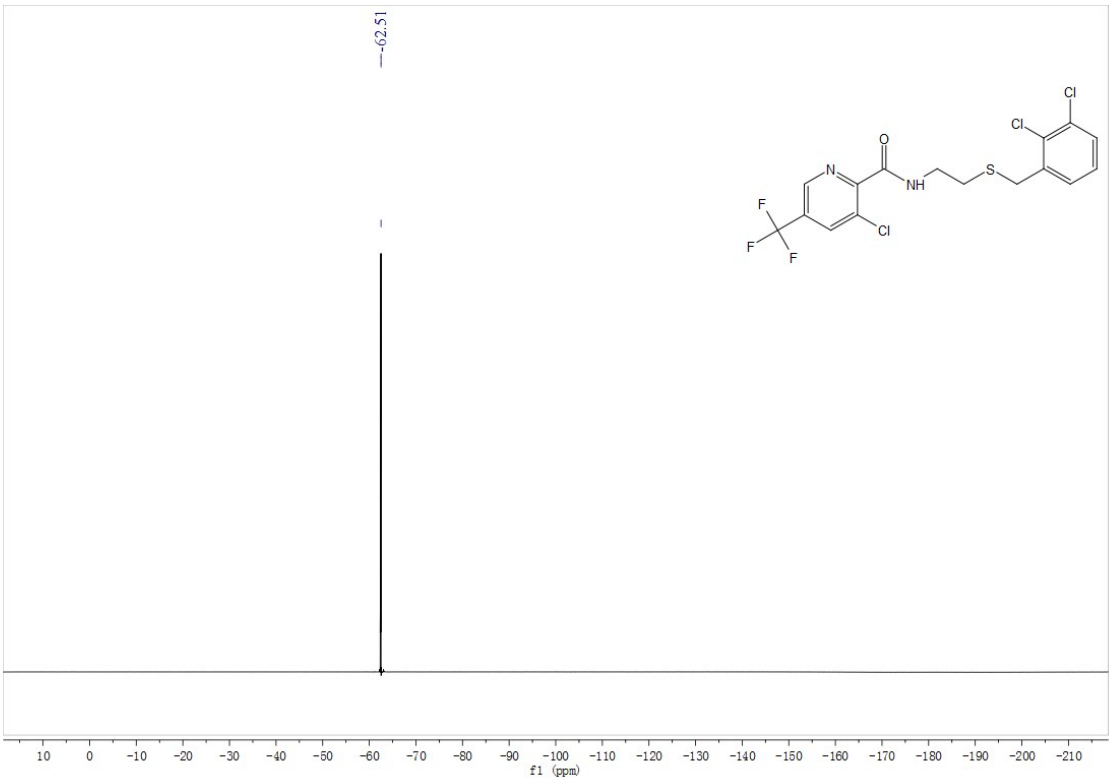

Fig. S18 ^{19}F NMR spectra of compound **E6**

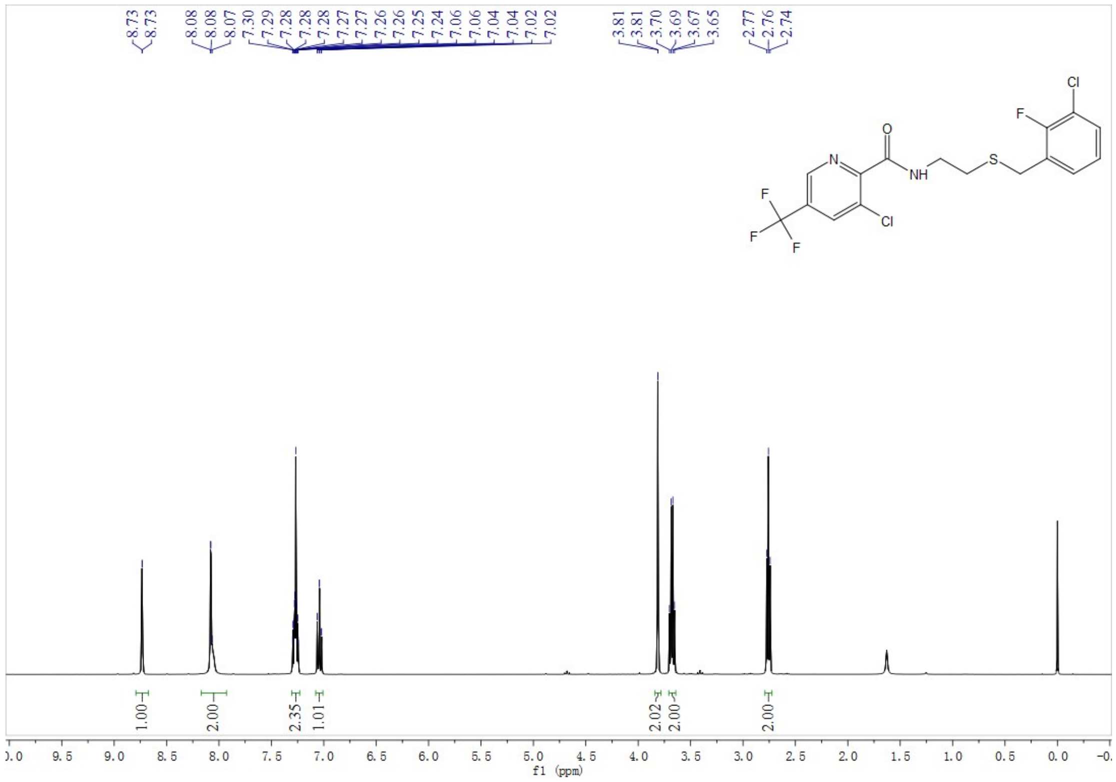

Fig. S19 ^{1}H NMR spectra of compound **E7**

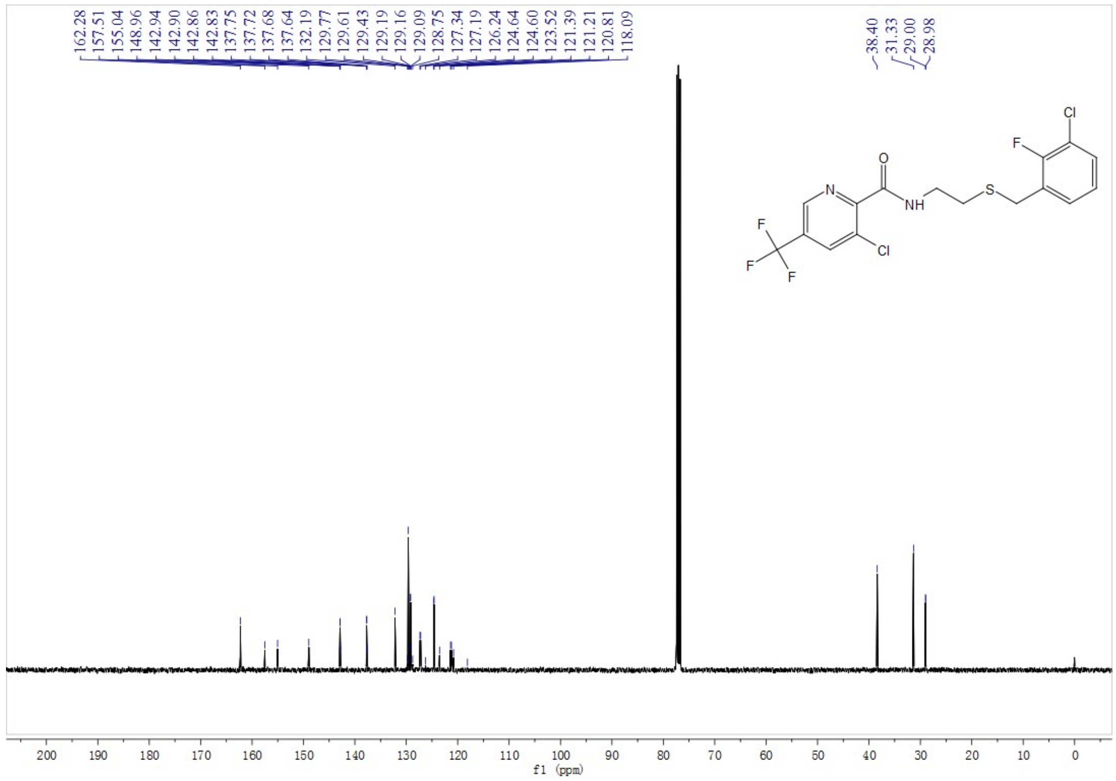

Fig. S20 ^{13}C NMR spectra of compound **E7**

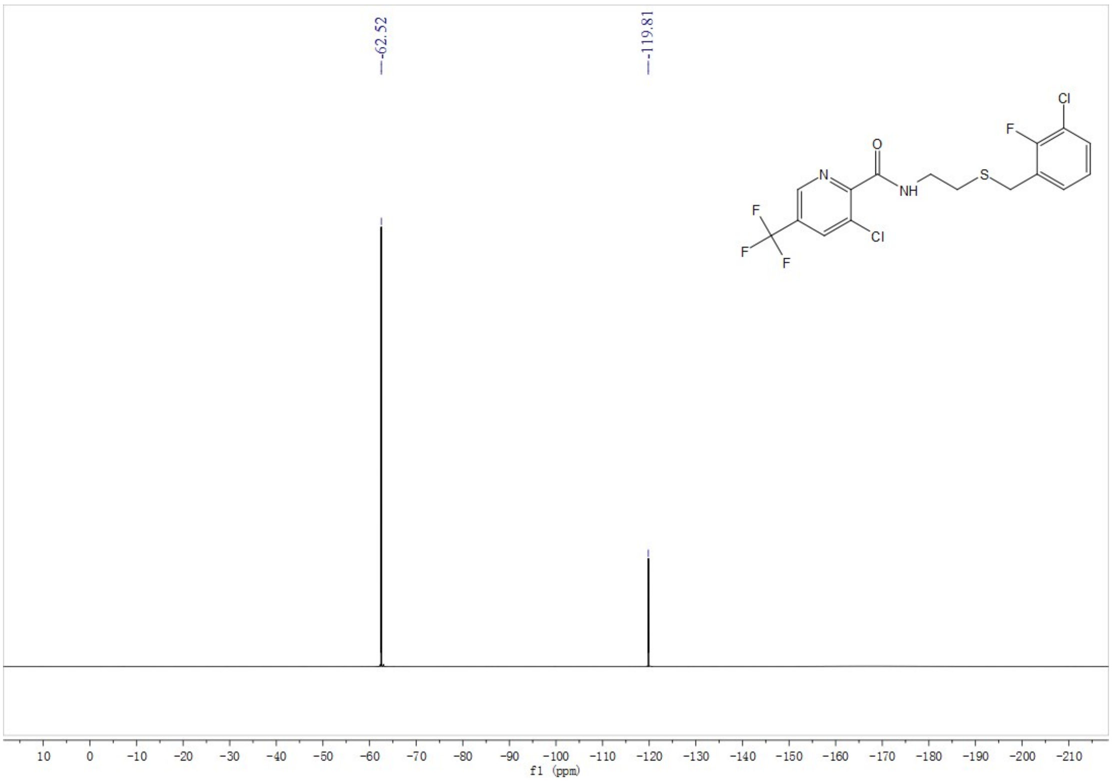

Fig. S21 ^{19}F NMR spectra of compound **E7**

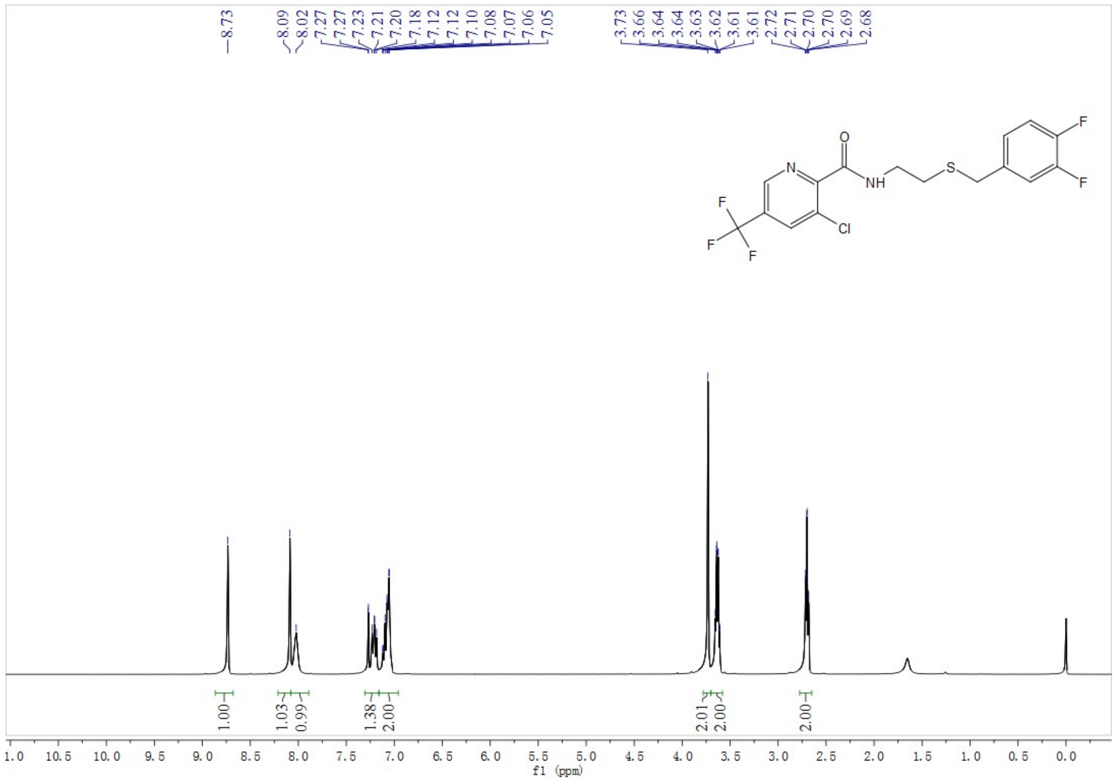

Fig. S22 ^{1}H NMR spectra of compound **E8**

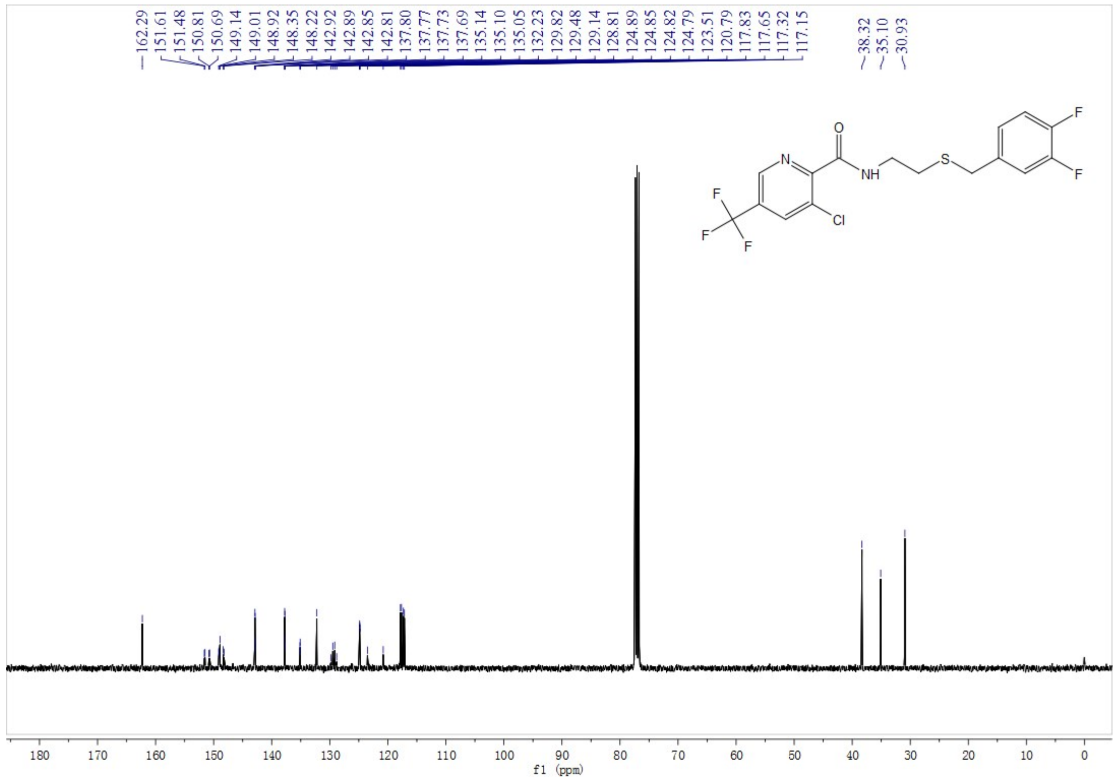

Fig. S23 ^{13}C NMR spectra of compound **E8**

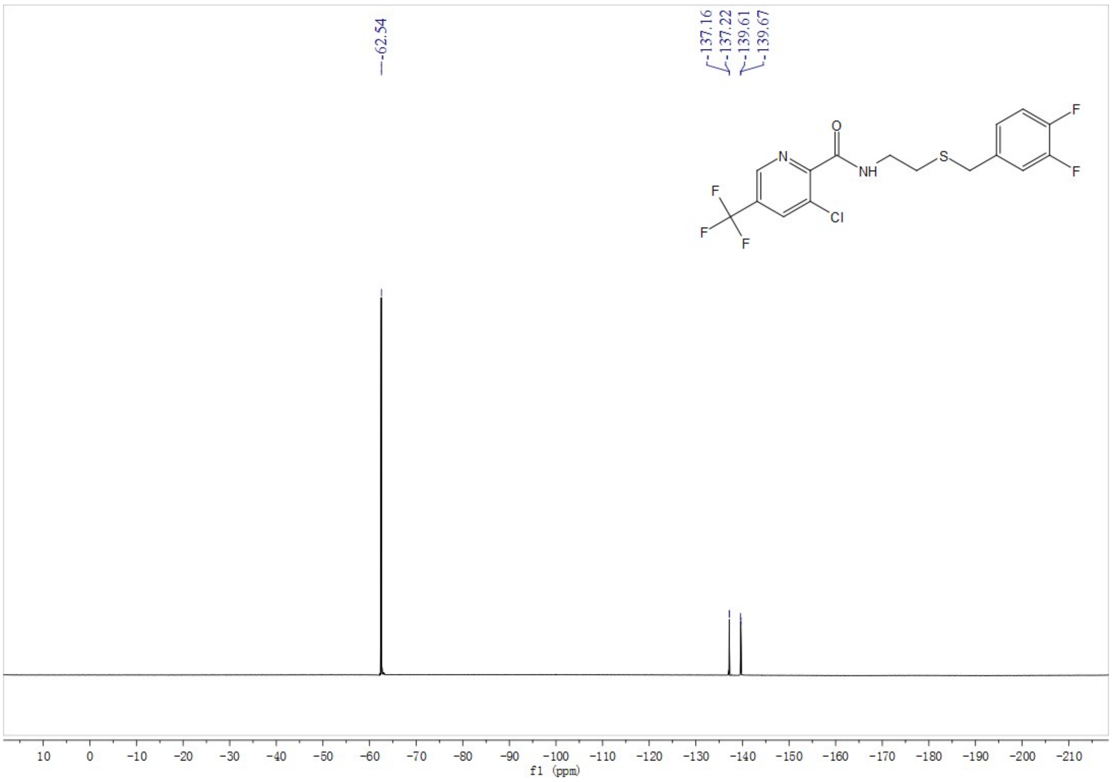

Fig. S24 ^{19}F NMR spectra of compound **E8**

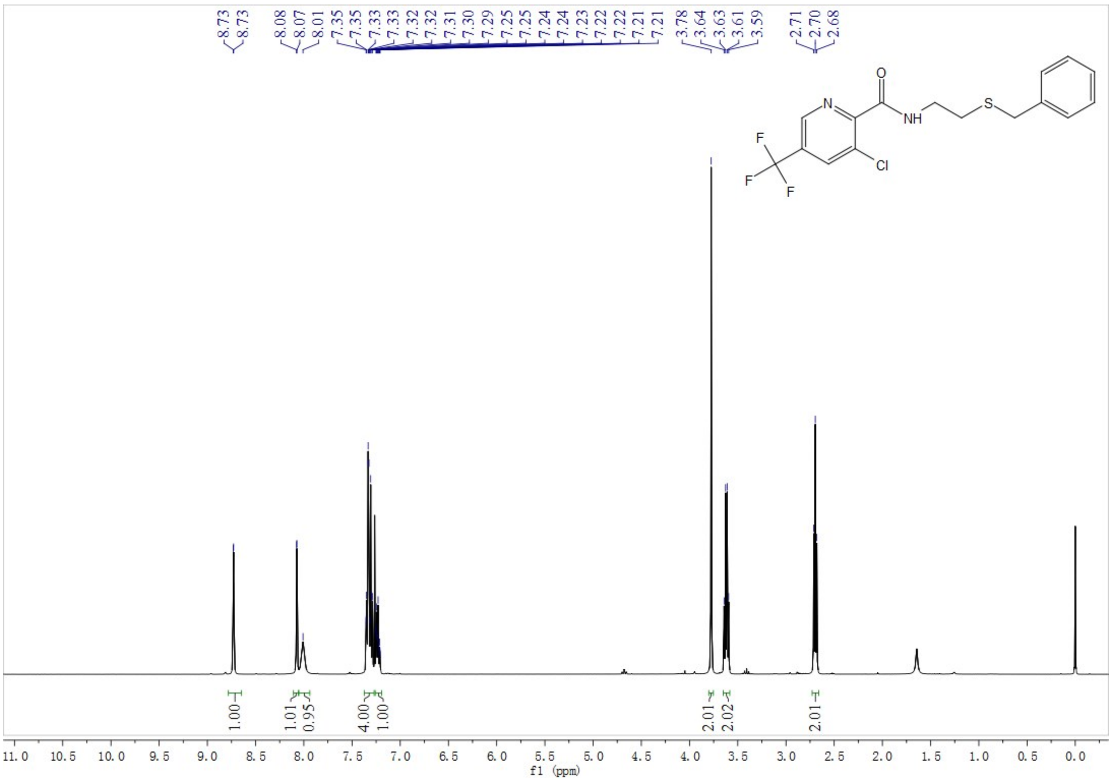

Fig. S25 ^{1}H NMR spectra of compound **E9**

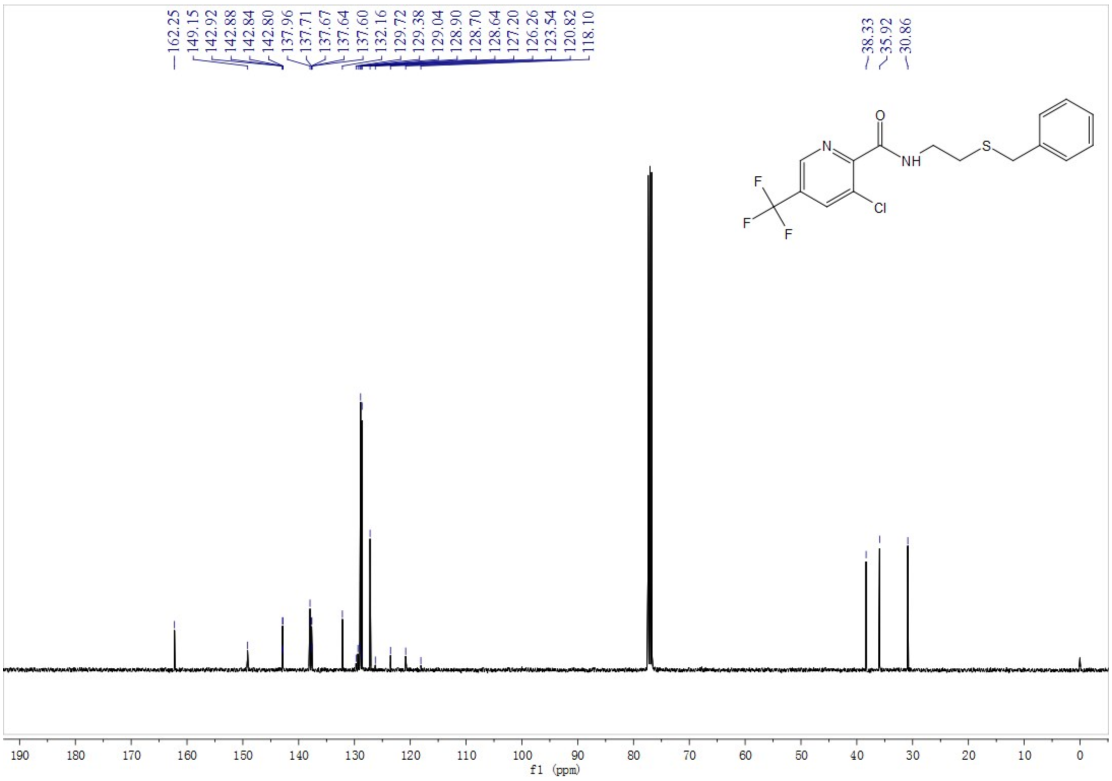

Fig. S26 ^{13}C NMR spectra of compound **E9**

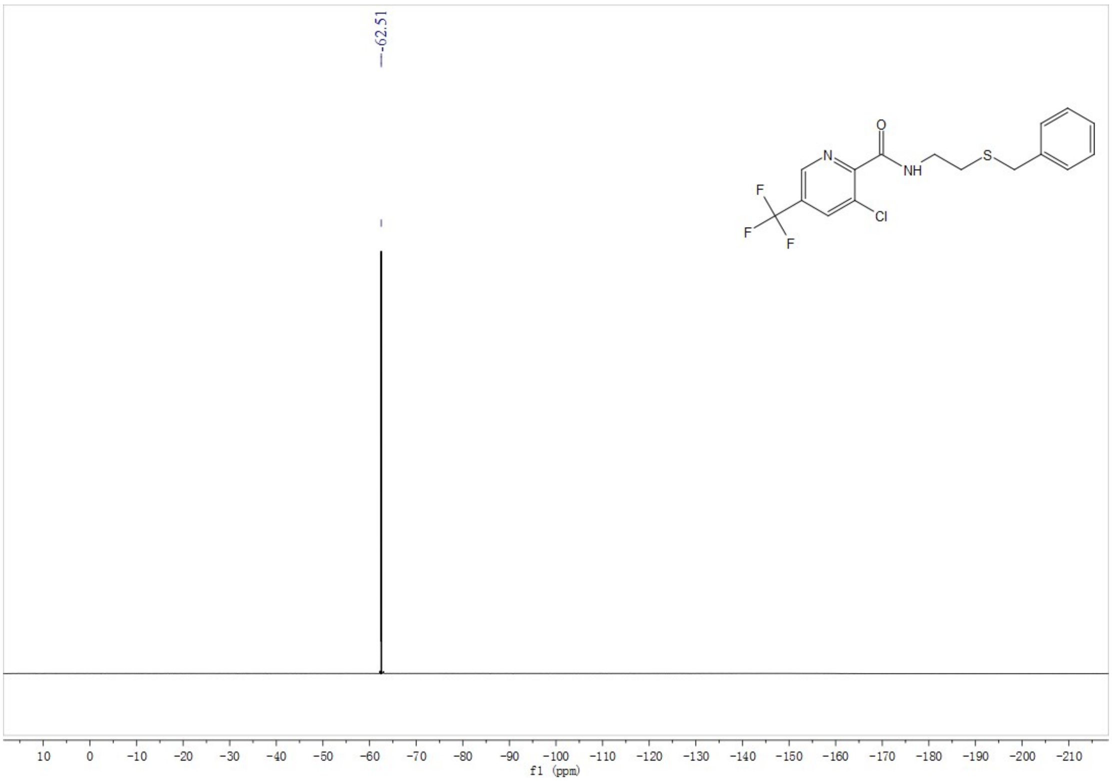

Fig. S27 ^{19}F NMR spectra of compound **E9**

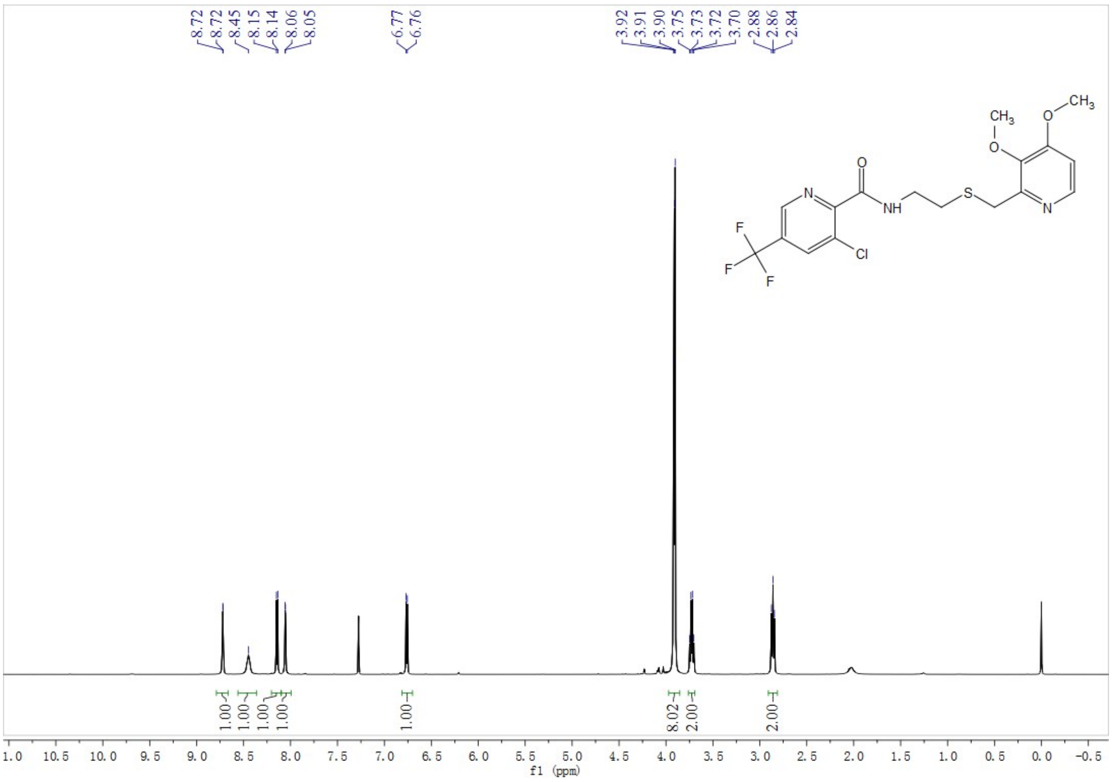

Fig. S28 ^{1}H NMR spectra of compound **E10**

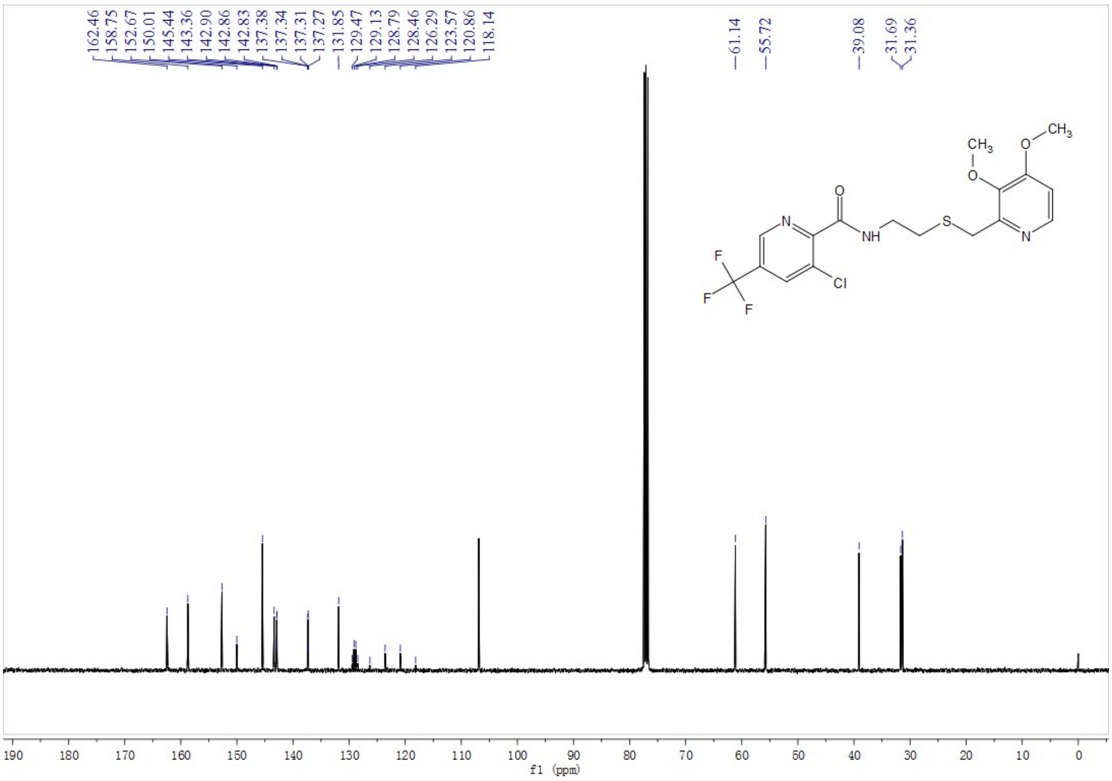

Fig. S29 ^{13}C NMR spectra of compound **E10**

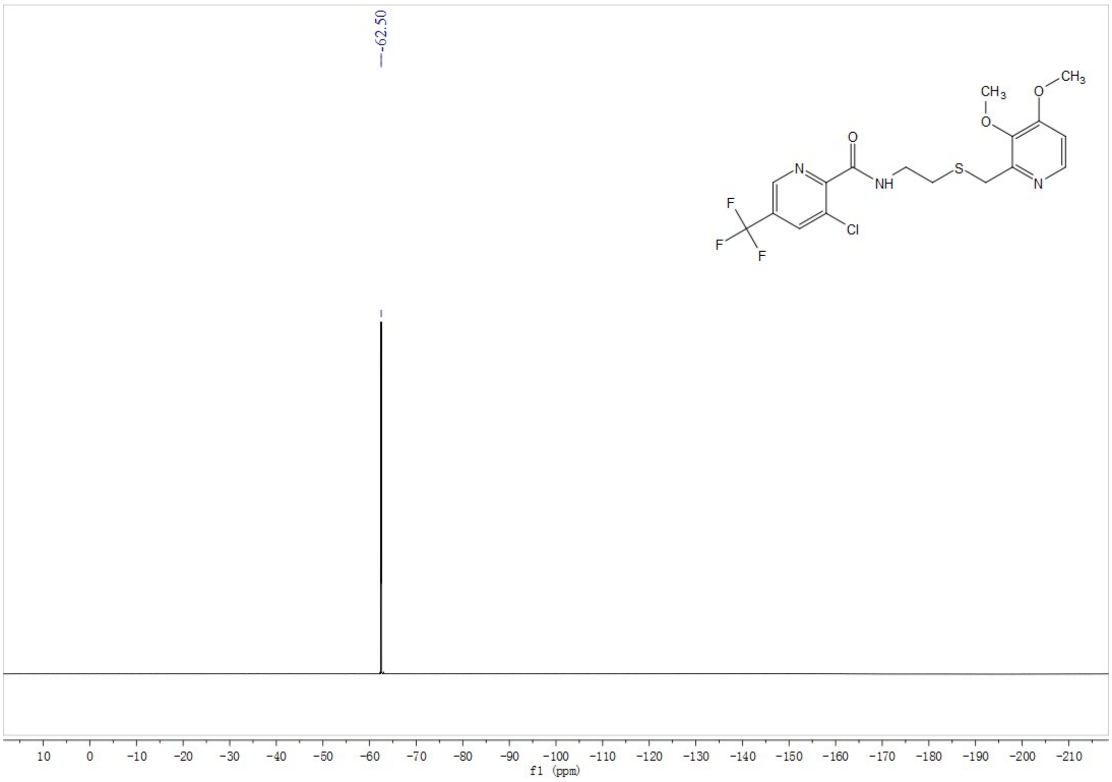

Fig. S30 ^{19}F NMR spectra of compound **E10**

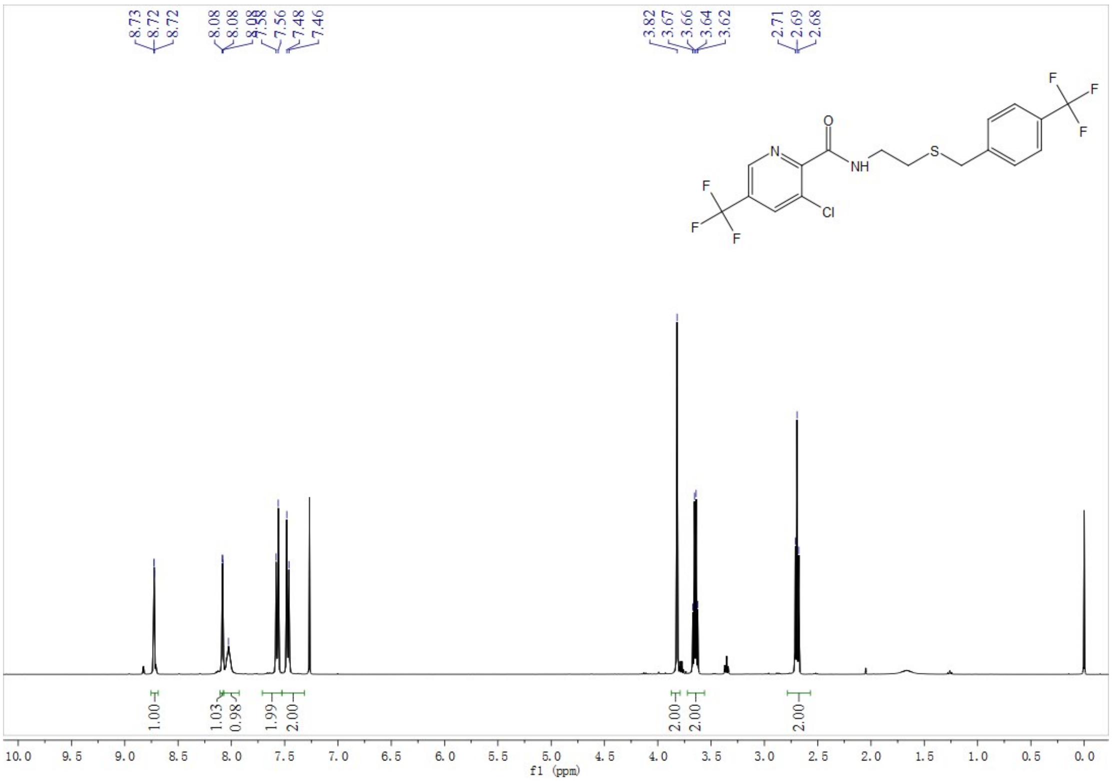

Fig. S31 ^{1}H NMR spectra of compound **E11**

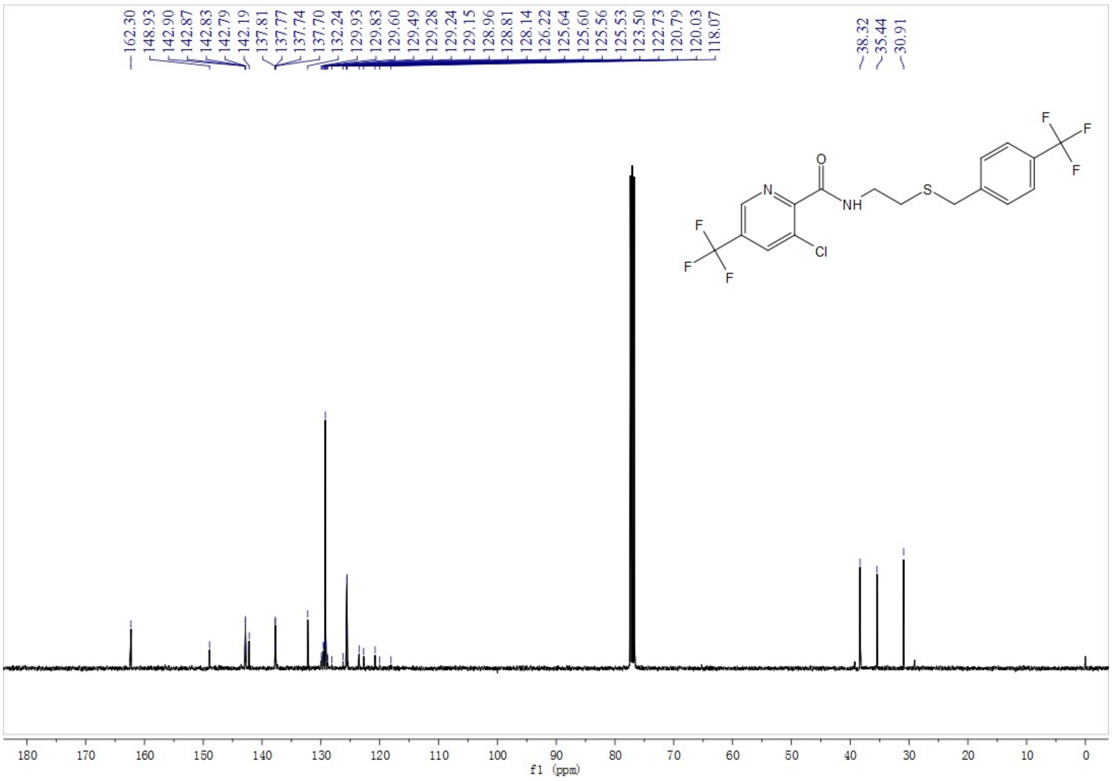

Fig. S32 ^{13}C NMR spectra of compound **E11**

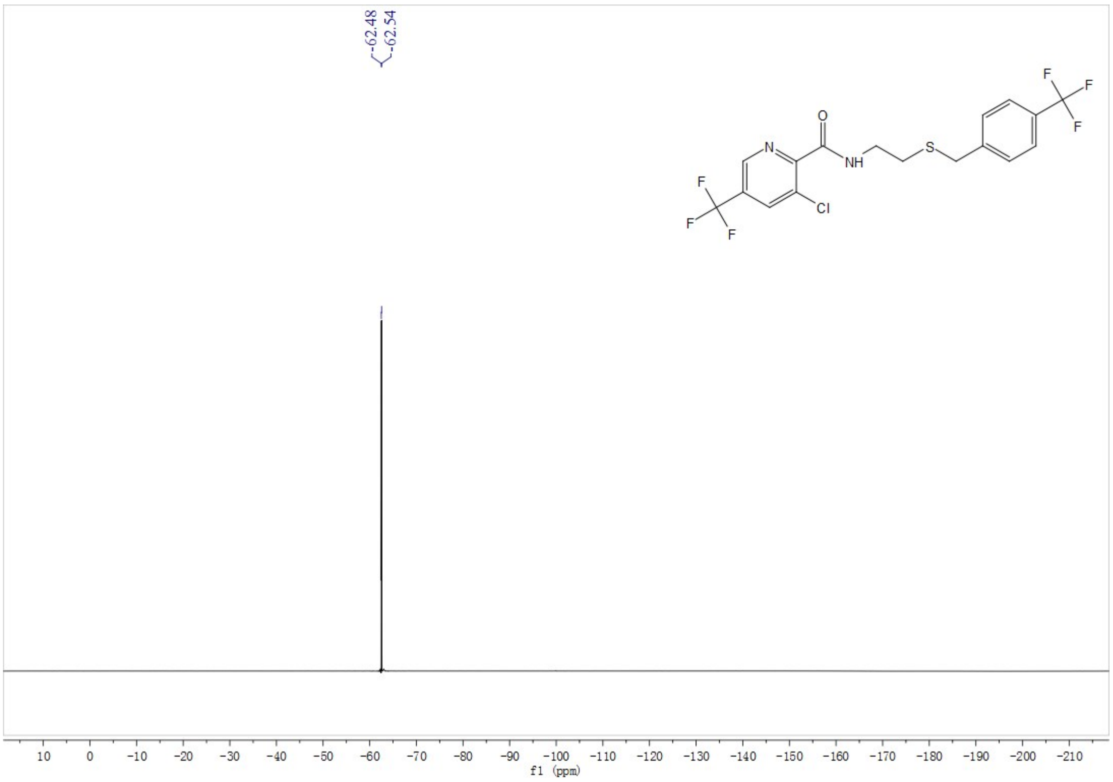

Fig. S33 ^{19}F NMR spectra of compound **E11**

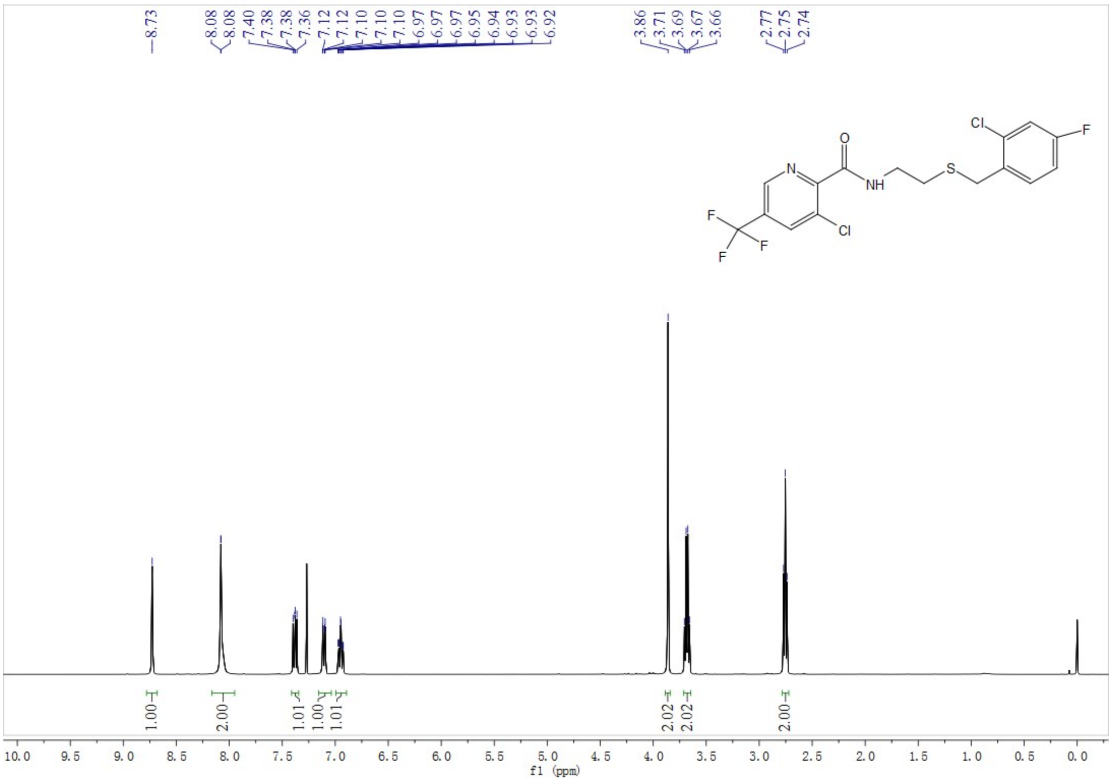

Fig. S34 ^{1}H NMR spectra of compound **E12**

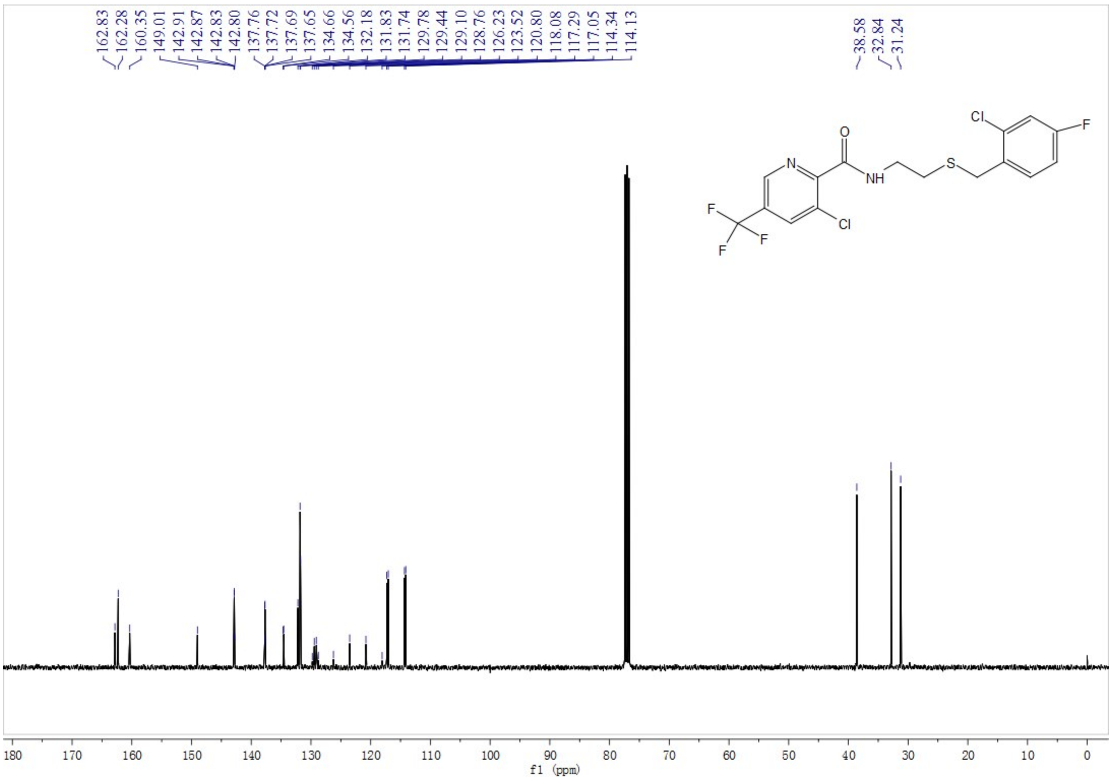

Fig. S35 ^{13}C NMR spectra of compound **E12**

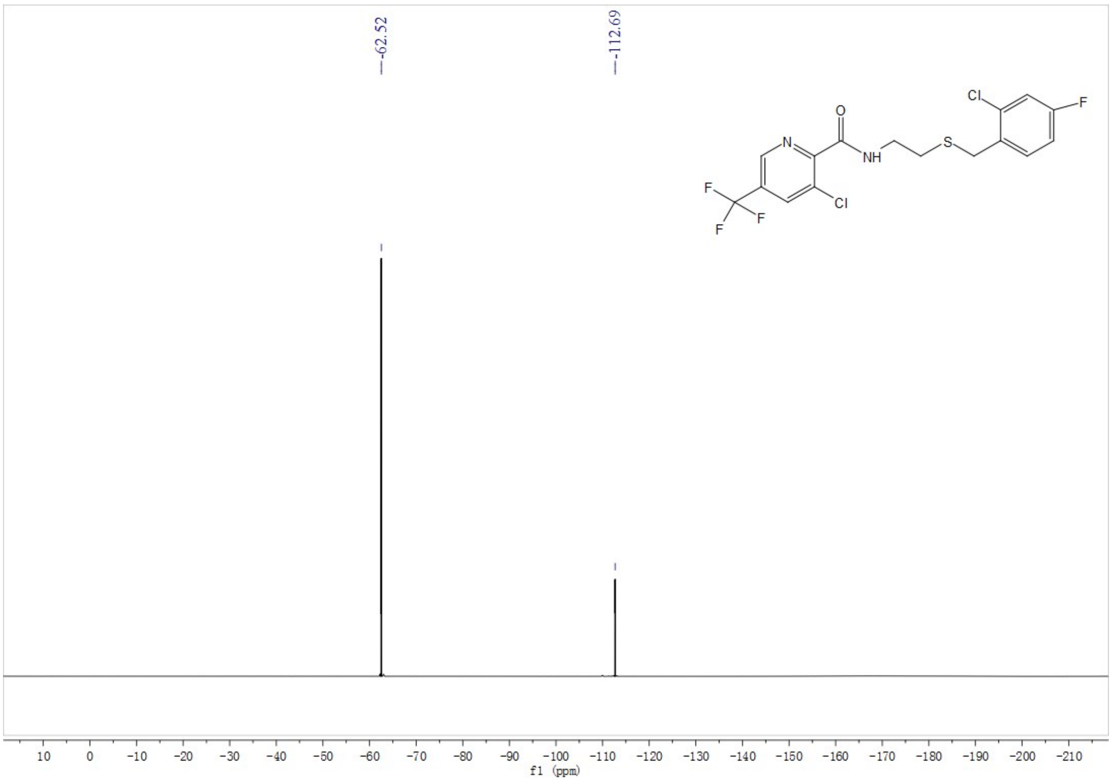

Fig. S36 ^{19}F NMR spectra of compound **E12**

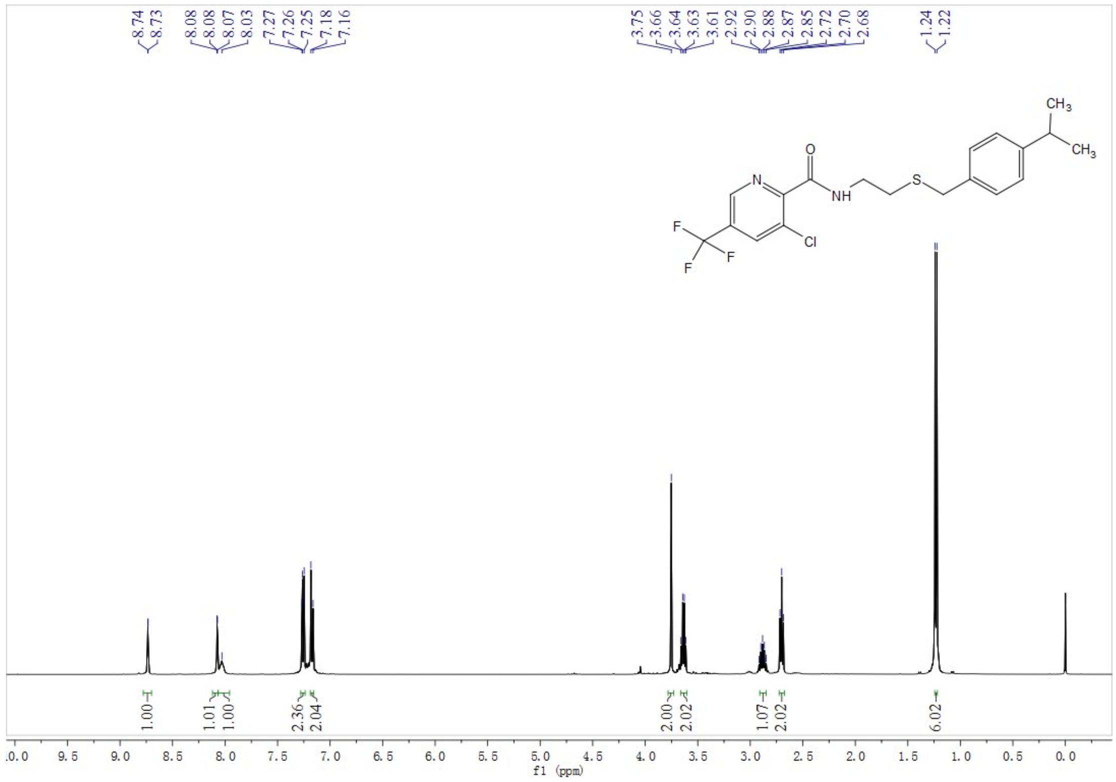

Fig. S37 ^{1}H NMR spectra of compound **E13**

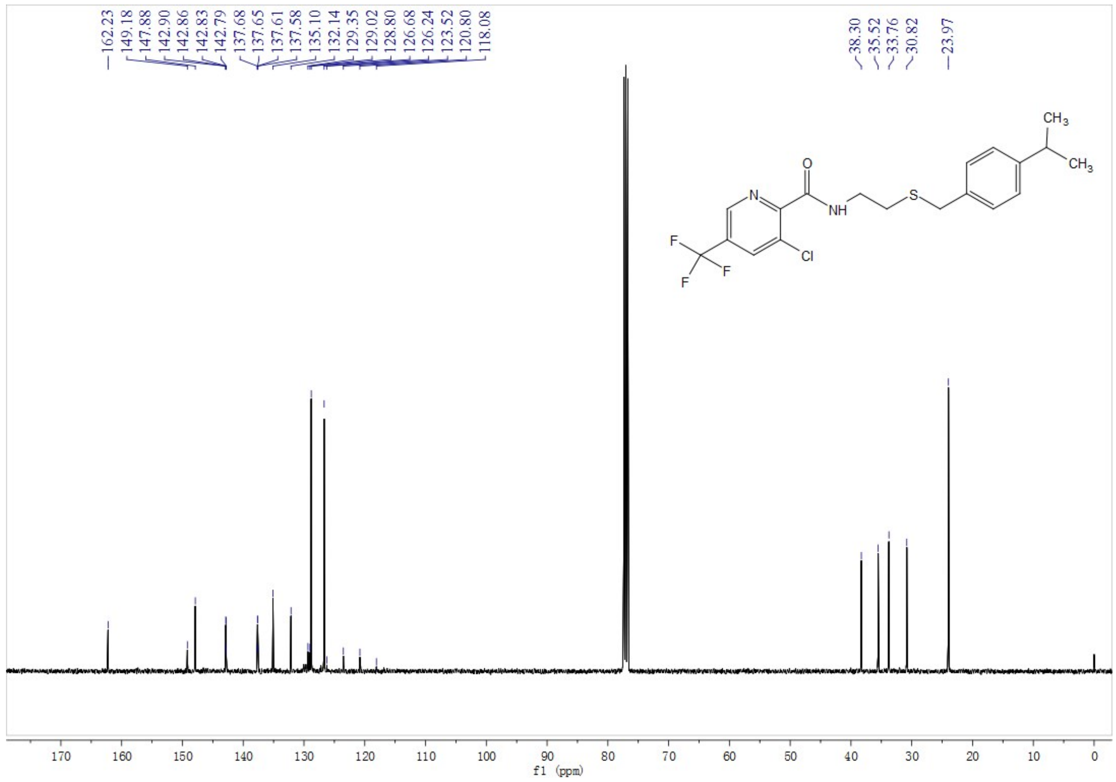

Fig. S38 ^{13}C NMR spectra of compound **E13**

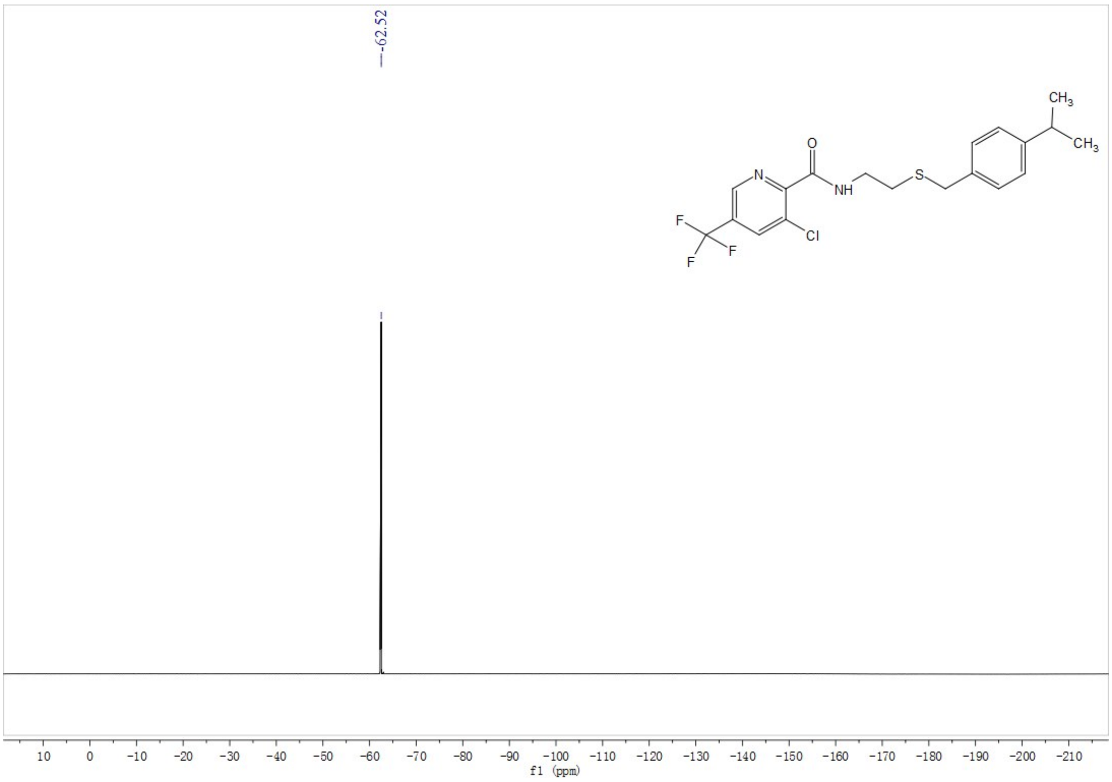

Fig. S39 ^{19}F NMR spectra of compound **E13**

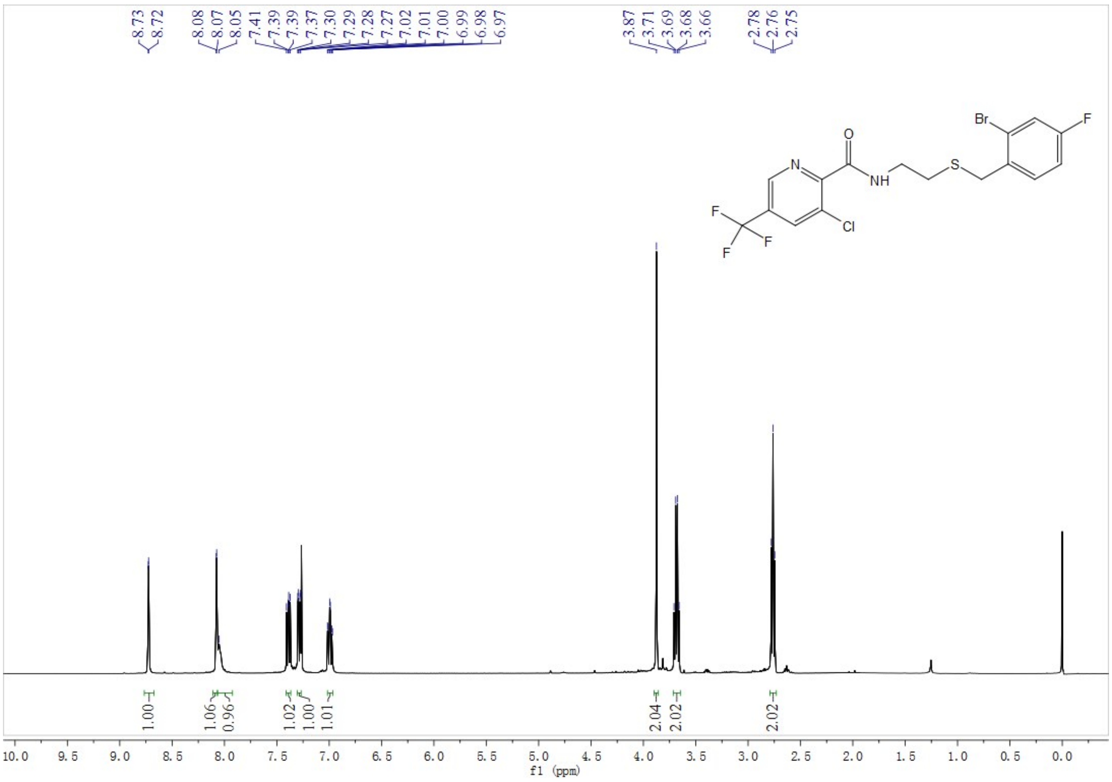

Fig. S40 ^{1}H NMR spectra of compound **E14**

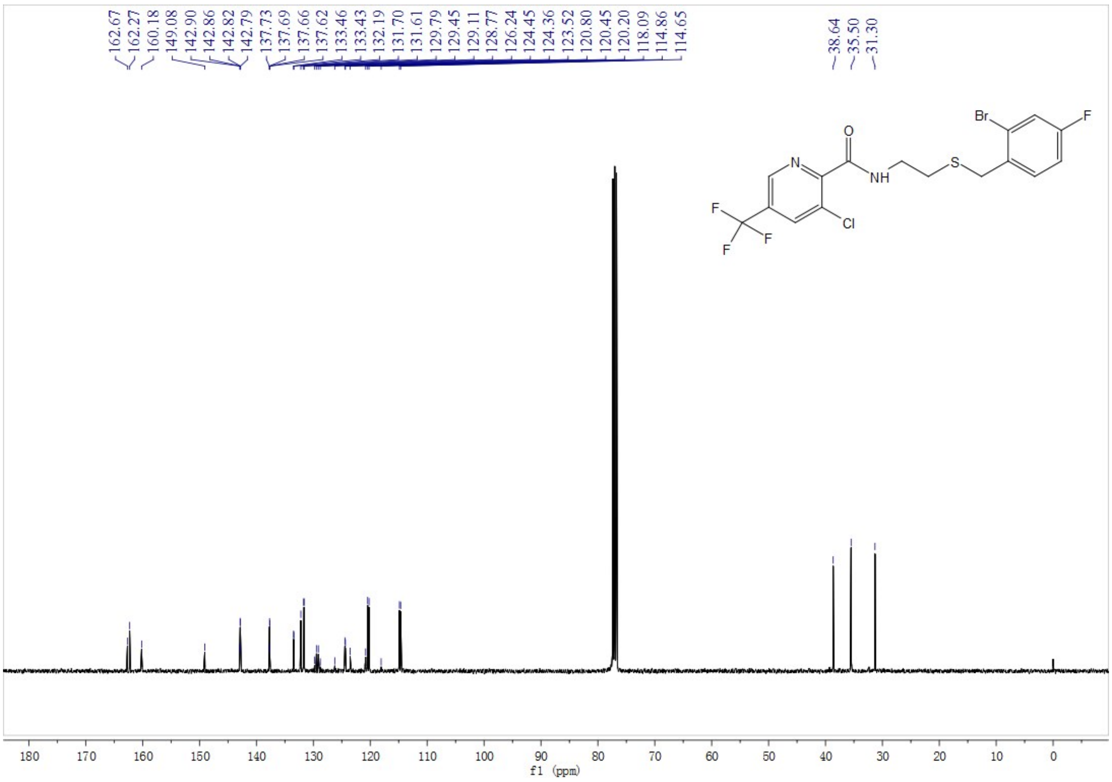

Fig. S41 ^{13}C NMR spectra of compound **E14**

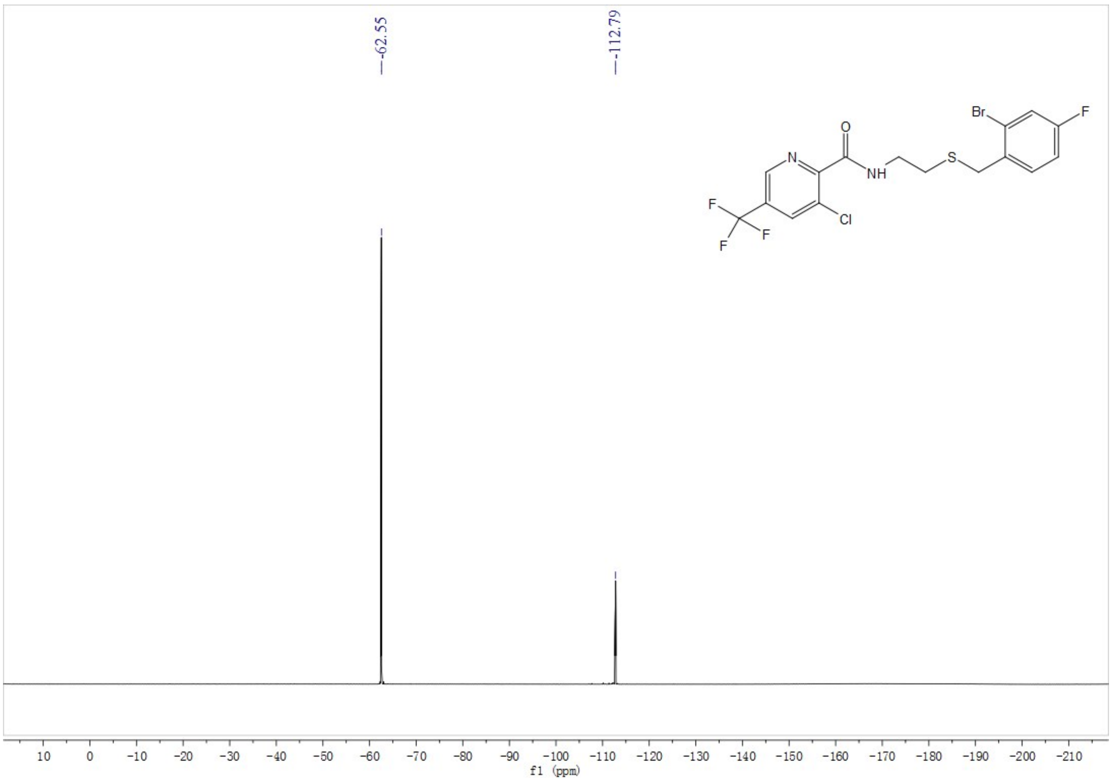

Fig. S42 ^{19}F NMR spectra of compound **E14**

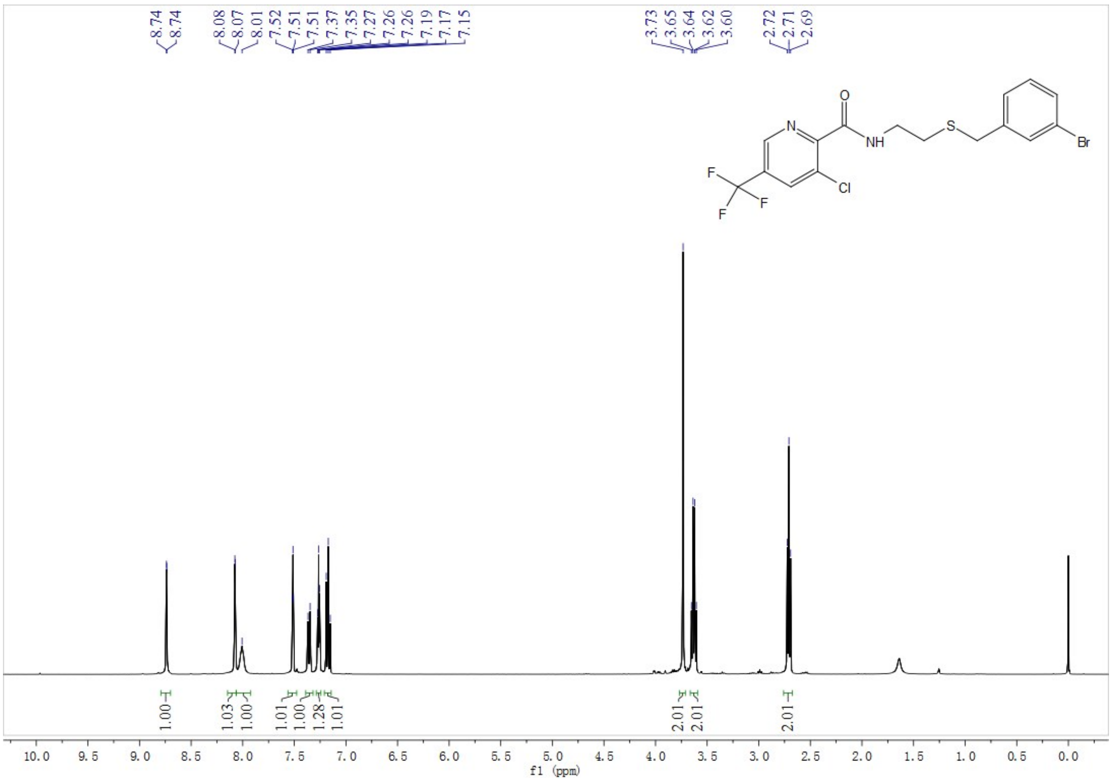

Fig. S43 ^{1}H NMR spectra of compound **E15**

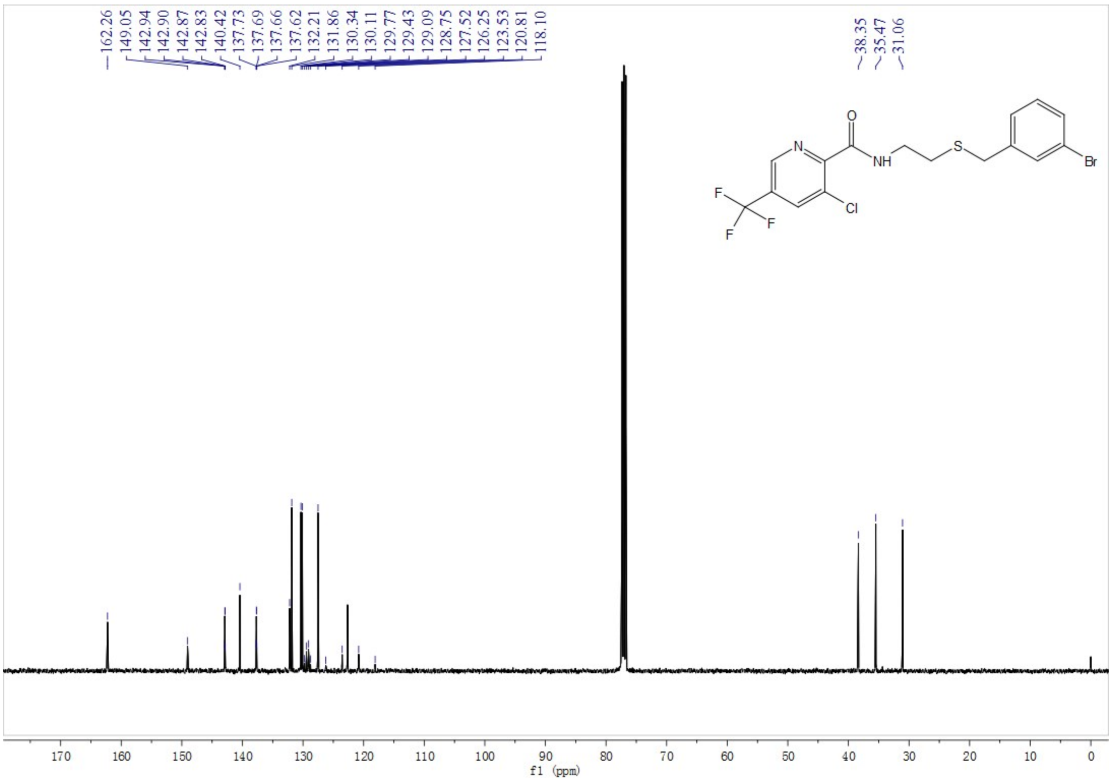

Fig. S44 ^{13}C NMR spectra of compound **E15**

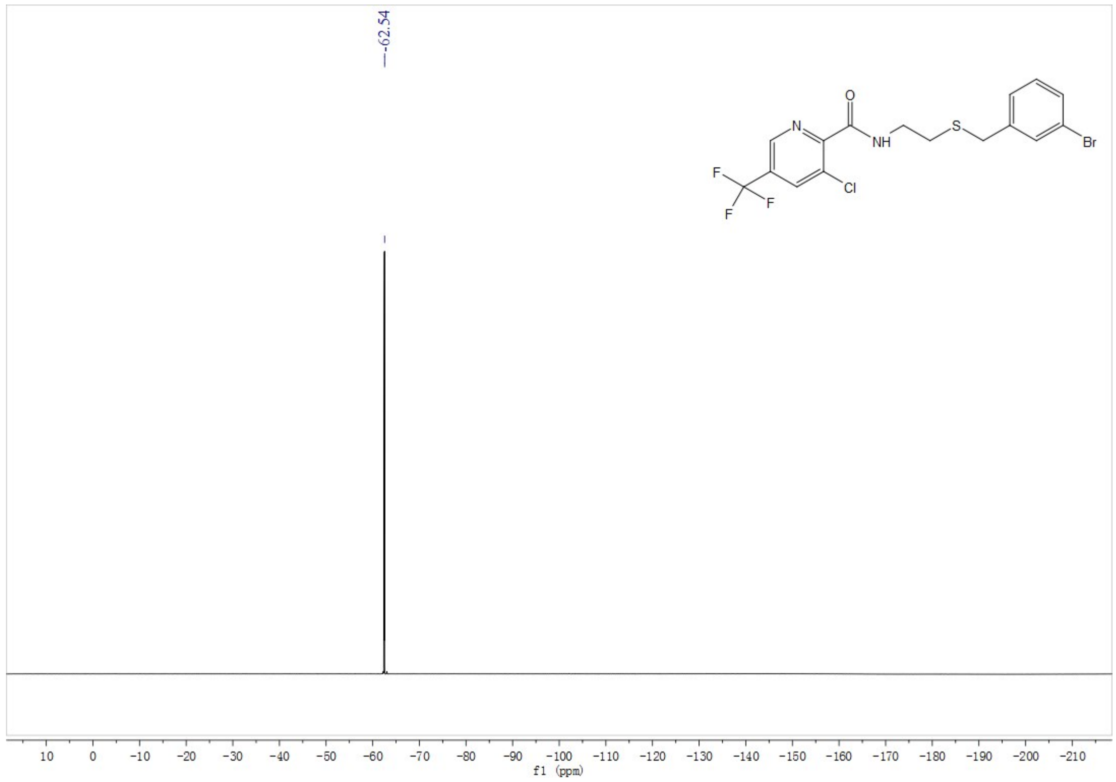

Fig. S45 ^{19}F NMR spectra of compound **E15**

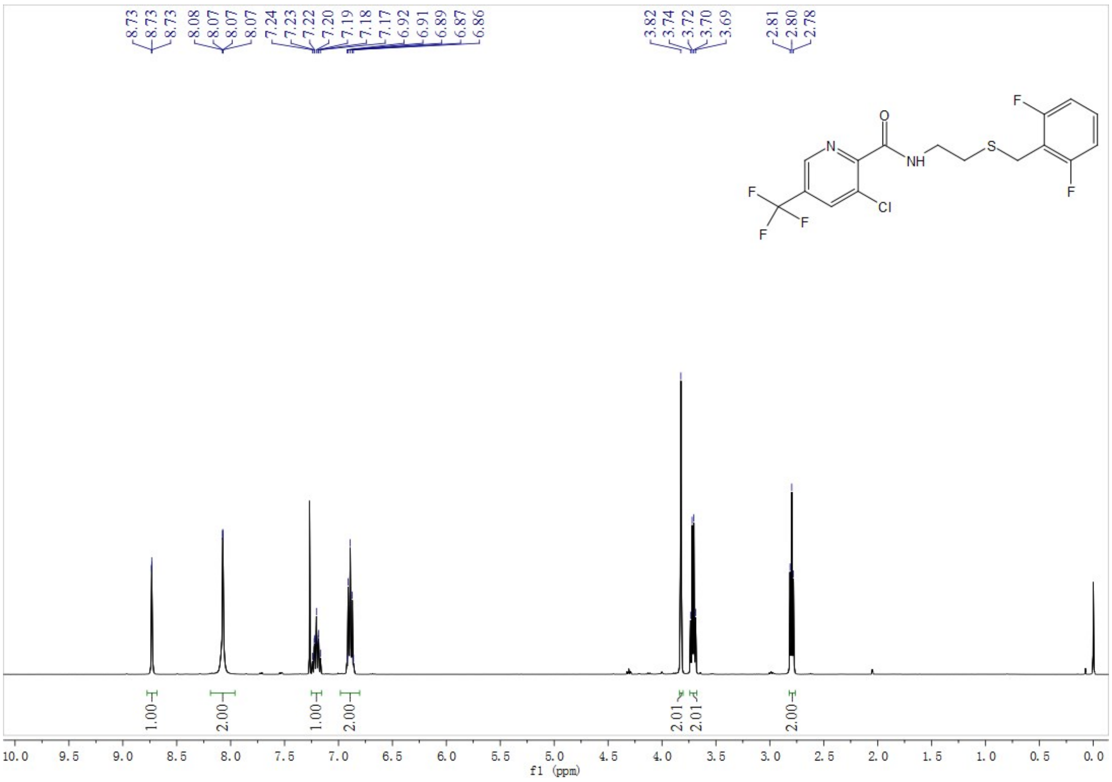

Fig. S46^{1}H NMR spectra of compound **E16**

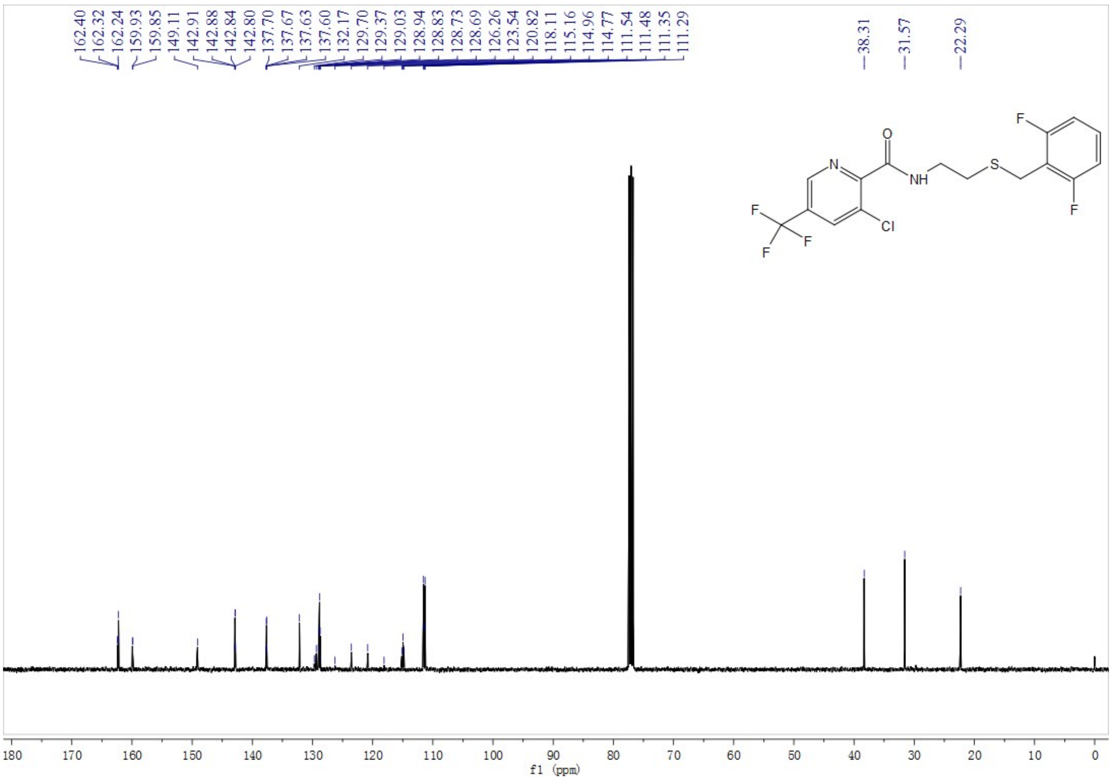

Fig. S47 ^{13}C NMR spectra of compound **E16**

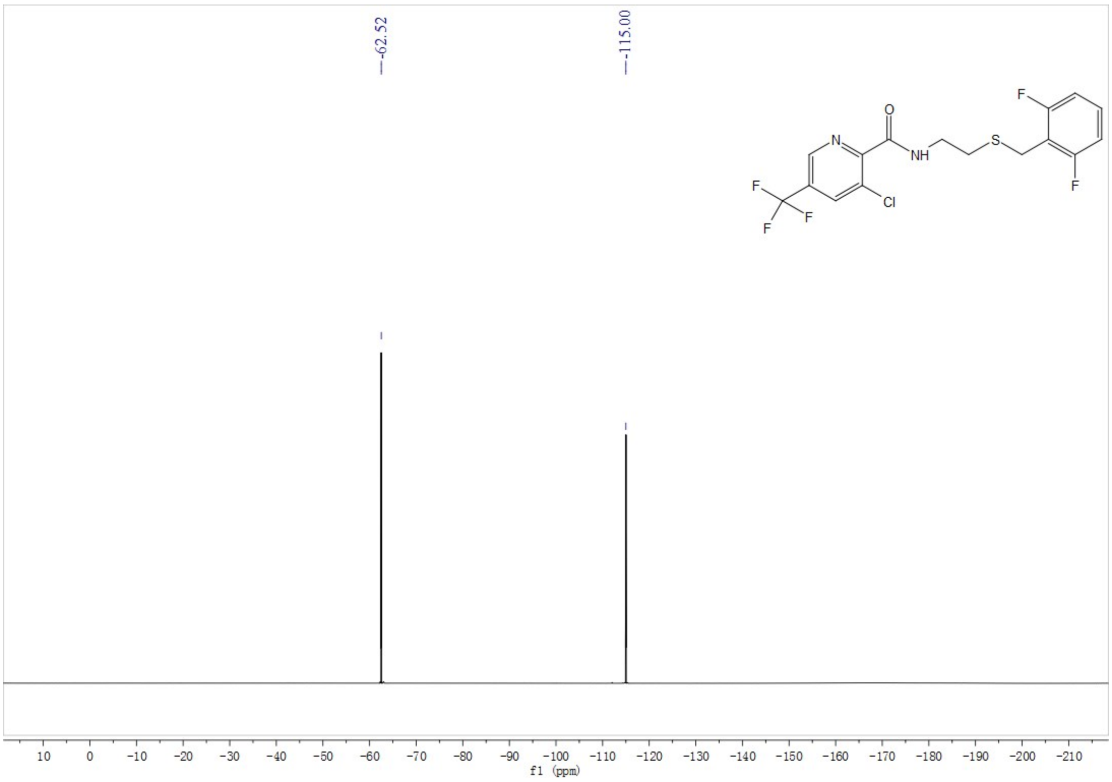

Fig. S48 ^{19}F NMR spectra of compound **E16**

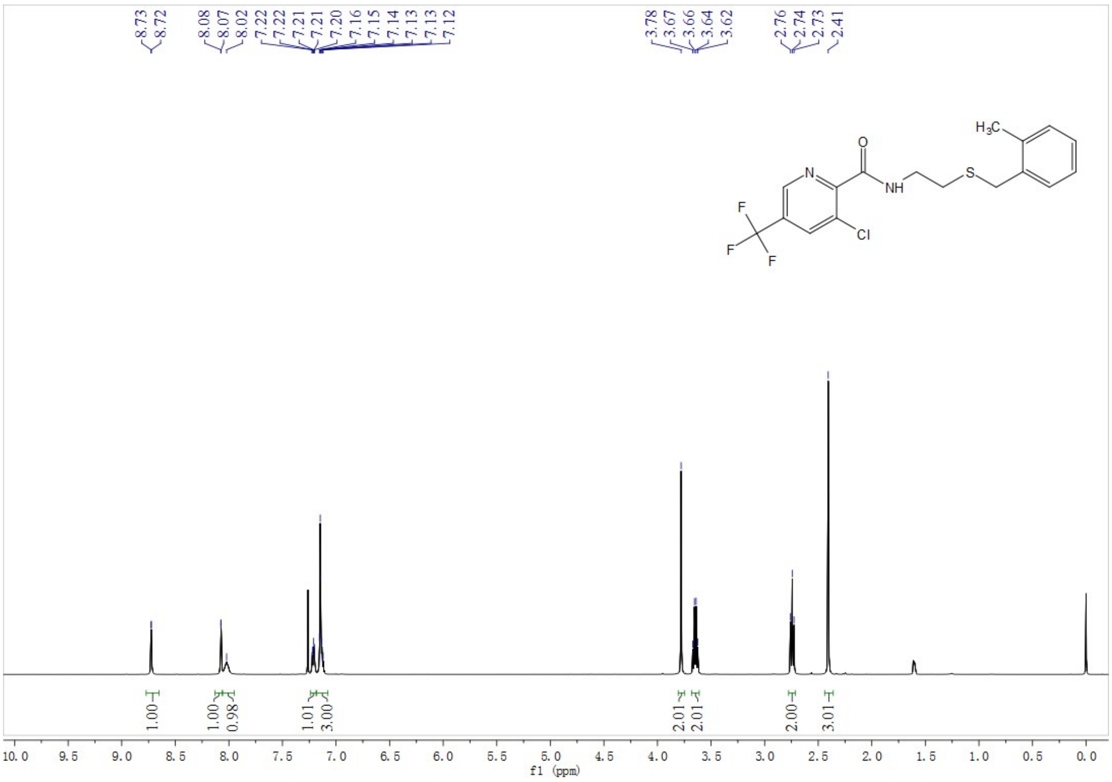

Fig. S49 ^{1}H NMR spectra of compound **E17**

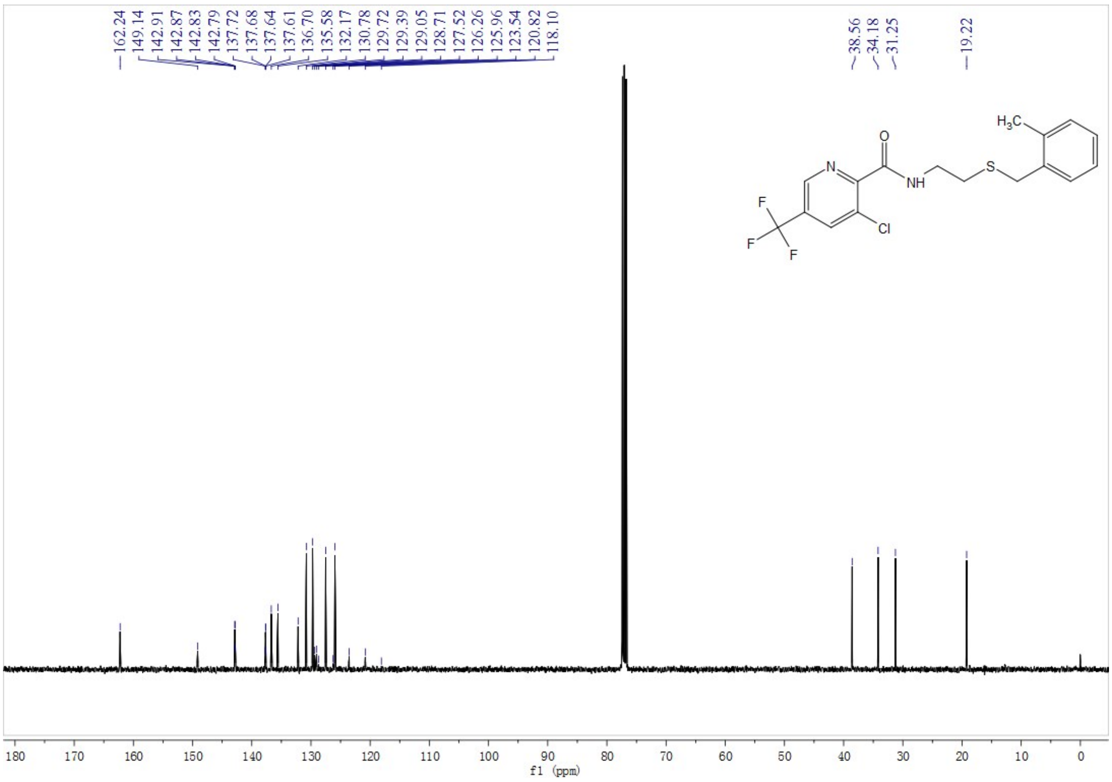

Fig. S50 ^{13}C NMR spectra of compound **E17**

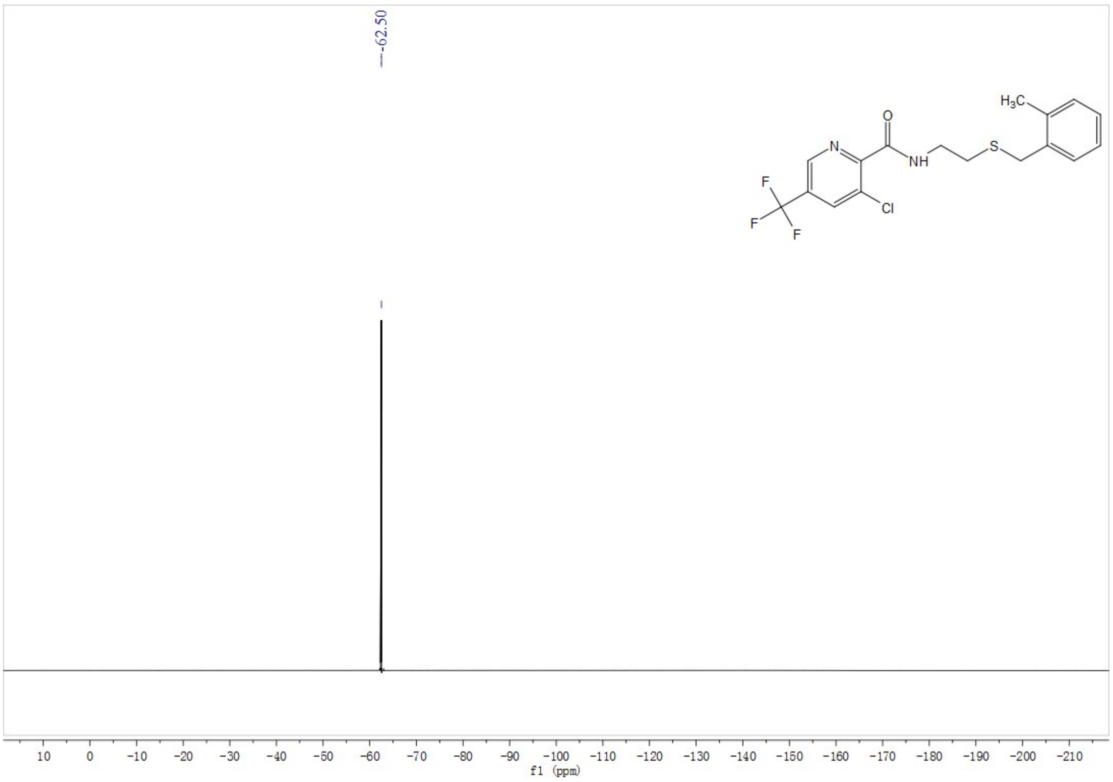

Fig. S51 ^{19}F NMR spectra of compound **E17**

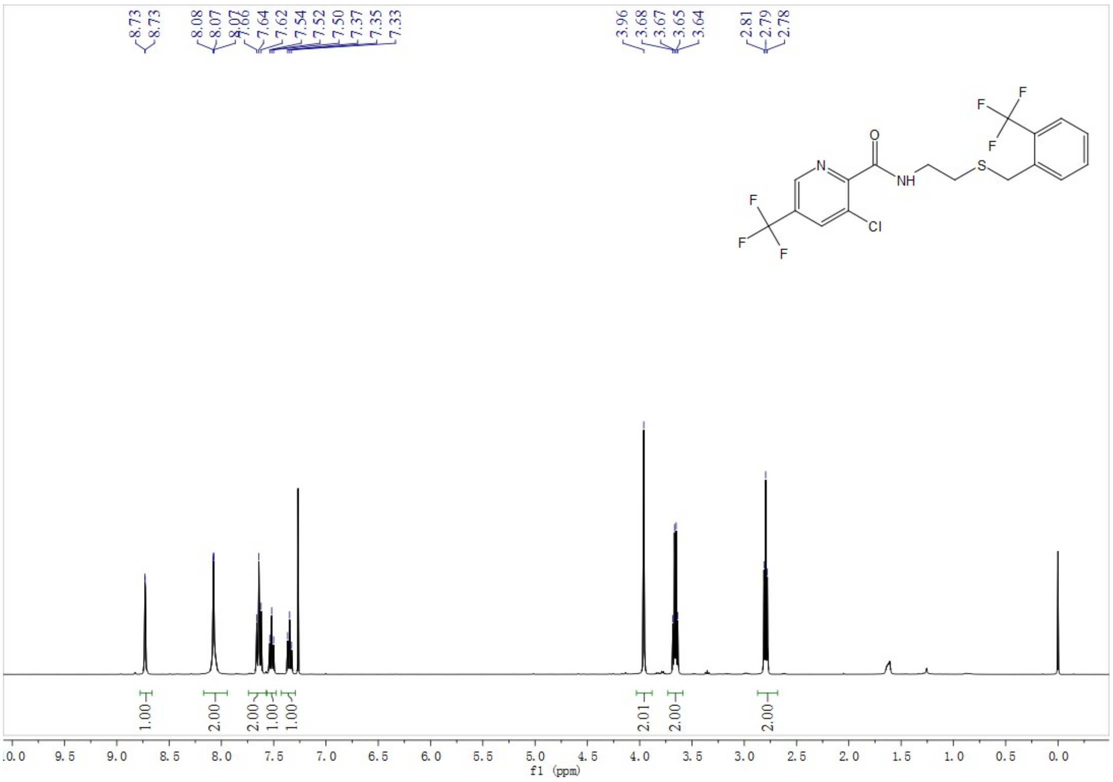

Fig. S52 ^{1}H NMR spectra of compound **E18**

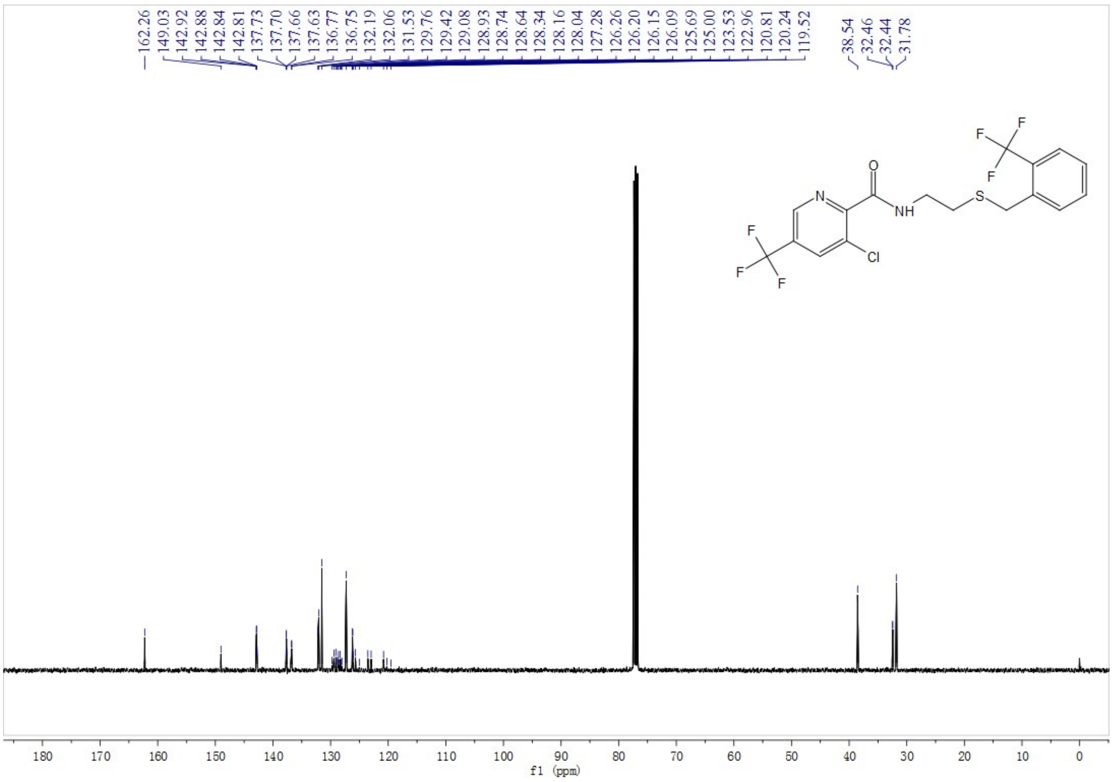

Fig. S53 ^{13}C NMR spectra of compound **E18**

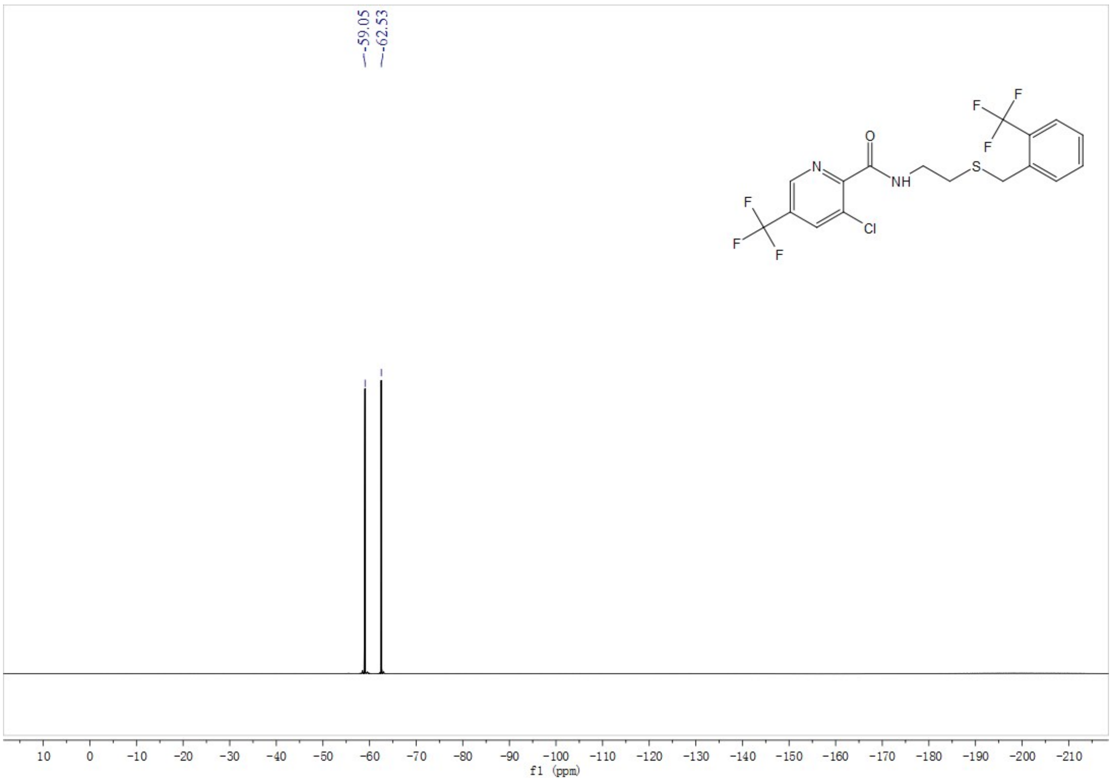

Fig. S54 ^{19}F NMR spectra of compound **E18**

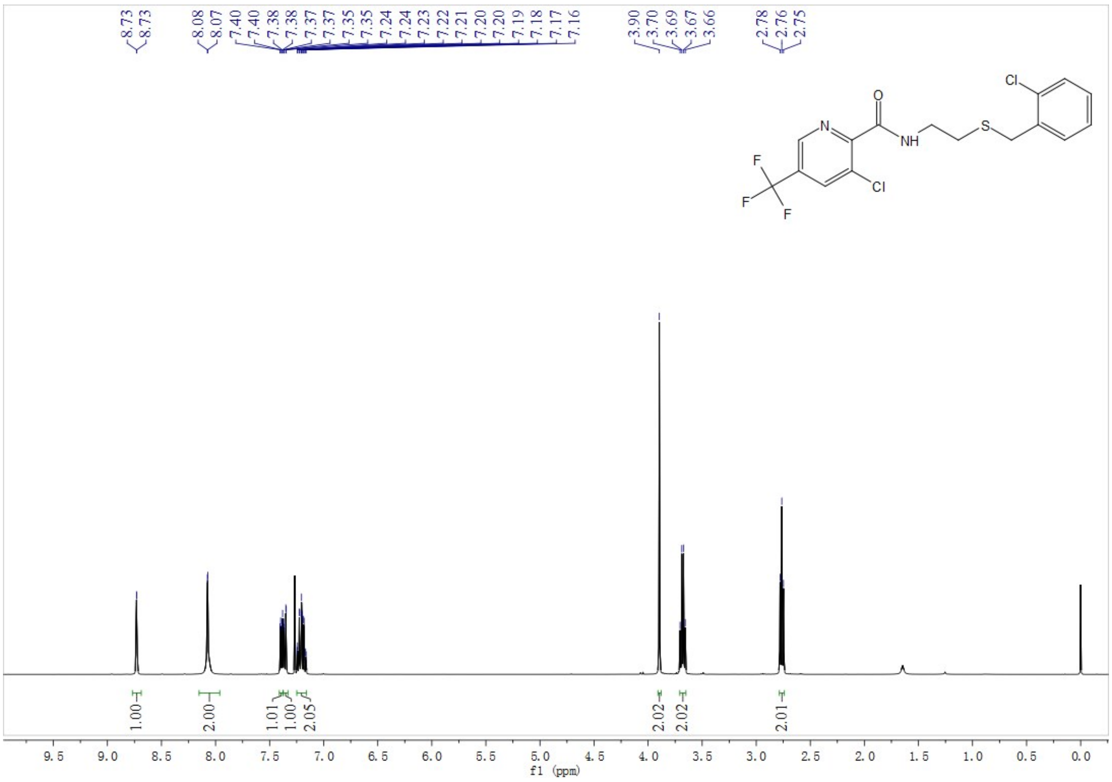

Fig. S55 ^{1}H NMR spectra of compound **E19**

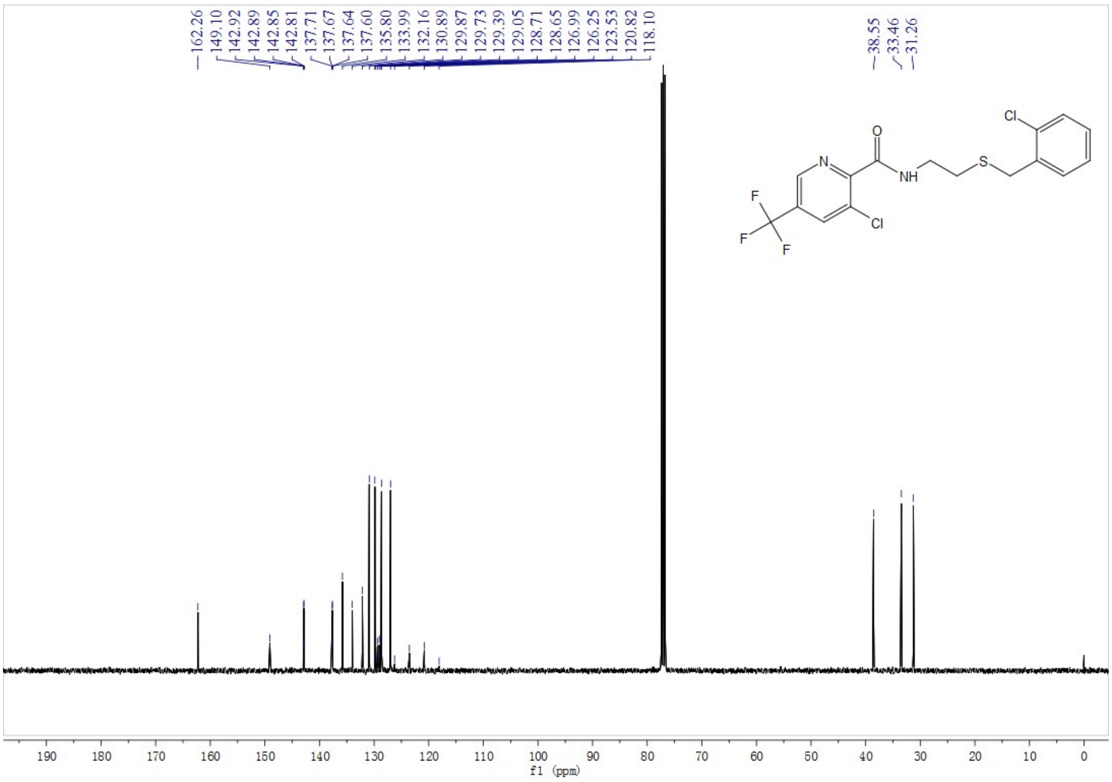

Fig. S56 ^{13}C NMR spectra of compound **E19**

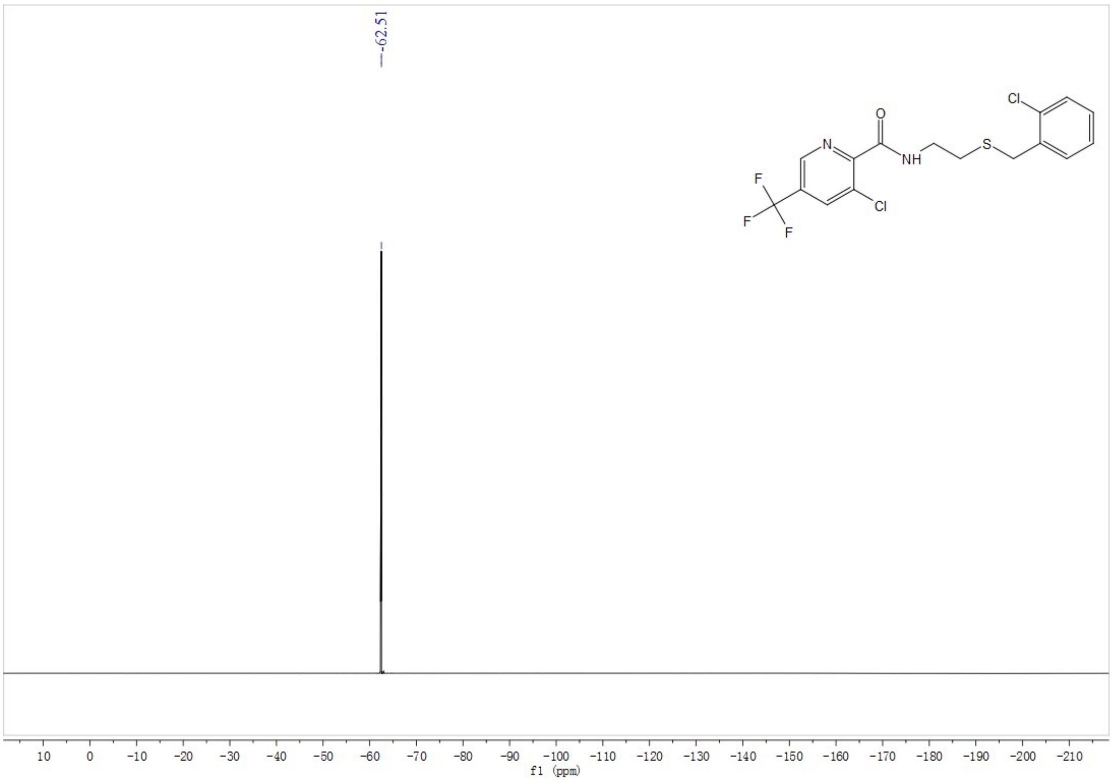

Fig. S57 ^{19}F NMR spectra of compound **E19**

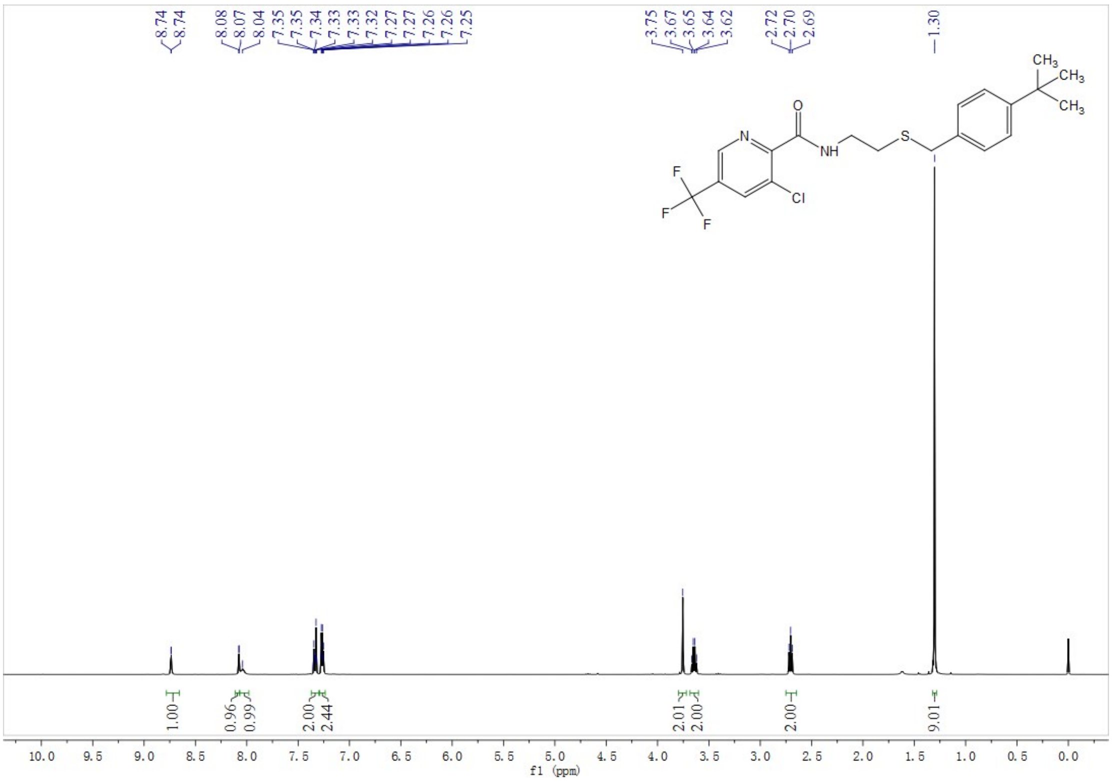

Fig. S58 ^{1}H NMR spectra of compound **E20**

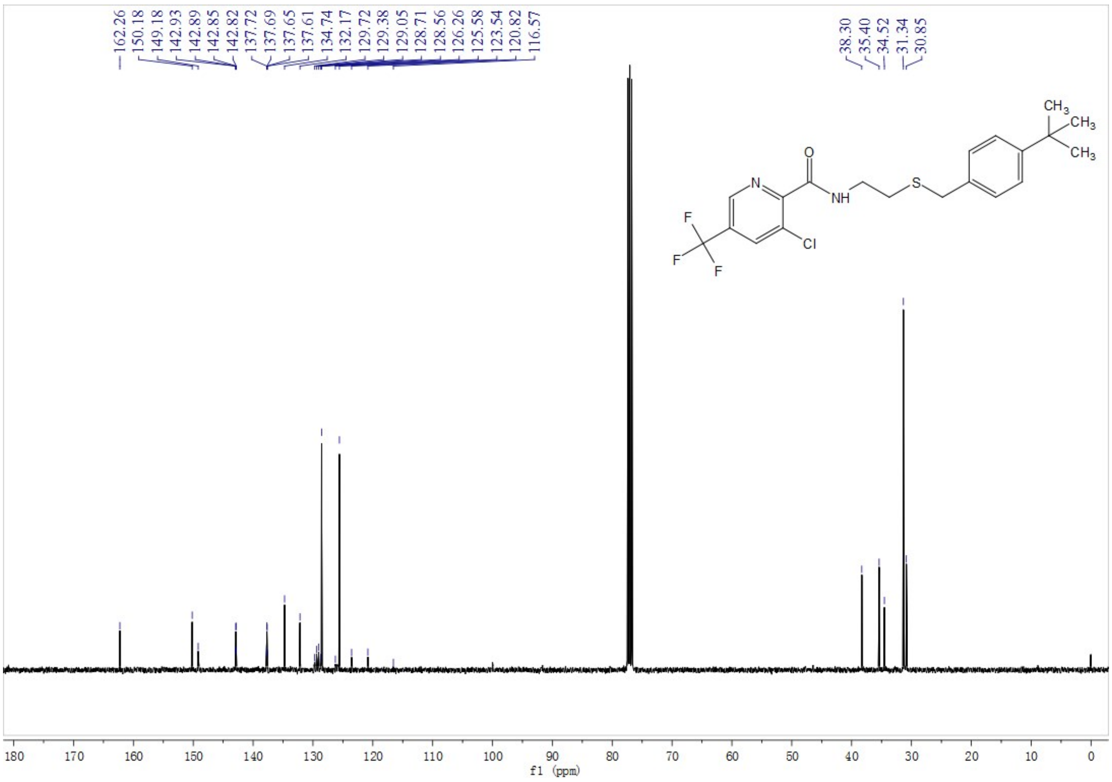

Fig. S59 ^{13}C NMR spectra of compound **E20**

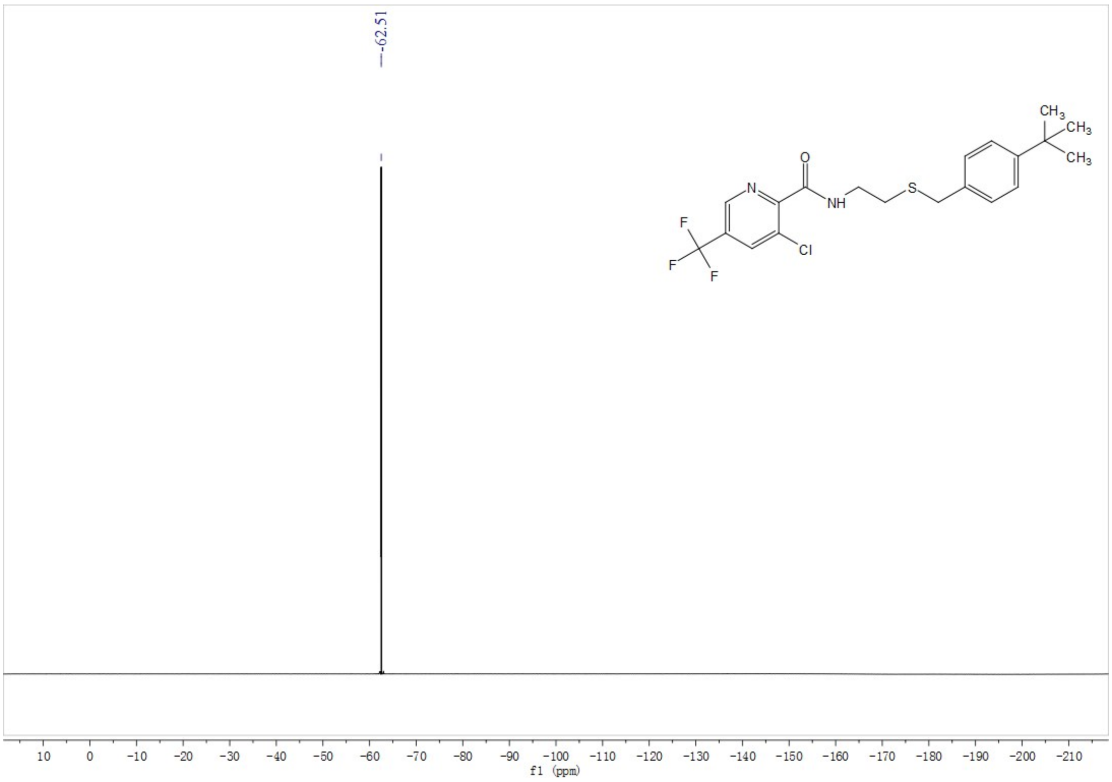

Fig. S60 ^{19}F NMR spectra of compound **E20**

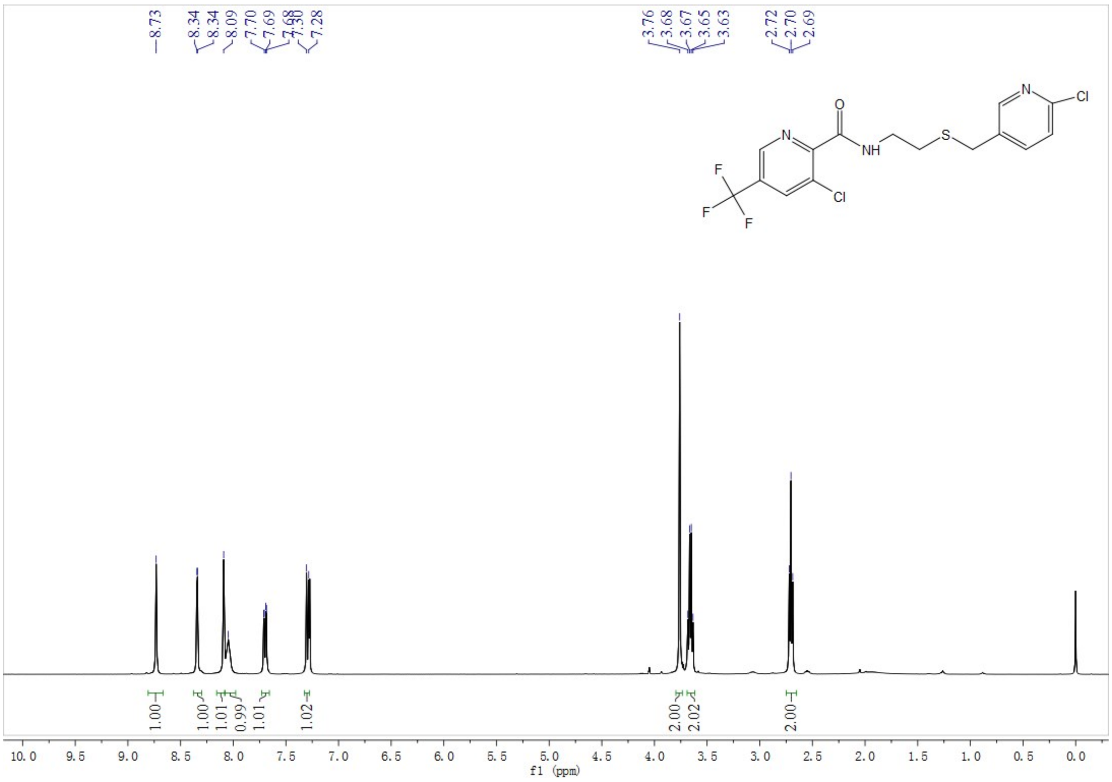

Fig. S61 ^{1}H NMR spectra of compound **E21**

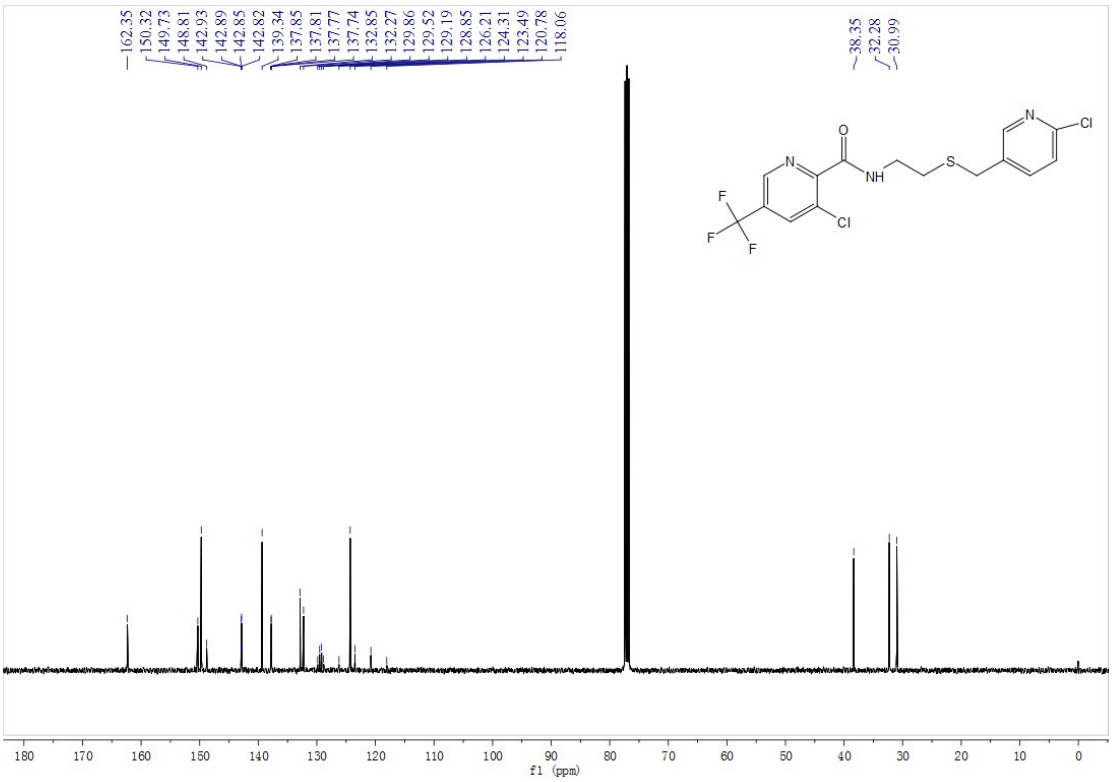

Fig. S62 ^{13}C NMR spectra of compound **E21**

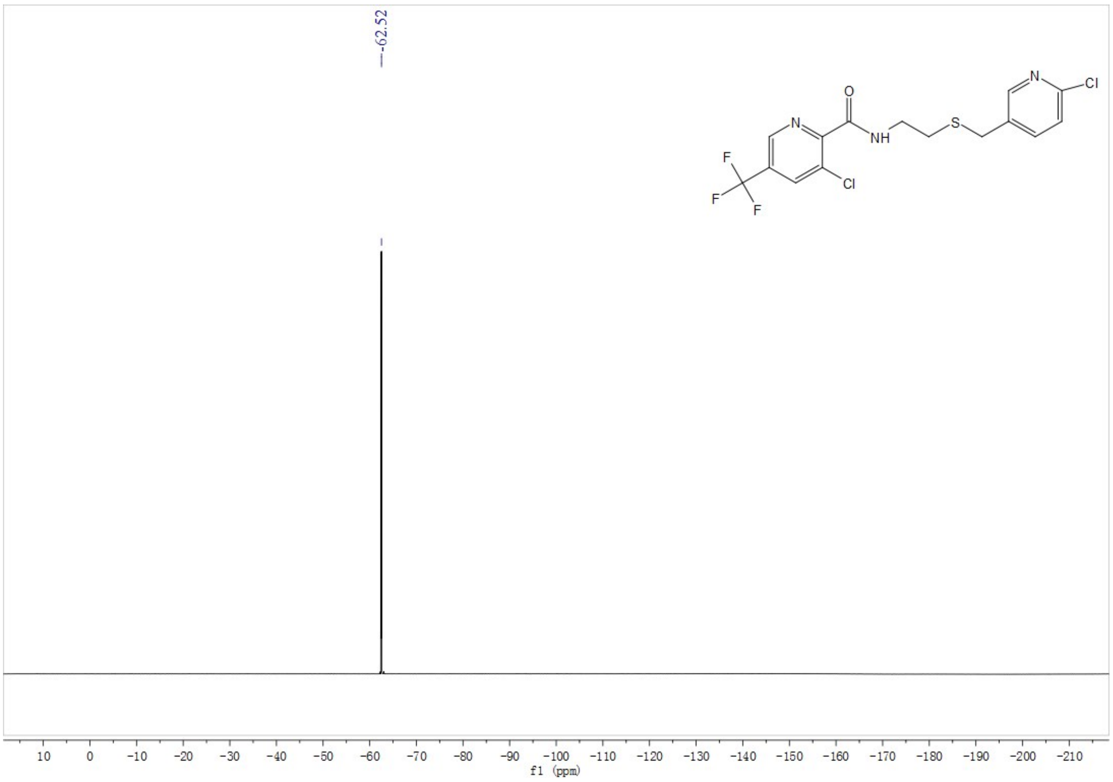

Fig. S63 ^{19}F NMR spectra of compound **E21**

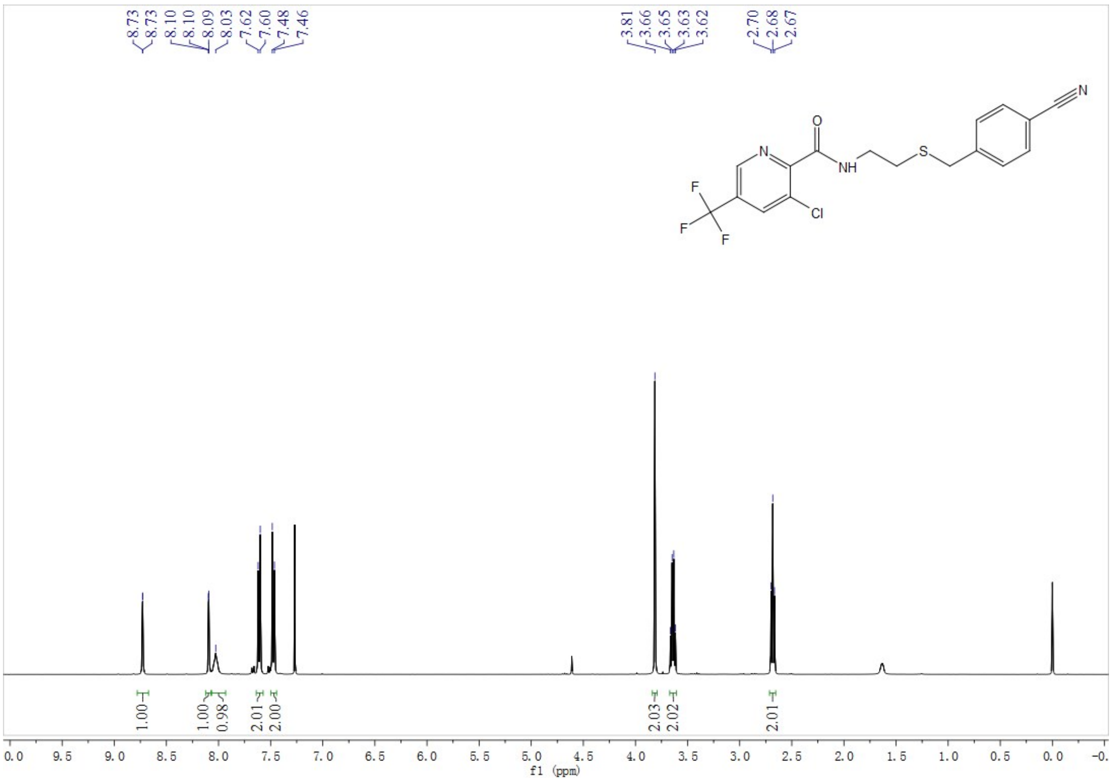

Fig. S64 ^{1}H NMR spectra of compound **E22**

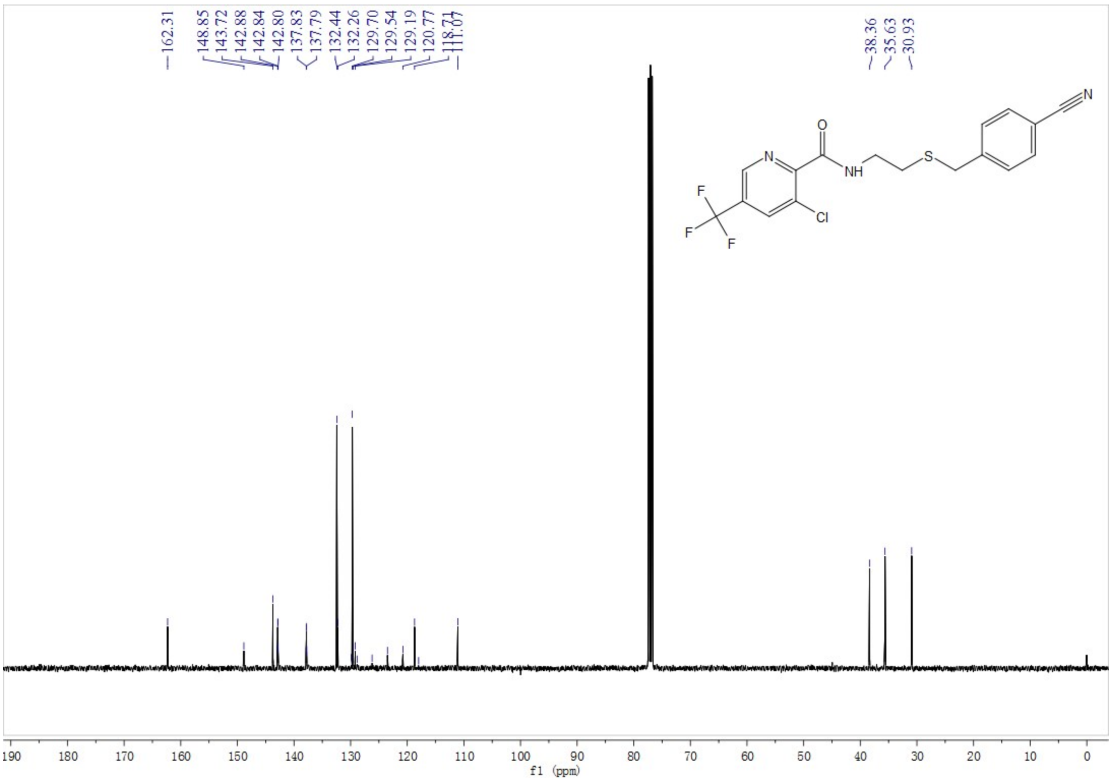

Fig. S65 ^{13}C NMR spectra of compound **E22**

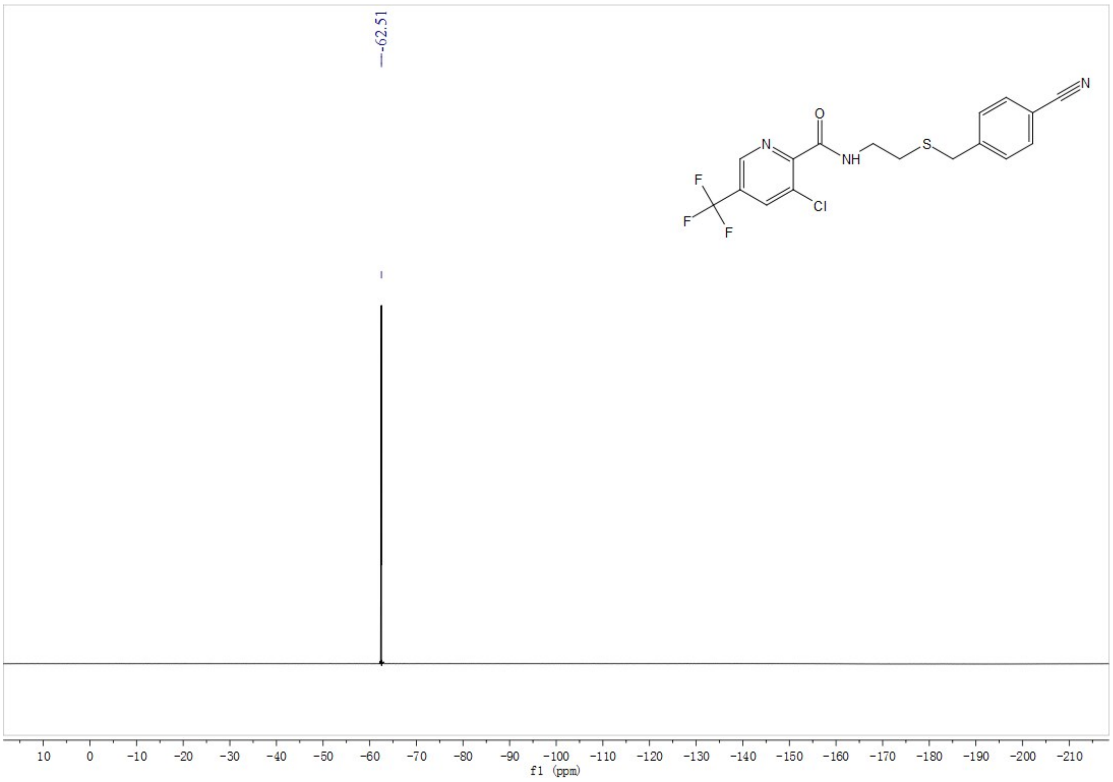

Fig. S66 ^{19}F NMR spectra of compound **E22**

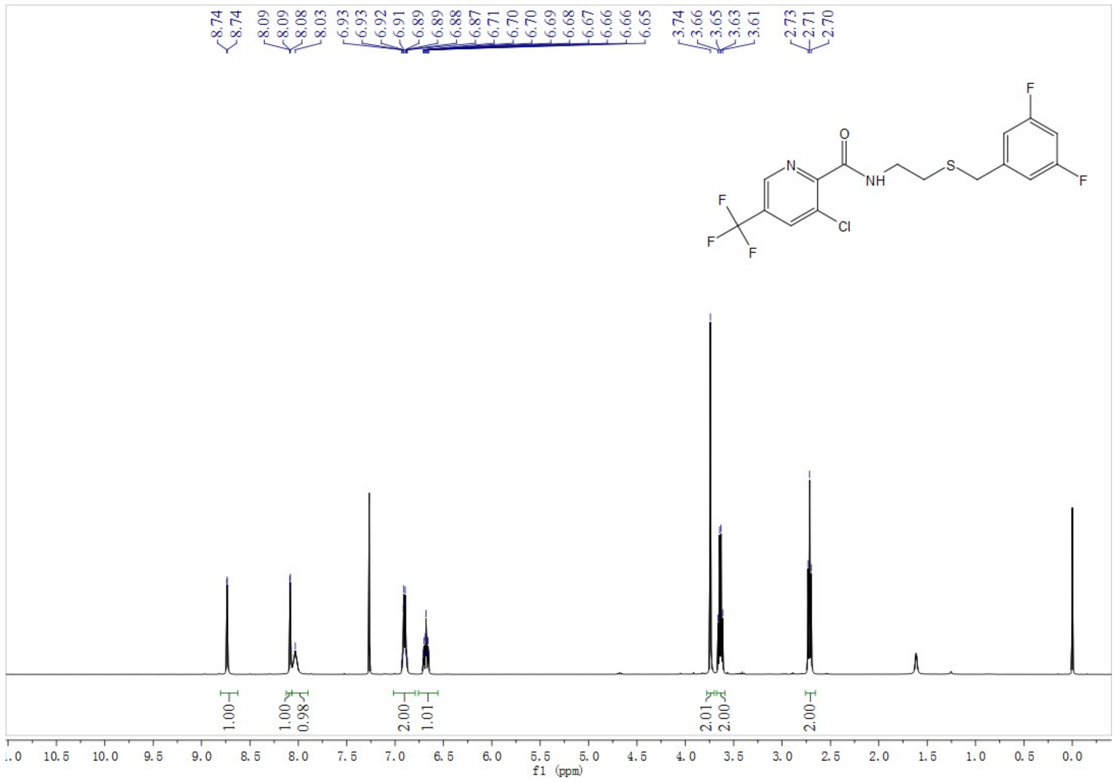

Fig. S67 ^{1}H NMR spectra of compound **E23**

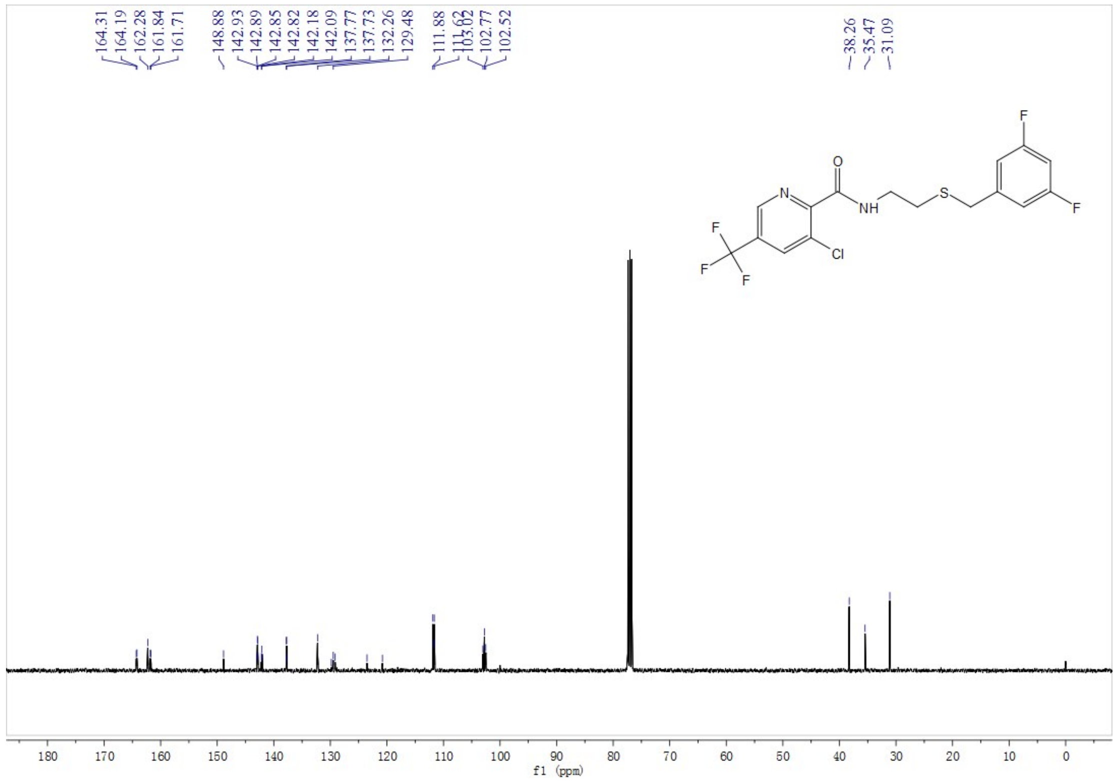

Fig. S68 ^{13}C NMR spectra of compound **E23**

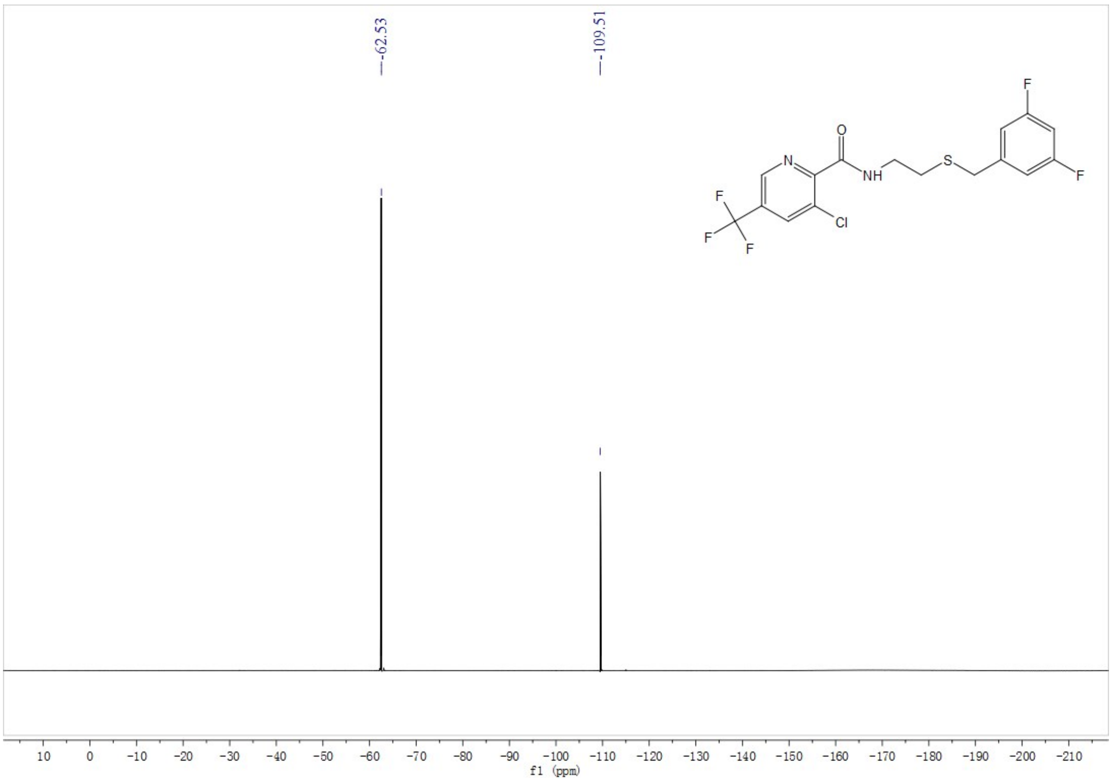

Fig. S69 ^{19}F NMR spectra of compound **E23**

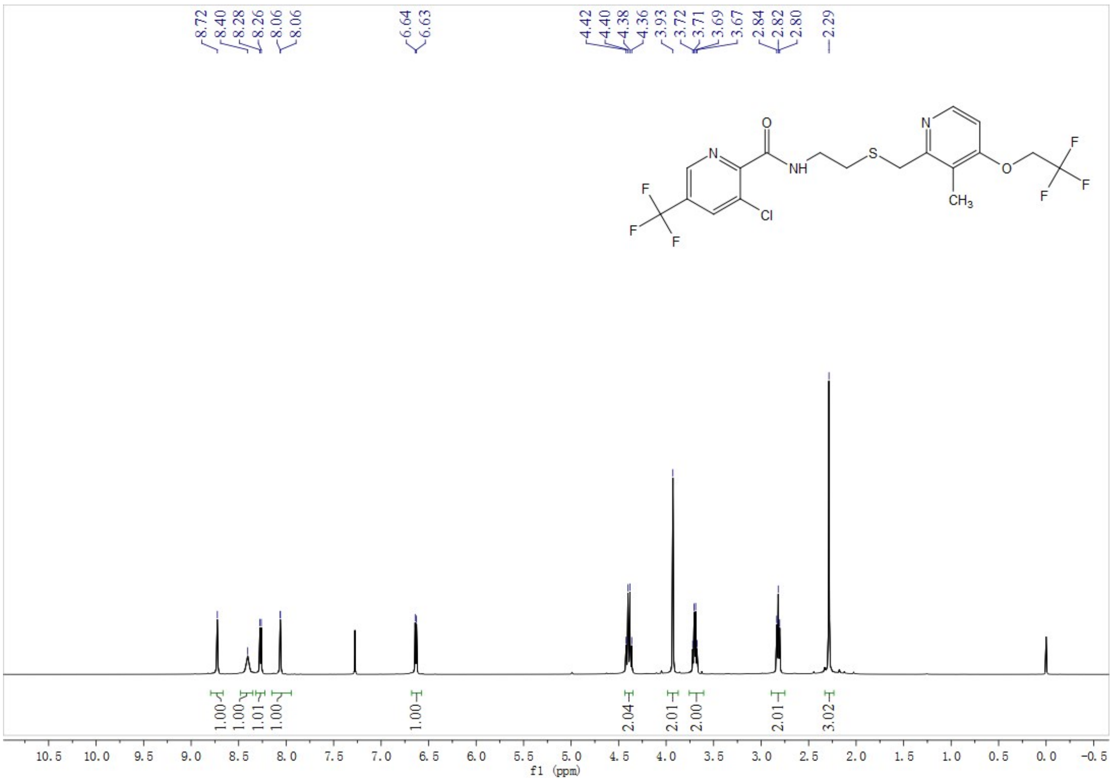

Fig. S70 ^{1}H NMR spectra of compound **E24**

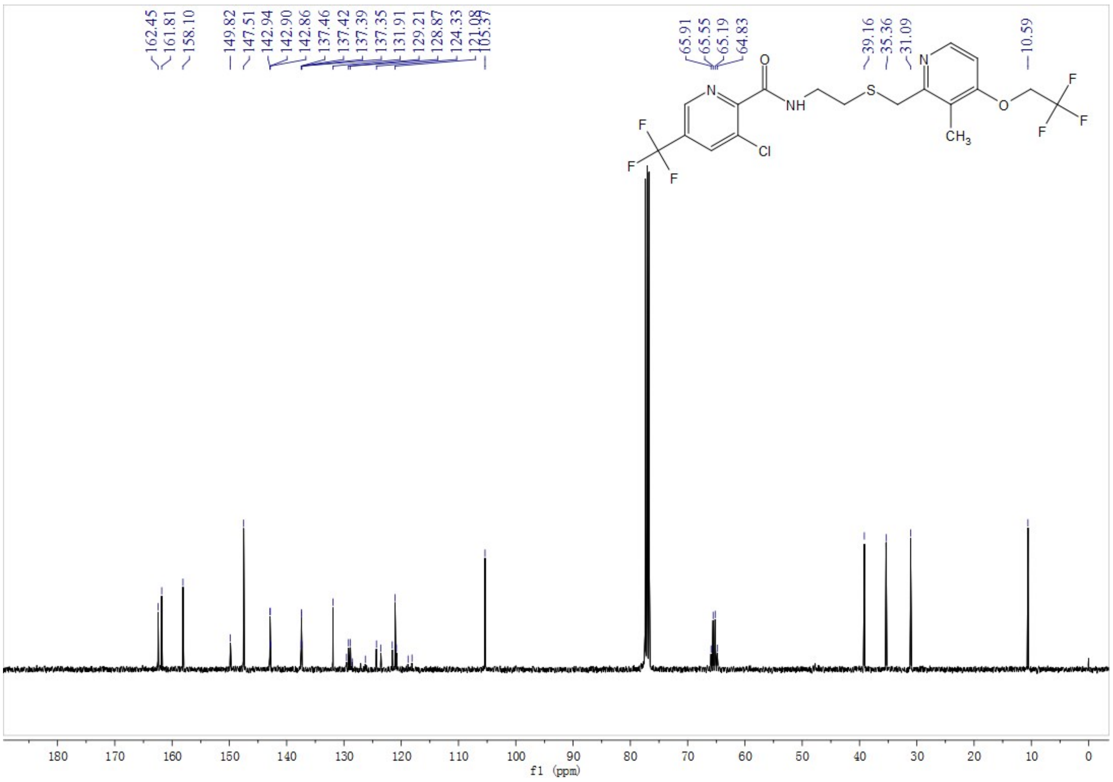

Fig. S71 ^{13}C NMR spectra of compound **E24**

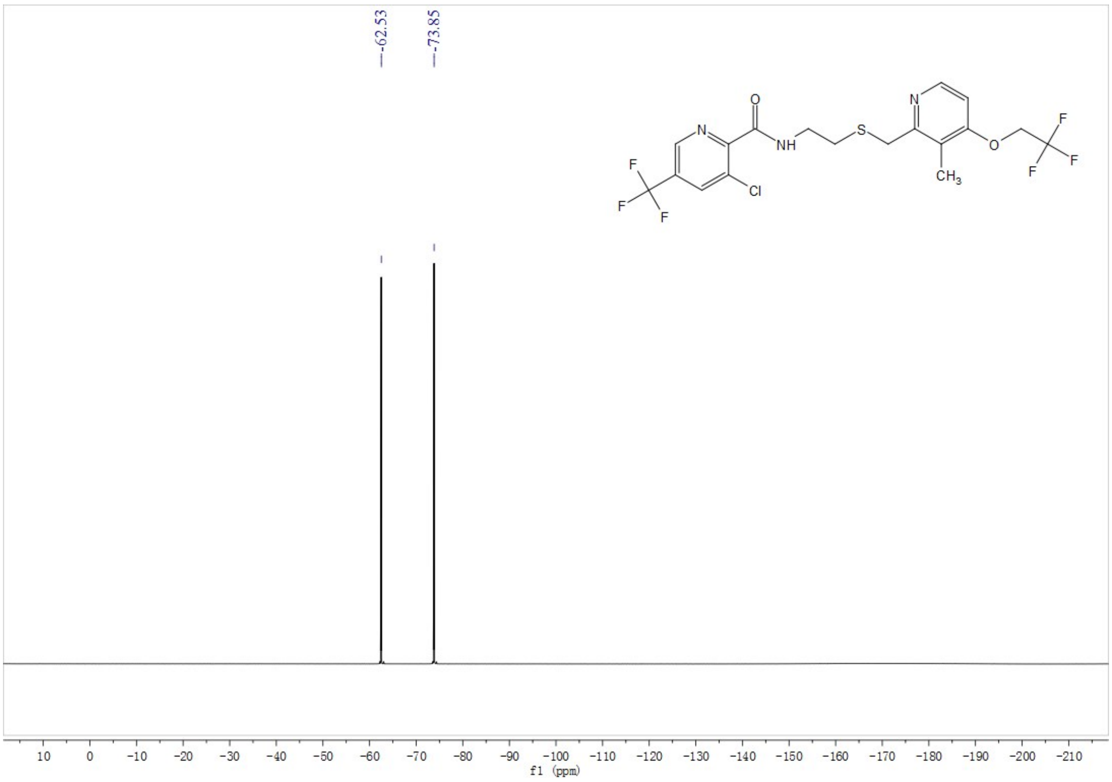

Fig. S72 ^{19}F NMR spectra of compound **E24**

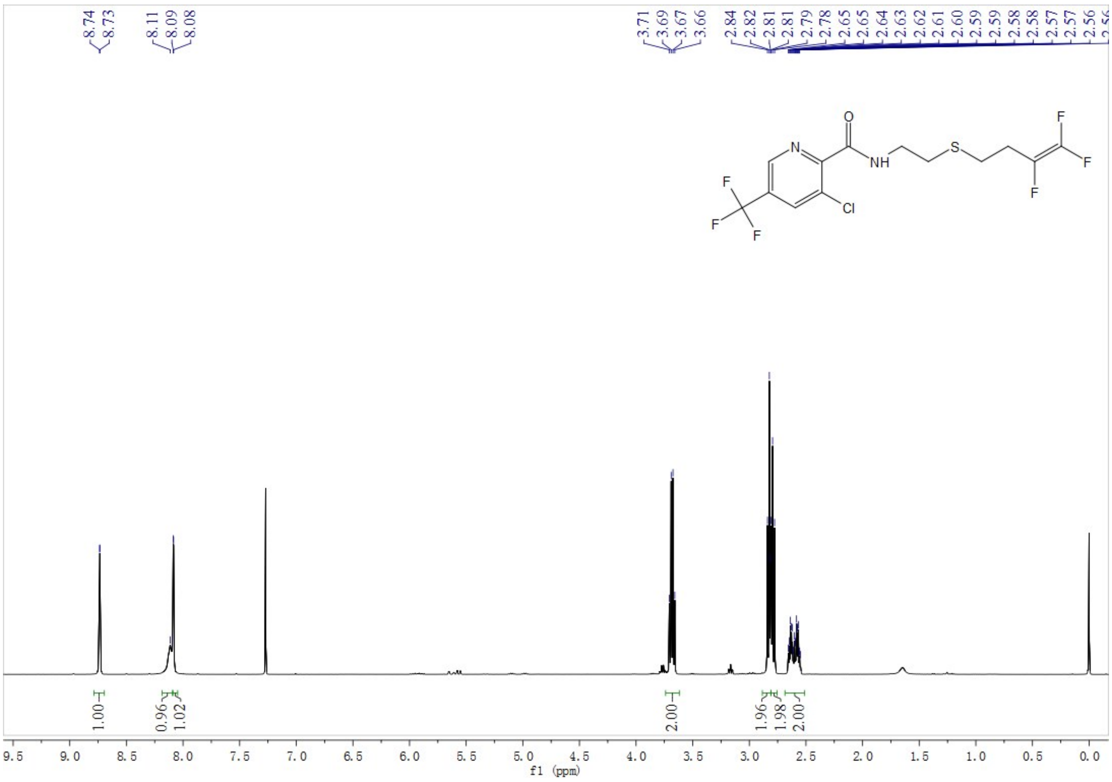

Fig. S73 ¹H NMR spectra of compound **E25**

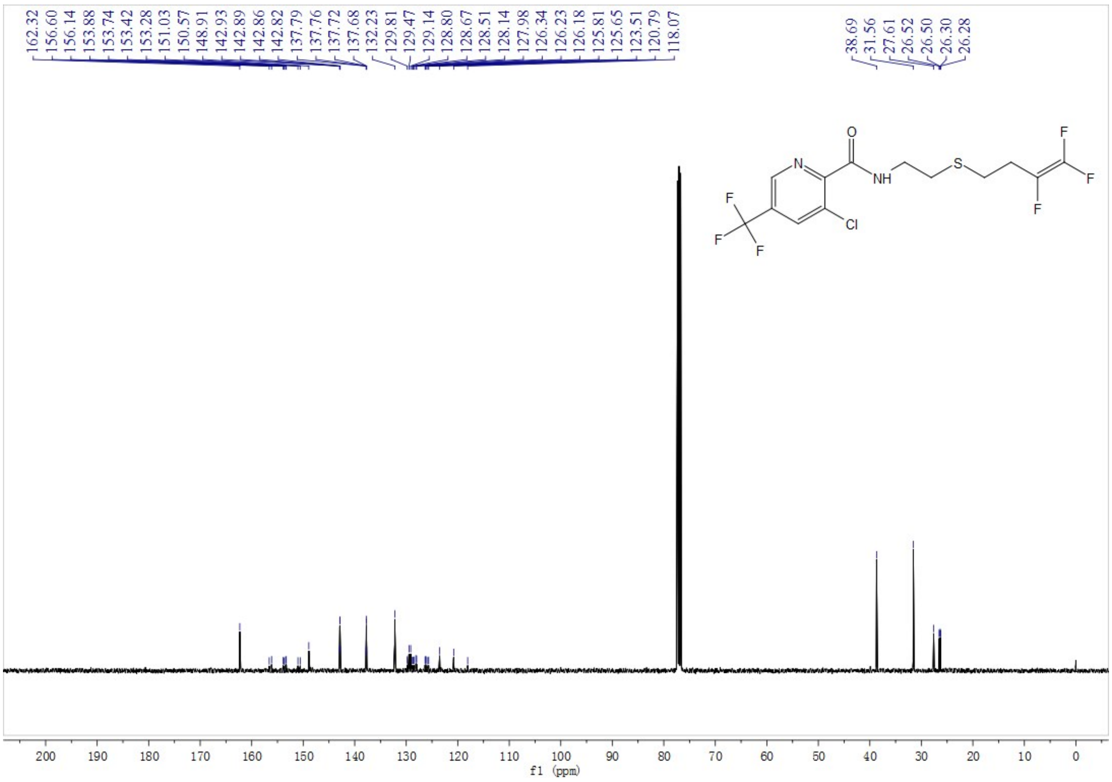

Fig. S74 ¹³C NMR spectra of compound **E25**

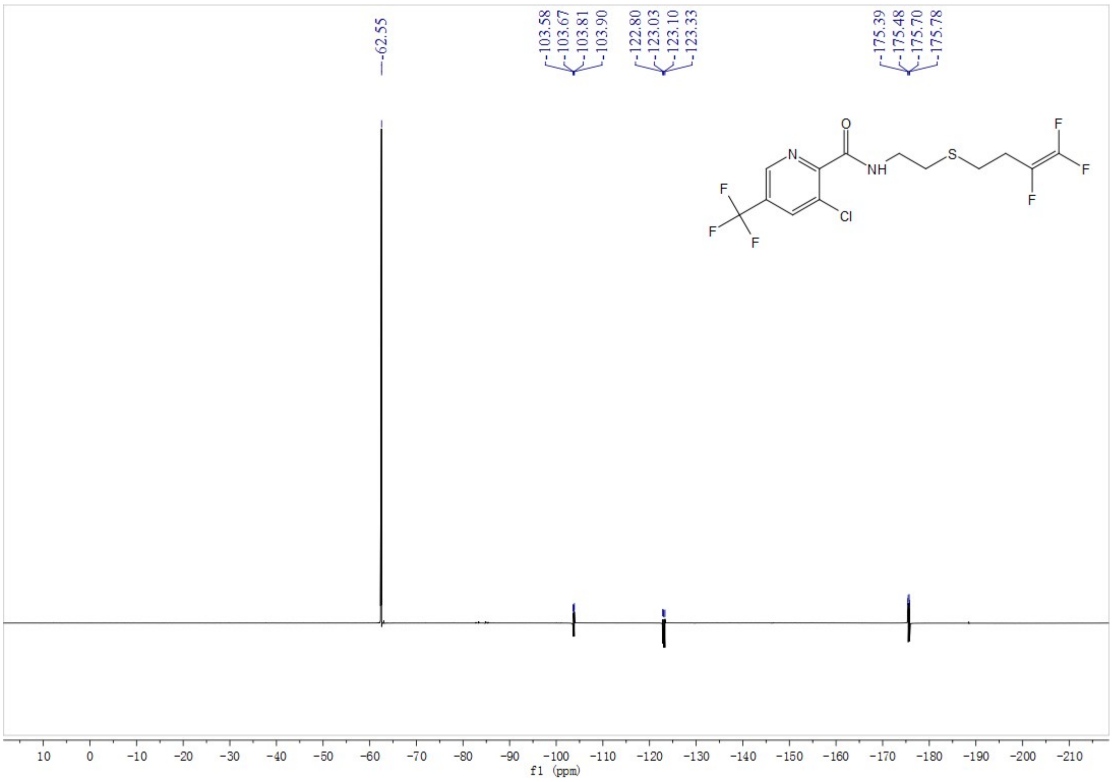

Fig. S75 ^{19}F NMR spectra of compound **E25**

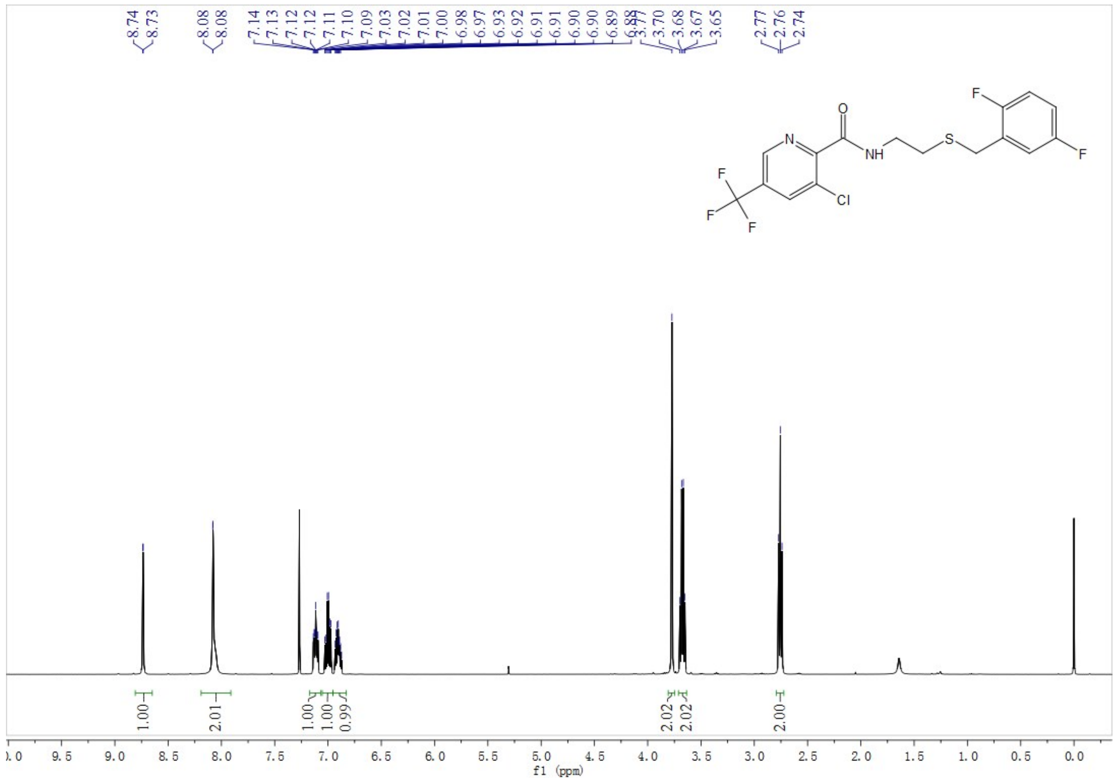

Fig. S76 ^{1}H NMR spectra of compound **E26**

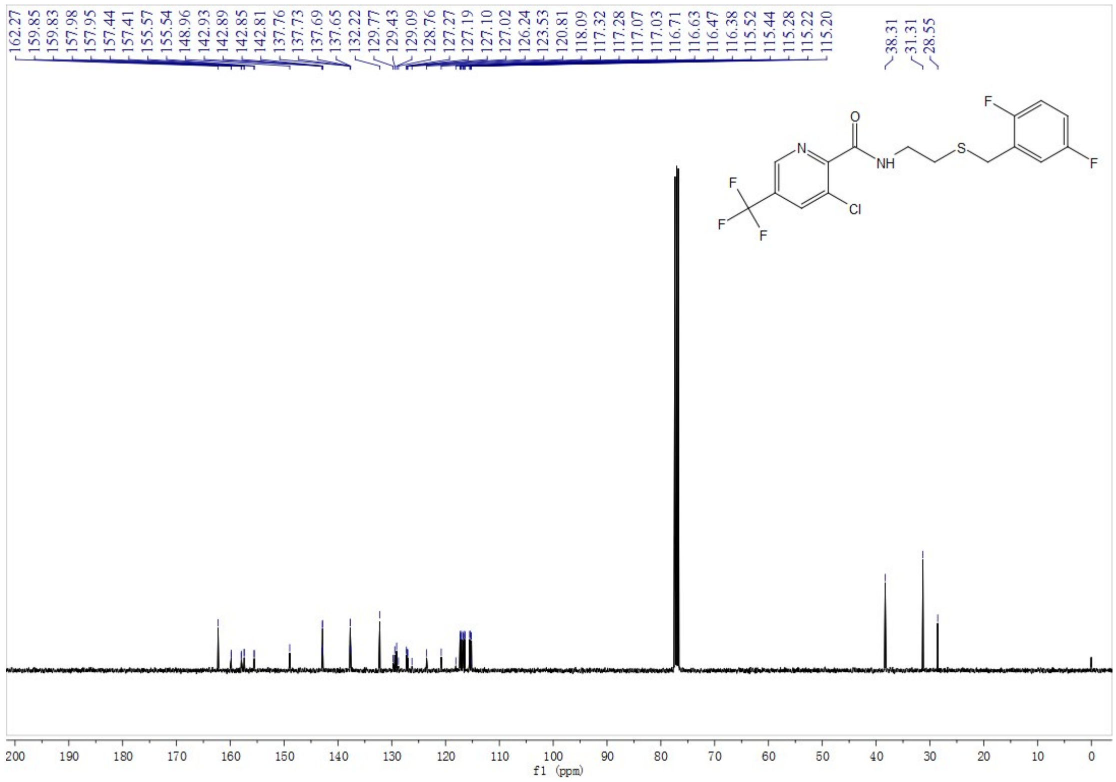

Fig. S77 ^{13}C NMR spectra of compound **E26**

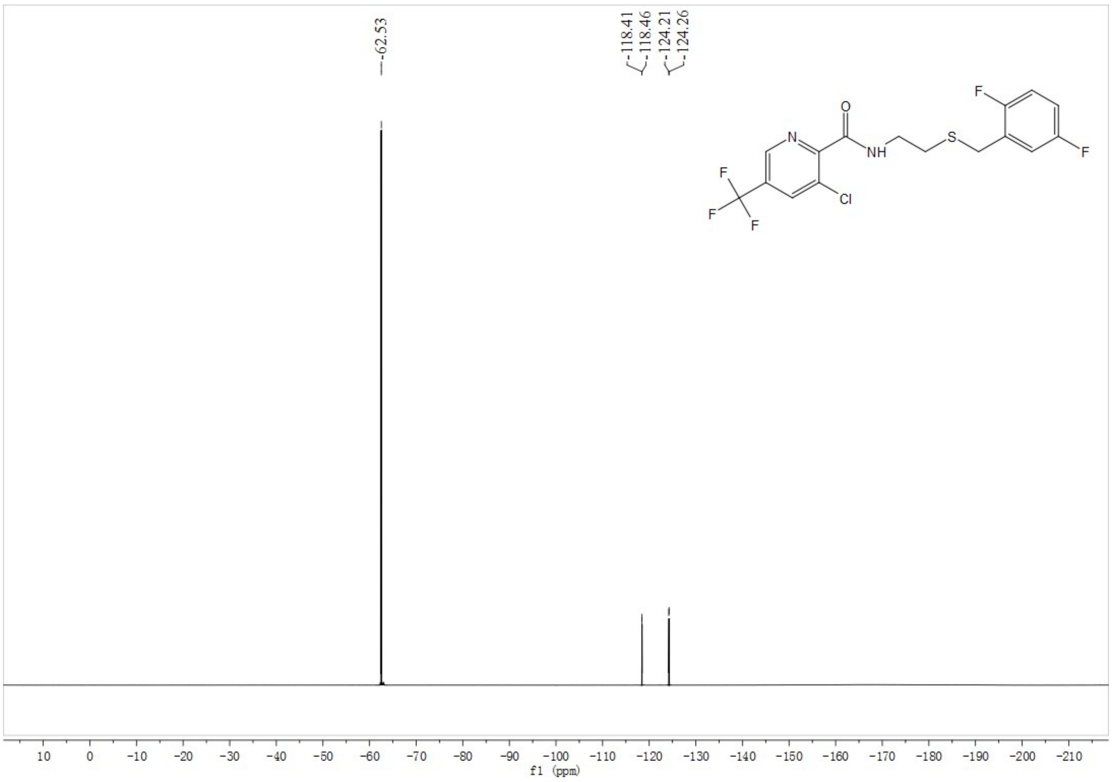

Fig. S78 ^{19}F NMR spectra of compound **E26**

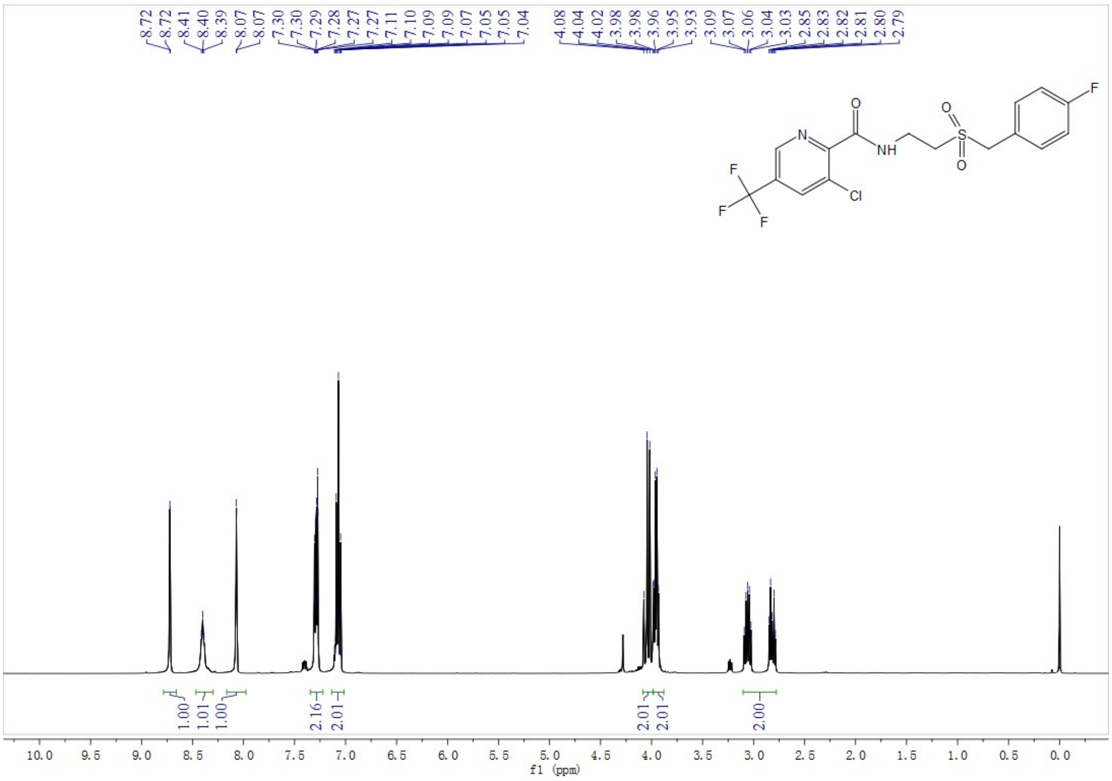

Fig. S79 ^{1}H NMR spectra of compound **F1**

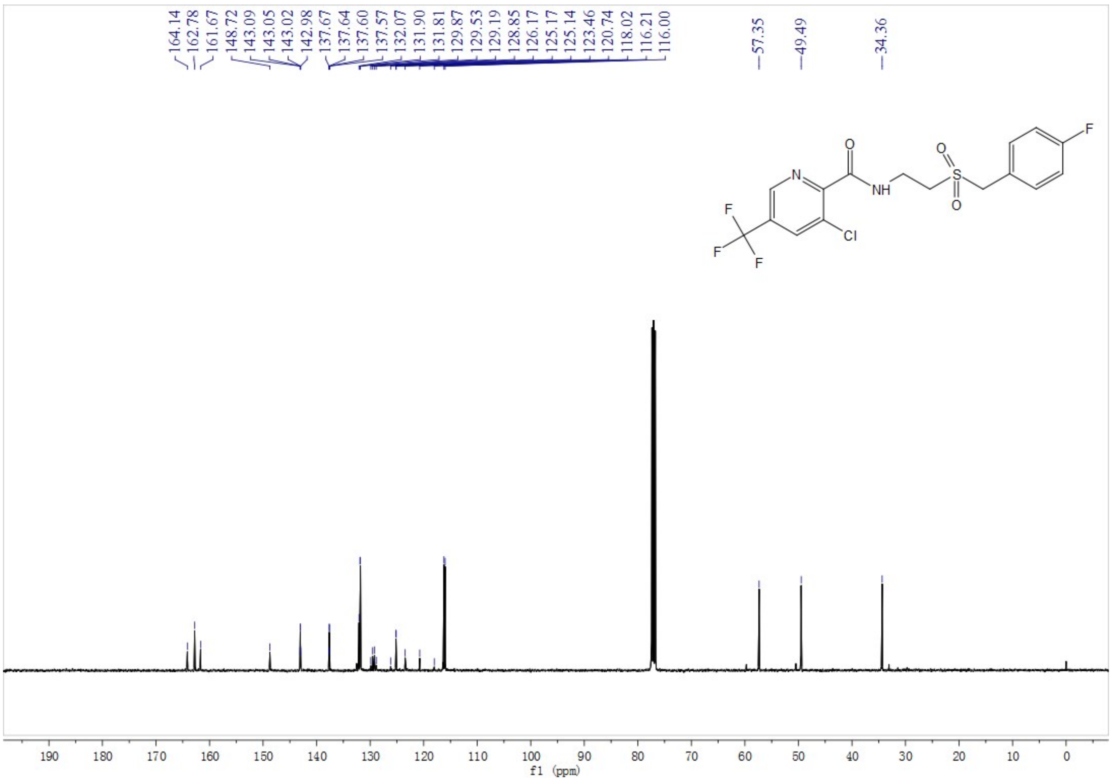

Fig. S80 ^{13}C NMR spectra of compound **F1**

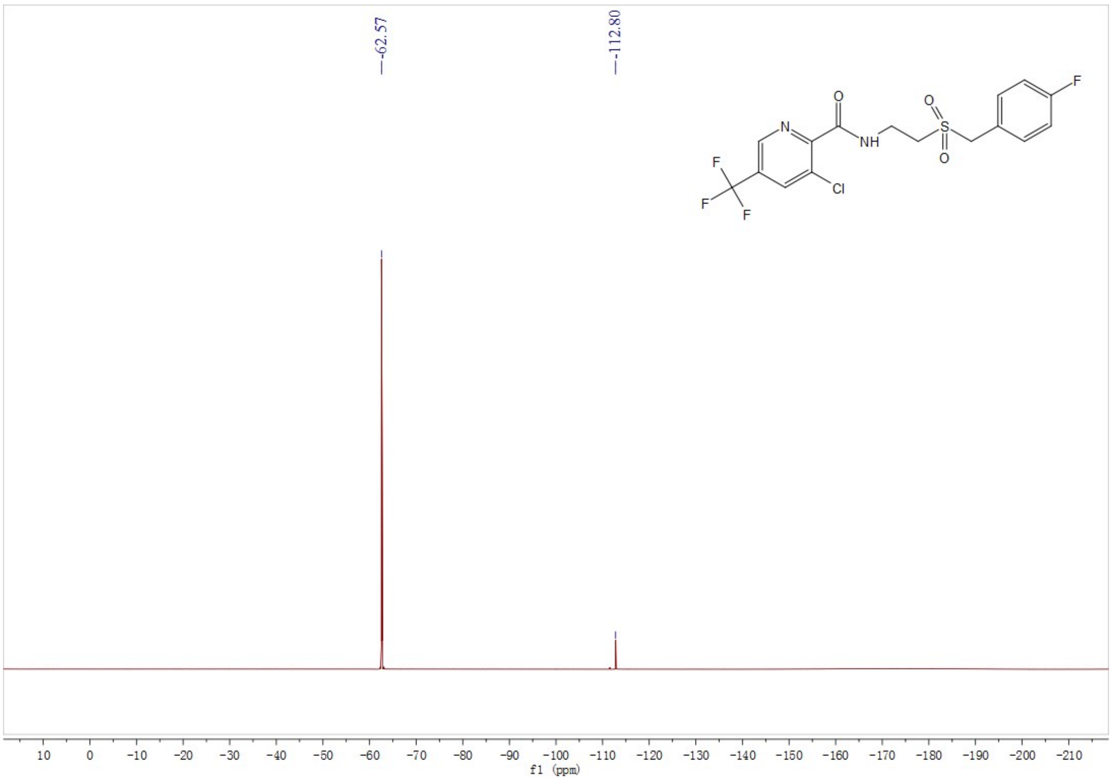

Fig. S81 ^{19}F NMR spectra of compound **F1**

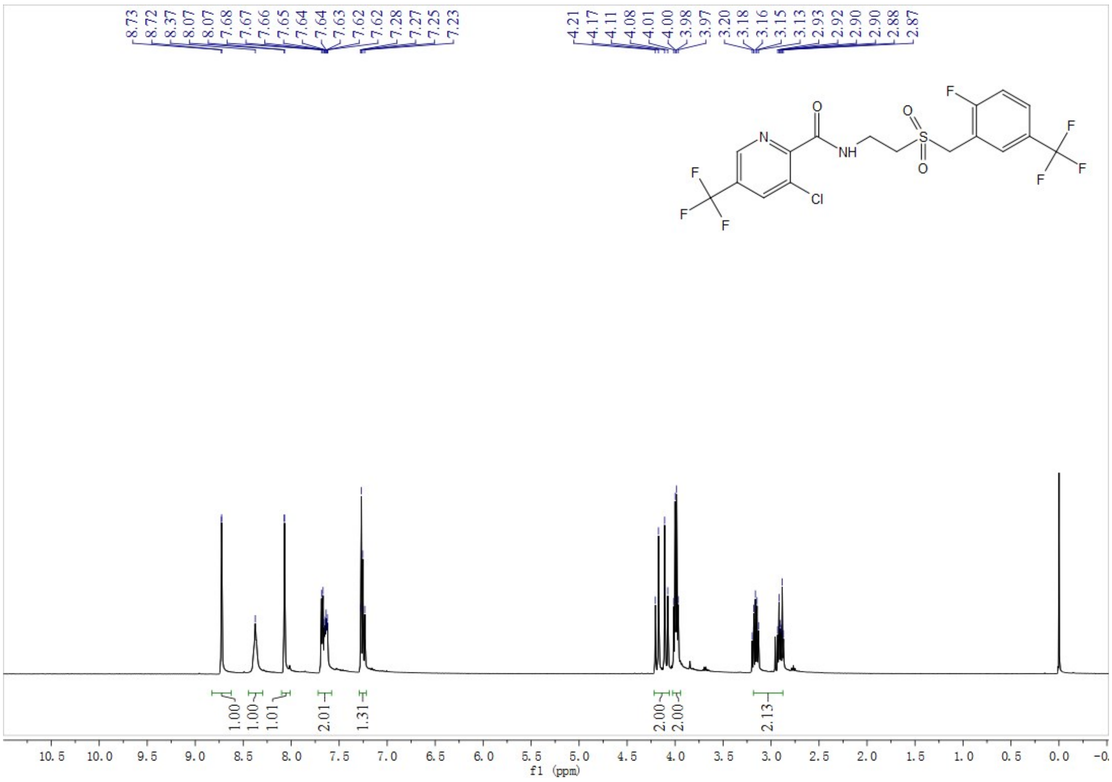

Fig. S82 ^{1}H NMR spectra of compound **F2**

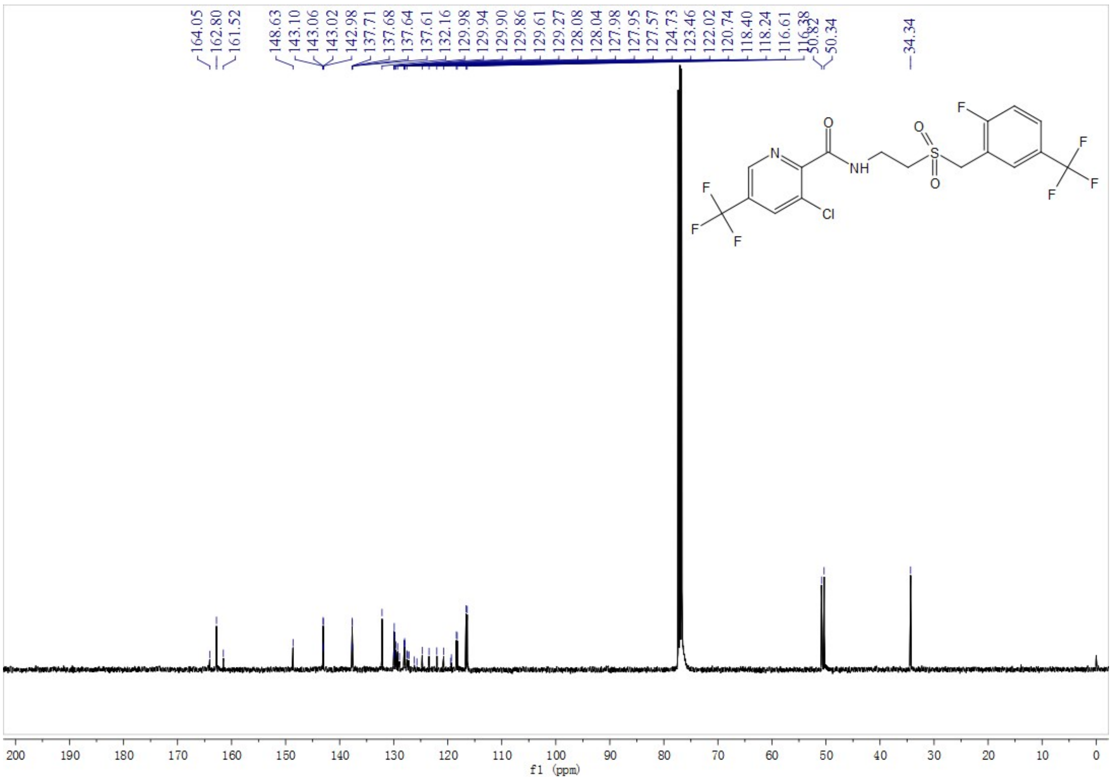

Fig. S83 ^{13}C NMR spectra of compound **F2**

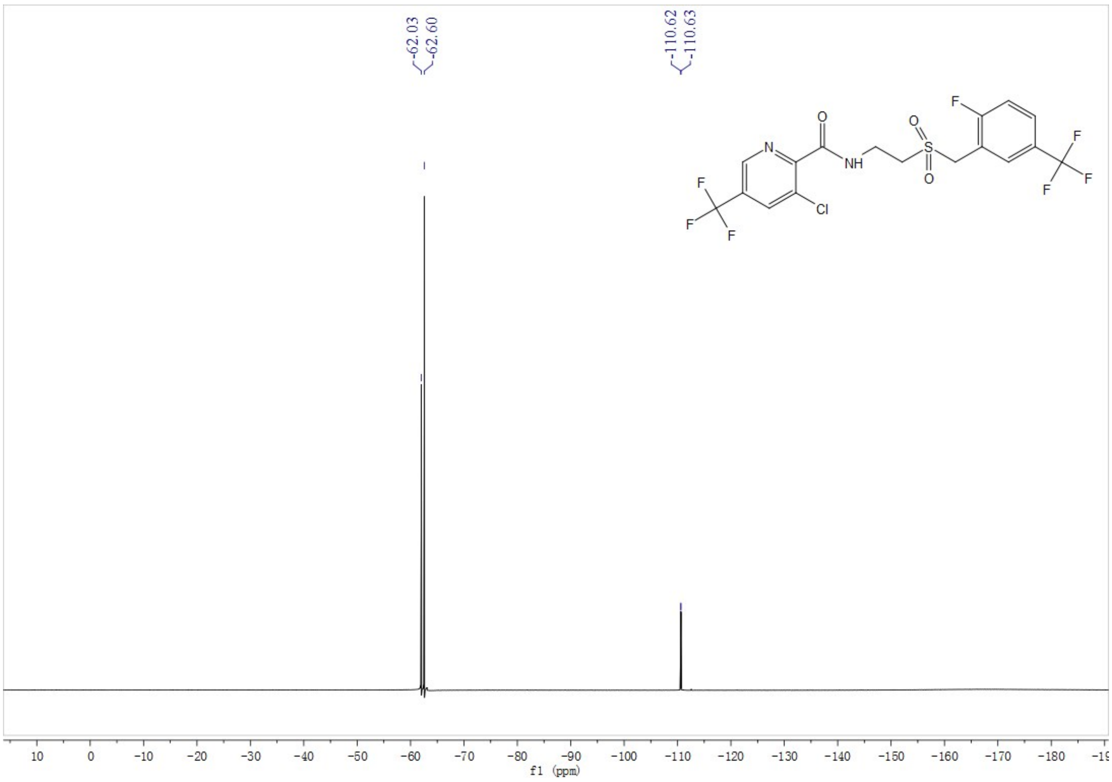

Fig. S84 ^{19}F NMR spectra of compound **F2**

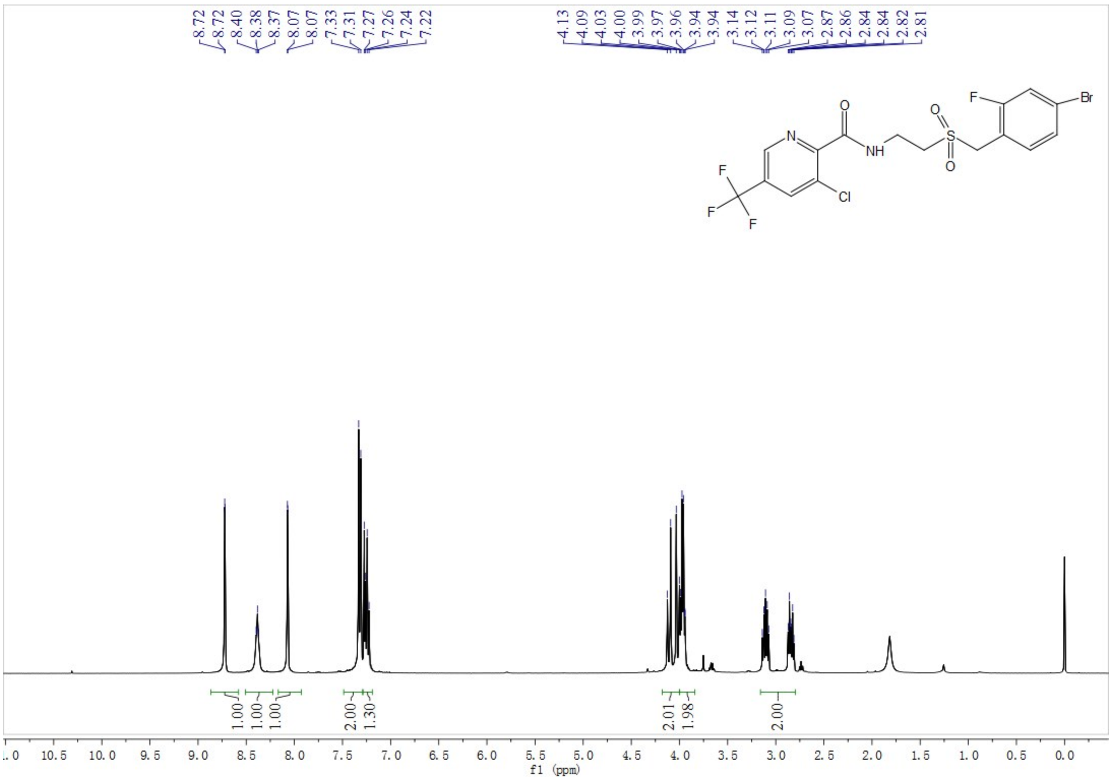

Fig. S85 ^{1}H NMR spectra of compound **F3**

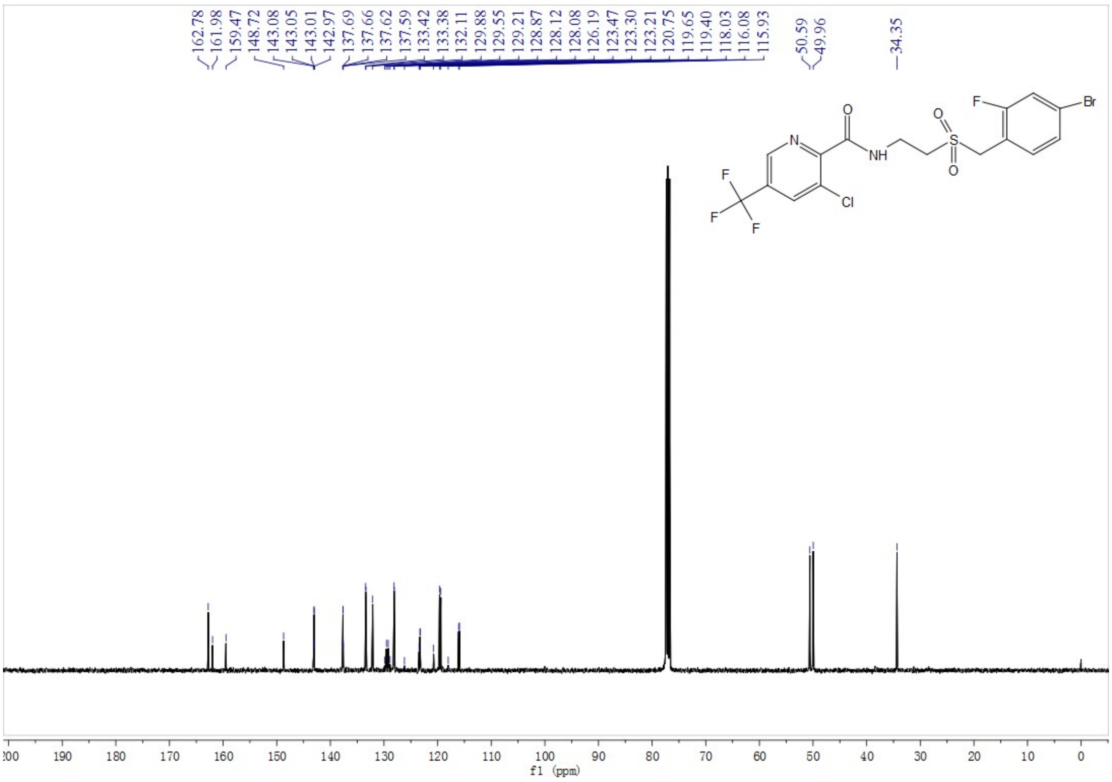

Fig. S86 ^{13}C NMR spectra of compound **F3**

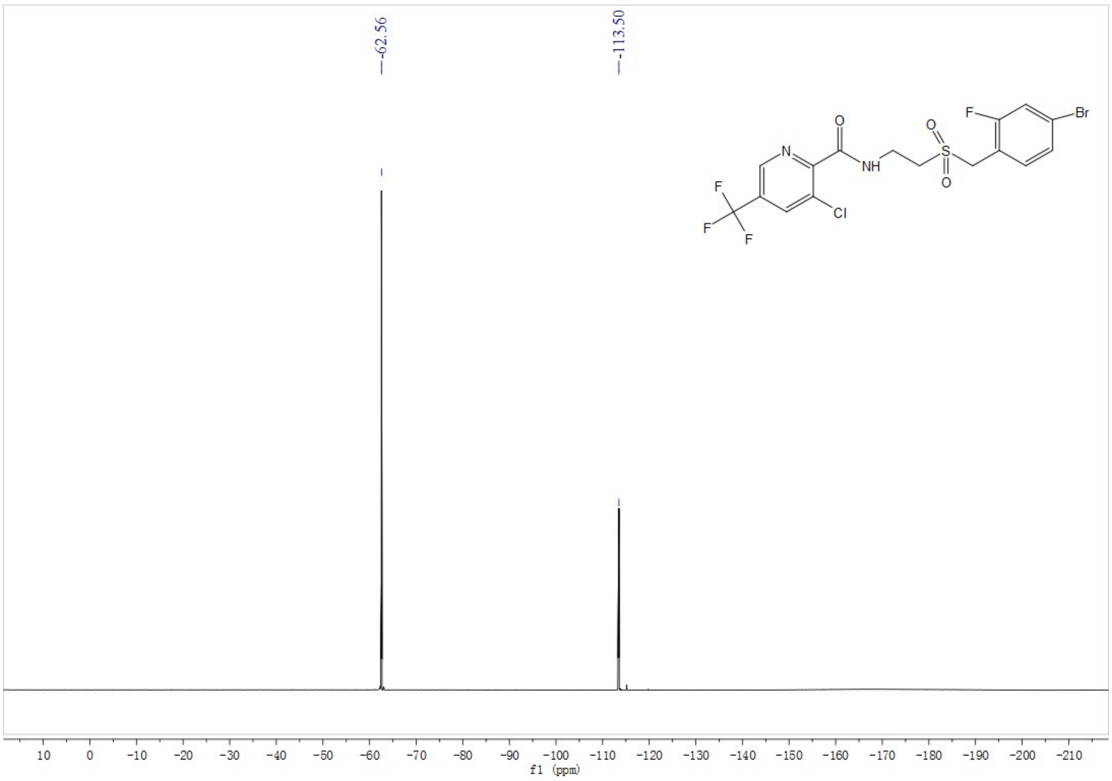

Fig. S87 ^{19}F NMR spectra of compound **F3**

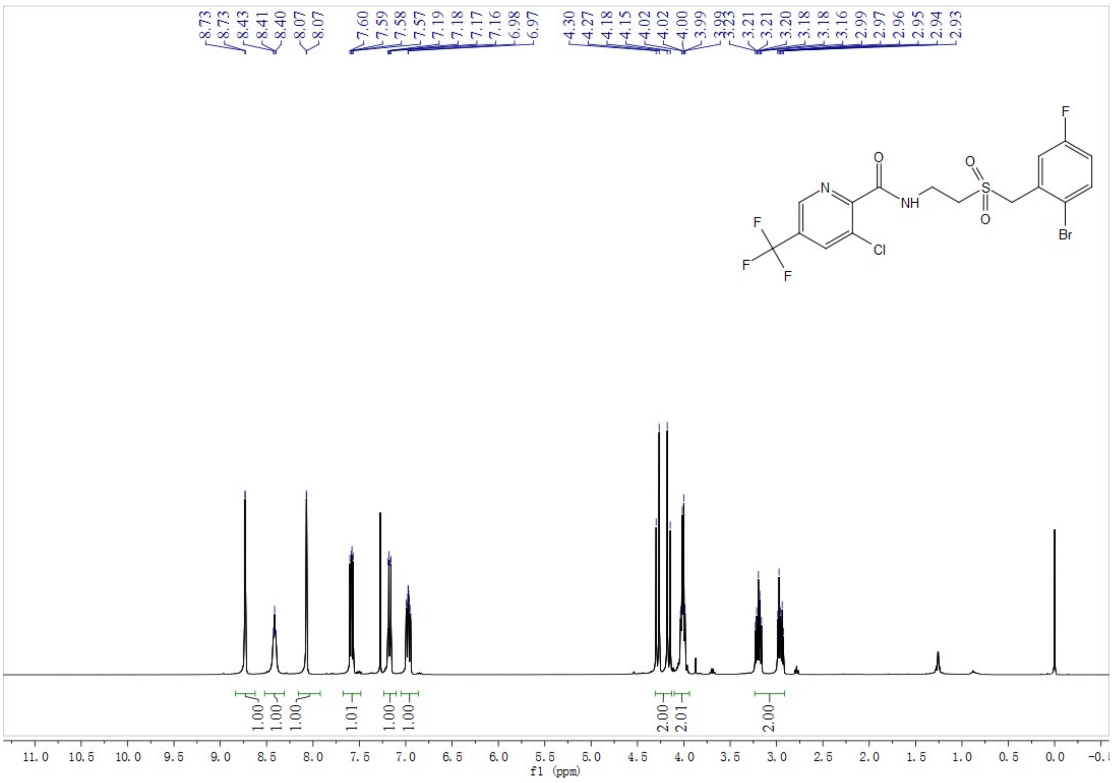

Fig. S88 ^{1}H NMR spectra of compound **F4**

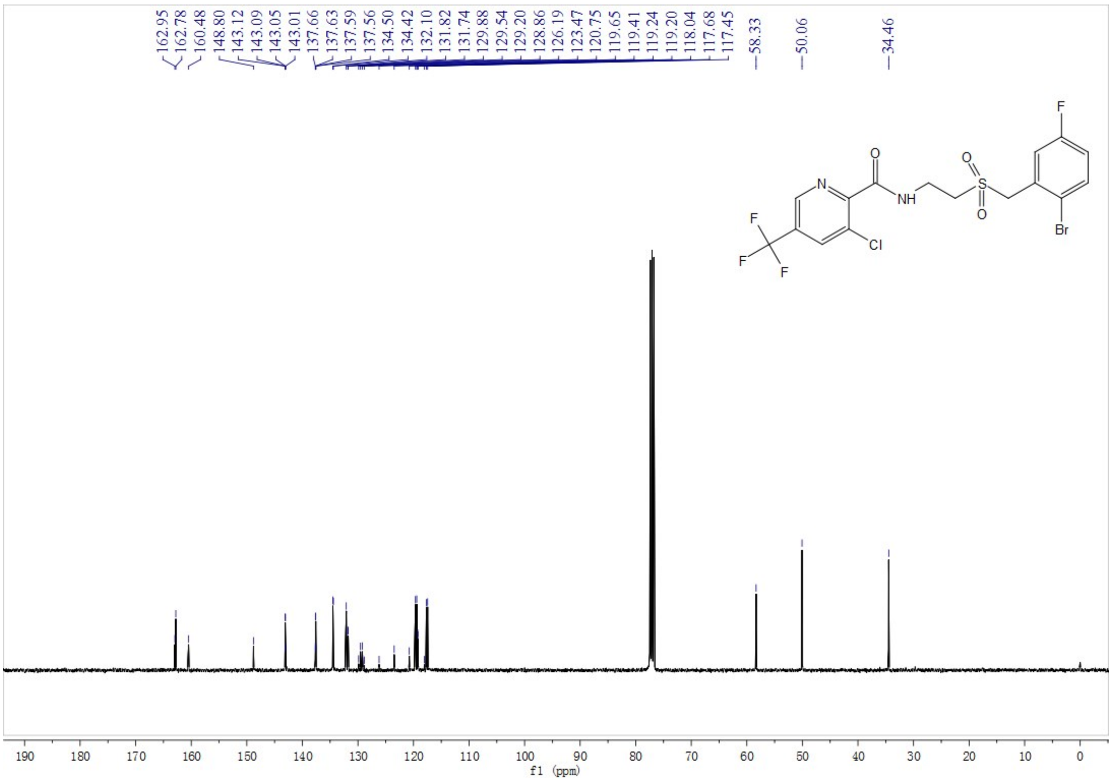

Fig. S89 ^{13}C NMR spectra of compound **F4**

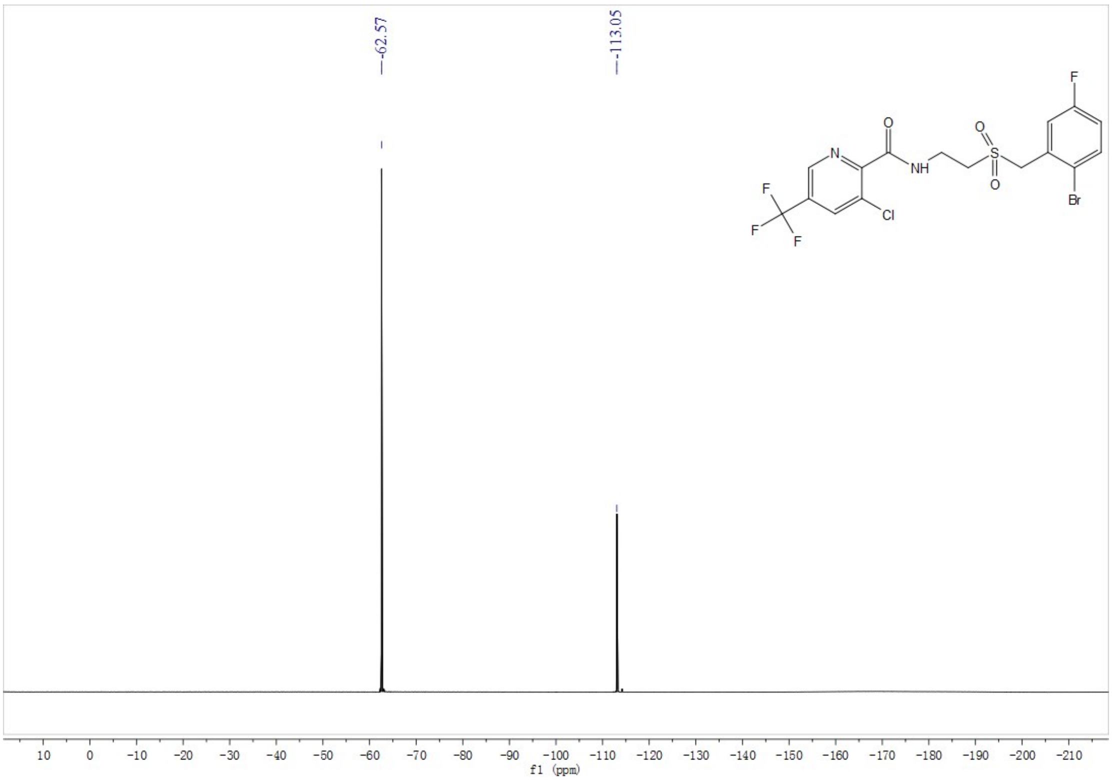

Fig. S90 ^{19}F NMR spectra of compound **F4**

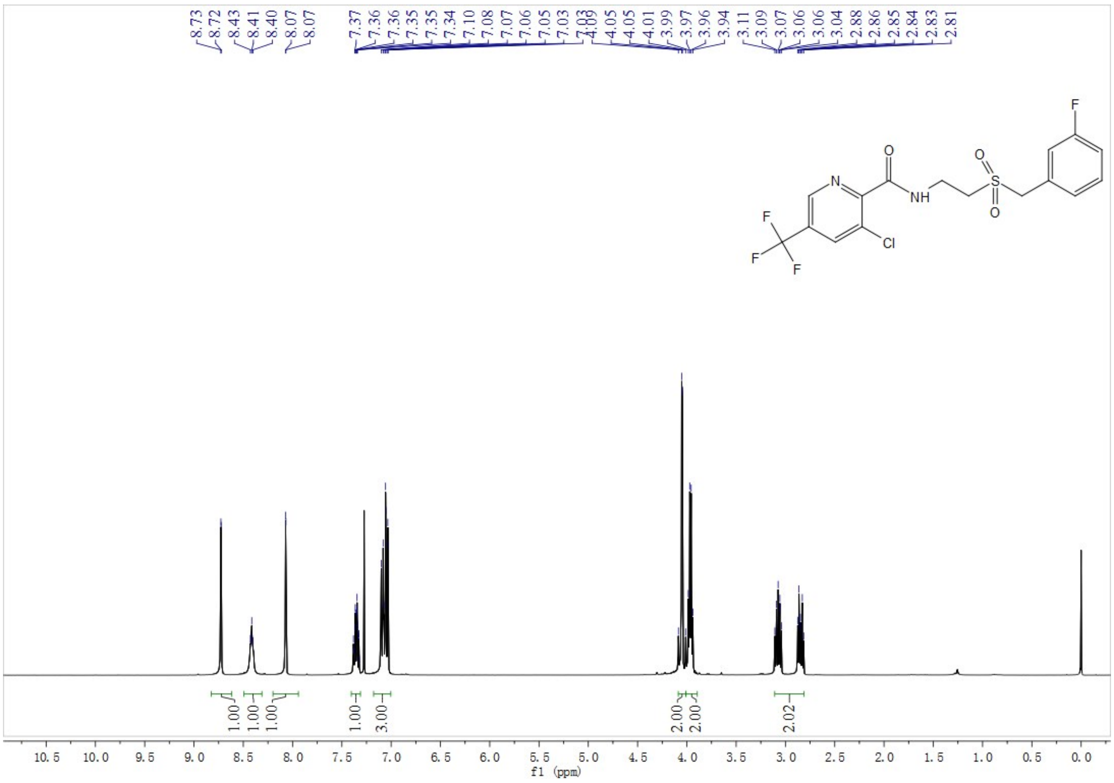

Fig. S91 ^{1}H NMR spectra of compound **F5**

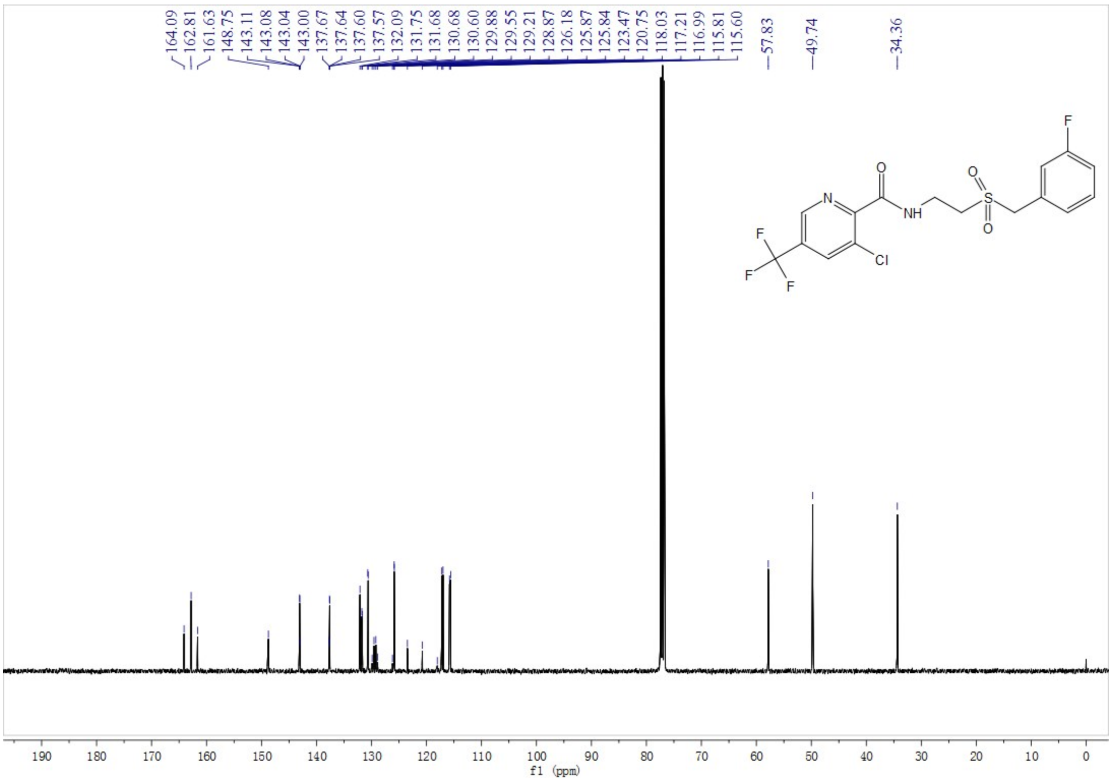

Fig. S92 ^{13}C NMR spectra of compound **F5**

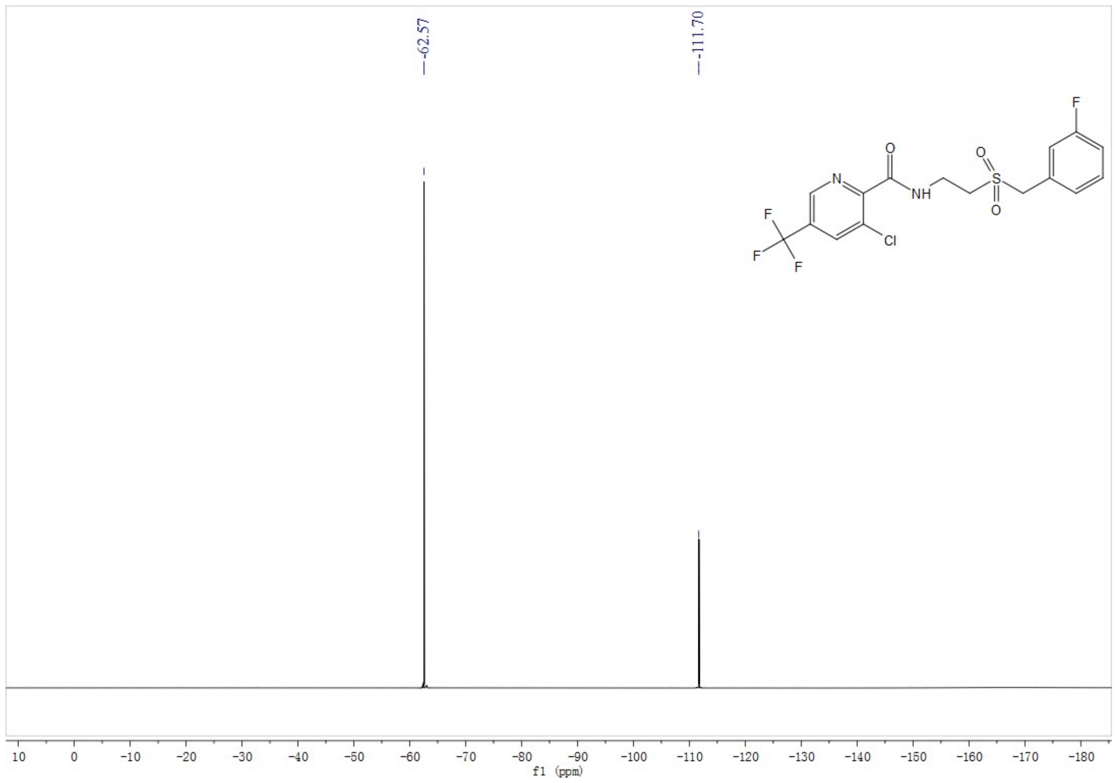

Fig. S93 ^{19}F NMR spectra of compound **F5**

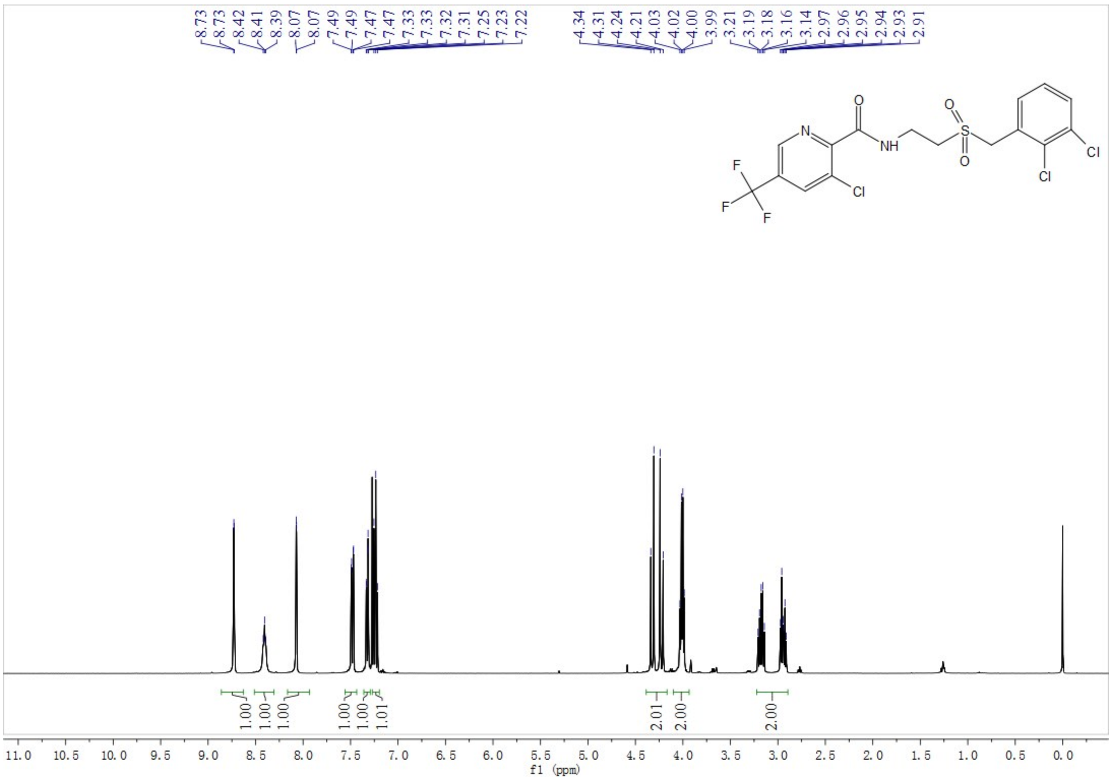

Fig. S94 ¹H NMR spectra of compound **F6**

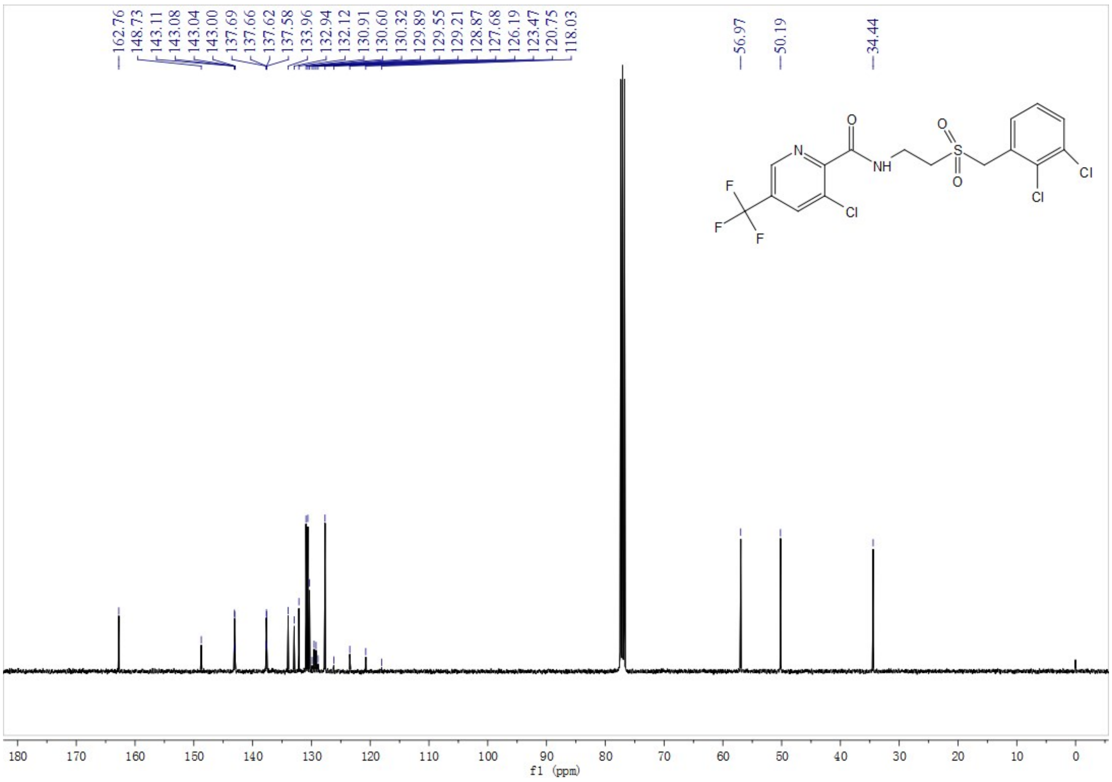

Fig. S95 ¹³C NMR spectra of compound **F6**

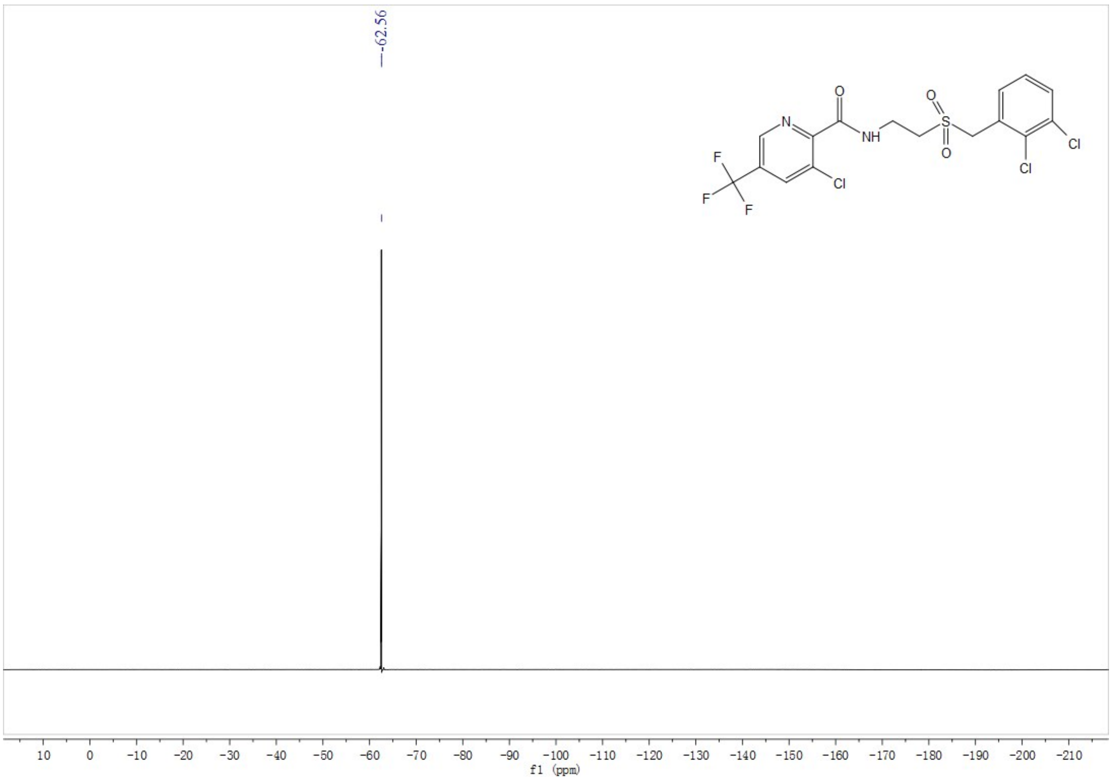

Fig. S96 ^{19}F NMR spectra of compound **F6**

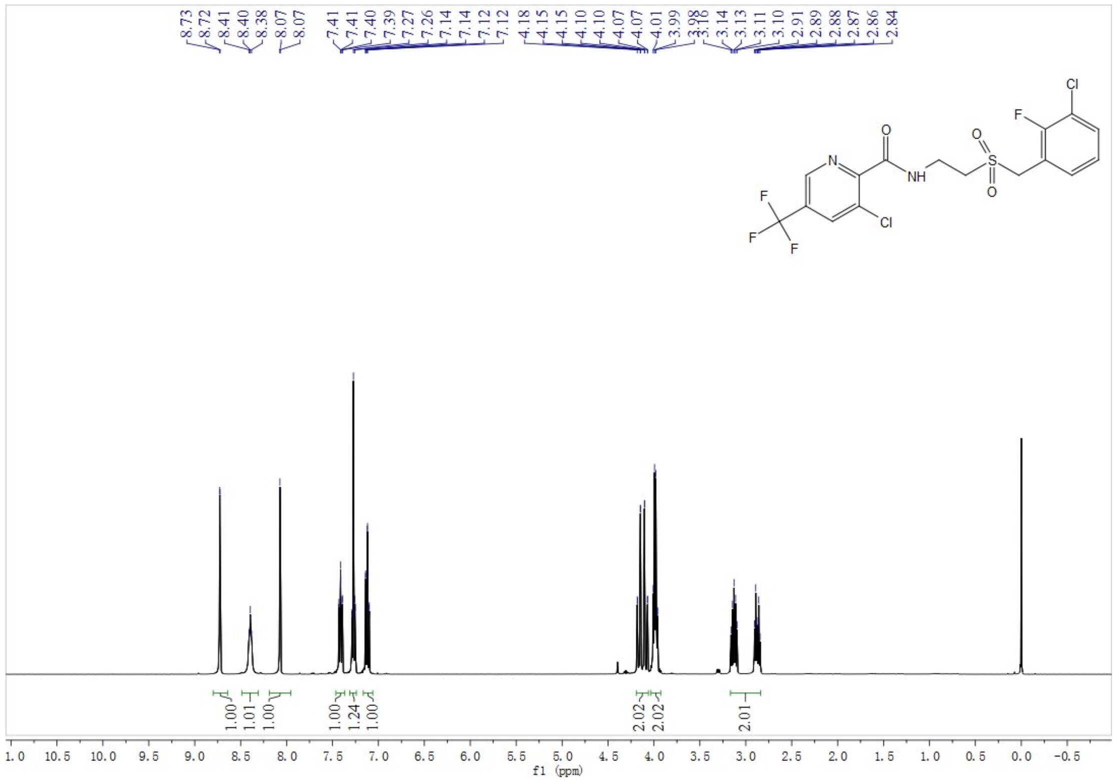

Fig. S97 ^{1}H NMR spectra of compound **F7**

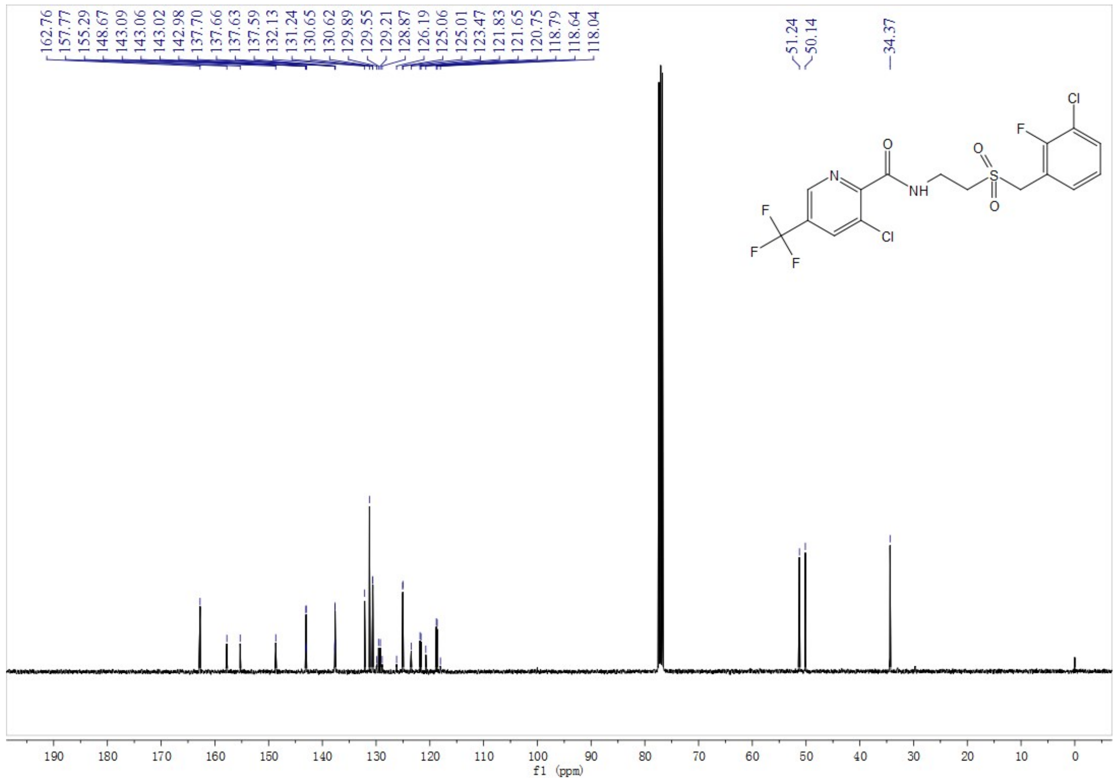

Fig. S98 ^{13}C NMR spectra of compound **F7**

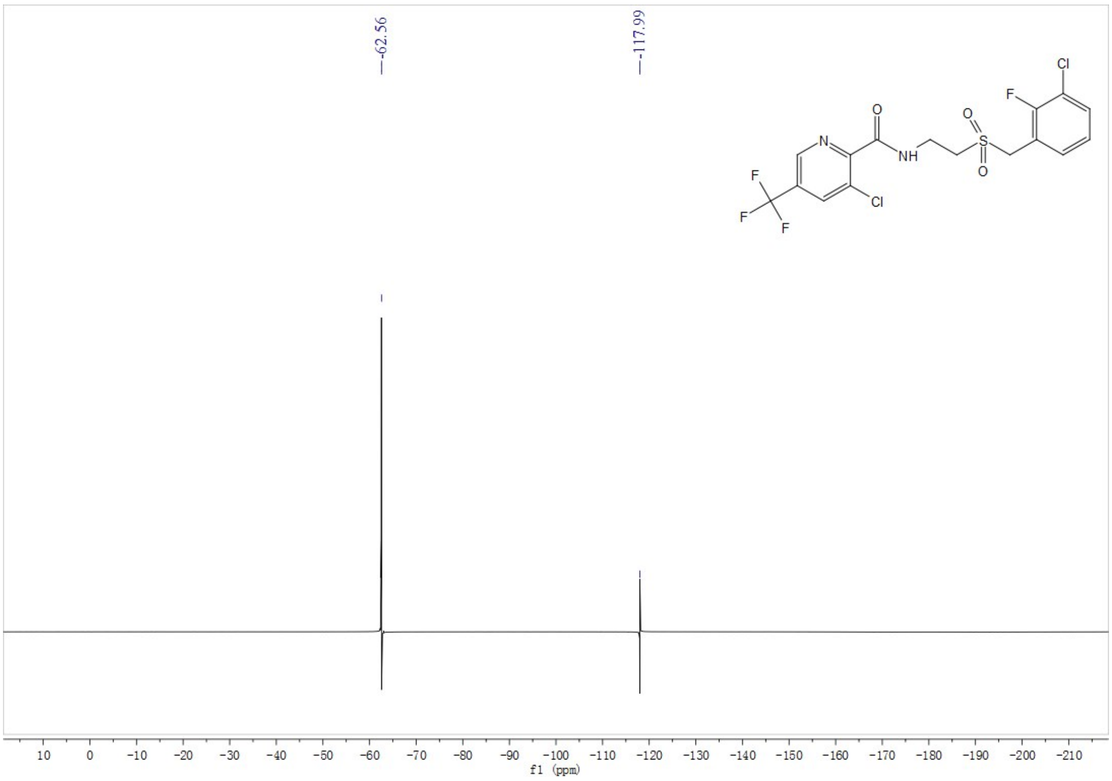

Fig. S99 ^{19}F NMR spectra of compound **F7**

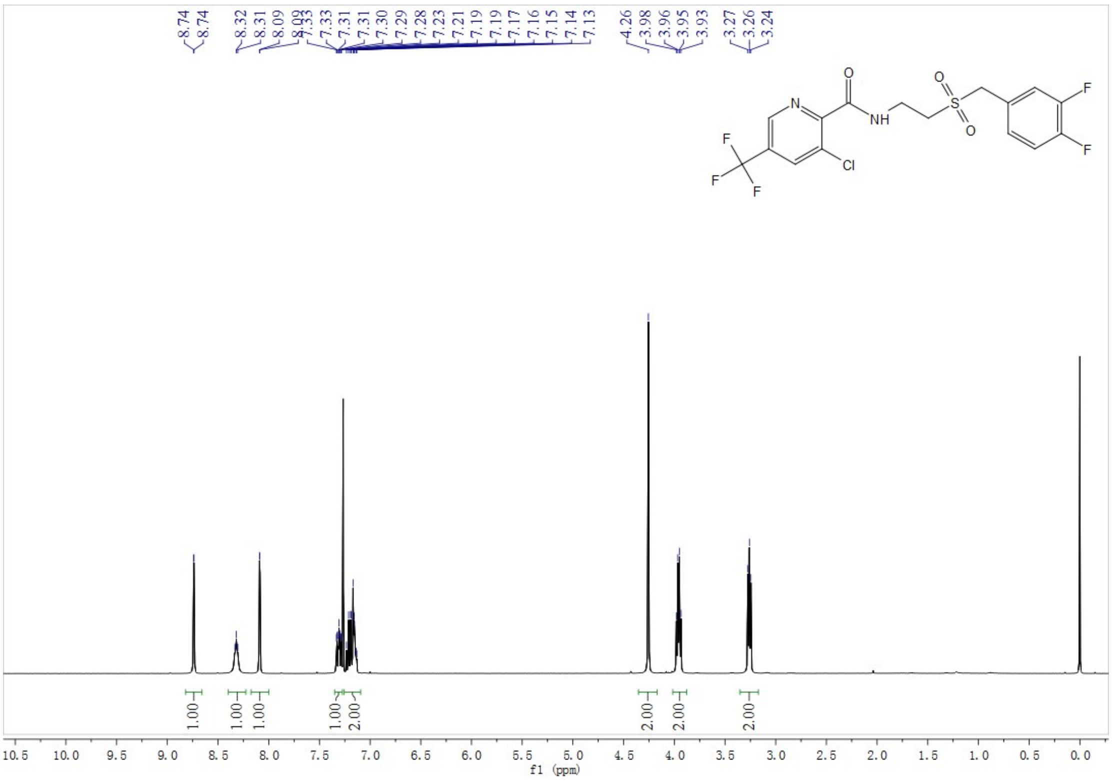

Fig. S100 ¹H NMR spectra of compound **F8**

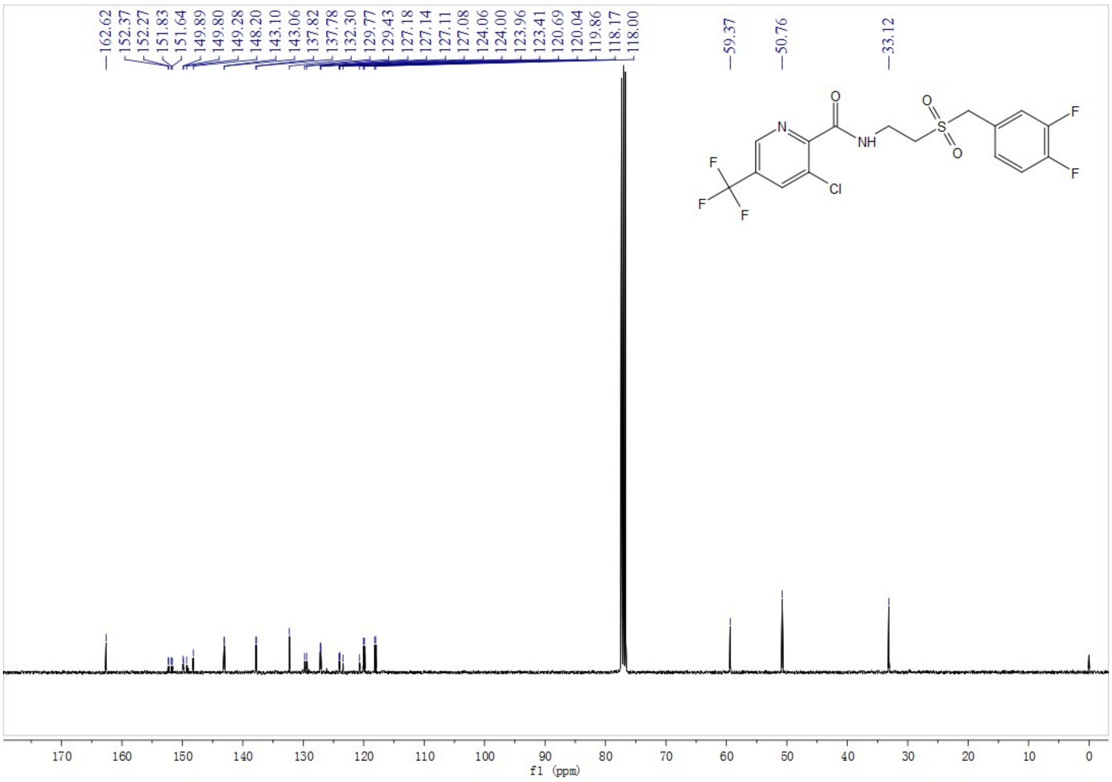

Fig. S101 ¹³C NMR spectra of compound **F8**

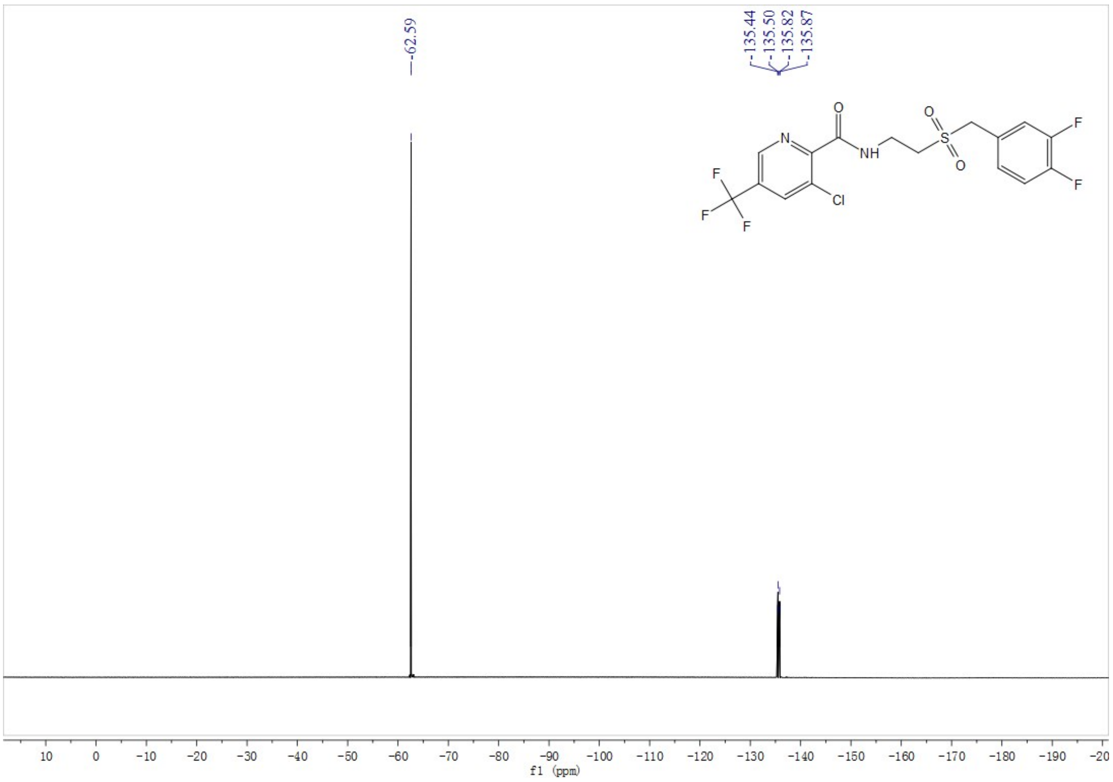

Fig. S102 ^{19}F NMR spectra of compound **F8**

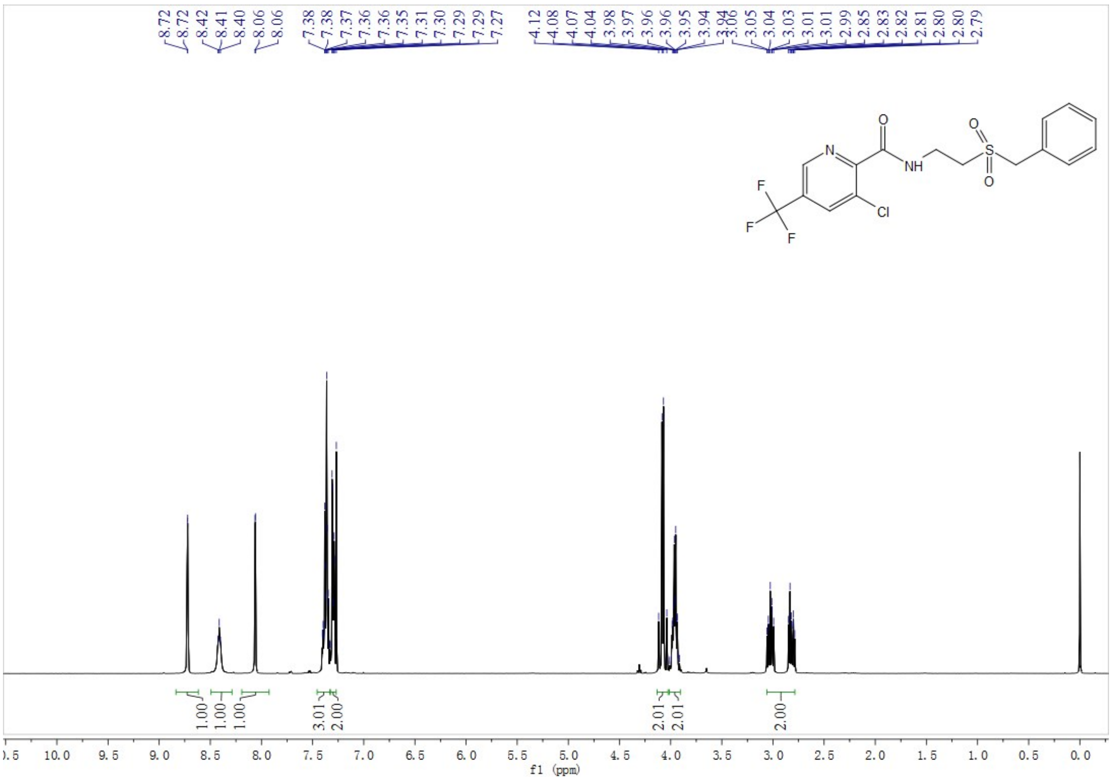

Fig. S103 ^{1}H NMR spectra of compound **F9**

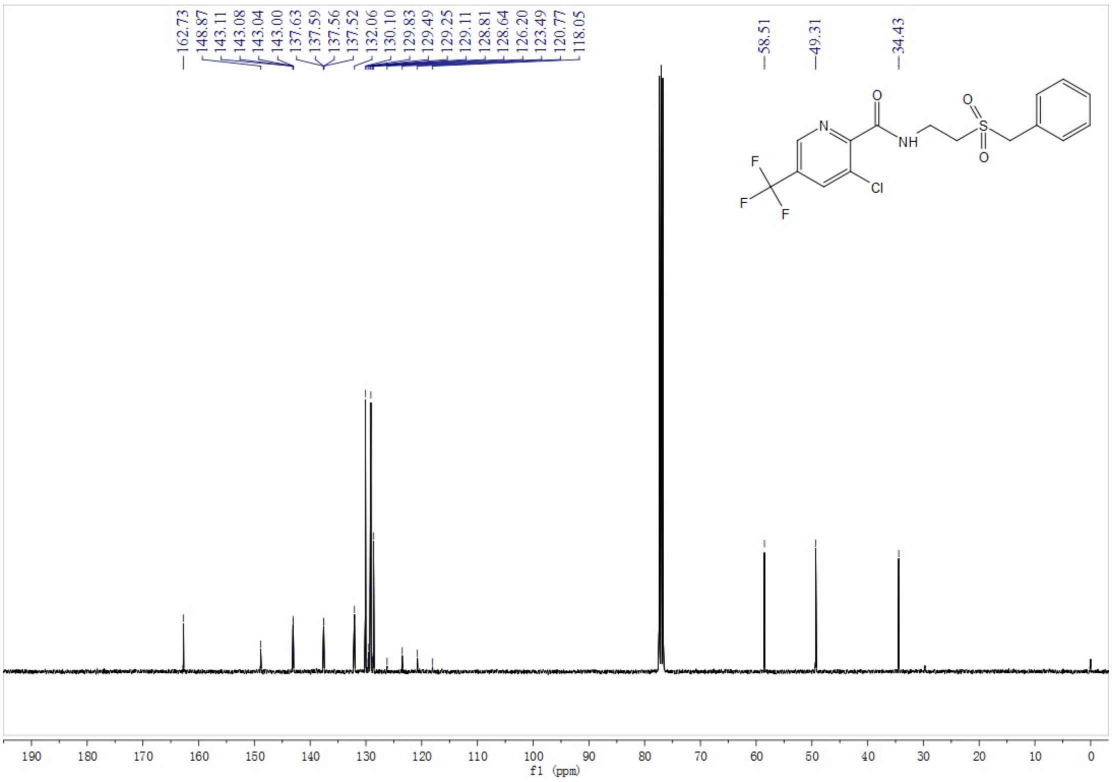

Fig. S104 ^{13}C NMR spectra of compound **F9**

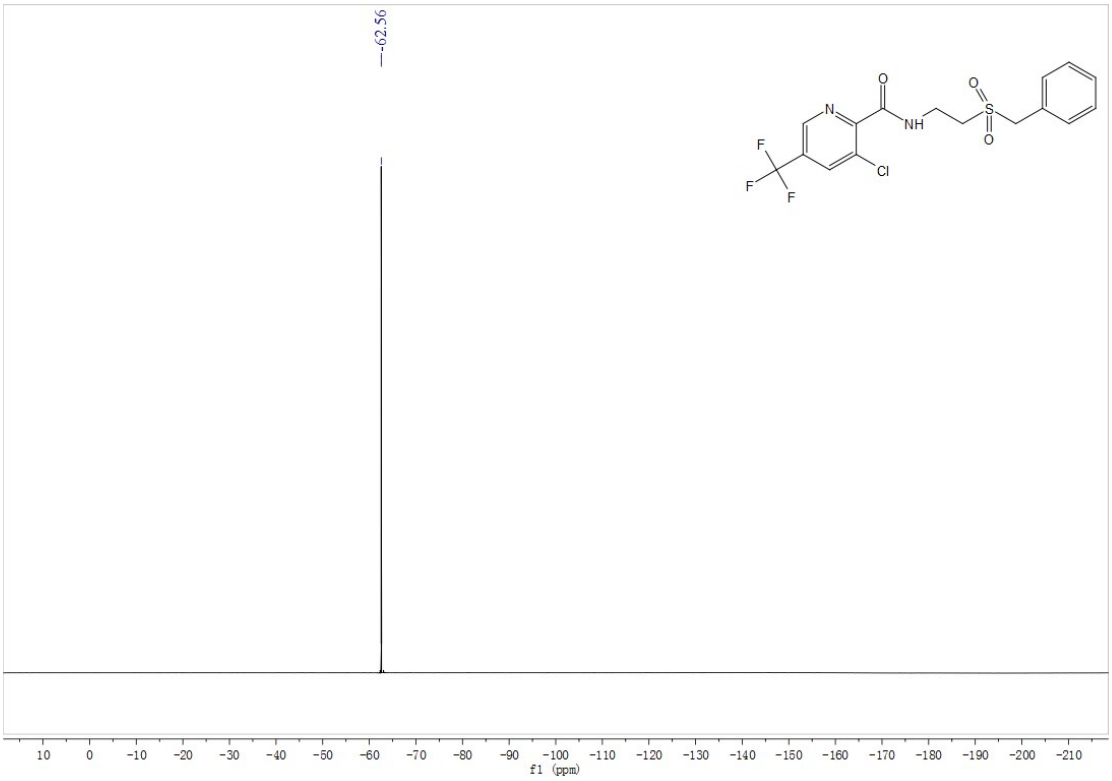

Fig. S105 ^{19}F NMR spectra of compound **F9**

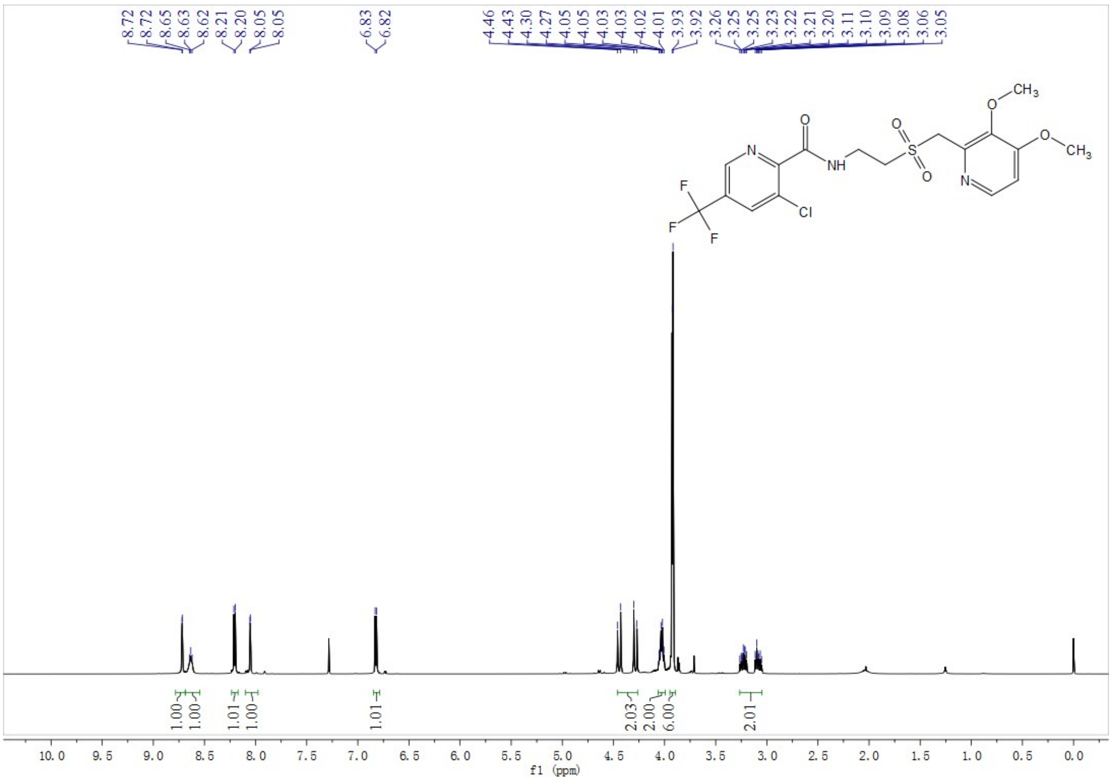

Fig. S106 ¹H NMR spectra of compound **F10**

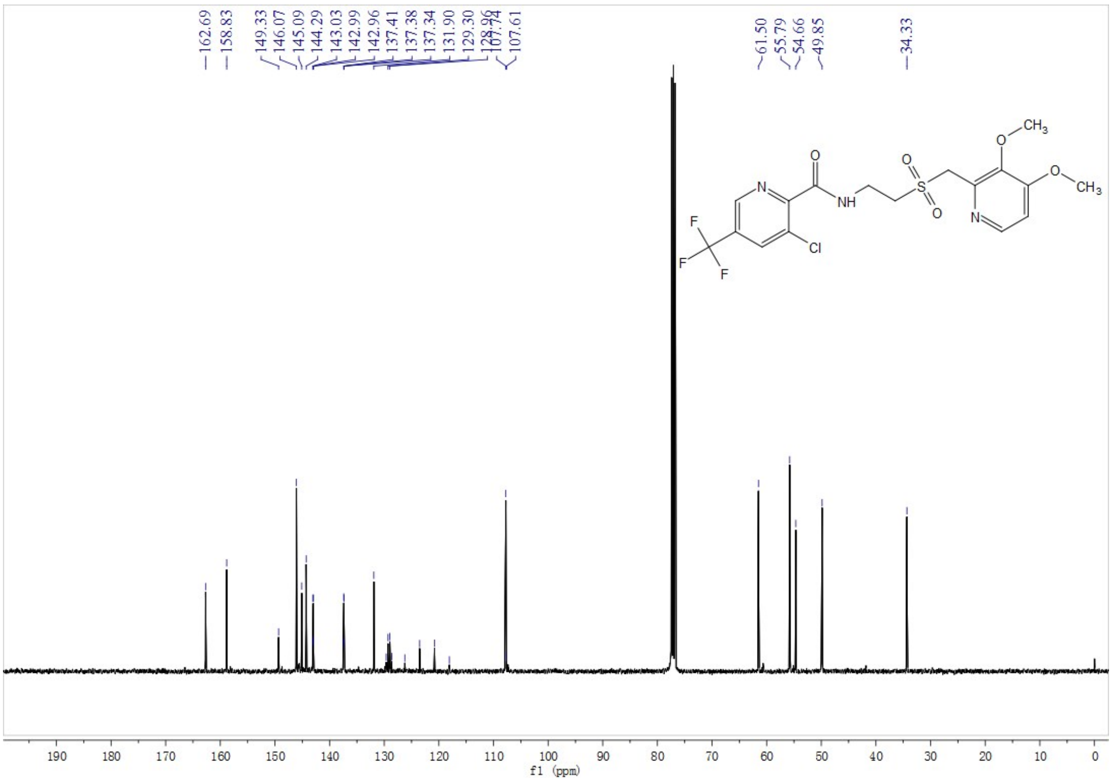

Fig. S107 ¹³C NMR spectra of compound **F10**

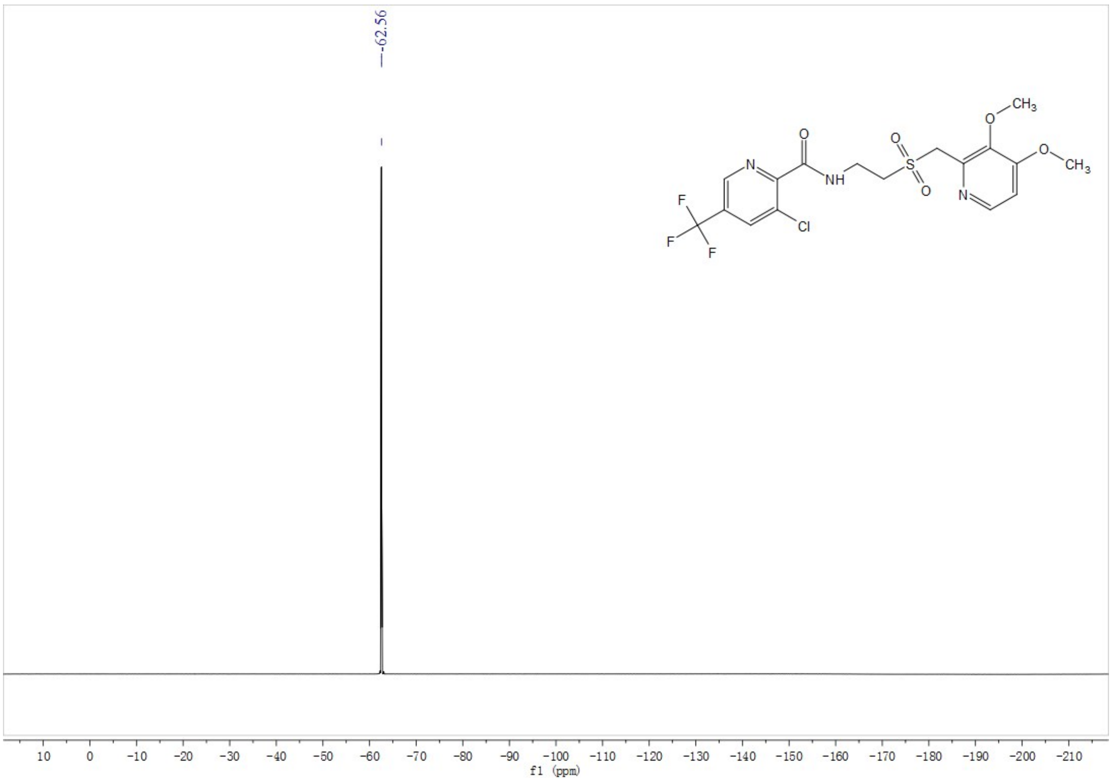

Fig. S108 ^{19}F NMR spectra of compound **F10**

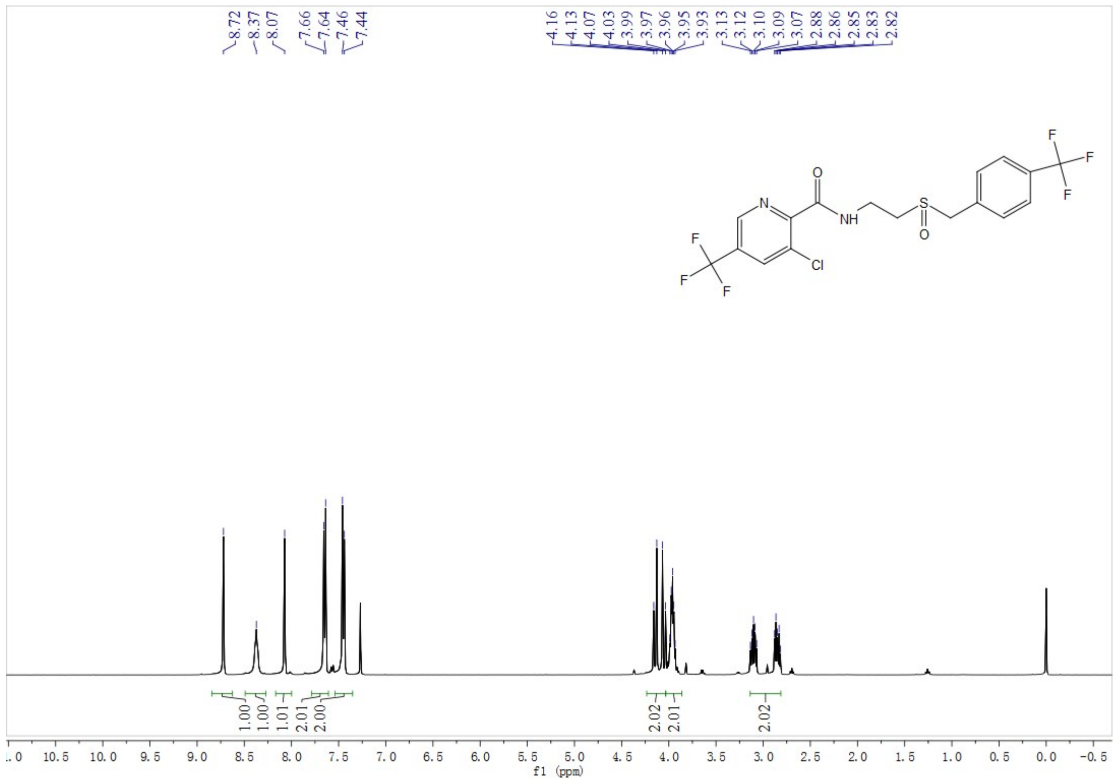

Fig. S109 ^{1}H NMR spectra of compound **G1**

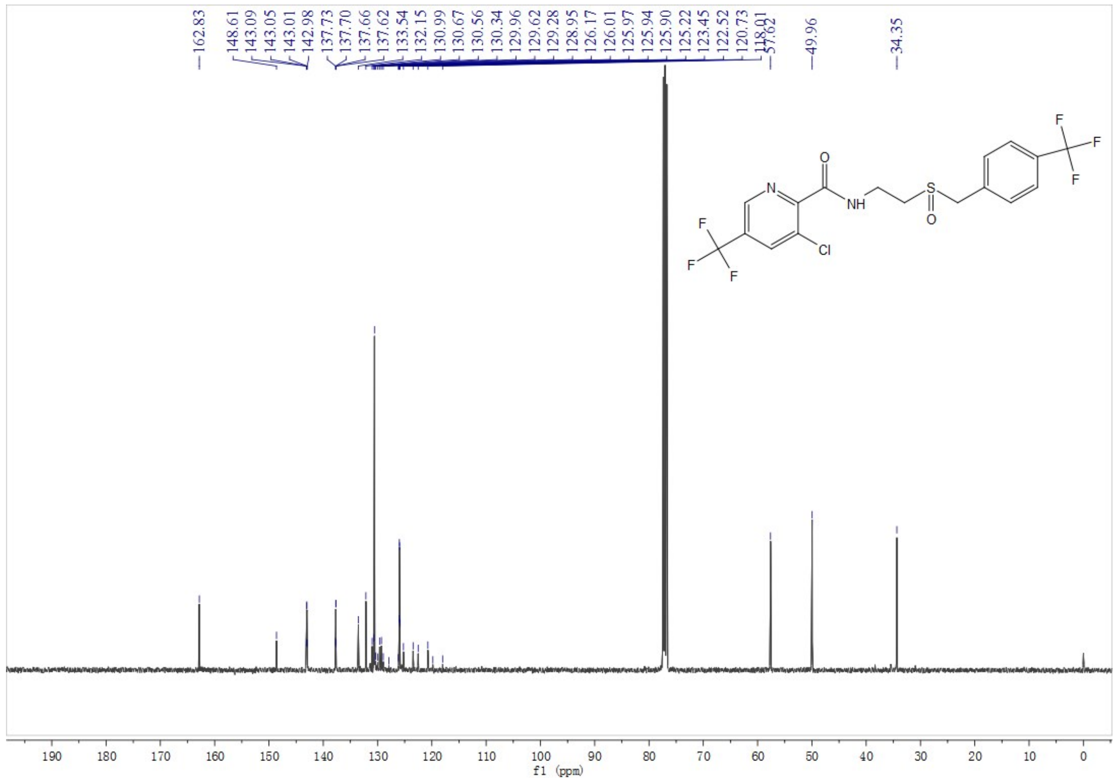

Fig. S110 ^{13}C NMR spectra of compound **G1**

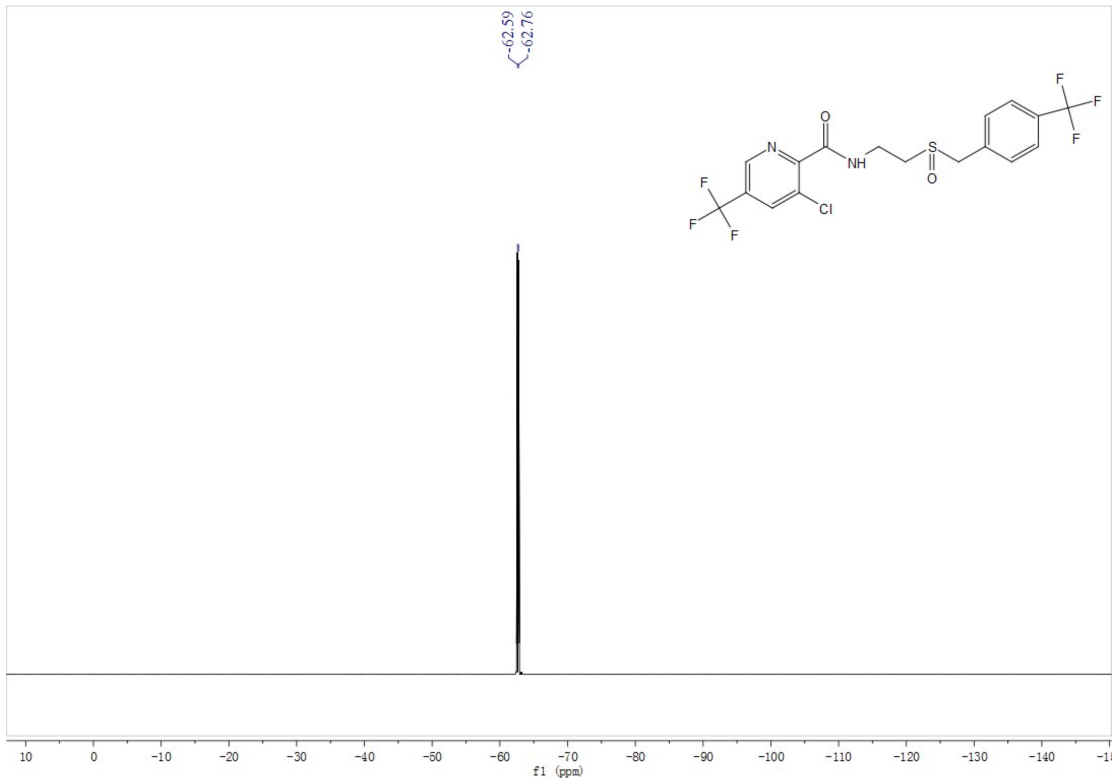

Fig. S111 ^{19}F NMR spectra of compound **G1**

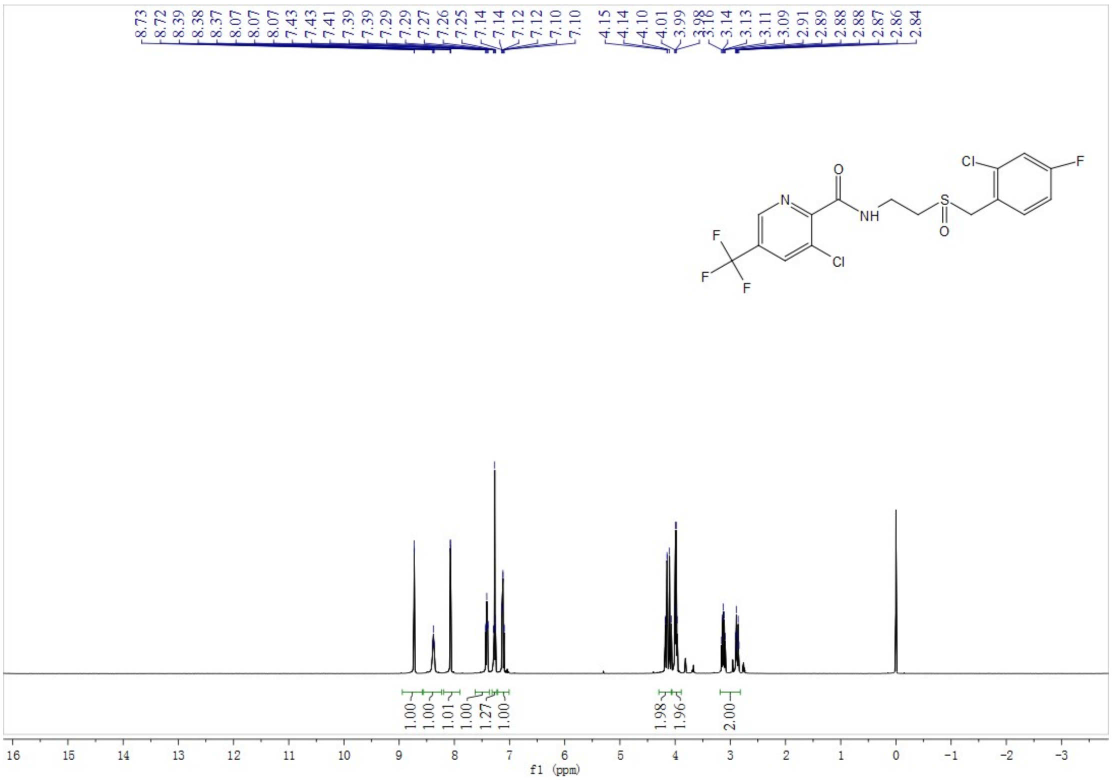

Fig. S112 ^{1}H NMR spectra of compound **G2**

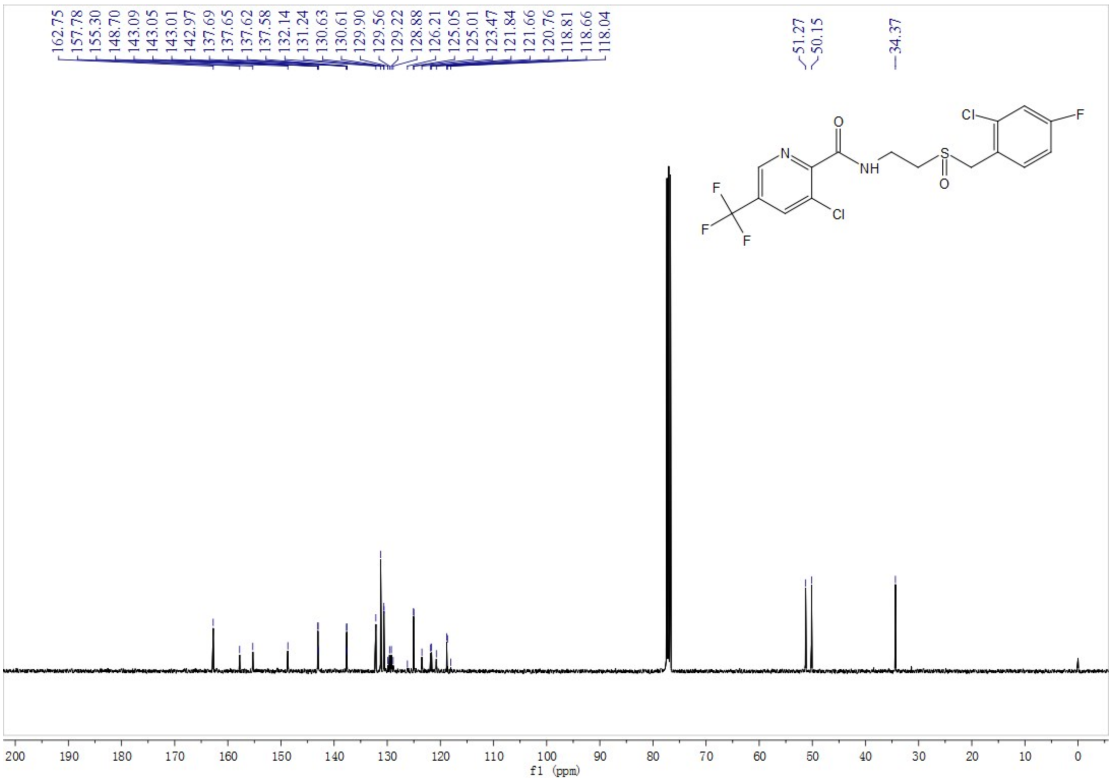

Fig. S113 ^{13}C NMR spectra of compound **G2**

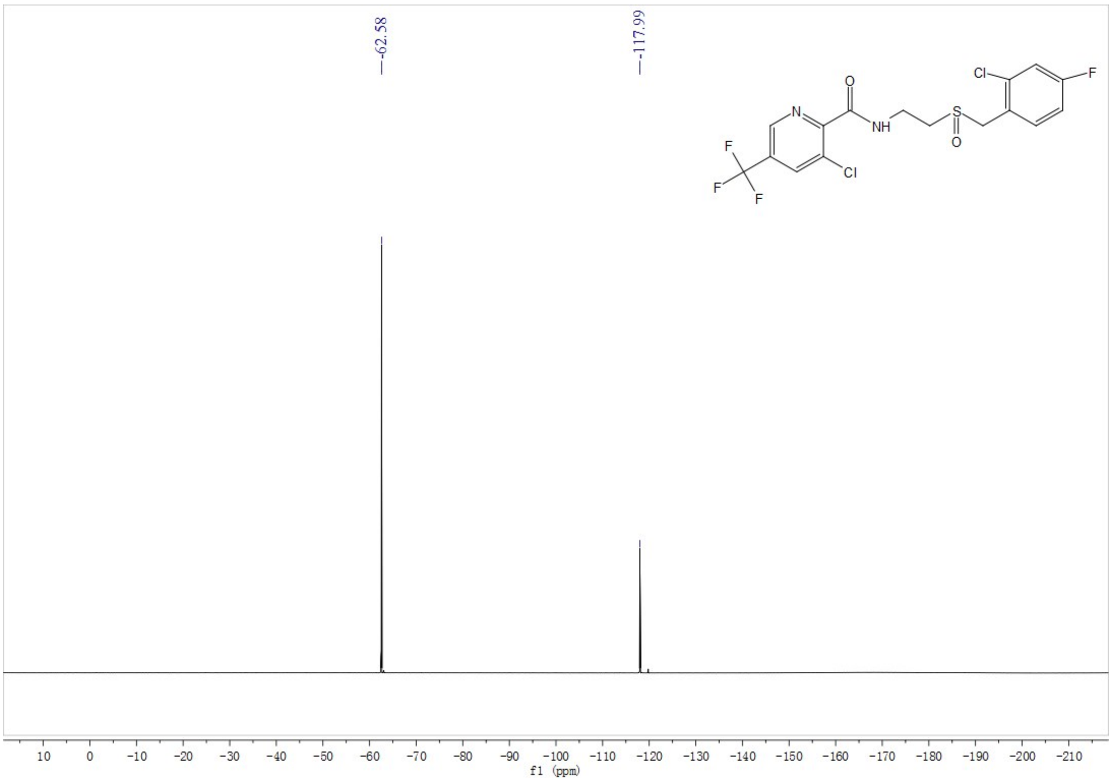

Fig. S114 ^{19}F NMR spectra of compound **G2**

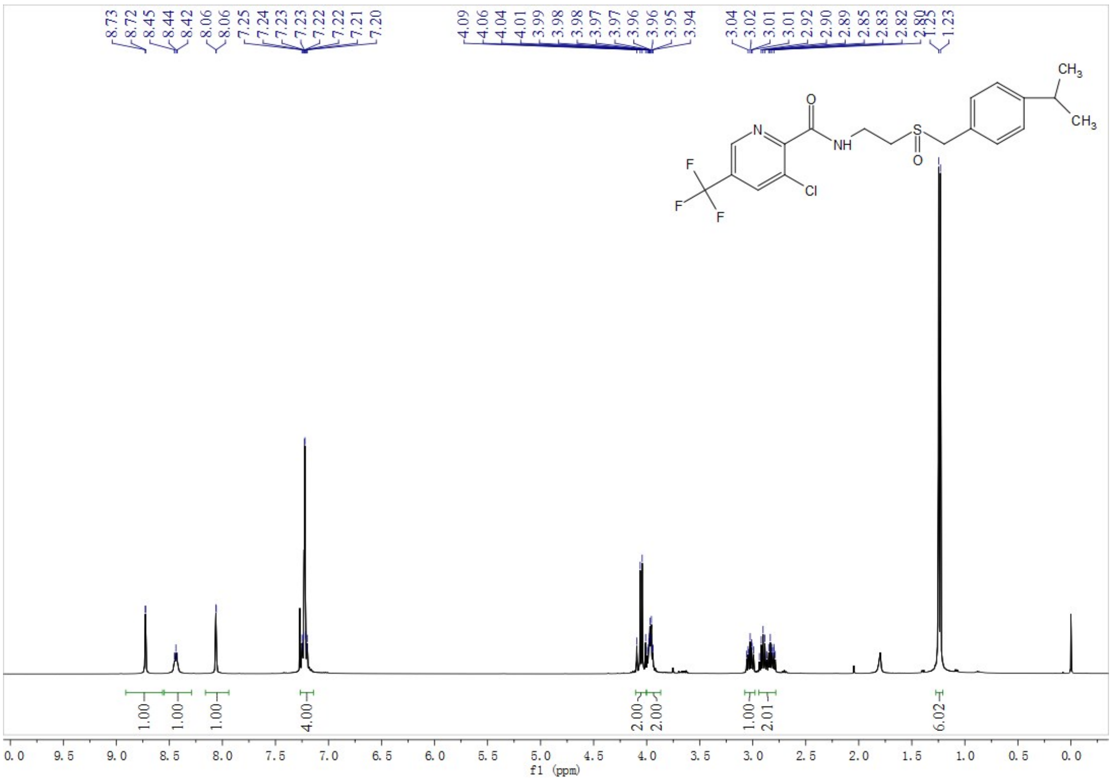

Fig. S115 ^{1}H NMR spectra of compound **G3**

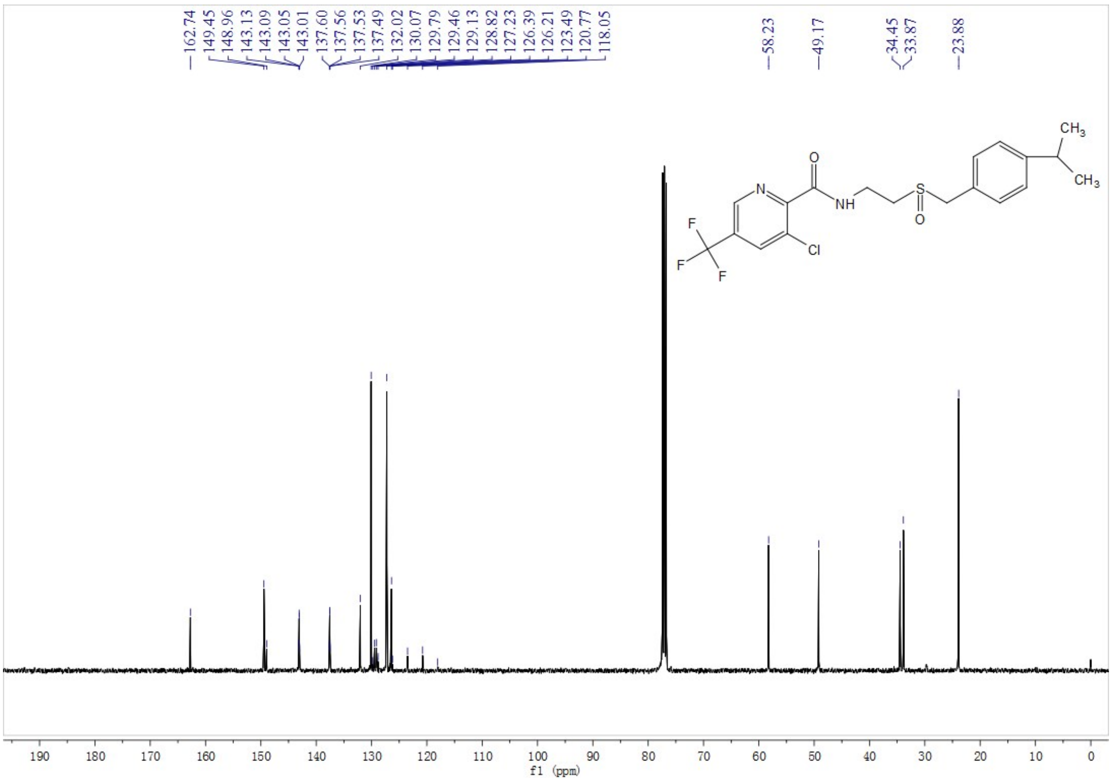

Fig. S116 ^{13}C NMR spectra of compound **G3**

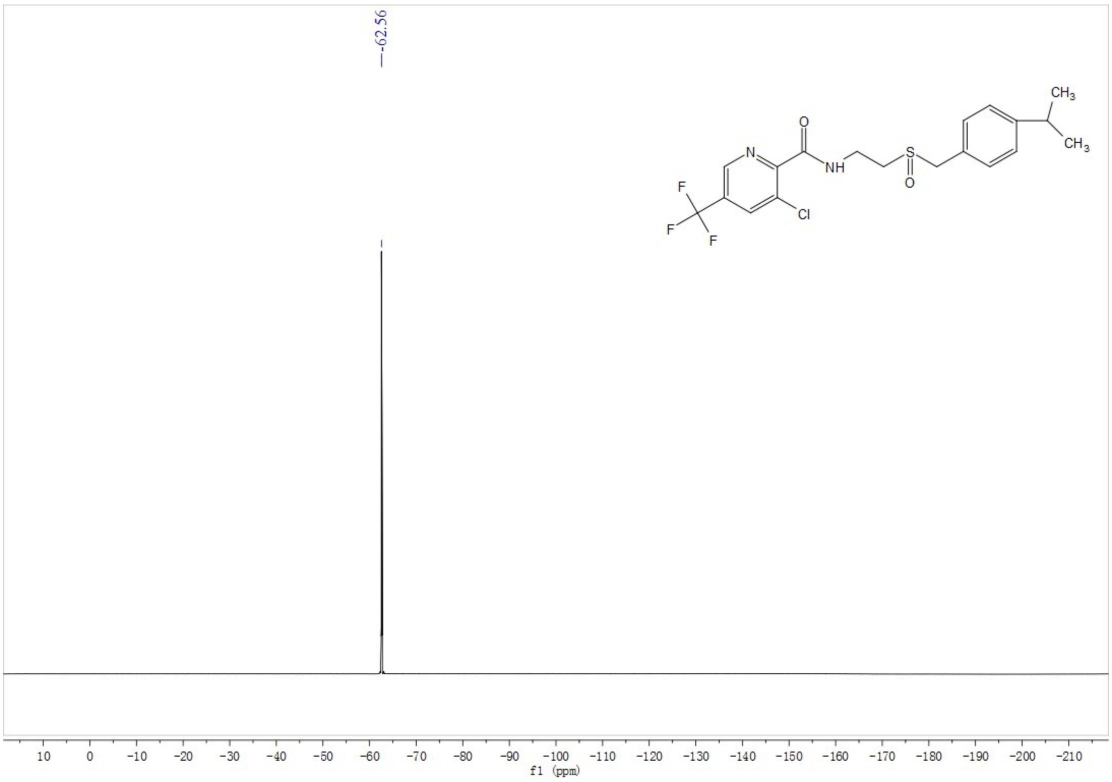

Fig. S117 ^{19}F NMR spectra of compound **G3**

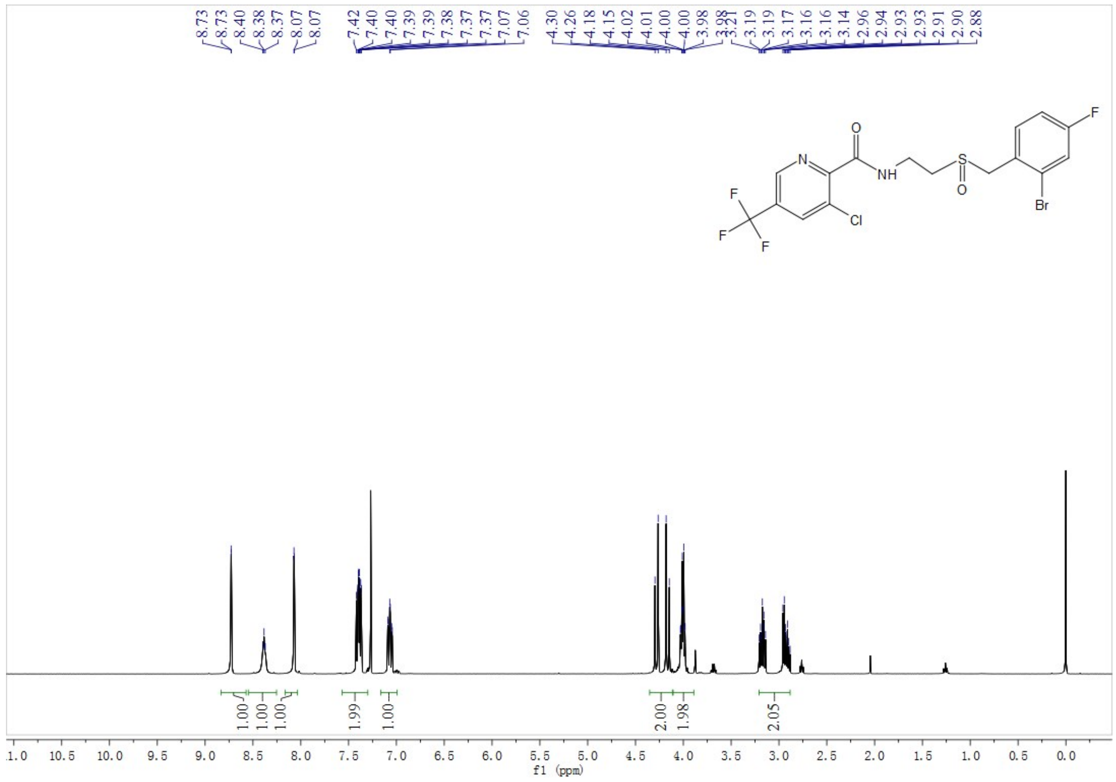

Fig. S118 ^{1}H NMR spectra of compound **G4**

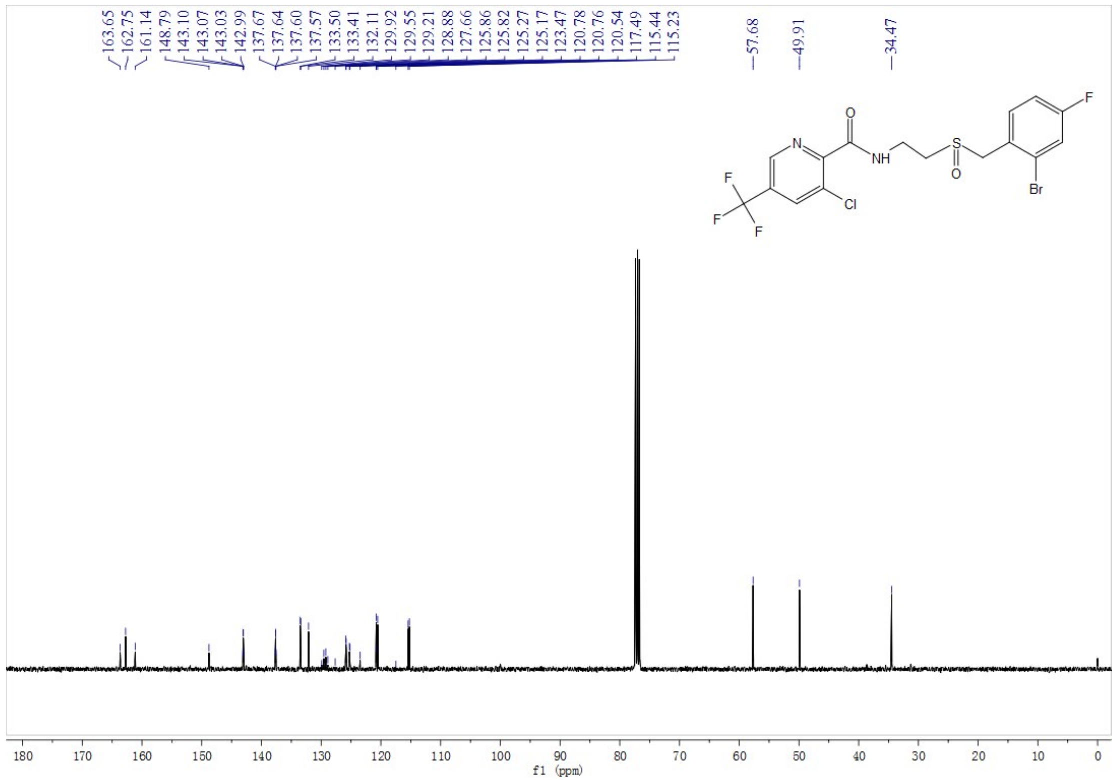

Fig. S119 ^{13}C NMR spectra of compound **G4**

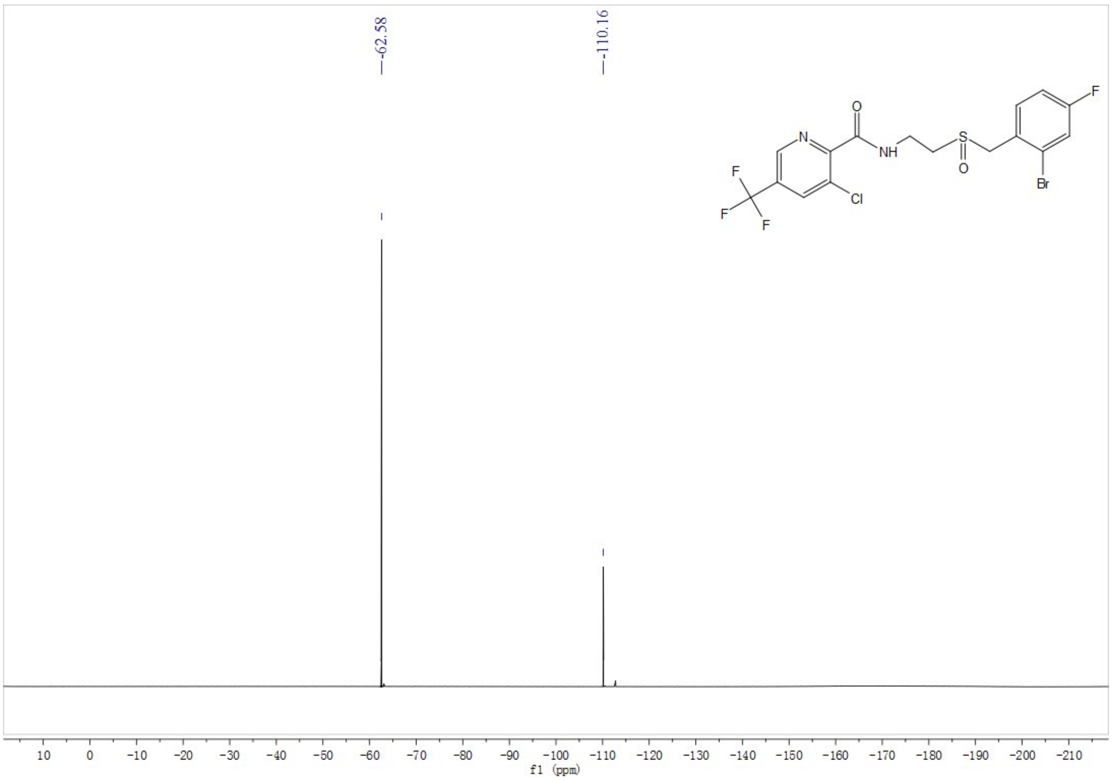

Fig. S120 ^{19}F NMR spectra of compound **G4**

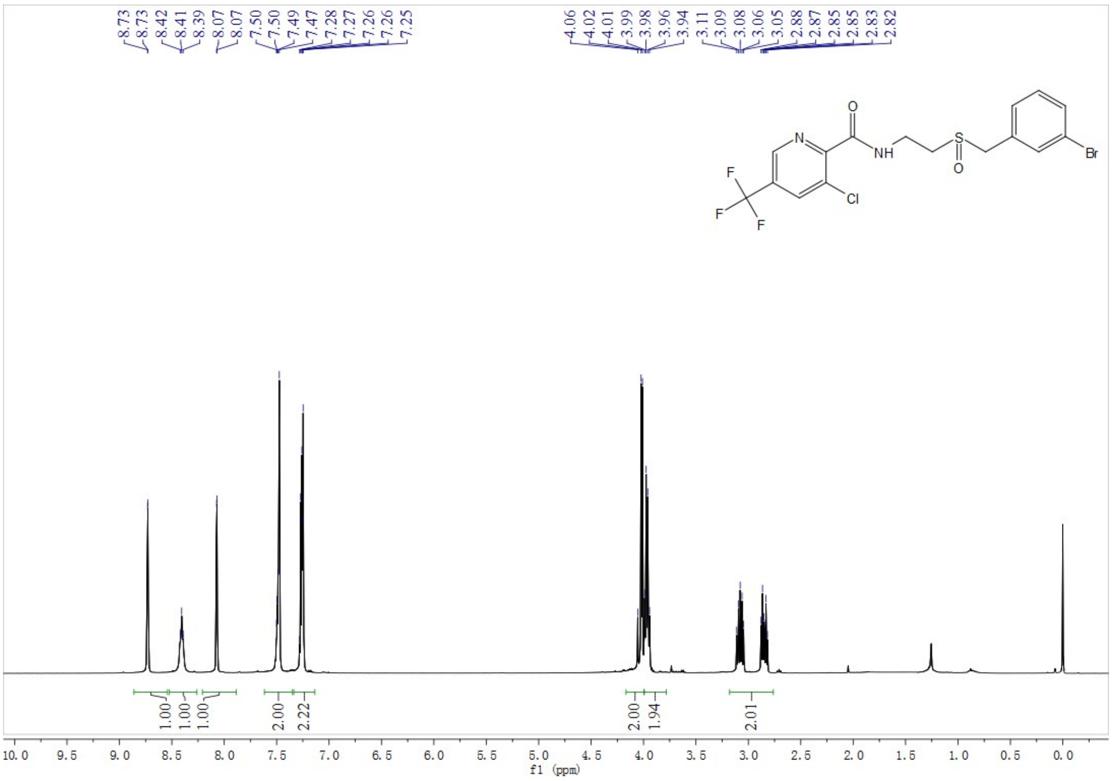

Fig. S121 ^{1}H NMR spectra of compound **G5**

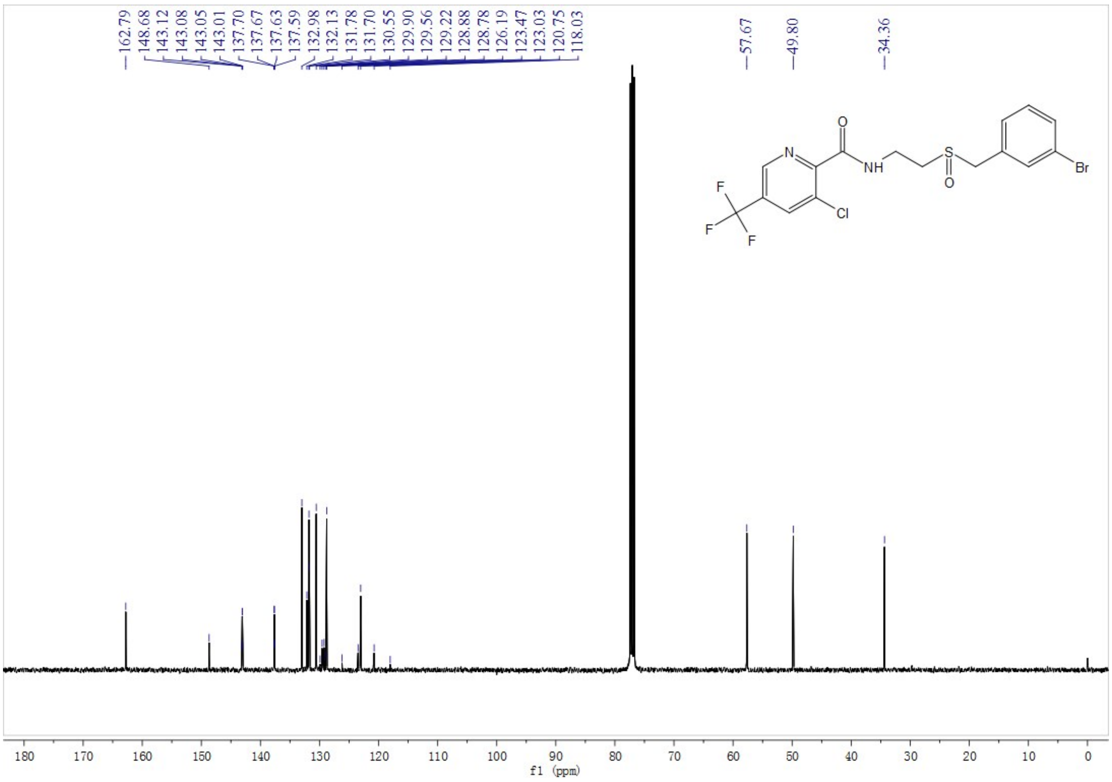

Fig. S122 ^{13}C NMR spectra of compound **G5**

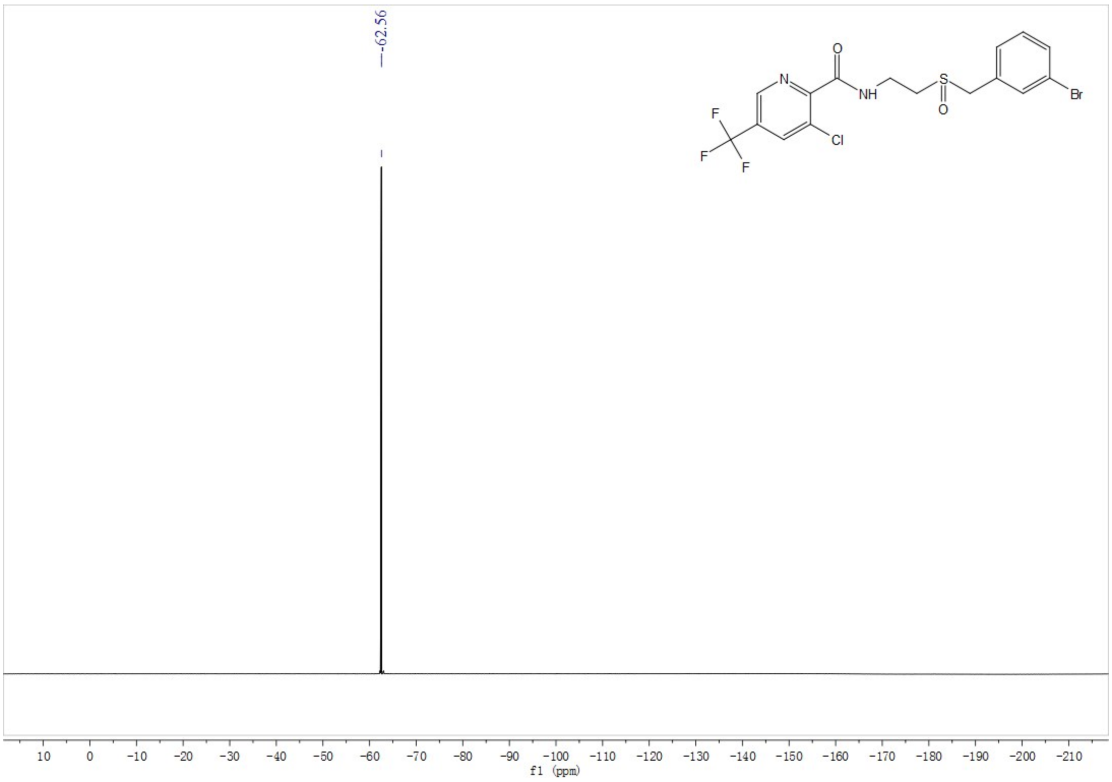

Fig. S123 ^{19}F NMR spectra of compound **G5**

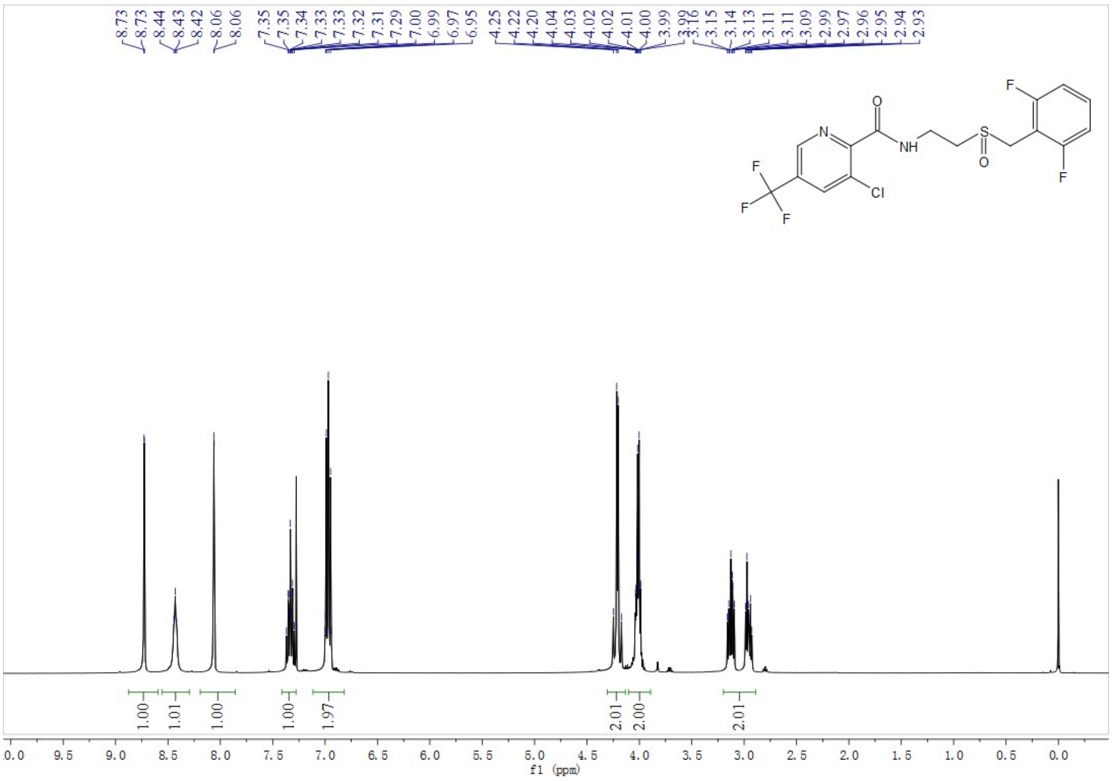

Fig. S124 ^{1}H NMR spectra of compound **G6**

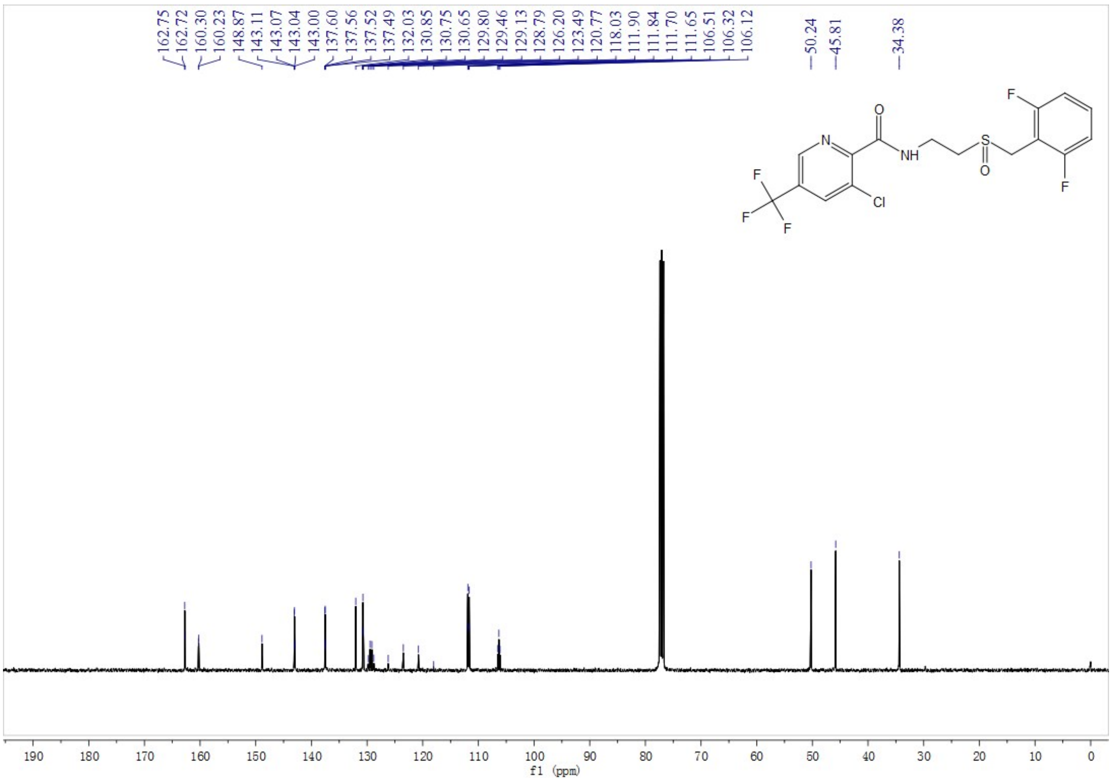

Fig. S125 ^{13}C NMR spectra of compound **G6**

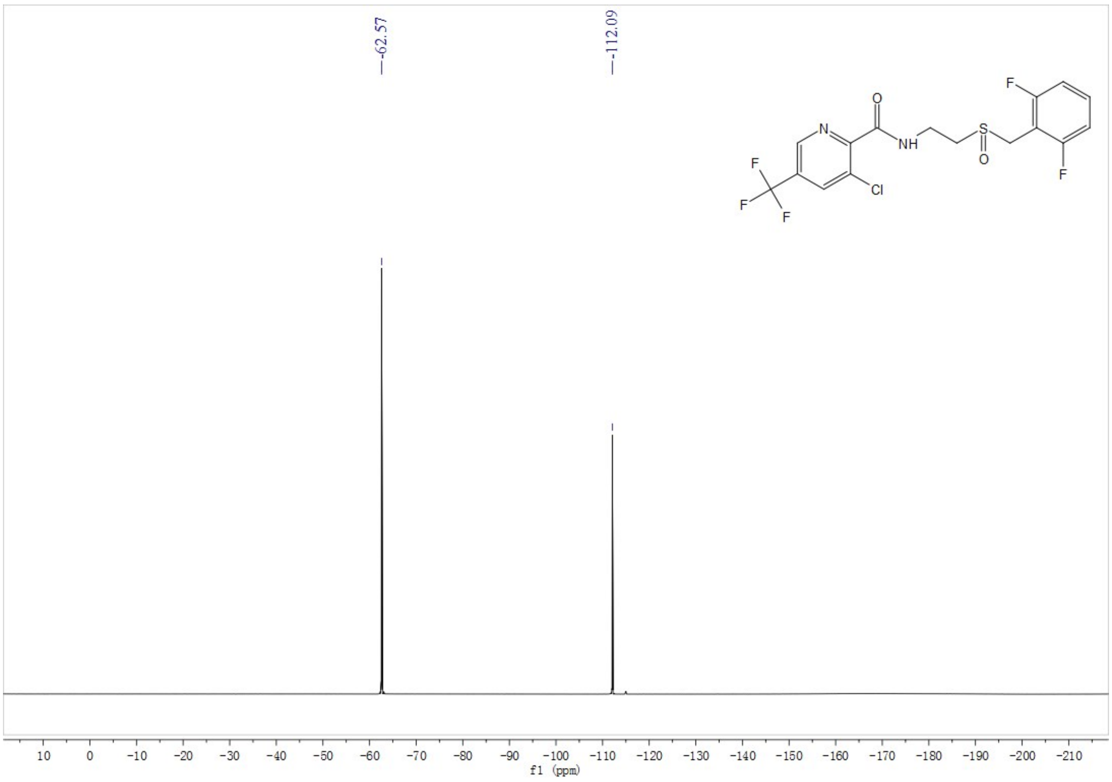

Fig. S126 ^{19}F NMR spectra of compound **G6**

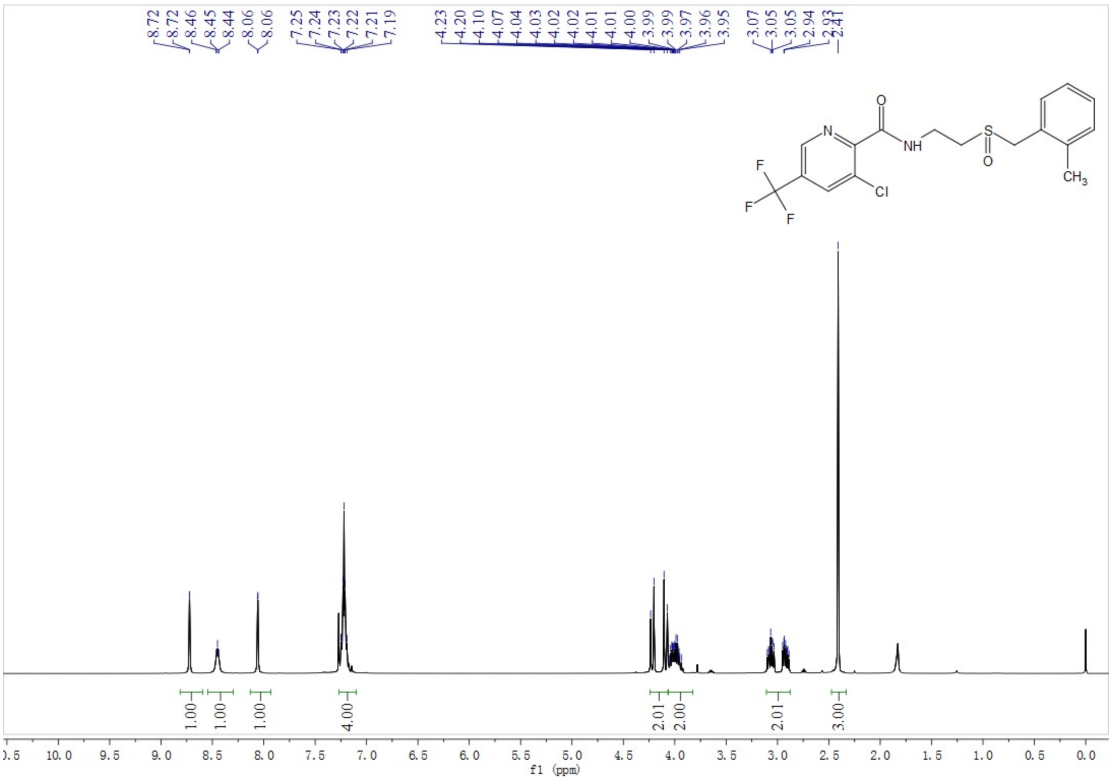

Fig. S127 ^{1}H NMR spectra of compound **G7**

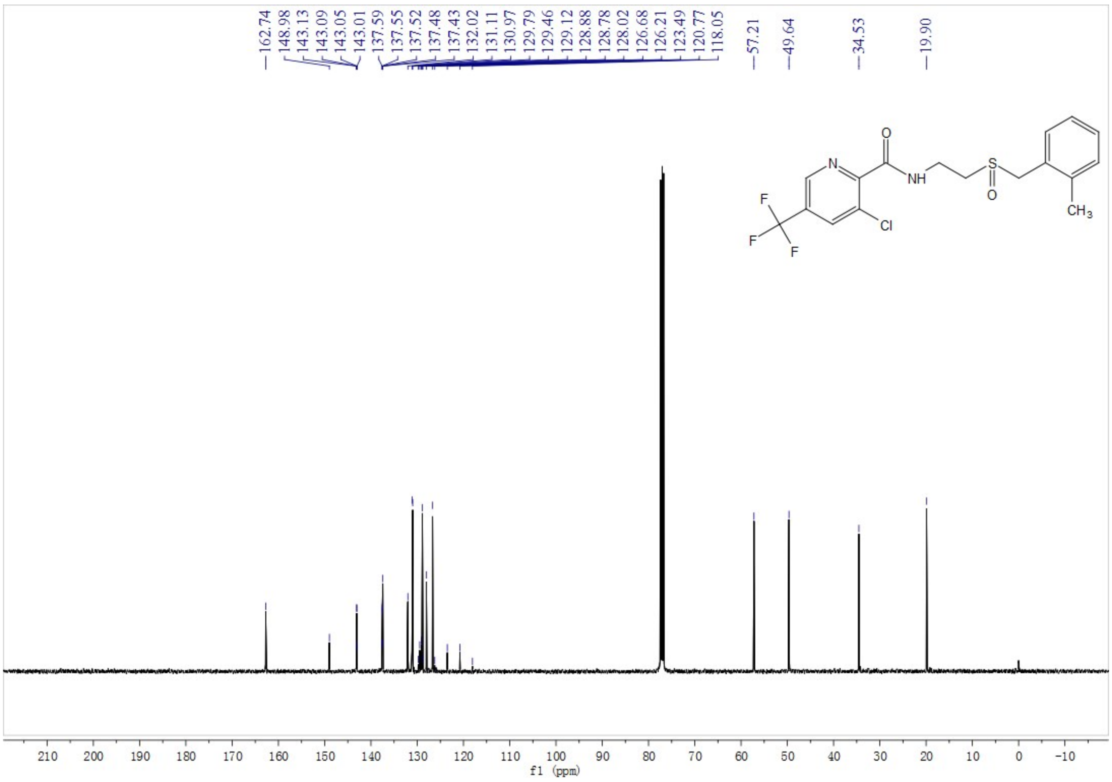

Fig. S128 ^{13}C NMR spectra of compound **G7**

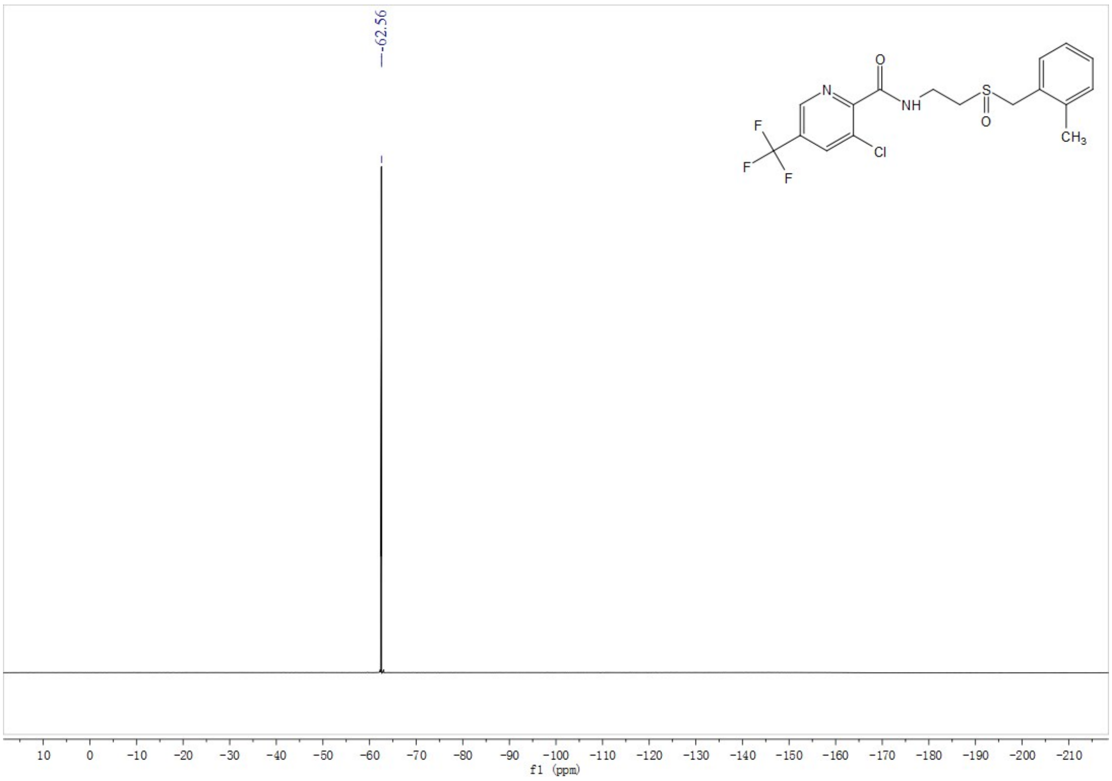

Fig. S129 ^{19}F NMR spectra of compound **G7**

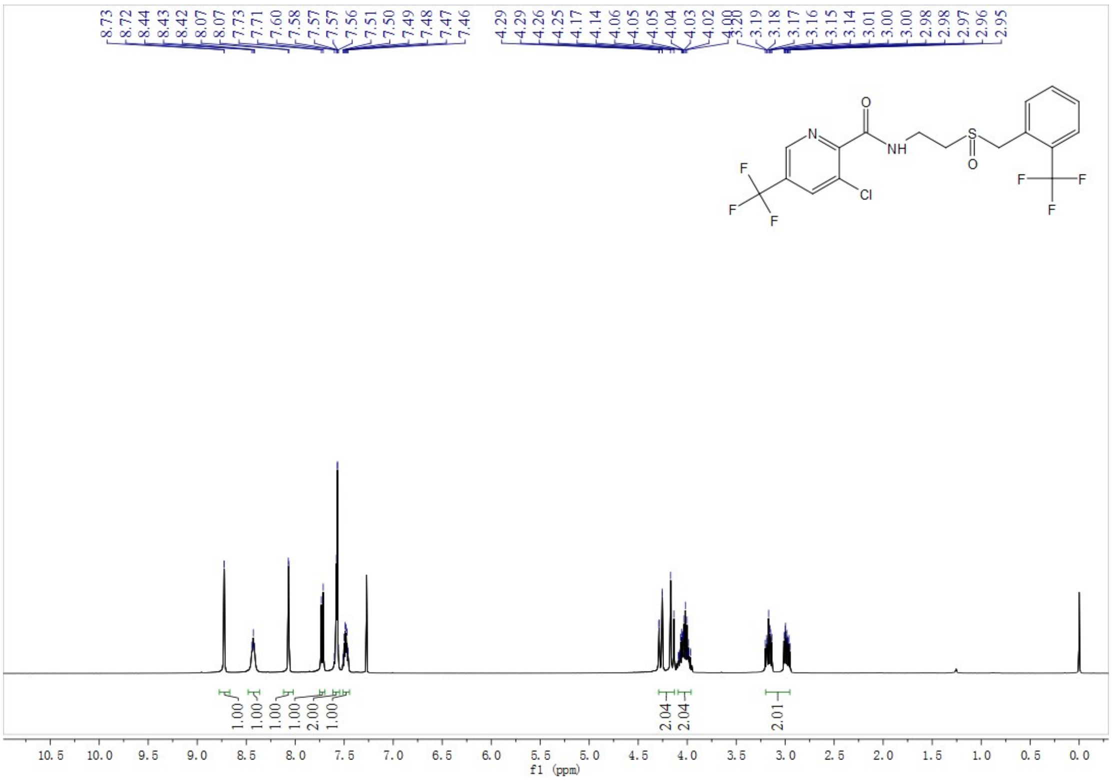

Fig. S130 ^{1}H NMR spectra of compound **G8**

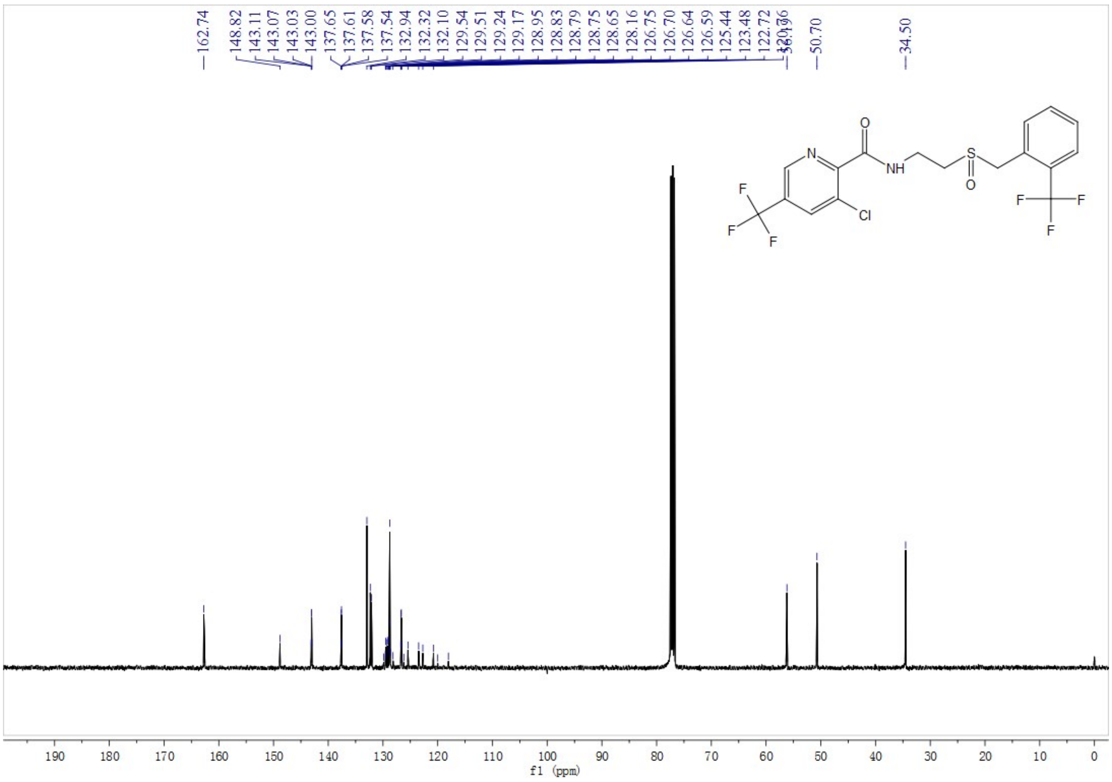

Fig. S131 ^{13}C NMR spectra of compound **G8**

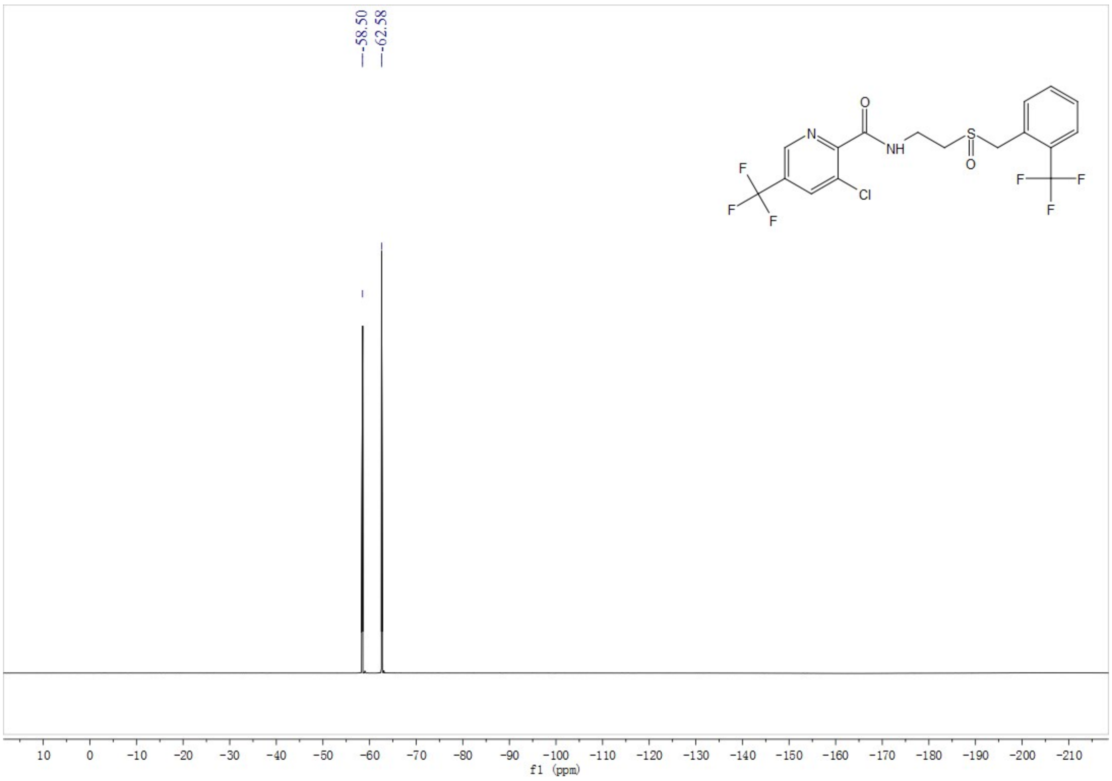

Fig. S132 ^{19}F NMR spectra of compound **G8**

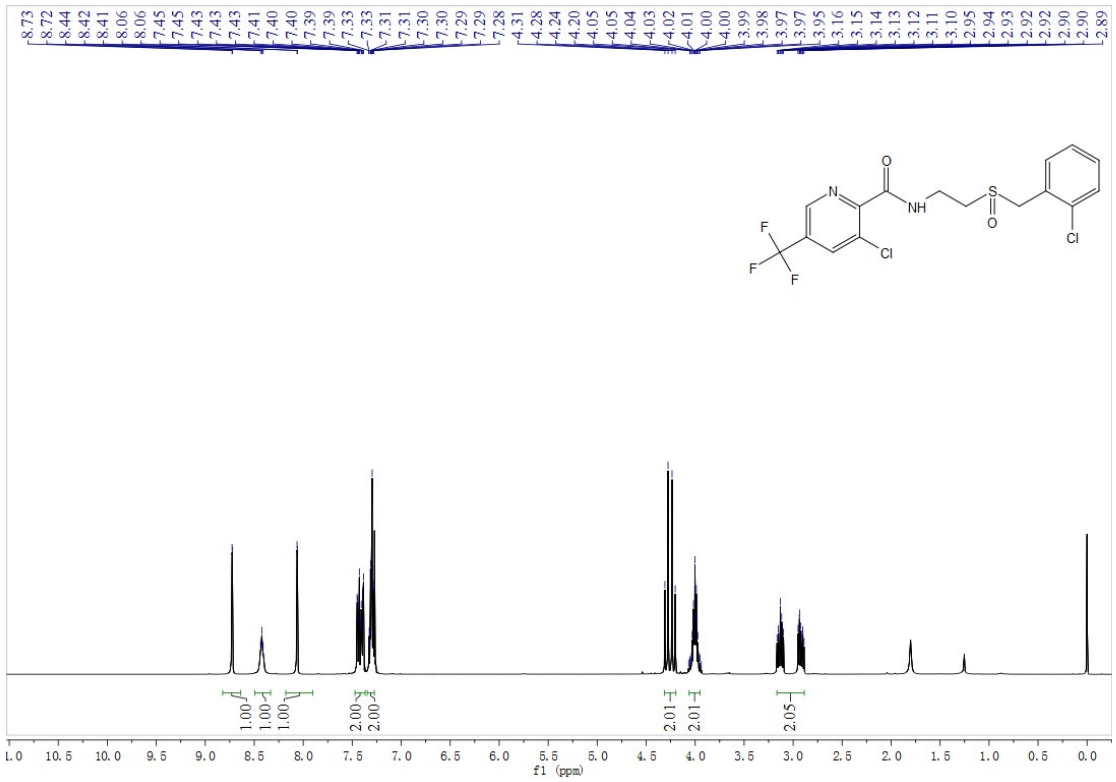

Fig. S133 ^{1}H NMR spectra of compound **G9**

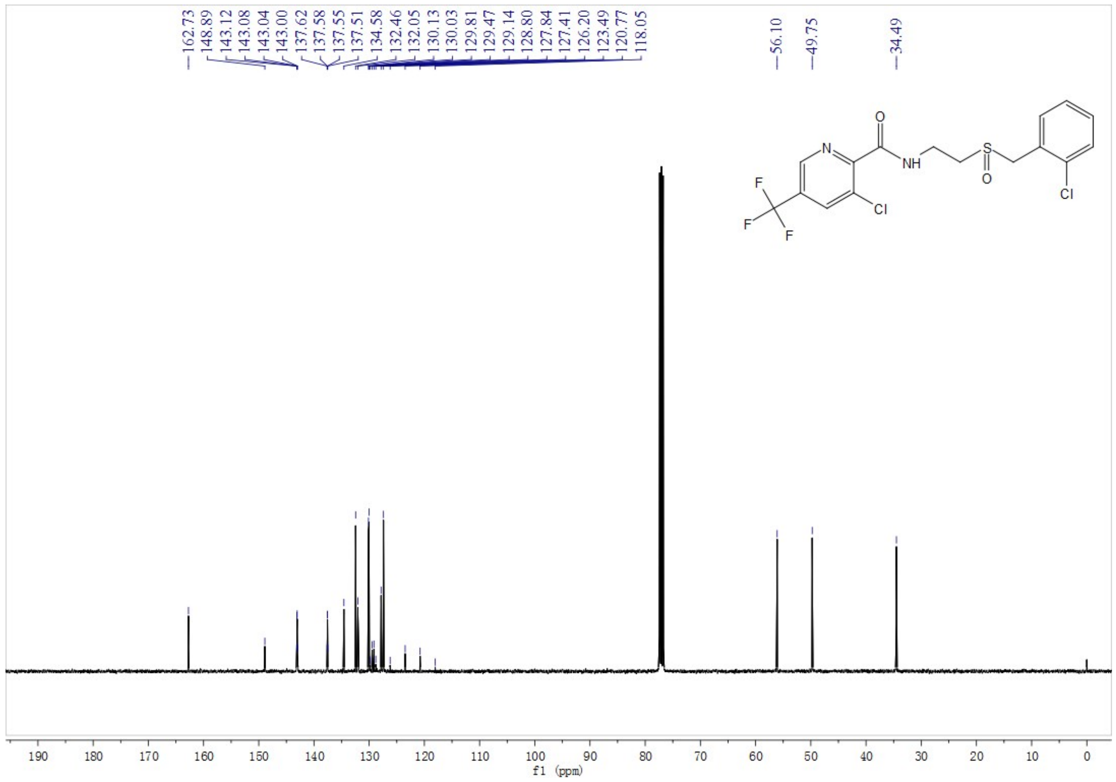

Fig. S134 ^{13}C NMR spectra of compound **G9**

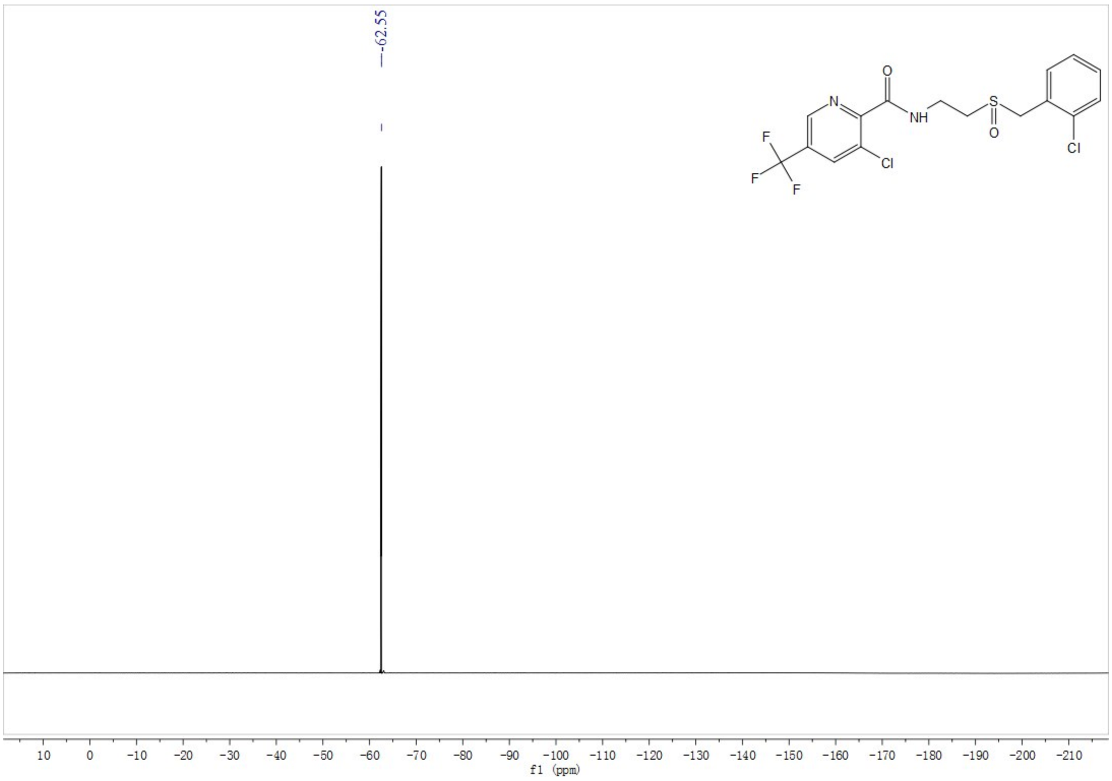

Fig. S135 ^{19}F NMR spectra of compound **G9**

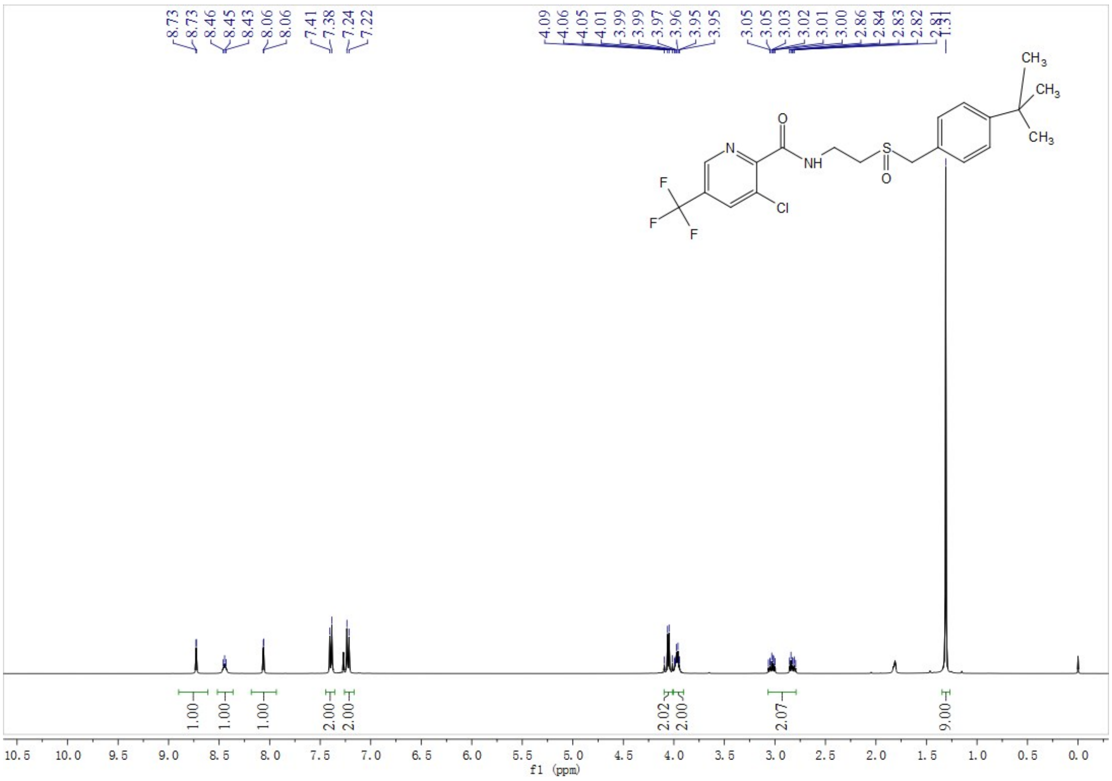

Fig. S136 ^{1}H NMR spectra of compound **G10**

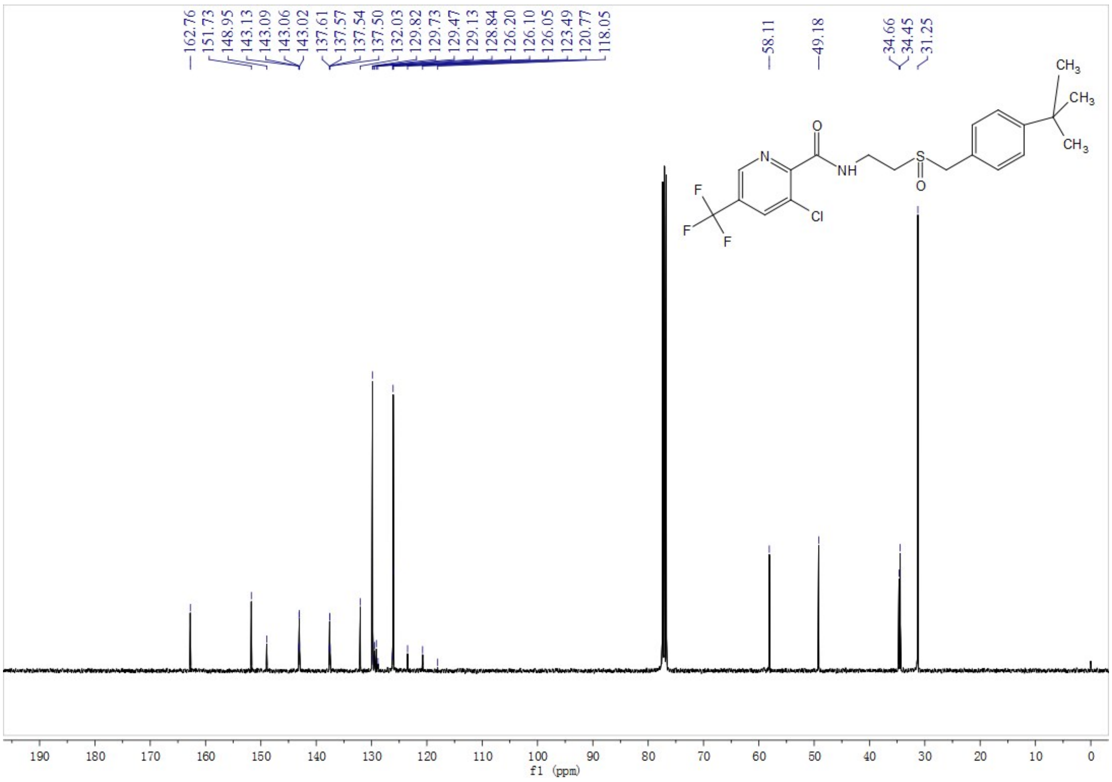

Fig. S137 ^{13}C NMR spectra of compound **G10**

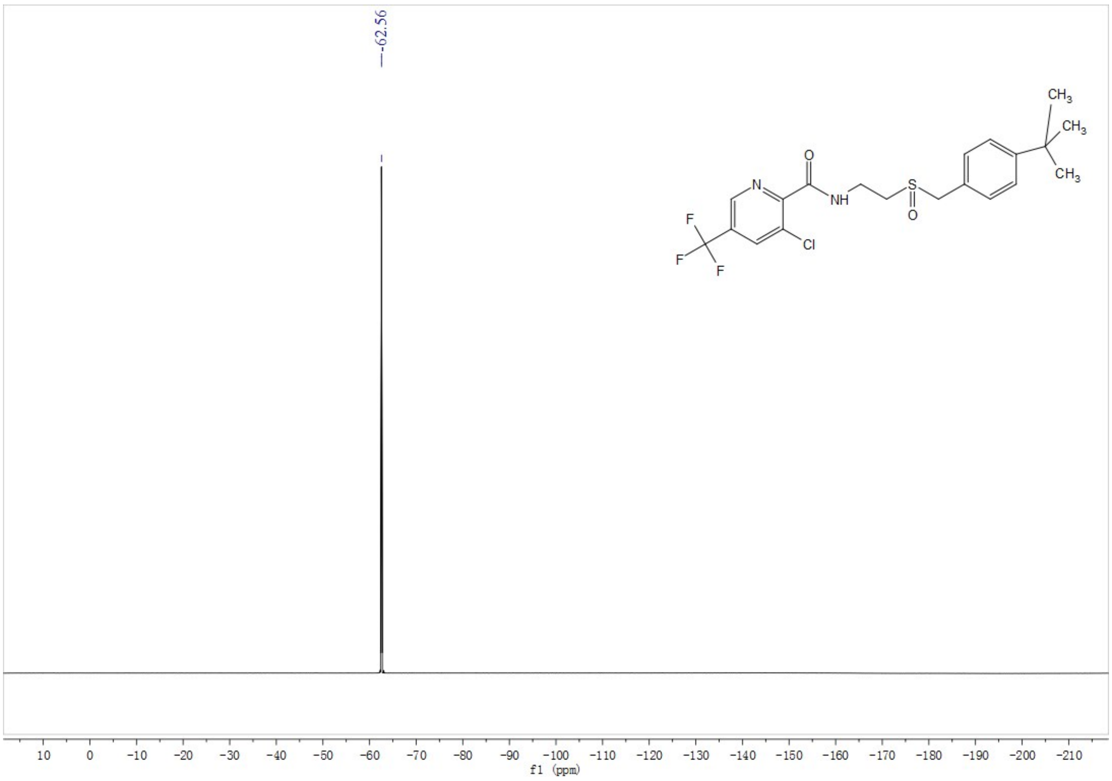

Fig. S138 ^{19}F NMR spectra of compound **G10**

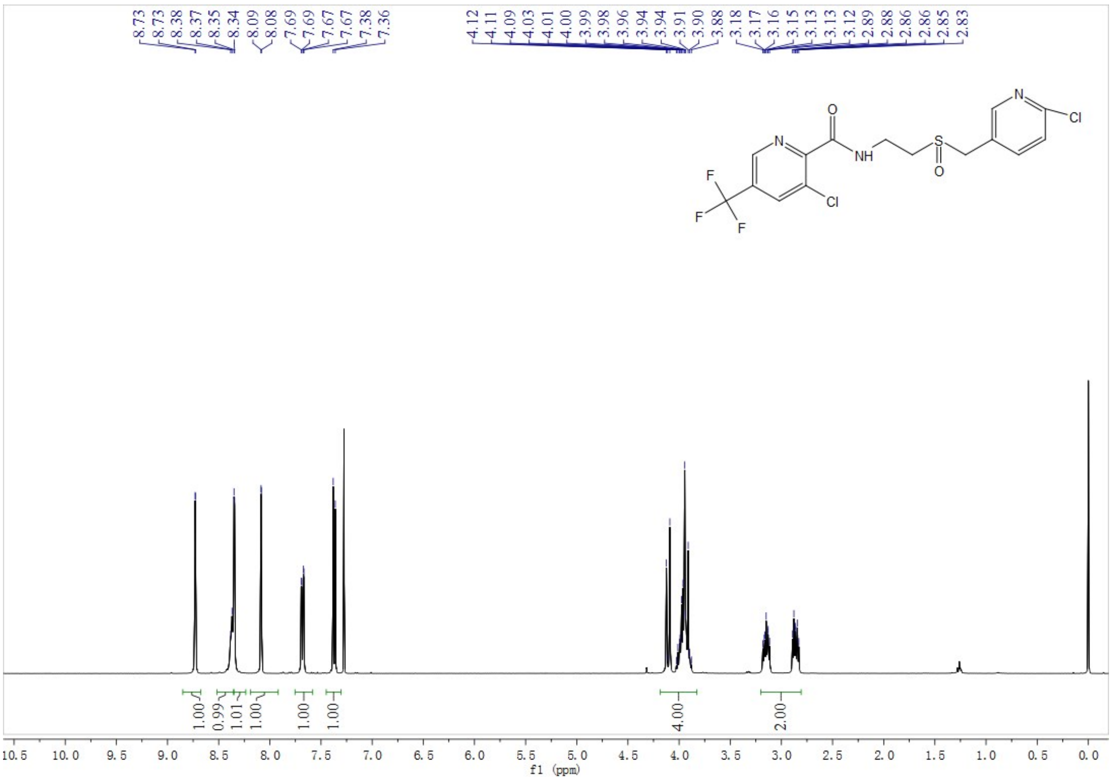

Fig. S139 ^{1}H NMR spectra of compound **G11**

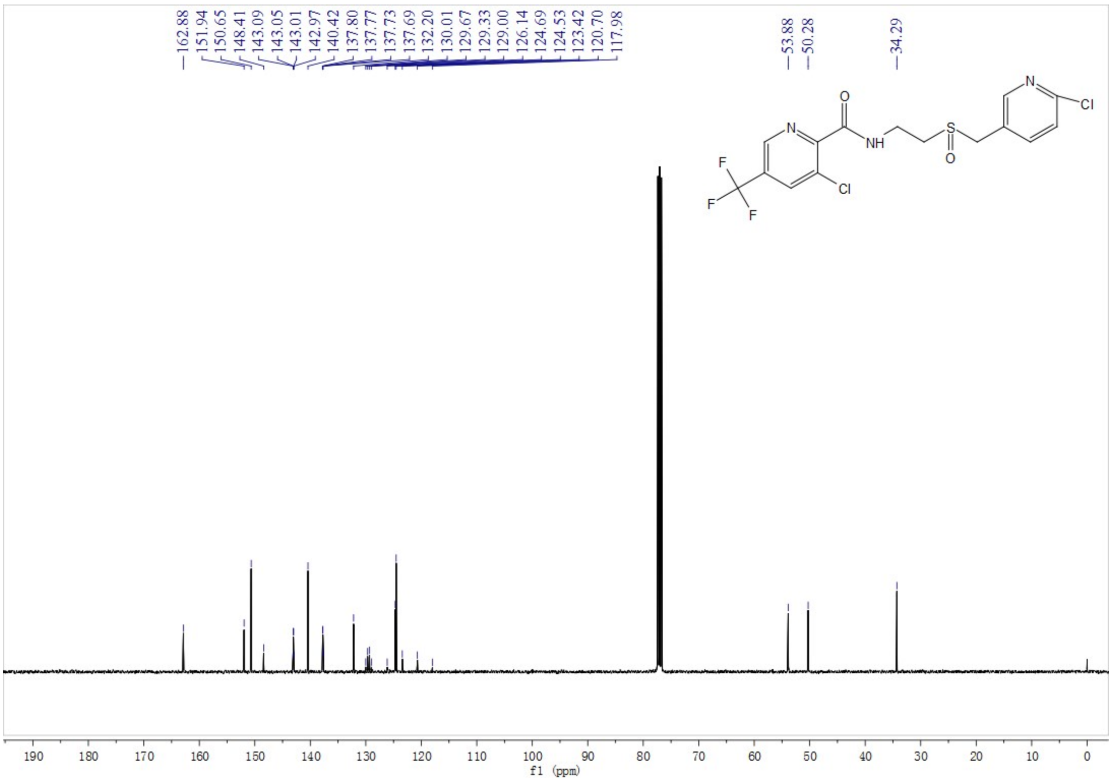

Fig. S140 ^{13}C NMR spectra of compound **G11**

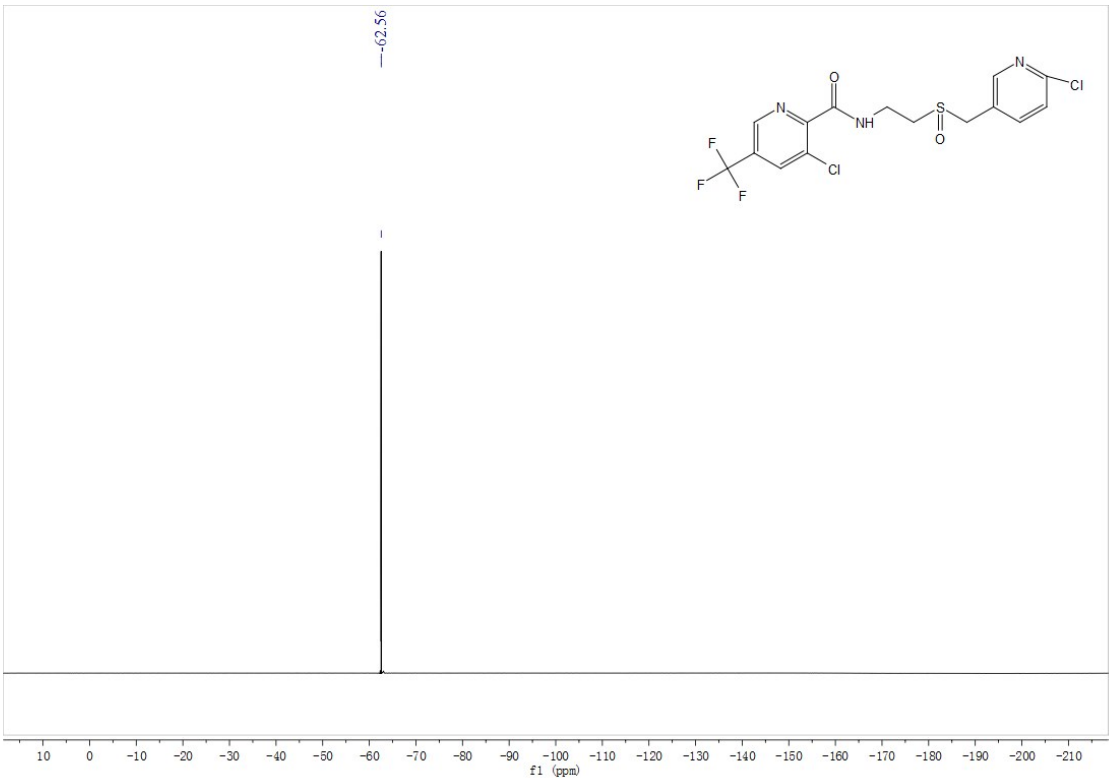

Fig. S141 ^{19}F NMR spectra of compound **G11**

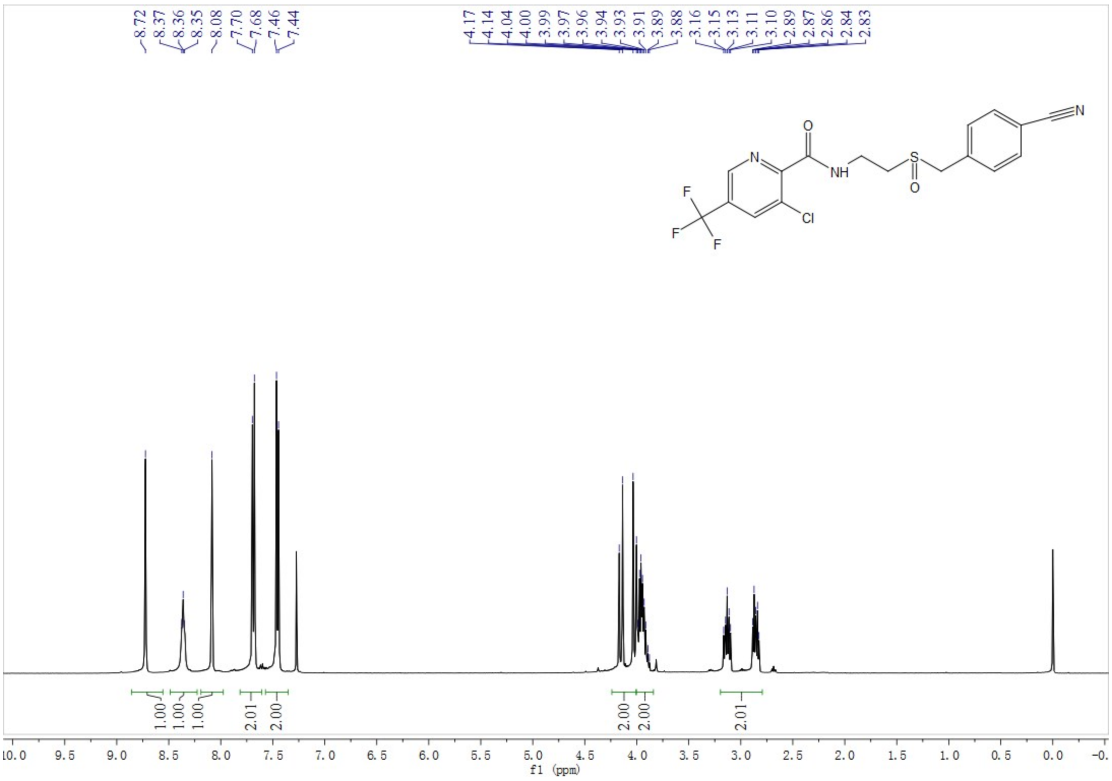

Fig. S142 ^{1}H NMR spectra of compound **G12**

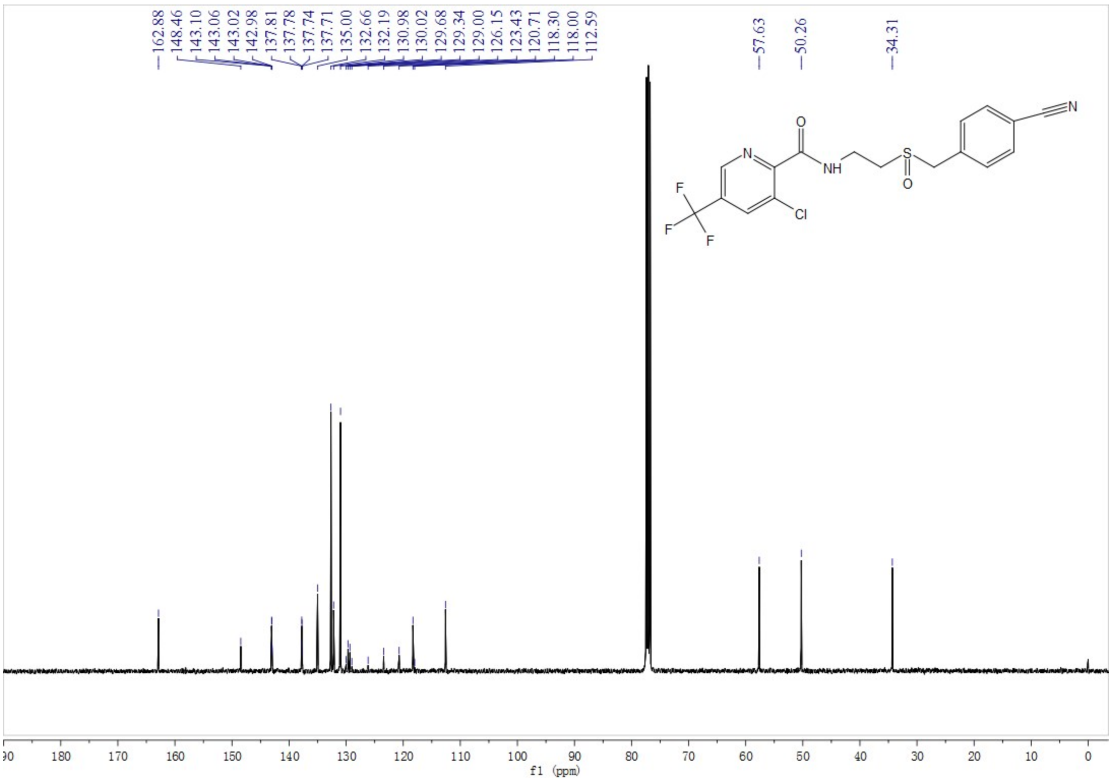

Fig. S143 ^{13}C NMR spectra of compound **G12**

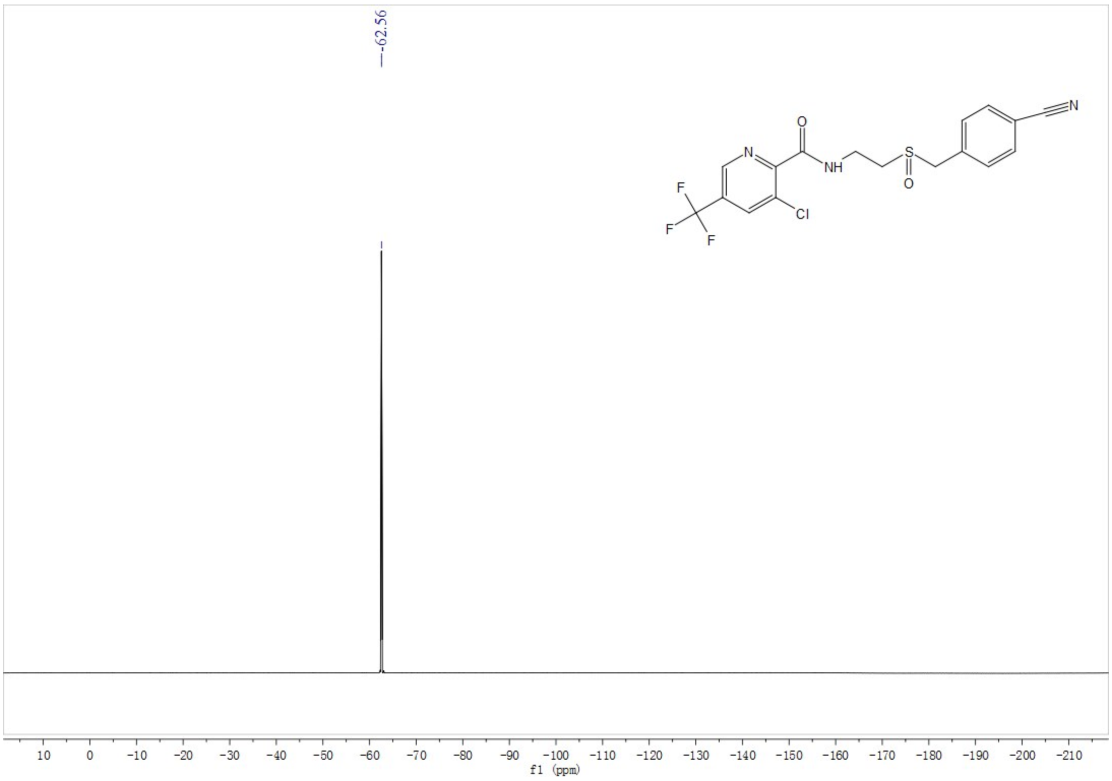

Fig. S144 ^{19}F NMR spectra of compound **G12**

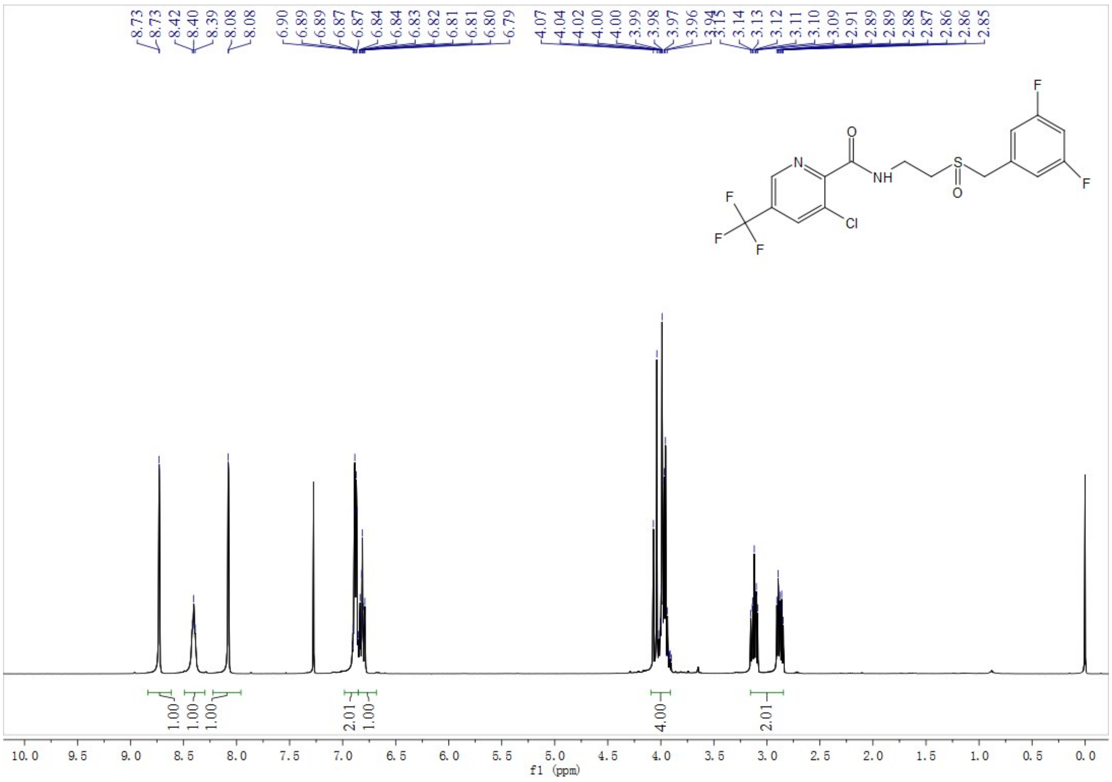

Fig. S145 ^{1}H NMR spectra of compound **G13**

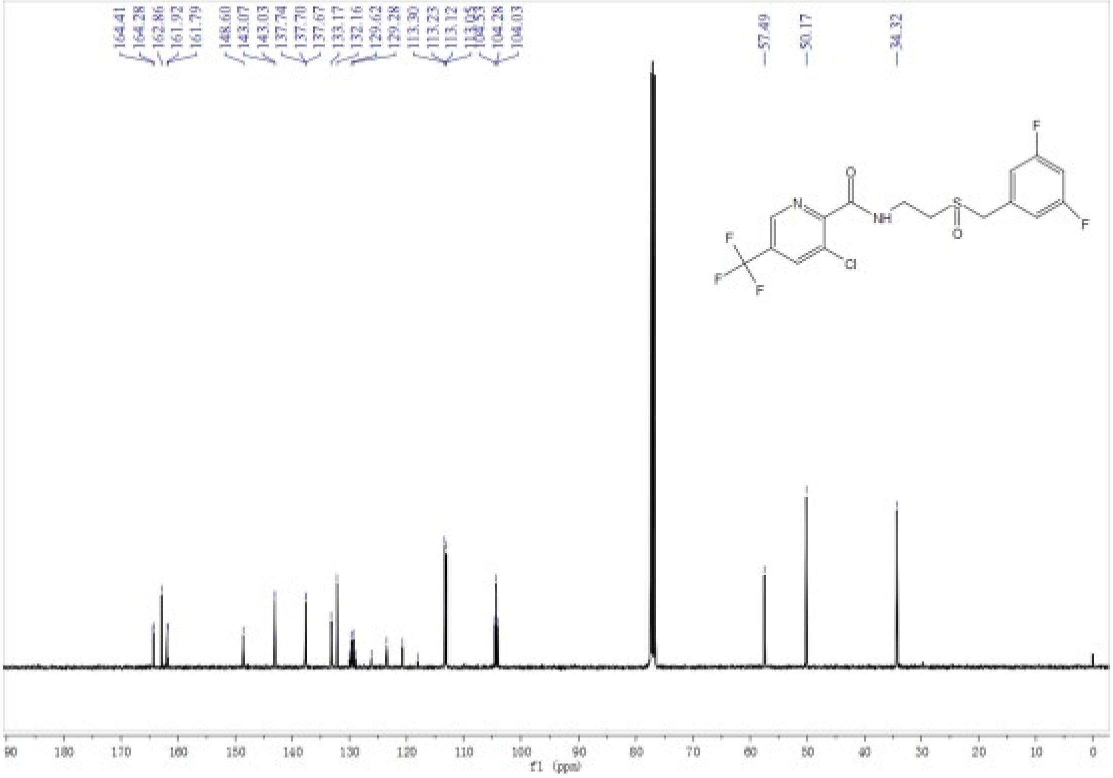

Fig. S146 ^{13}C NMR spectra of compound **G13**

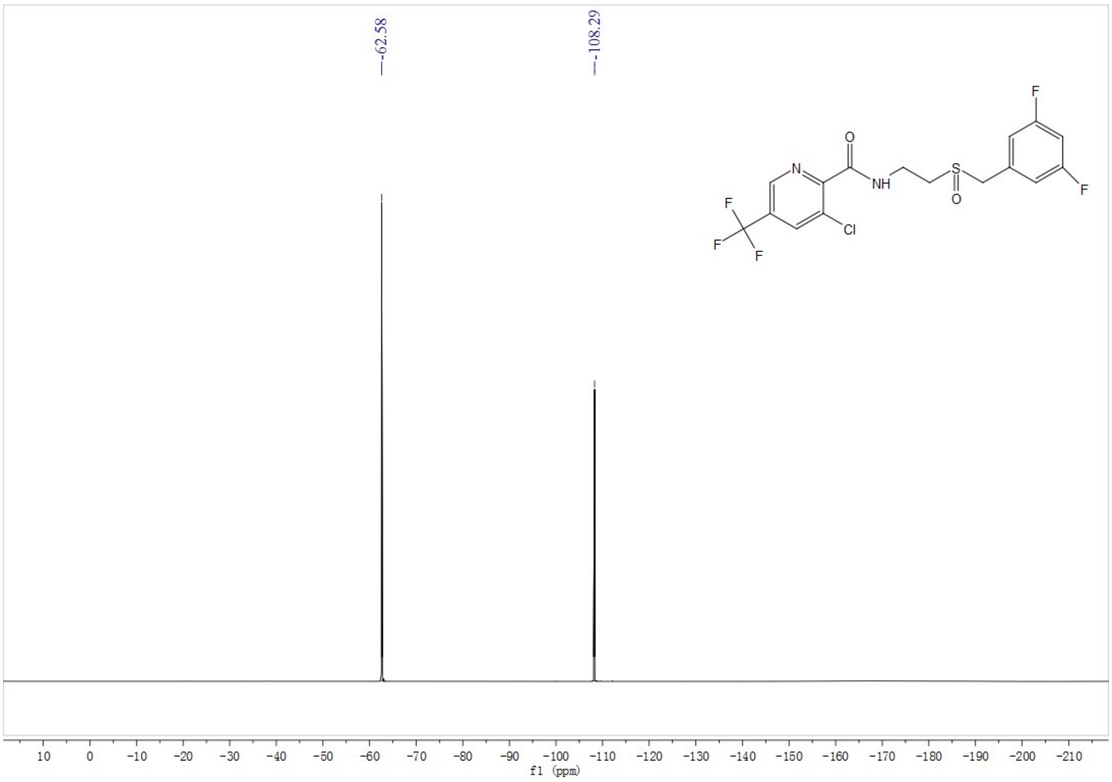

Fig. S147 ^{19}F NMR spectra of compound **G13**

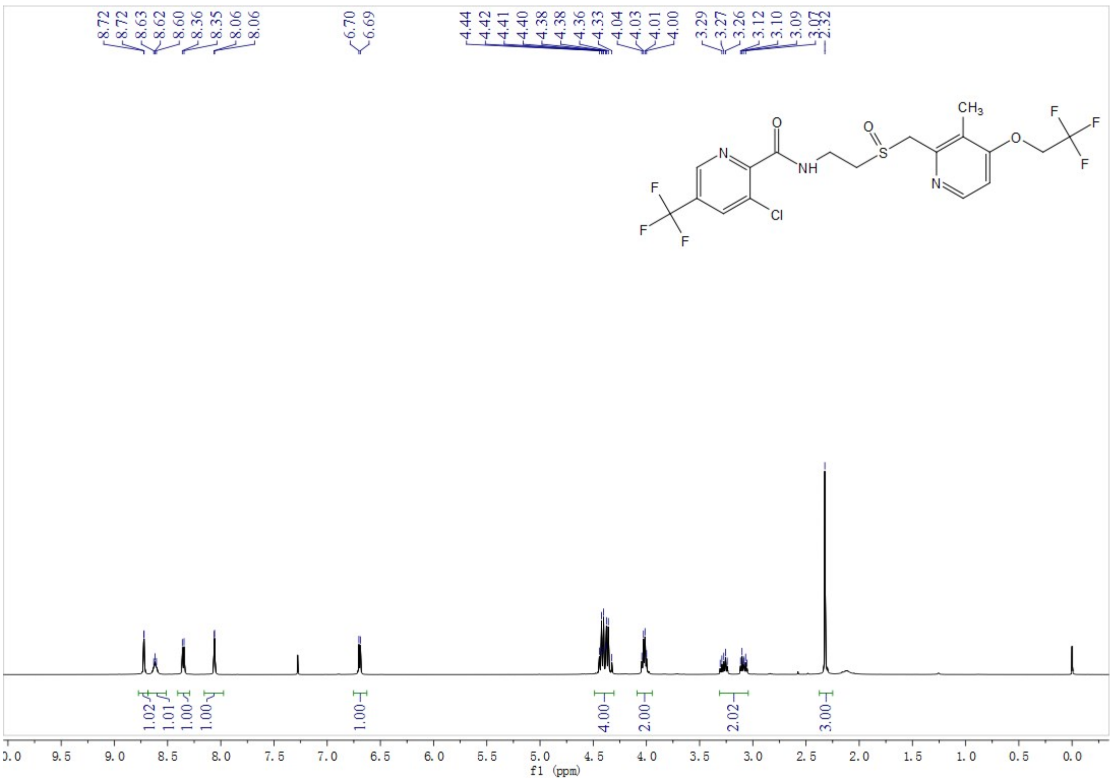

Fig. S148 ^{1}H NMR spectra of compound **G14**

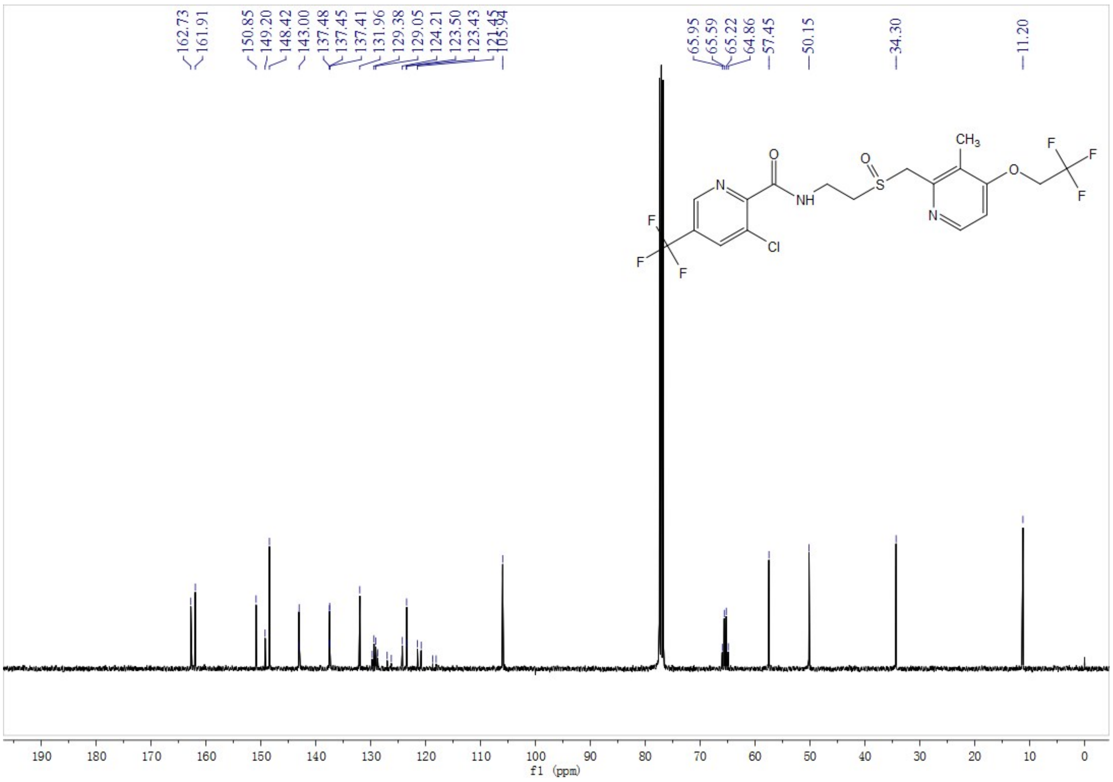

Fig. S149 ^{13}C NMR spectra of compound **G14**

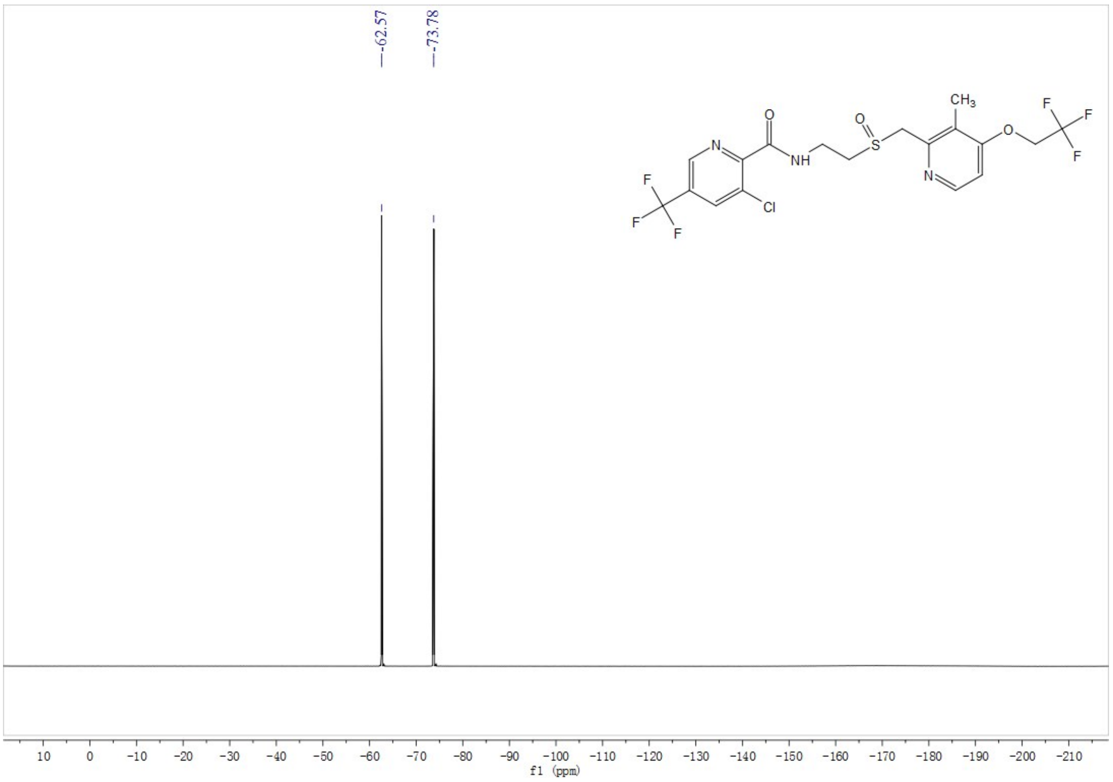

Fig. S150 ^{19}F NMR spectra of compound **G14**

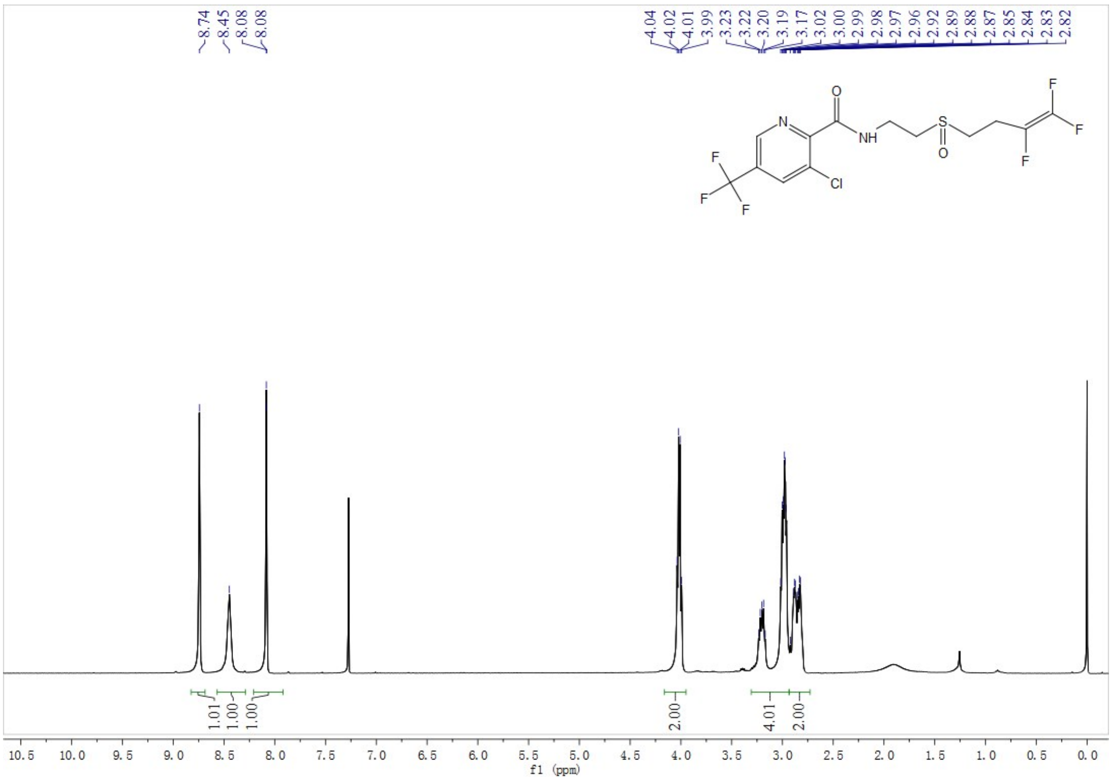

Fig. S151 ^{1}H NMR spectra of compound **G15**

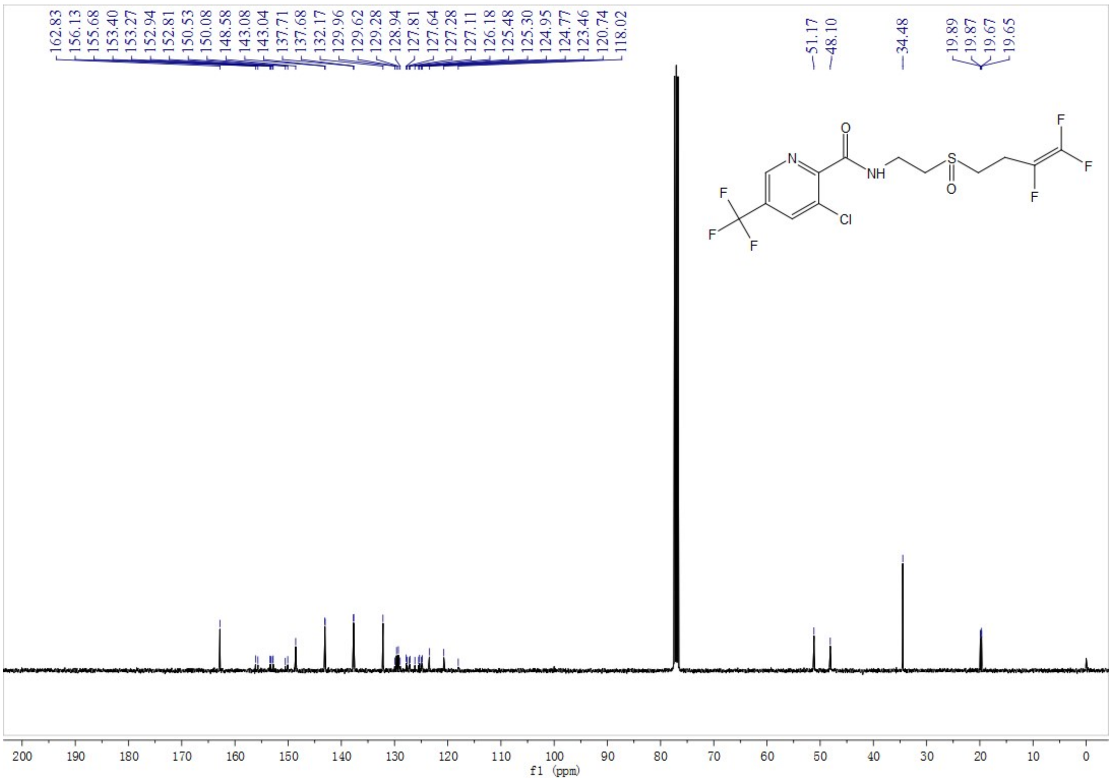

Fig. S152 ^{13}C NMR spectra of compound **G15**

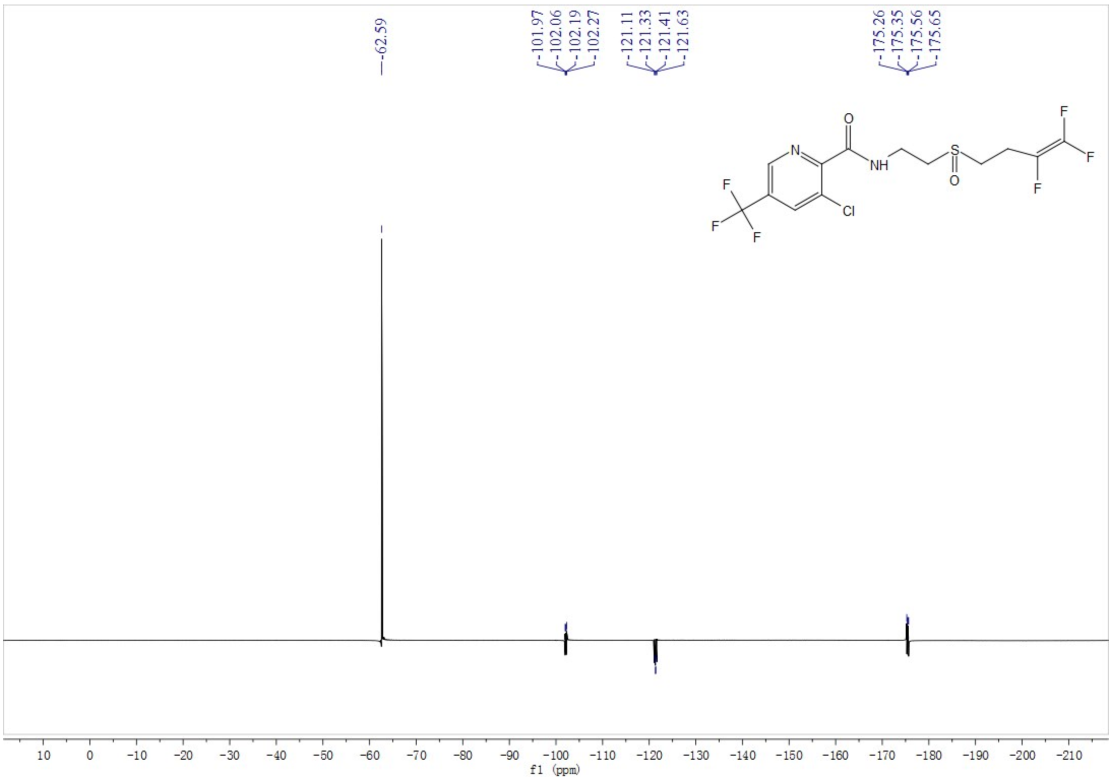

Fig. S153 ^{19}F NMR spectra of compound **G15**

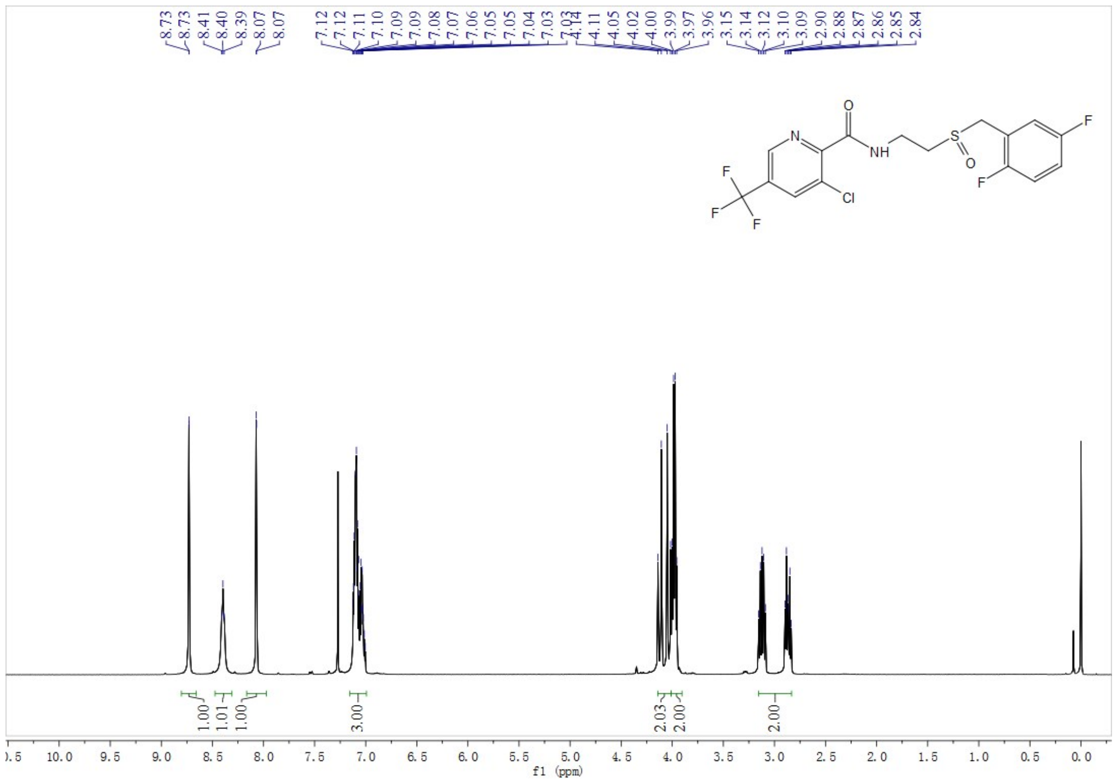

Fig. S154 ^{1}H NMR spectra of compound **G16**

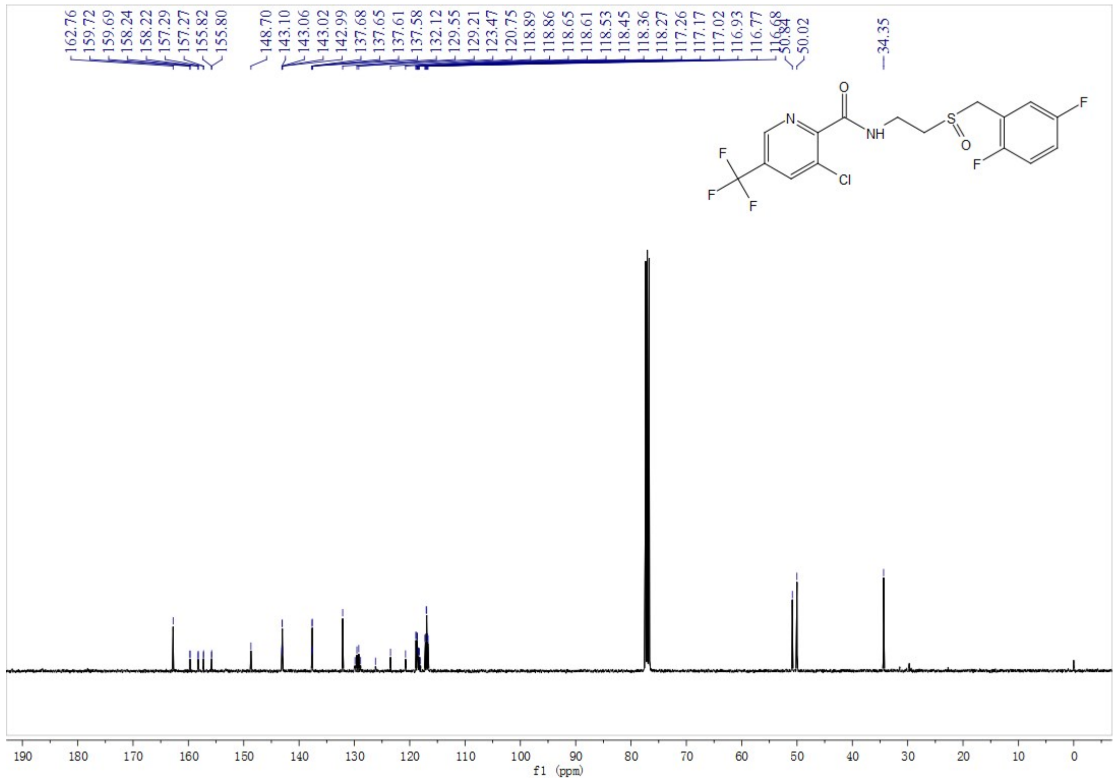

Fig. S155 ^{13}C NMR spectra of compound **G16**

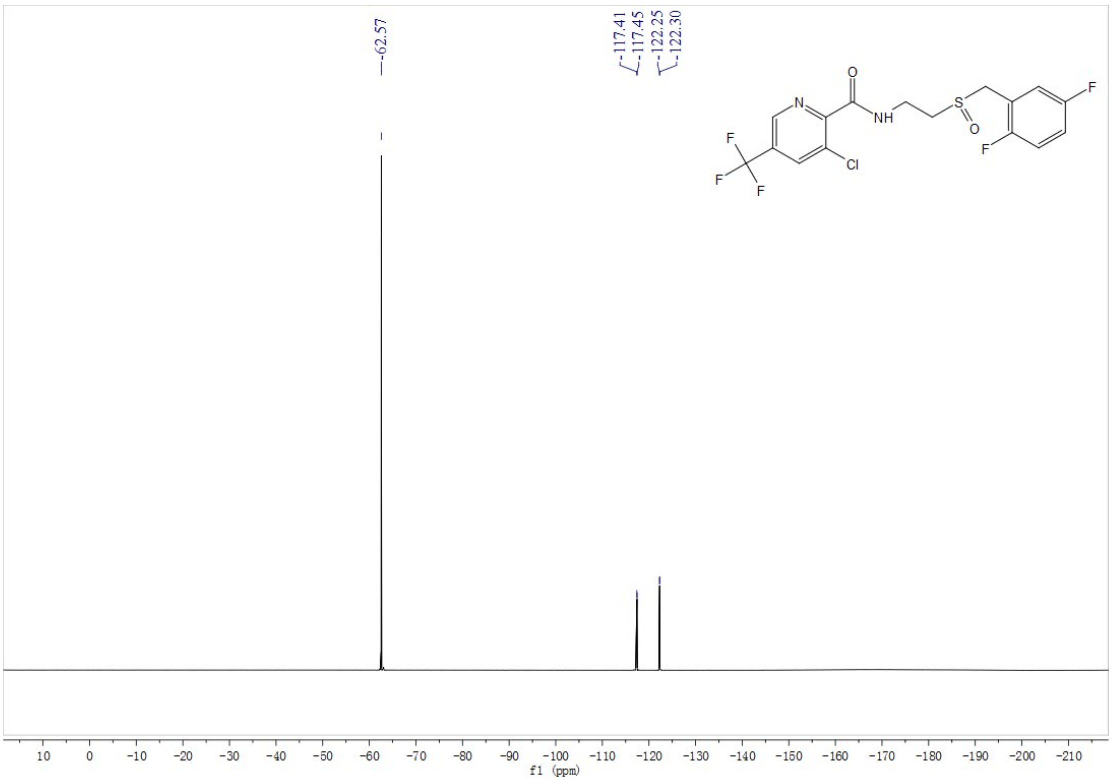

Fig. S156 ^{19}F NMR spectra of compound **G16**
